# Supplementary material for: Unravelling key enzymatic steps in C-ring cleavage during angucycline biosynthesis
Source: Commun Chem. 2023 Dec 18;6:281. doi: 10.1038/s42004-023-01059-1 (PMC10728087; doi:10.1038/s42004-023-01059-1)
Supplement: Supplementary file 2 — Supplementary Information [file 42004_2023_1059_MOESM2_ESM.pdf]

Supplementary Information

Unravelling key enzymatic steps in C-ring cleavage during angucycline biosynthesis

Somayah S. Elsayed[1,7,*], Helga U. van der Heul[1,7], Xiansha Xiao[2], Aleksi Nuutila[3], Laura R. Baars[4], Changsheng Wu[5], Mikko Metsä-Ketelä[3], Gilles P. van Wezel[1,6,*]

[1]Department of Molecular Biotechnology, Institute of Biology, Leiden University, Sylviusweg 72, 2333BE, Leiden, The Netherlands

[2]Department of Structural Biology, Van Andel Institute, Grand Rapids, Michigan, United States

[3]Department of Life Technologies, University of Turku, Tykistönkatu 6, FIN-20014, Turku, Finland

[4]Department of Systems Pharmacology and Pharmacy, Leiden Academic Centre for Drug Research, Leiden University, Einsteinweg 55, 2333CC, Leiden, The Netherlands

[5]State Key Laboratory of Microbial Technology, Institute of Microbial Technology, Shandong University, 266237, Qingdao, P.R. China

[6]Department of Microbial Ecology, Netherlands Institute of Ecology (NIOO-KNAW), Droevendaalsesteeg 10, 6708PB, Wageningen, The Netherlands

[7]These authors contributed equally

*Correspondence: s.elsayed@biology.leideuniv.nl (S.S.E), g.wezel@biology.leidenuniv.nl (G.P.vW.)

Supplementary Tables

Table S1. Functional annotation of the gene products of the *lug* gene cluster in *Streptomyces* sp. QL37

Protein	Gene Annotation	Aa	Putative function	Nearest homologue	Identity (%)	Coverage(%)	Accession
Orf1	WP_187355741.1	378	Hypothetical protein	*Streptomyces mediolani*	97,4	100	WP_030801858.1
Orf2	WP_104791672.1	264	GntR-like regulator, Nudix hydrolase	*Streptomyces pratensis*	98,5	100	MBD2831978.1
Orf3	WP_203186292.1	143	Hypothetical protein	*Streptomyces pratensis*	84,6	100	MBD2831977.1
LugM	WP_104785779.1	199	NADPH-dependent FMN reductase	*Streptomyces* sp. M3	91.4	100	WP_129263039.1
LugRI	WP_104785781.1	280	XRE family transcriptional regulator	*Streptomyces* sp. M3	88.2	100	WP_129263041.1
LugRII	WP_104785782.1	223	LuxR family transcriptional regulator	*Streptomyces* sp. W007	52.1	97	EHM27498.1
LugRIII	WP_104785784.1	239	TetR family transcriptional regulator	*Streptomyces* sp. L-9-10	63.1	96	RYJ29277.1
LugTI	WP_104785785.1	493	Transporter	*Streptomyces* sp. W007	76	98	WP_032792571.1
LugX	WP_104785787.1	144	Hypothetical protein	---	---	---	---
LugN	WP_104791674.1	346	O-methyltransferase	*Streptomyces* sp. CB00072	71.2	99	WP_073867019.1
LugOI	WP_104785789.1	490	FAD-dependent Monooxygenase	*Streptomyces* scopuliridis	81.4	100	WP_030349271.1
LugF	WP_104785790.1	109	Cyclase	*Streptomyces scopuliridis*	83.	98	WP_030349270.1
LugA	WP_104785792.1	427	Polyketide-α-ketoacyl synthase II	*Streptomyces scopuliridis*	87.3	100	WP_030349269.1
LugB	WP_104785793.1	407	Polyketide-β-ketoacyl synthase	*Streptomyces Scopuliridis*	80.3	99	WP_030349268.1
LugC	WP_104785795.1	91	Acyl carrier protein	*Streptomyces scopuliridis*	70.8	98	WP_030349267.1
LugD	WP_104785796.1	262	Ketoacyl reductase	*Streptomyces scopuliridis*	85.1	100	WP_030349266.1
LugE	WP_104785798.1	527	Cylclase	*Streptomyces scopuliridis*	81.3	100	WP_030349265.1
LugOII	WP_104785800.1	656	FAD dependent Monooxygenase-SDR family reductase	*Streptomyces* sp. CB00072	76.4	100	WP_073867012.1
LugG	WP_107528480.1	262	Short chain dehydrogenase (SDR)/reductase	*Streptomyces* sp. b84	77.	98	WP_097910307.1
LugH	WP_104791675.1	527	Acyl-coA carboxylase subunit beta	*Streptomyces* sp. w007	87.1	97	WP_050987785.1
LugI	WP_104785801.1	79	Acyl-CoA carboxylase subunit epsilon	*Streptomyces* sp. NBRC 110028	53.8	94	WP_055547361.1
LugTII	WP_104785803.1	417	Transporter	*Streptomyces scopuliridis*	67.7	96	WP_051745235.1

LugJ	WP_104785804.1	211	NAD(P)H dependent FMN reductase	*Streptomyces* sp. Ru71	63.9	90	WP_103781763.1
LugOIII	WP_104785806.1	214	Antibiotic biosynthesis monooxygenase	*Streptomyces* sp. CB00072	62.3	99	WP_073867008.1
LugK	WP_104785807.1	245	4'-phosphopantetheinyl transferase superfamily protein	*Goodfellowiella* sp. AN110305	51.1	94	WP_104785807.1
LugRIV	WP_104785809.1	267	Response regulator transcription factor	*Streptomyces violens*	62	91	WP_078601061.1
LugTIII	WP_104785811.1	458	Transporter	*Streptomyces violens*	75.5	84	WP_078601068.1
LugL	WP_104785812.1	307	Alpha/beta hydrolase	*Streptomyces scopuliridis*	69	94	WP_030349255.1
LugOIV	WP_104785814.1	275	SDR family NAD(P)-dependent oxidoreductase	*Streptomyces* sp. TSRI0281	75.6	100	WP_107468115.1
LugRV	WP_104785815.1	646	AfsR/SARP family transcriptional regulator	*Streptomyces scopuliridis*	60.8	93	WP_078490261.1
LugOV	WP_146111254.1	229	Hypothetical protein	*Streptomyces zhaozhouensis*	57.5	90	WP_141514657.1
Orf 4	WP_104785818.1	425	Phosphoribosyltransferase	*Streptomyces* sp. ADI93-02	93	100	WP_124276433.1
Orf 5	WP_104791676.1	989	Alpha_L-rhamnosidase	*Streptomyces* sp. wa22	93	96	WP_147960798.1
Orf 6	WP_104785820.1	868	glycoside hydrolase family 127	*Streptomyces* sp. TRM S81-3	96	76.1	WP_188185628.1
Orf 7	WP_104785821.1	1133	glycoside hydrolase family 106 (alpha L-rhamnosidase)	*Streptomyces* sp. TRM S81-3	100	74.6	WP_188185460.1
Orf 8	WP_104785823.1	1092	glycoside hydrolase family 78	*Streptomyces* sp. TRM S81-3	99	77.7	WP_188185461.1
Orf 9	WP_104785824.1	184	GNAT-family acetyltransferase	*Streptomyces* sp. For3	98	81.8	WP_202079774.1
Orf 10	WP_104785825.1	507	Sugar ABC-transport system	*Streptomyces* sp. wa22	100	97.8	WP_147960792.1

Table S2. Strains used in this study

**Bacterial strains**	**Characteristics**	**Reference**
Escherichia coli JM109	*end*A1, *rec*A1, *gyr*A96, *thi, hsd*R17 (rk–, mk+), *rel*A1, *sup*E44, λ–, Δ(*lac-pro*AB), [F´, *tra*D36, *pro*AB, *lac*IqZΔM15], lDE3	[1]
Escherichia coli ET12567 / PUZ8002	Methylation deficient strain with deletion in *dam*, *dcm* an *hdsM* genes<br>with chloramphenicol resistance marker. The strain harbors a non-transmittable plasmid pUZ8002 that contains the *tra* genes, required for conjugation.	[2]
Streptomyces sp. QL37	lugdunomycin and angucycline producer	[3,4]
Streptomyces sp. QL37 Δ*lugOI*	QL37 with in-frame deletion of *lugOI*	This study
Streptomyces sp. QL37 Δ*lugOII*	QL37 with in-frame deletion of *lugOII*	[5]

Streptomyces sp. QL37 Δ*lugOIII*	QL37 with in-frame deletion of *lugOIII*	This study
Streptomyces sp. QL37 Δ*lugOIV*	QL37 with in-frame deletion of *lugOIV*	This study
Streptomyces sp. QL37 Δ*lugOV*	QL37 with in-frame deletion of *lugOV*	This study
Streptomyces sp. QL37 Δ*lug-pks*	QL37 deleted for *lugA–OII*	[3]
Streptomyces sp. QL37 Δ*lugOIII*+pO*lugOIII*	*Streptomyces* sp. Δ*lugOIII*::pWHM3-*oriT- ermE** -*lugOIII*	This study
Streptomyces sp. QL37 Δ*lugOV*+pO*lugOV*	*Streptomyces* sp. Δ*lugOV*::pWHM3-*oriT- ermE** -*lugOV*	This study
Streptomyces sp. QL37 Δ*lugOI*+pO*pgaE*	*Streptomyces* sp. Δ*lugOI*::pS-GK-*pgaE*	This study

Table S3. List of primers used in this study

**Gene**	**Application**	**Sequence (5' -> 3')**[a]	**Position**[b]	**Length Gene(nt)**
lugOI	Left flank	CGATAAGCTTAGATCTTCCATCCCGCCTTCTGAAGAC	-3/+1467	1470
		CGATTCTAGAGACTACGACTGATGCGTCCATGTG		
	Right flank	CGATTCTAGAGGACCGGCCAGGTGAGATTC		
		CGATCTGCAGGCCGGTCGTTCTGCTTGGTC		
	Verification mutant	CGAGTGCCATGCCCTGATGAAG		
		GCGACGATCAGAGTGCTTGG		
lugOII	Left flank	CGATAAGCTTGGTACCGAGCTGTGGCTGGACGTGATCAAC	+9/+1967	1968
		CGATTCTAGACGTGGTGCCCACGGGTCAGC		
	Right flank	CGATTCTAGAGGCGGGACGCTCCTCGGATG		
		CGATGAATTCGGTGAGGGCCGGCGAGTAGG		
	Verification mutant	TCGACCCACACCACGGAATC		
		TTCCTCGCCGATGTGCTTGG		
lugOIII	Left flank	CGATAAGCTTGGTACCCGGGTTCGCGGAAAGTGAAG	+24/+624	642
		CGATTCTAGACGCGAGAGAGATCTGACGAG		
	Right flank	CGATTCTAGACTGCACGGCATCCGGTGATCG		
		CGATGAATTCGTCGAACTCCTTGCGGGTGAGC		
	Verification mutant	TGGTGACGCCCGAGTACAAC		
		GACATGACCTTGCGGAGCAC		
	Complementation mutant	CGATGGATCCATATGCCTCGTCAGATCTCTCTC	+1/+651	
		CGATTCTAGAAGCTCGTCGATCACCGGATG		
lugOIV	Left flank	CAGTGAATTCCACCAGGGTGCCGAGGTAGC	-32/+811	825
		CTAGTCTAGATCGACGAGCTGACCCGAGAAC		

	Right flank	CTAGTCTAGATTCGCACGCGGTCGGATGAG		
		CTAGGGATCCGGCCGCCCAGATGAAGGGTG		
	Verification mutant	GTATCCGGCCCGCATGCCTC		
		CCCTCGCCGACGCACACTTC		
lugOV	Left flank	CTAGAAGCTTAGCAGGTCGGCCAAGGGCAG	-24/+764	687
		CTAGTCTAGATGCGCGTCCTCACGACCAAC		
	Right flank	CTAGTCTAGATGCGCGACTCCCATGAATGGATACG		
		CTAGGAATTCGCCCGGCTCCTCGAACAGGTG		
	Verification mutant	GATAGGCGGGCCGCATGTGG		
		CCCAGCTCCGGGTGGTAGGG		
	Complementation mutant	CTAGGGATCCGCGCAGGCCCTTCCGCTCAG	+1/+703	
		CTAGCATATGCCCCGCCCTGTCGACCATCT		

[a] Restriction sites underlined: GAATTC, EcoRI; AAGCTT, HindIII; TCTAGA, XbaI; CTGCAG, PstI; GGATCC, BamHI ; AGATCT, BglII; CATATG, NdeI; ATGCAT, NsiI; CCTAGG, AvrII

[b] Position relative to the translational start site (+1) of the respective genes.

Table S4. LC-MS data processing parameters in MZmine

**MZmine 2.53**	
Mass detection	MS level: 1, Polarity: +, Mass detector: Centroid, Noise level: 2.0E2<br>MS level: 2, Polarity: +, Mass detector: Centroid, Noise level: 0
ADAP Chromatogram Builder	MS level: 1, Polarity: +, Min group size in # of scans: 10, Group intensity threshold: 2.0E2, Min highest intensity: 4.0E2, *m/z* tolerance: 0.002 *m/z* or 10.0 ppm
Smoothing	Filter width: 9
Chromatogram deconvolution	Algorithm: Local minimum search (Chromatographic threshold: 90%, Search minimum in RT range: 0.05, Minimum relative height: 1%, Minimum absolute height: 4.0E2, Minimum ratio of peak top/edge: 2, Peak duration range: 0.03 – 3.00), *m/z* center calculation: median<br>m/z range for MS2 scan pairing: 0.02 Da<br>RT for MS2 scan pairing: 0.05 min
Isotopic peaks grouper	*m/z* tolerance: 0.002 *m/z* or 10.0 ppm, RT tolerance: 0.1 min, Monotonic shape, Maximum charge: 2, Representative isotope: Most intense
Alignment: Join aligner	*m/z* tolerance: 0.002 *m/z* or 10.0 ppm, Weight for *m/z*: 20, RT tolerance: 0.1 min, Weight for RT: 20, Compare isotopic pattern (Isotope *m/z* tolerance: 0.002 *m/z* or 10.0 ppm, Minimum absolute intensity: 4.0E2, Minimum score: 50%)
Gap filling: Peak finder	Intensity threshold: 1%, *m/z* tolerance: 0.002 *m/z* or 10.0 ppm, RT tolerance: 0.1 min
Fragment search	RT tolerance: 0.05 min, *m/z* tolerance of MS2 data: 0.002 *m/z* or 10.0 ppm, Maximum fragment peak height: 50%, Minimum MS2 peak height: 0.0E0
Adduct search	RT tolerance: 0.05 min; Adducts: $[M+Na]^+$, $[M+K]^+$ and $[M+NH_4]^+$; *m/z* tolerance: 0.002 *m/z* or 10.0 ppm; Maximum relative adduct peak height: 3000%
Complex search	Ionization method: $[M+H]^+$, RT tolerance: 0.05 min, *m/z* tolerance: 0.002 *m/z* or 10.0 ppm, Maximum complex peak height: 50%
Duplicate peak filter	Filter mode: Single feature, *m/z* tolerance: 0.002 *m/z* or 10.0 ppm, RT tolerance: 0.05 min

Duplicate peak filter	Filter mode: Old average, *m/z* tolerance: 1.0 *m/z* or 1000.0 ppm, RT tolerance: 0.05 min
Peak list rows filter	RT: 1.0–10.0 min, Keep rows that match all criteria, Reset the peak number ID
**MZmine 2.37.1.IIN 17.7 LS**	
MetaCorrelate	RT tolerance: 0.1 min, Min height: 4.0E2, Noise level: 2.0E2, Correlation grouping (Min data points: 5, Min data points on edge: 2, Measure: Pearson, Min feature shape correlation: 85%)
Ion identity networking	*m/z* tolerance: 0.002 *m/z* or 10.0 ppm, Check: All Features, Min height: 4.0E2, Ion identity library (MS mode: positive, Maximum charge: 2, Maximum molecules/cluster: 3, Adducts: $[M+H]^+$ $[M+Na]^+$ $[M+NH4]^+$, Modifications: $[M-H_2O]$ $[M-2H_2O]$

Table S5. ^1H NMR data of **16**–**21** at 298 K in DMSO-d_6

Position	δ_H, mult. (*J* in Hz)					
	**16**[a]	**17**[a]	**18**[a]	**19**[b]	**20**[a]	**21**[a]
**1**						5.48, t (6.8)
**2**	5.94, m	a: 2.95, d (12.7)	5.80, q (1.4)	5.71, t (2.0)	a: 3.00, d (13.2)	a: 2.10, m
		b: 2.58, dd (12.7, 3.0)			b: 2.67, m	b: 1.76, dd (13.3, 6.8)
**4**	a: 2.67, ddd (18.4, 2.9, 1.4)	a: 2.08, dd (14.6, 3.0)	2.58, s	a:2.99, br d (17.6)	a: 3.04, dd (17.6, 1.8)	2.67, m
	b: 2.56, m	b: 2.00, d (14.6)		b: 1.93, m	b: 2.84, d (17.6)	
**5**	a: 1.97, dd (13.7, 3.4)	a: 1.91, td (13.7, 3.0)	3.80, dd (11.0, 4.9)	a: 1.67, dt (14.0, 3.2)		
	b: 1.80, dt (13.7, 3.9)	b: 1.76, dt (13.7, 3.5)		b: 1.56, td (14.0, 4.4)		
**6**	a: 2.56, m	a: 2.53, m	2.02, m	a: 2.36, ddt (14.0, 4.4, 3.2)	7.55, s	7.49, m
	b: 1.45, dt (13.6, 3.9)	b: 1.38, dt (13.7, 3.5)		b: 1.92, m		
**6a**				3.27, br t (3.2)		
**7**	4.80, d (5.3)	4.81, d (5.4)				
**9**	7.26, dd (7.8, 1.6)	7.29, dd (8.0, 1.4)	7.27, m	7.34, dd (8.3, 1.1)	7.49, m	7.49, m
**10**	7.39, t (7.8)	7.40, dd (8.0, 7.8)	7.70, t (7.9)	7.82, dd (8.3, 7.4)	7.79, m	7.79, m
**11**	7.41, dd (7.8, 1.6)	7.44, dd (7.8, 1.4)	7.23, m	7.43, dd (7.4, 1.1)	7.53, dd (7.6, 1.0)	7.72, dd (7.7, 1.0)
**12a**			3.43, d (10.5)			
**12b**			3.13, d (10.5)	2.33, s		
**3-Me**	1.94, d (1.4)	1.27, s	1.91, d (1.4)	1.90, t (1.2)	1.34, s	1.30, s
**8-MeO**	3.86, s	3.86, s			3.93, s	3.93, s
**3-OH**		5.60, s			4.91, br s[d]	4.47, br s[d]
**1-OH**						4.96, br s
**4a-OH**	4.89, s	5.49, s	6.33/4.32, br s[c]	4.56, s		
**6a-OH**	5.16, s	5.01, s	6.33/4.32, br s[c]			
**7-OH**	5.49, d (5.3)	5.56, d (5.4)				
**8-OH**			11.47, br s	12.15, s		
**12a-OH**				5.56, s		

[a] ^1H 600 MHz
[b] ^1H 850 MHz
[c,d] No correlations to assign either of them

Table S6. ^{1}H NMR data of **23**–**27** at 298 K in DMSO-d_6

**Position**	δ_H, **mult. (*J* in Hz)**				
	**23**[a]	**24**[a]	**25**[b]	**26**[a]	**27**[a]
**1**				5.43, q (5.5)	
**2**	7.27, d (1.6)	2.32, S	2.41, d (1.4)	a: 2.09, ddd (13.6, 5.9, 1.3)<br>b: 1.93, ddd (13.6, 5.1, 1.1)	a: 2.54, m<br>b: 2.40, d (16.4)
**4**	7.47, dd (1.7, 1.0)	a: 3.03, d (13.1)<br>b: 2.95, d (13.1)	a: 3.07, d (13.2)<br>b: 2.88, d (13.2)	a: 2.90, d (16.3)<br>b: 2.73, d (16.3)	2.54, m
**5**	7.89, d (8.7)	7.74, m	7.42, s	7.18, d (7.9)	a: 2.61, m<br>b: 2.13, dd (17.3, 5.3)
**6**	8.02, d (8.7)	8.06, d (7.9)		7.78, d (7.9)	a: 2.72, m<br>b: 1.67, dt (5.3, 13.2)
**7**					5.33, s
**9**	7.16, dd (8.5, 1.0)	7.30, dd (8.4, 1.2)	7.41, m	7.12, dd (8.5, 1.0)	7.27, d (7.3)
**10**	7.65, dd (8.5, 7.6)	7.74, m	7.84, t (7.5)	7.60, dd (8.5, 7.6)	7.44, t (7.3)
**11**	7.32, m	7.64, dd (7.5, 1.2)	7.81, d (7.5)	7.20, m	7.15, d (7.3)
**12**	6.85, d (8.5)			6.35, d (7.5)	
**12b**		8.02, d (1.7)			
**3-Me**	2.48, d (1.0)	1.18, s		1.30, s	1.19, s
**8-MeO**	3.89, s			3.85, s	3.85, s
**1-OH**				5.07, d (5.6)	
**3-OH**			4.84, s	4.50, s	4.82, br s
**6-OH**			12.10, s		
**7-OH**					5.94, br s
**8-OH**		12.4, br s	12.08, s		
**12-OH**	6.69, d (8.5)			5.76, d (7.5)	
**1′**	5.10, d (7.7)				
**2′**	3.69, ddd (9.0, 7.7, 3.0)				
**3′**	3.42, t (9.0)				
**4′**	3.30, m				
**5′**	3.55, m				
**6′**	a: 3.81, m<br>b: 3.55, m				
**2′-OH**	6.25, d (3.0)				
**3′-OH**	5.28, s				
**4′-OH**	5.19, d (5.0)				
**6′-OH**	4.73, t (5.6)				

[a] ^{1}H 600 MHz
[b] ^{1}H 850 MHz

Table S7. ^{13}C NMR data of **16**–**21** at 298 K in DMSO-d_6

**Position**	δ_C, **type**					
	**16**[b]	**17**[b]	**18**[b]	**19**[b]	**20**[c]	**21**[c]
**1**	189.8, C	203.3, C	196.4, C	197.0, C	196.8, C	64.2, CH
**2**	125.8, CH	55.9, CH_2	124.3, CH	125.2, CH	52.6, CH_2	43.7, CH_2

**3**	157.7, C	74.0, C	155.3, C	160.8, C	71.2, C	66.4, C
**4**	47.0, CH_2	49.3, CH_2	42.1, CH_2	41.1, CH_2	37.2, CH_2	38.1, CH_2
**4a**	72.4, C	74.0, C	74.1, C	70.9, C	135.5, C	129.6, C
**5**	32.5, CH_2	31.8, CH_2	69.5, CH	35.3, CH_2	159.8, C	160.4, C
**6**	28.2, CH_2	27.2, CH_2	33.3, CH_2	18.6, CH_2	112.0, CH	110.3, CH
**6a**	74.1, C	72.3, C	76.3, C	53.5, CH	ND	ND
**7**	67.6, CH	66.5, CH	201.1, C	202.3, C	180.3, C	181.3, C
**7a**	131.6, C	132.2, C	114.5, C	117.2, C	119.5, C	119.5, C
**8**	157.4, C	157.2, C	161.0, C	160.4, C	159.1, C	159.1, C
**9**	115.4, CH	115.8, CH	122.3, CH	122.8, CH	117.5, CH	117.5, CH
**10**	128.2, CH	128.3, CH	136.4, CH	137.6, CH	135.3, CH	135.3, CH
**11**	117.4, CH	117.7, CH	116.1, CH	118.5, CH	118.1, CH	118.7, CH
**11a**	132.8, C	132.0, C	137.1, C	135.0, C	137.0, C	137.0, C
**12**	188.2, C	186.7, C	194.3, C	197.4, C	182.2, C	184.4, C
**12a**	140.7, C	134.3, C	49.2, CH	80.6, C	125.1, C	122.4, C
**12b**	148.1, C	153.1, C	47.6, CH	57.2, CH	136.5, C	143.6, C
**3-Me**	23.5, CH_3	29.9, CH_3	23.3, CH_3	24.3, CH_3	29.7, CH_3	29.7, CH_3
**8-MeO**	55.5, CH_3	55.6, CH_3			56.0, CH_3	56.0, CH_3

[b] ^{13}C 213 MHz
[c] ^{13}C chemical shifts inferred from multiplicity-edited HSQC and HMBC spectra
ND not determined

Table S8. ^{13}C NMR data of **23**–**27** at 298 K in DMSO-d_6

Position	δ_C, type				
	**23**[c]	**24**[a]	**25**[b]	**26**[b]	**27**[a]
**1**	155.1, C	172.3, C	172.4, C	63.8, CH	194.2, C
**2**	113.4, CH	45.6, CH_2	46.6, CH_2	46.0, CH_2	50.8, CH_2
**3**	138.4, C	70.1, C	71.0, C	67.7, C	68.8, C
**4**	121.8, CH	46.5, CH_2	39.4, CH_2	44.9, CH_2	44.8, CH_2
**4a**	136.9, C	146.3, C	140.7, C	141.6, C	162.8, C
**5**	128.2, CH	136.6, CH	131.5, CH	129.2, CH	29.2, CH_2
**6**	122.8, CH	125.7, CH	155.8, C	125.3, CH	25.3, CH_2
**6a**	131.1, C	130.5, C	111.2, C	132.7, C	64.6, C
**7**	182.8, C	187.9, C	189.9, C	183.6, C	62.8, CH
**7a**	119.4, C	115.5, C	116.1, C	120.1, C	128.6, C
**8**	158.8, C	161.2, C	161.5, C	159.4, C	156.4, C
**9**	111.6, CH	123.7, CH	124.5, CH	112.0, CH	115.4, CH
**10**	134.1, CH	136.6, CH	137.3, CH	134.4, CH	129.5, CH
**11**	122.0, CH	118.7, CH	119.1, CH	122.0, CH	117.8, CH
**11a**	145.9, C	132.9, C	133.2, C	146.6, C	132.7, C
**12**	64.5, CH	181.7, C	186.5, C	63.1, CH	193.8, C
**12a**	138.0, C	132.1, C	111.8, C	141.1, C	56.5, C
**12b**	119.5, C	128.7, CH	157.2, C	136.9, C	127.4, C
**3-Me**	21.2, CH_3	26.6, CH_3	26.4, CH_3	29.9, CH_3	29.0, CH_3
**8-MeO**	55.6, CH_3			55.9, CH_3	55.7, CH_3
**1′**	102.0, CH				
**2′**	73.6, CH				
**3′**	75.8, CH				
**4′**	69.5, CH				
**5′**	77.3, CH				

**6′**	60.5, CH_2

[a] ^{13}C 150 MHz
[b] ^{13}C 213 MHz
[c] ^{13}C chemical shifts inferred from multiplicity-edited HSQC and HMBC spectra

Supplementary Figures

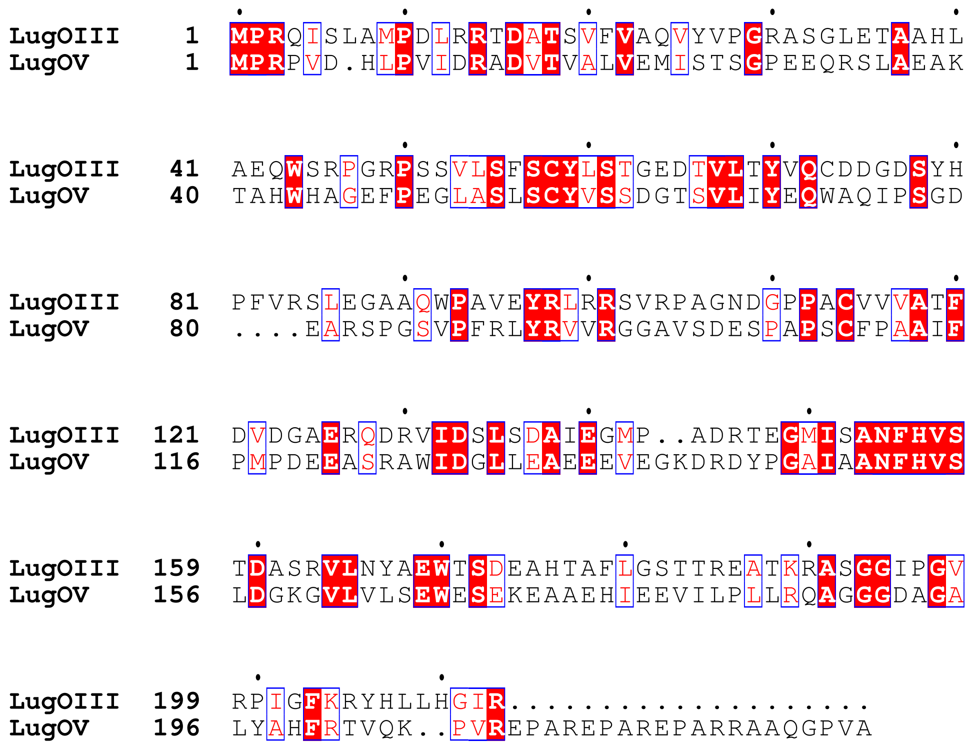

Fig. S1. Amino acid sequence alignment of LugOIII and LugOV. White letters with red background indicate identical amino acids, blue boxed letters indicate similar amino acids. Every tenth amino acid is indicated with a black dot

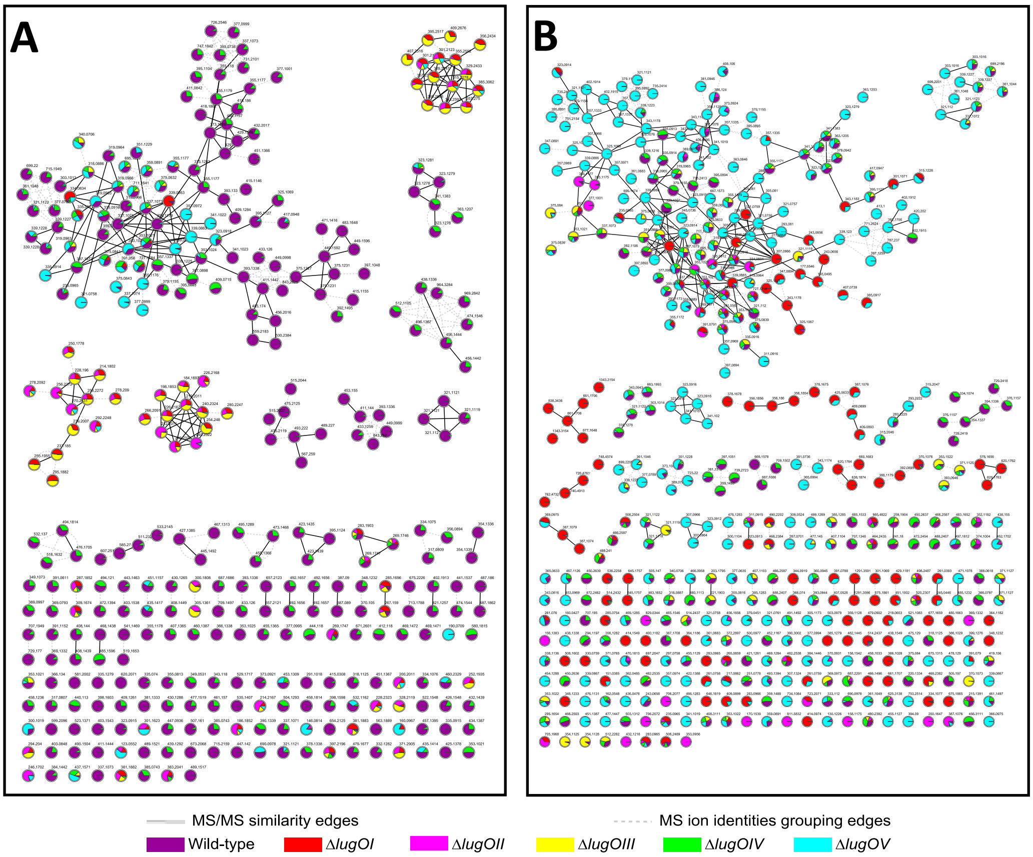

Fig. S2. Molecular network of the ions detected in the extracts of *Streptomyces* sp. QL37 and its *lugO* mutants, grown on either MM (**A**) or R5 (**B**) agar. The nodes are labelled by the precursor mass of their ions and pie charts are mapped to the nodes representing the relative intensities of the ions in the different samples

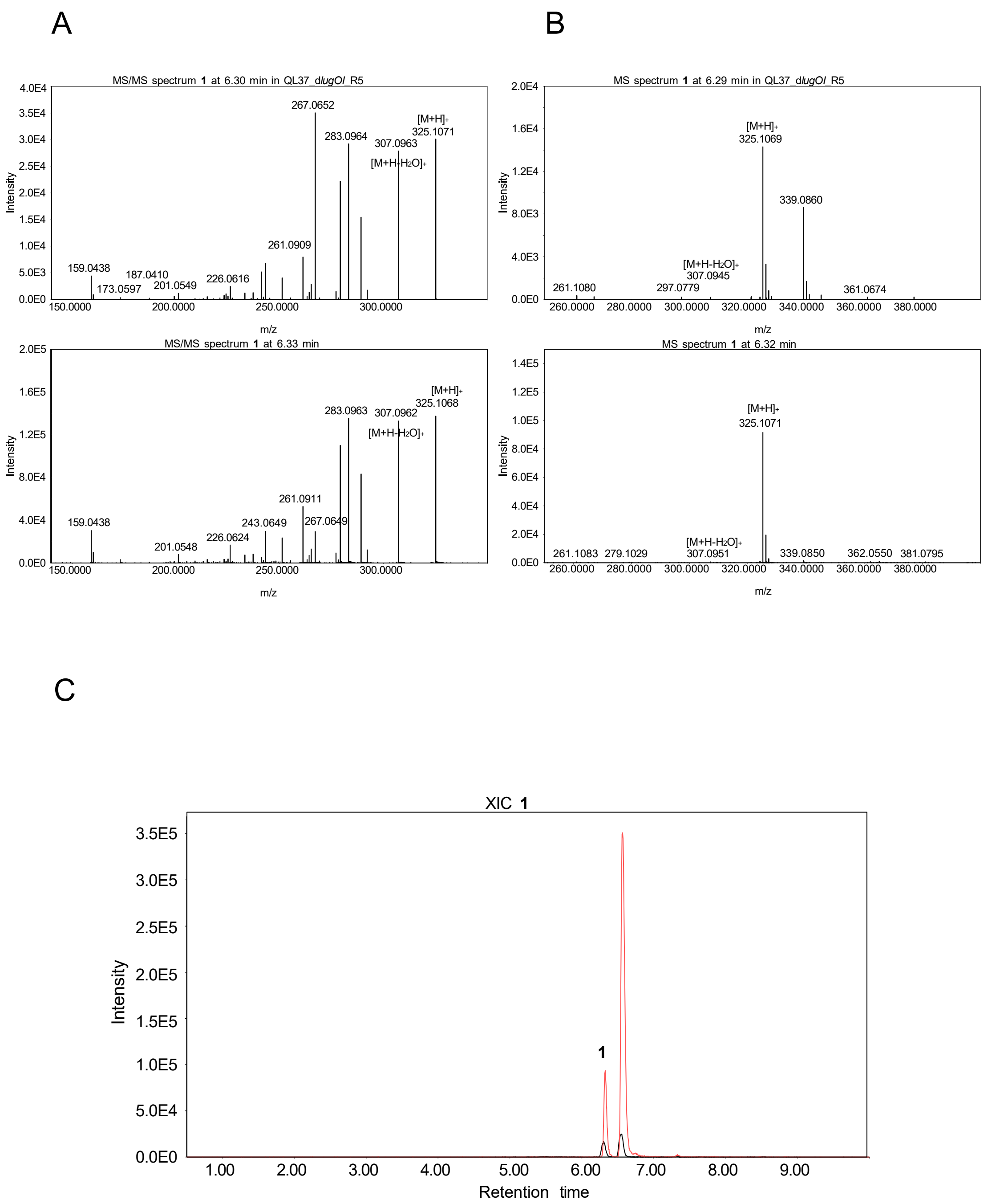

Fig. S3. Identification of **1** in the crude extract of *Streptomyces* sp. QL37. Comparison of the MS/MS (A) and MS spectra (B) of the semi-pure compound **1** (bottom) and its corresponding peak in the bacterial crude extract (top). The extracted ion chromatograms of the two peaks are shown in (C), where the red trace is the previously purified compound

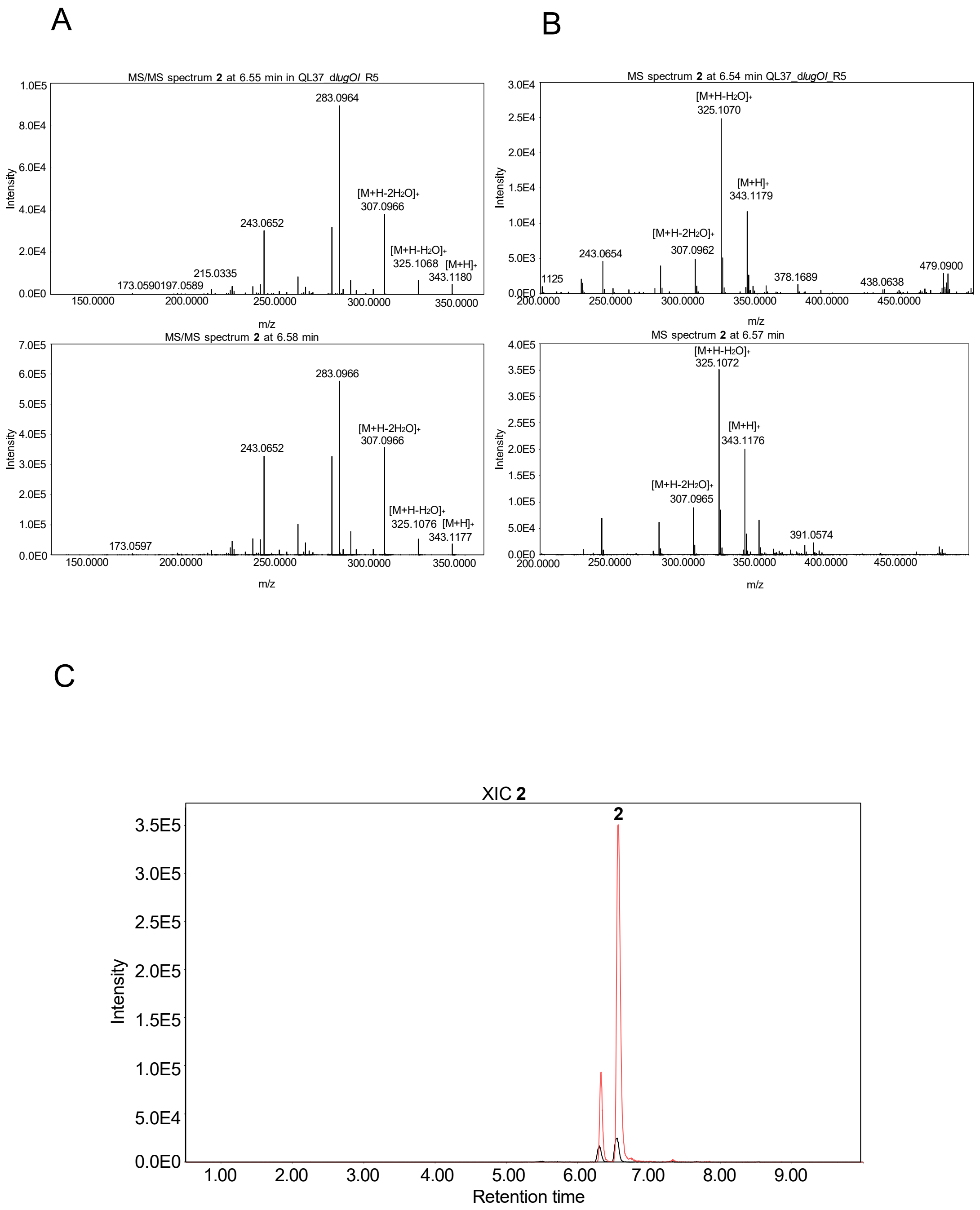

Fig. S4. Identification of **2** in the crude extract of *Streptomyces* sp. QL37. Comparison of the MS/MS (A) and MS spectra (B) of the semi-pure compound **2** (bottom) and its corresponding peak in the bacterial crude extract (top). The extracted ion chromatograms of the two peaks are shown in (C), where the red trace is the previously purified compound

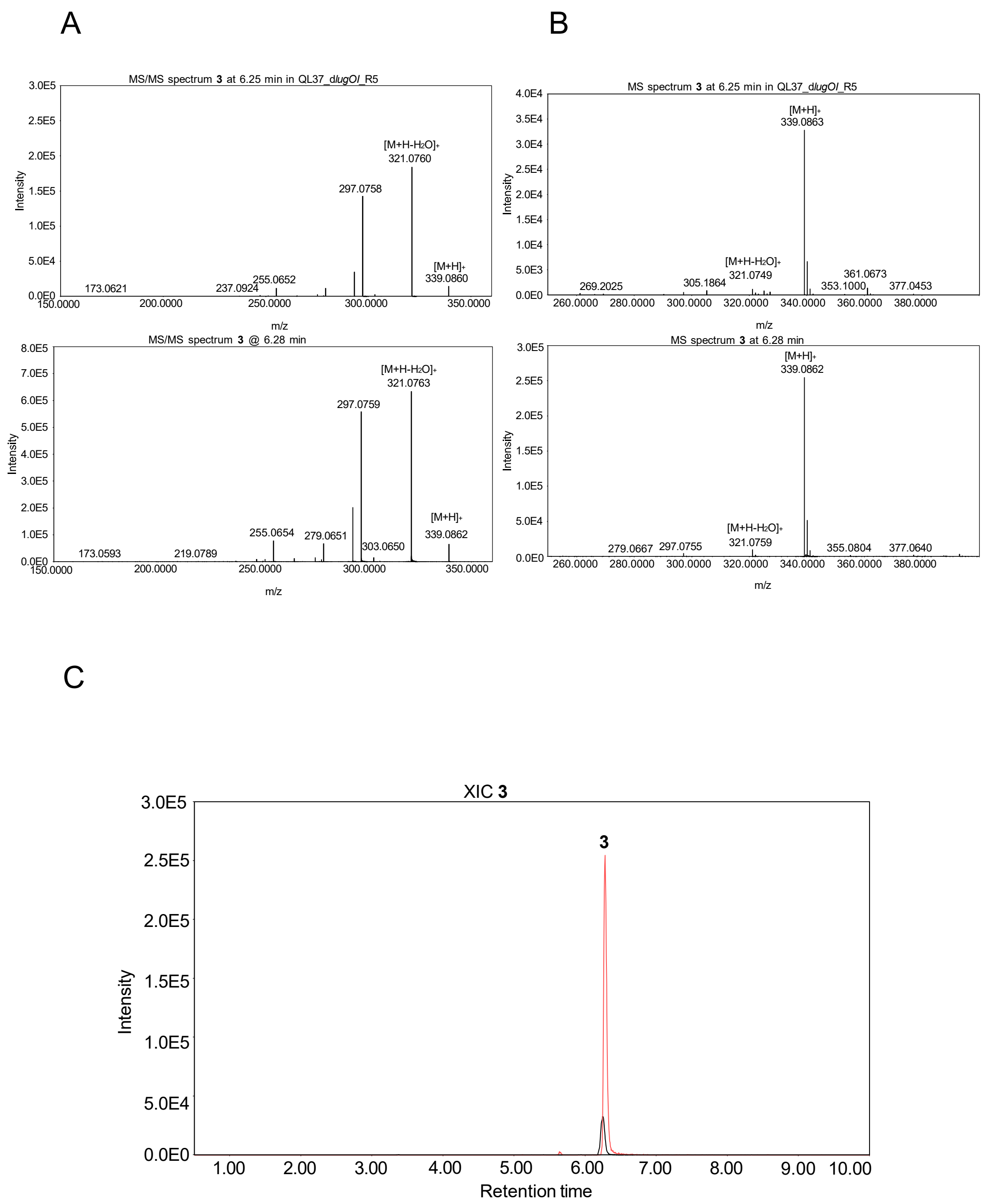

Fig. S5. Identification of **3** in the crude extract of *Streptomyces* sp. QL37. Comparison of the MS/MS (A) and MS spectra (B) of the semi-pure compound **3** (bottom) and its corresponding peak in the bacterial crude extract (top). The extracted ion chromatograms of the two peaks are shown in (C), where the red trace is the previously purified compound

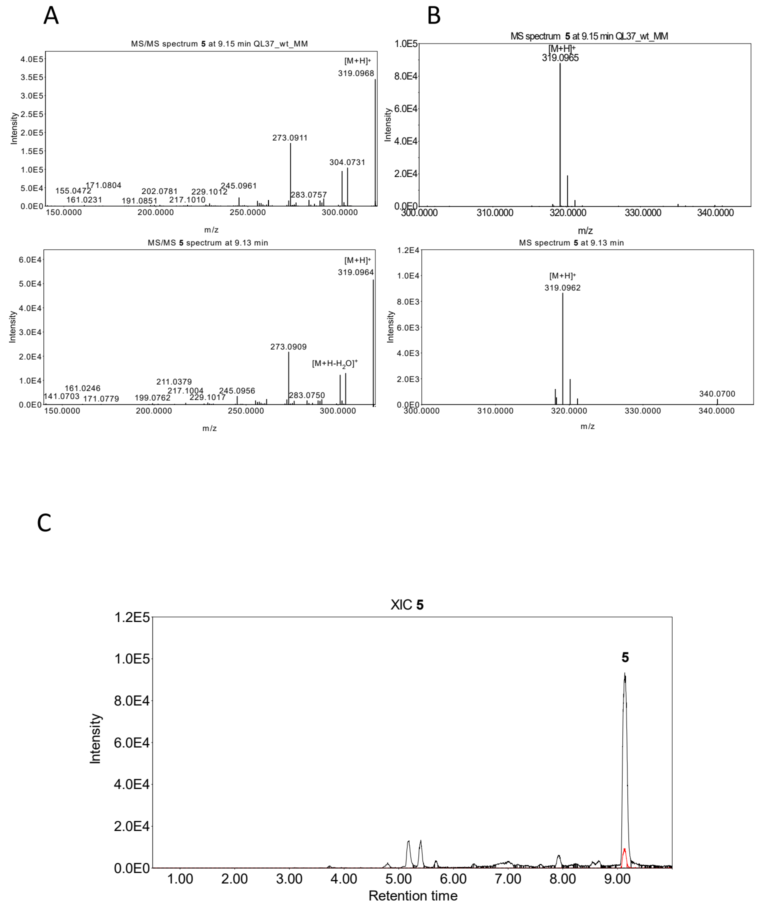

Fig. S6. Identification of **5** in the crude extract of *Streptomyces* sp. QL37. Comparison of the MS/MS (A) and MS spectra (B) of the semi-pure compound **5** (bottom) and its corresponding peak in the bacterial crude extract (top). The extracted ion chromatograms of the two peaks are shown in (C), where the red trace is the previously purified compound

A

B

C

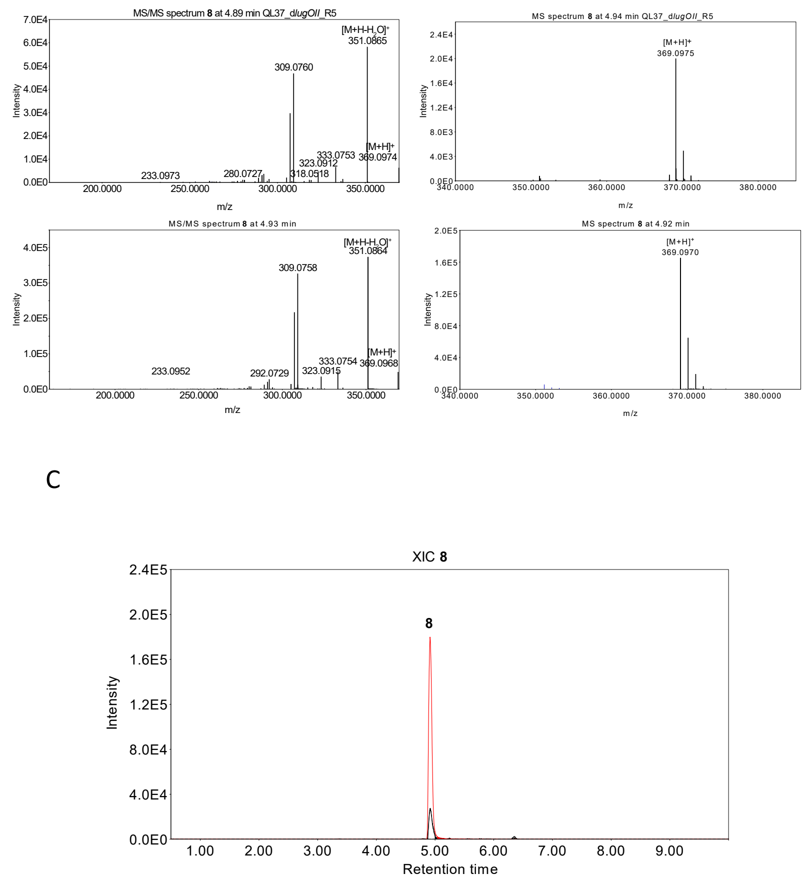

Fig. S7. Identification of **8** in the crude extract of *Streptomyces* sp. QL37. Comparison of the MS/MS (A) and MS spectra (B) of the semi-pure compound **8** (bottom) and its corresponding peak in the bacterial crude extract (top). The extracted ion chromatograms of the two peaks are shown in (C), where the red trace is the previously purified compound

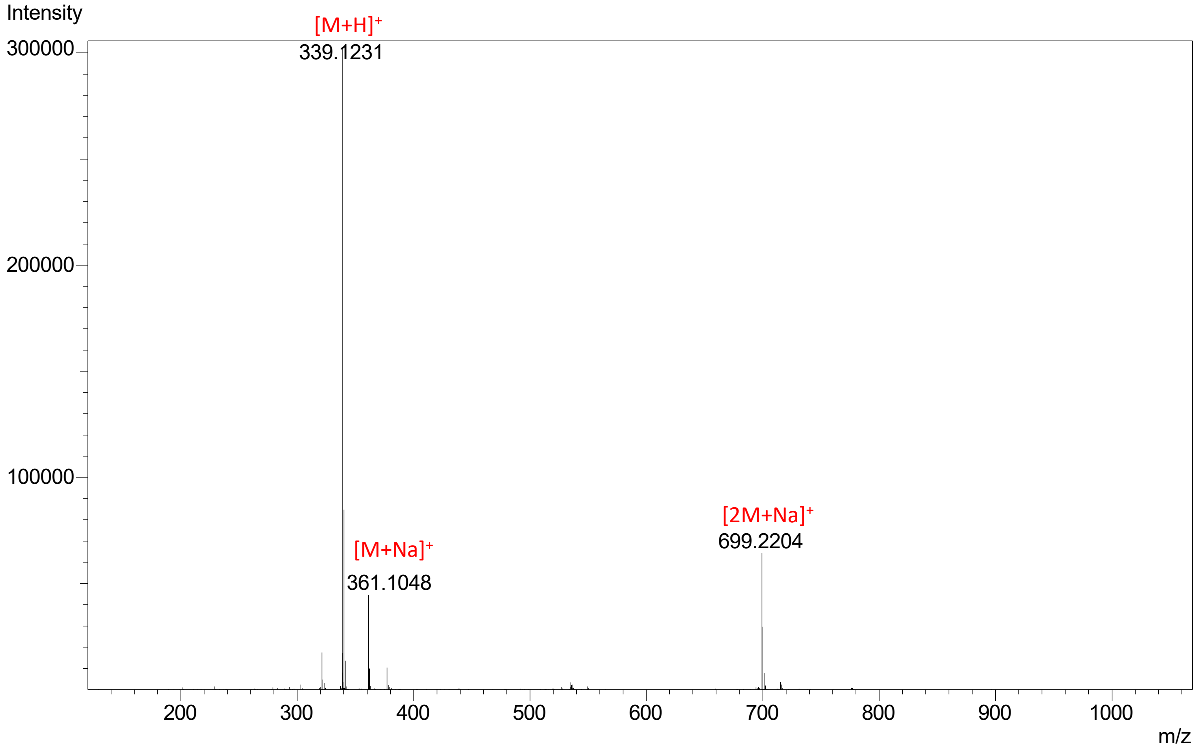

Fig. S8. HRESIMS spectrum of the peak annotated as compound **9** in the crude extract of *Streptomyces* sp. QL37

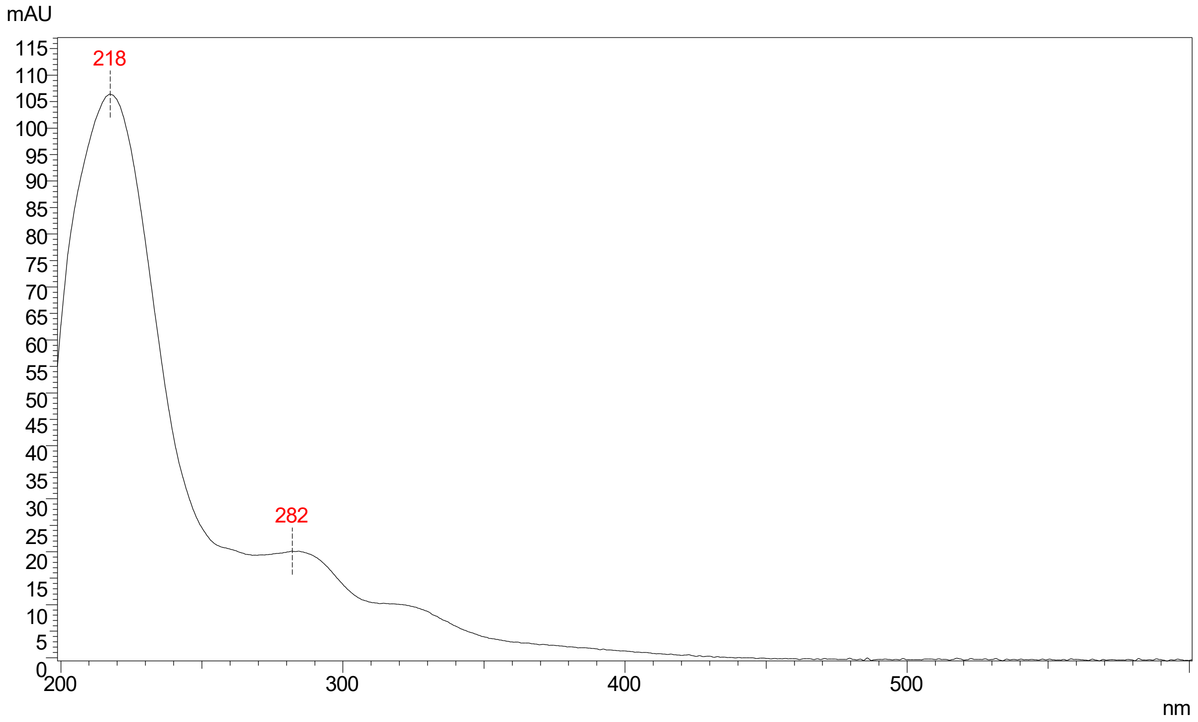

Fig. S9. UV spectrum of the peak annotated as compound **9** in the crude extract of *Streptomyces* sp. QL37

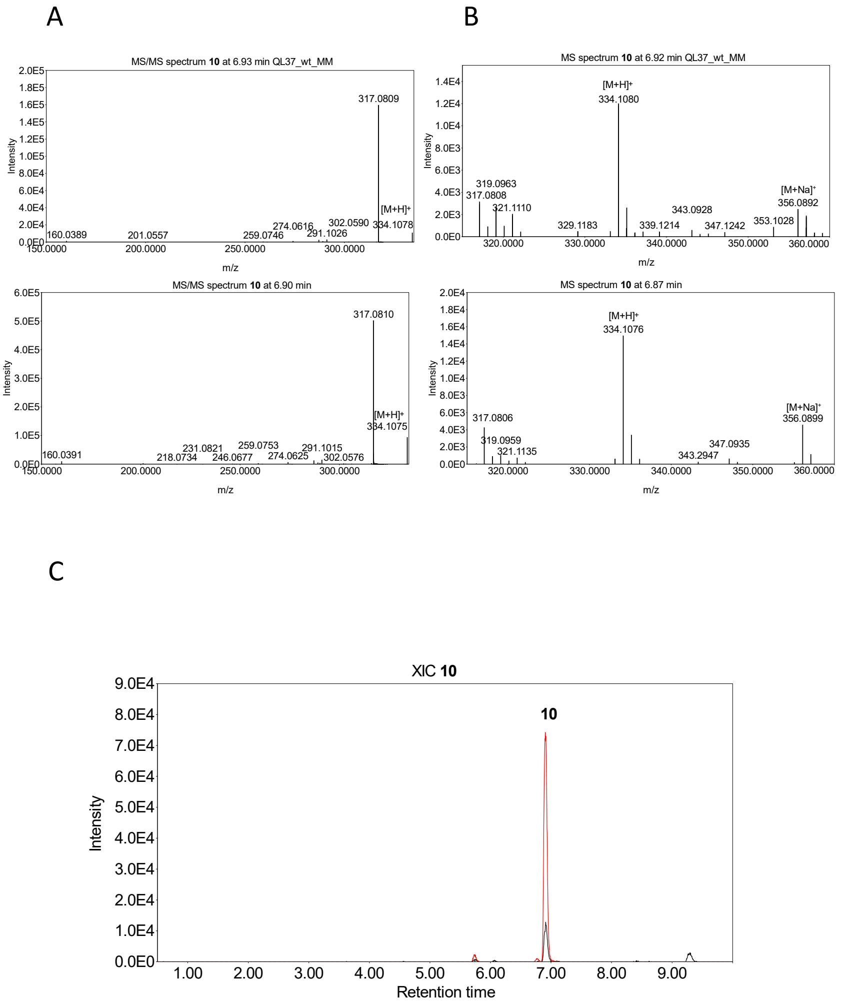

Fig. S10. Identification of **10** in the crude extract of *Streptomyces* sp. QL37. Comparison of the MS/MS (A) and MS spectra (B) of the semi-pure compound **10** (bottom) and its corresponding peak in the bacterial crude extract (top). The extracted ion chromatograms of the two peaks are shown in (C), where the red trace is the previously purified compound

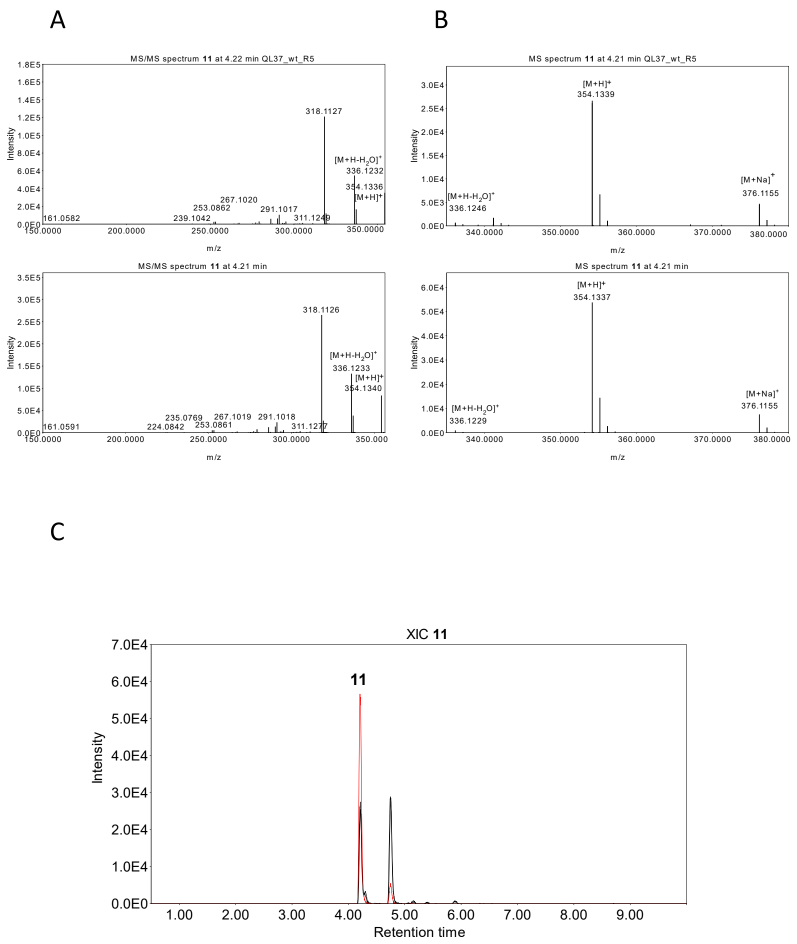

Fig. S11. Identification of **11** in the crude extract of *Streptomyces* sp. QL37. Comparison of the MS/MS (A) and MS spectra (B) of the semi-pure compound **11** (bottom) and its corresponding peak in the bacterial crude extract (top). The extracted ion chromatograms of the two peaks are shown in (C), where the red trace is the previously purified compound

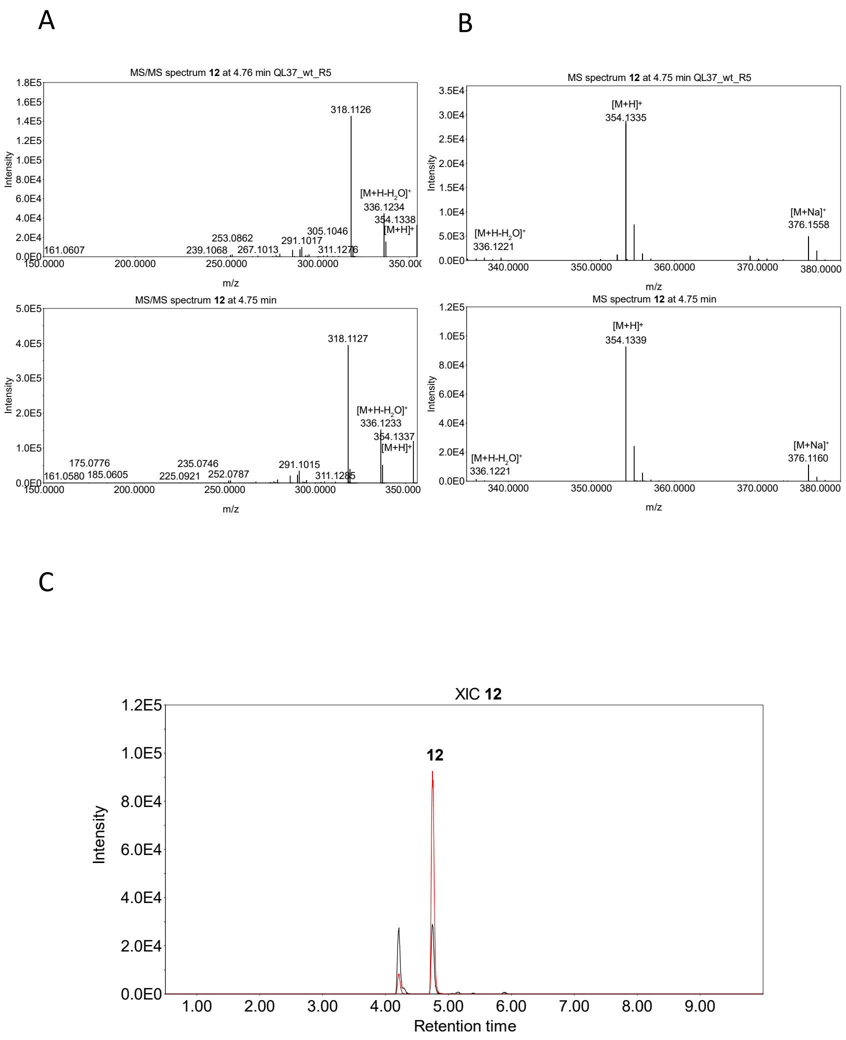

Fig. S12. Identification of **12** in the crude extract of *Streptomyces* sp. QL37. Comparison of the MS/MS (A) and MS spectra (B) of the semi-pure compound **12** (bottom) and its corresponding peak in the bacterial crude extract (top). The extracted ion chromatograms of the two peaks are shown in (C), where the red trace is the previously purified compound

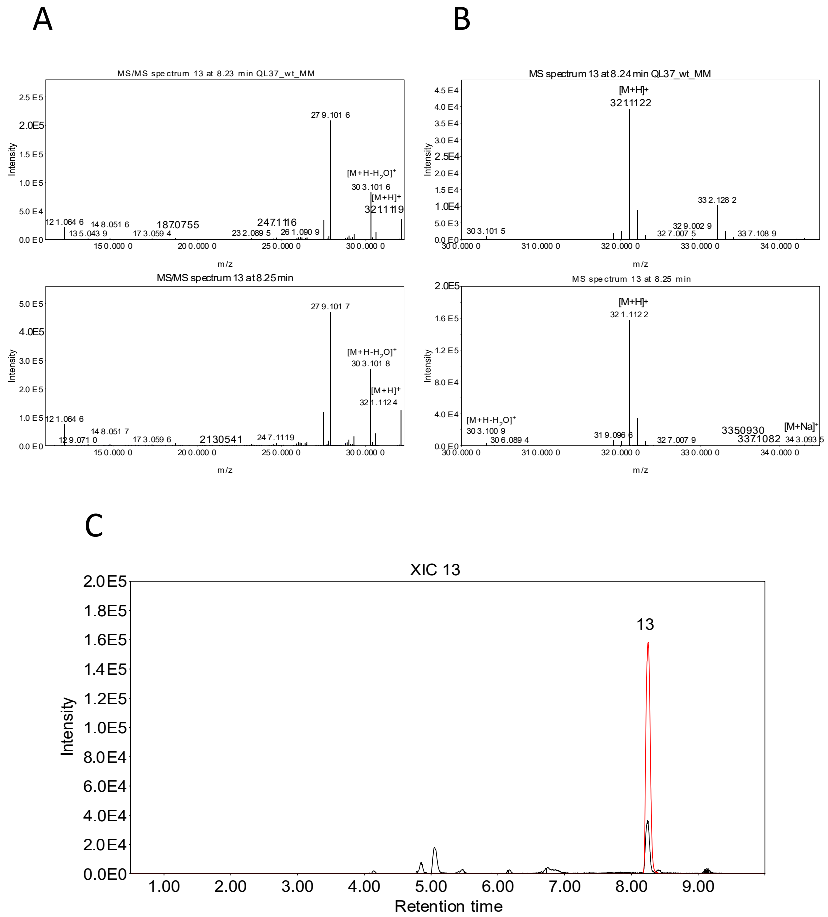

Fig. S13. Identification of **13** in the crude extract of *Streptomyces* sp. QL37. Comparison of the MS/MS (A) and MS spectra (B) of the semi-pure compound **13** (bottom) and its corresponding peak in the bacterial crude extract (top). The extracted ion chromatograms of the two peaks are shown in (C), where the red trace is the previously purified compound

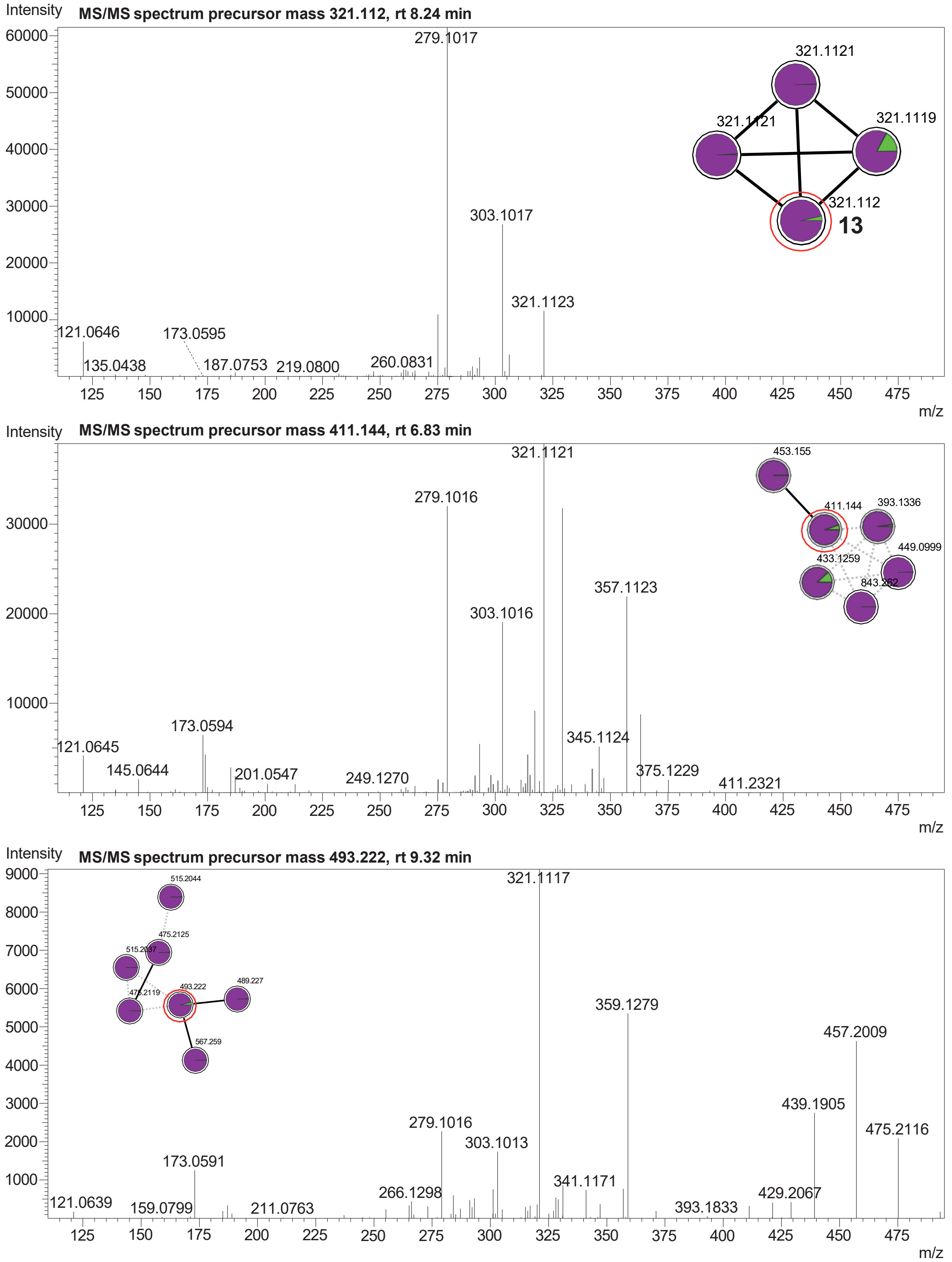

Fig. S14. Molecular families with similar MS/MS fragmentation as elmonin. MS/MS spectrum of the elmonin (**13**) (top) as compared to the MS/MS spectra of other mass features in different molecular families, which showed a fragment ion with the same mass as **13** that further fragments similarly. The nodes whose MS/MS spectra are given are highlighted

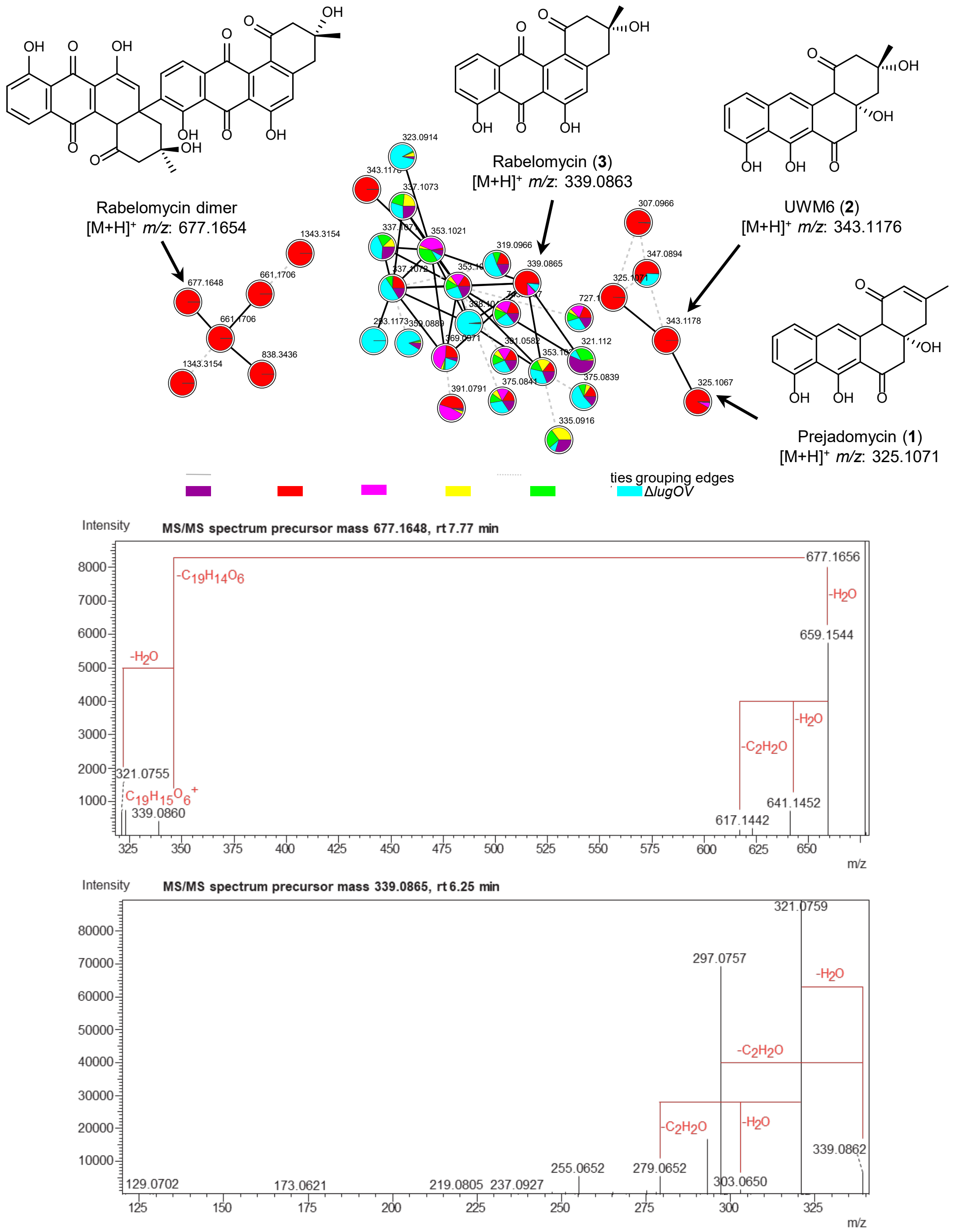

Fig. S15. (A) Nodes in the molecular network generated using the extracts of the R5-grown cultures showing **1**, **2**, **3** and metabolites which could be annotated as angucycline dimers related to **3**. (B) MS/MS spectrum of a likely dimer of two rabelomycin molecules (top), as compared to that of **3** (bottom). The dimer shows in the MS/MS spectrum a loss of a rabelomycin molecule ($C_{19}H_{14}O_6$) resulting in the fragment ion of the second rabelomycin ($C_{19}H_{15}O_6^+$). Additionally, a similar fragmentation pattern could be observed for both the dimer and rabelomycin

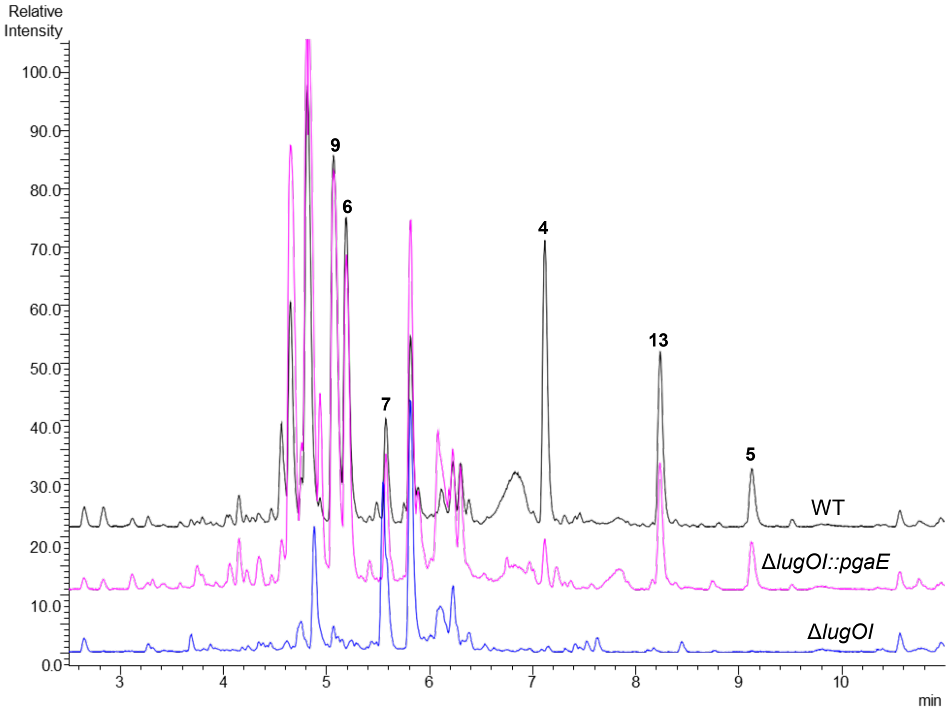

Fig. S16. LC-MS chromatogram overlay showing the extracts of *Streptomyces* sp. QL37 wild-type strain (WT), the *lugOI* deletion mutant (Δ*lugOI*), and the *lugOI* mutant complemented with *pgaE* (Δ*lugOI::pgaE*). The complementation of Δ*lugOI* with *pgaE* restored the wild-type metabolomic profile. The compound numbers are given above their respective peaks

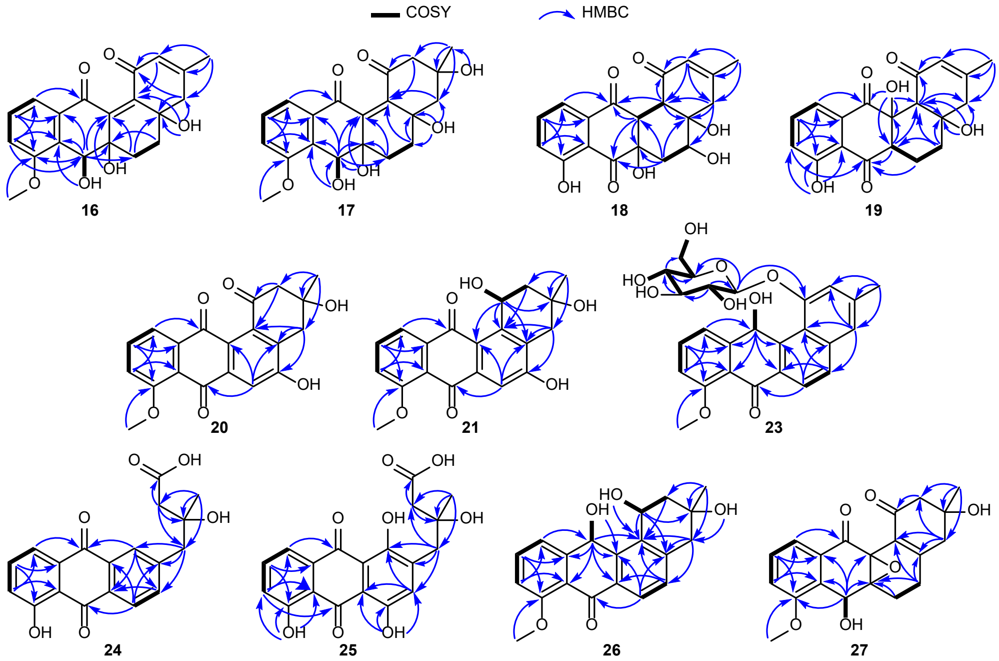

Fig. S17. Key COSY and HMBC correlations observed for the new angucyclines isolated from the Δ*lugOV* and Δ*lugOV::lugOV* strains grown on R5

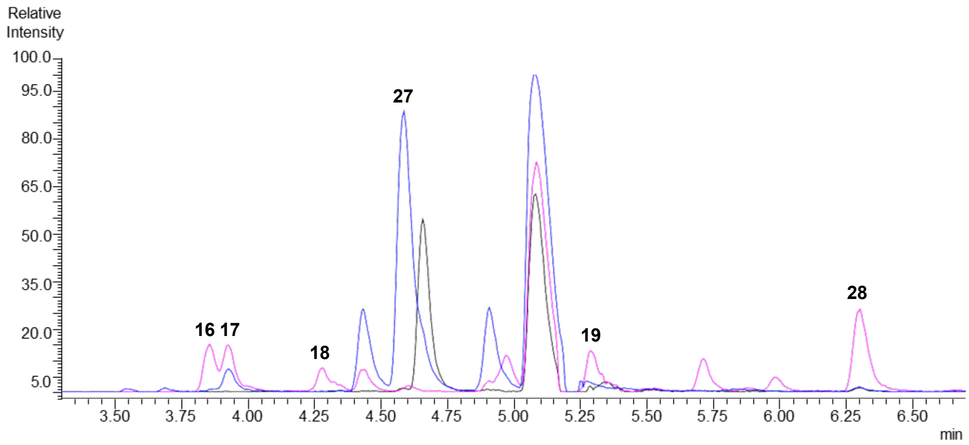

Fig. S18. Extracted ion chromatogram of selected peaks in the extracts of the R5-grown cultures of *Streptomyces* sp. QL37 wild-type (black), its *lugOV* mutant (pink), and the *lugOV* mutant complemented with *lugOV* (blue). The deletion of *lugOV* resulted in the upregulation of non-rearranged angucyclinones having a ring-opened epoxide moiety like **16**–**19**, as well as having an 8-hydroxy substitution like **18**, **19**, and **28**. The complementation of the *lugOV* mutant with *lugOV* suppressed the production of these metabolites and resulted in a considerable upregulation of **27** which has an intact epoxide moiety

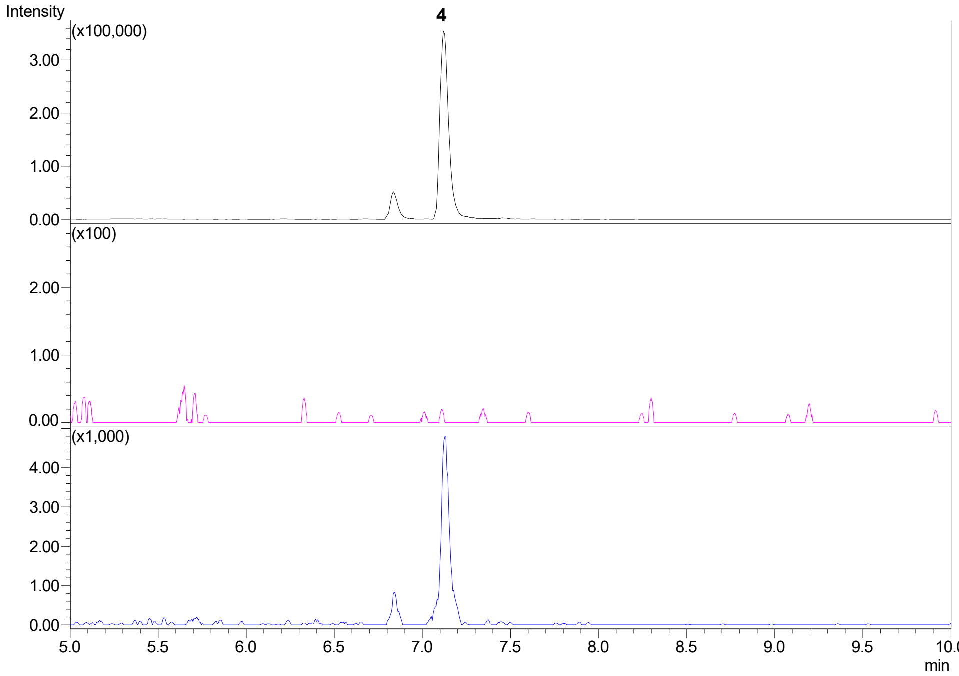

Fig. S19. Extracted ion chromatogram (XIC) of the mass peak of compound **4** in the extracts of the MM-grown cultures of *Streptomyces* sp. QL37 wild-type (WT), its *lugOV* mutant (Δ*lugOV*), and the *lugOV* mutant complemented with *lugOV* (Δ*lugOV*::*lugOV*). The complementation restored the production of **4**, although at lower levels than the wild-type

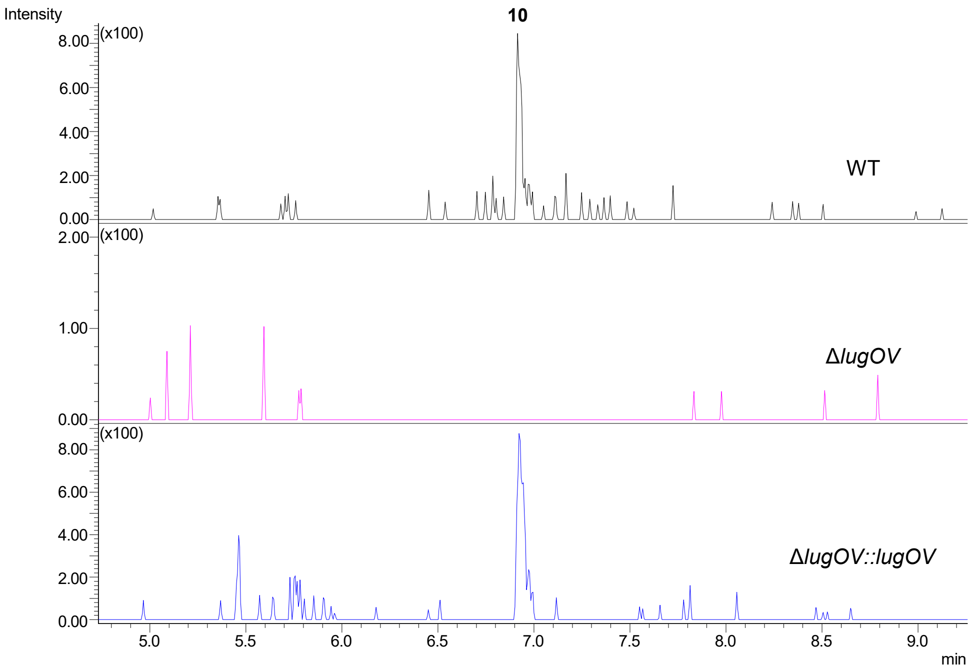

Fig. S20. Extracted ion chromatogram (XIC) of the mass peak of compound **10** in the extracts of the MM-grown cultures of *Streptomyces* sp. QL37 wild-type (WT), its *lugOV* mutant (Δ*lugOV*), and the *lugOV* mutant complemented with *lugOV* (Δ*lugOV*::*lugOV*). The complementation restored the production of **10**, although at lower levels than the wild-type

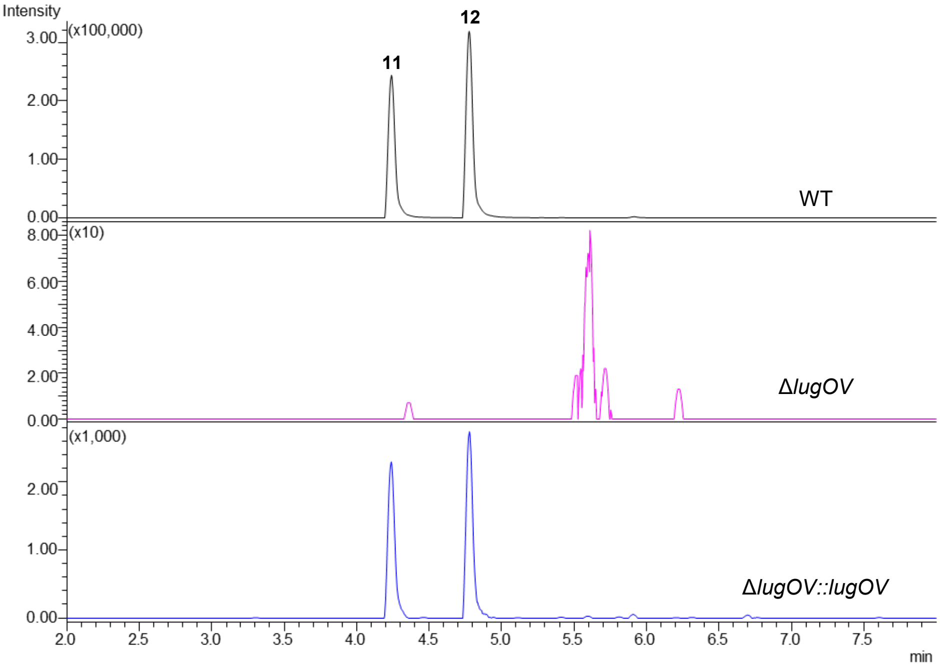

Fig. S21. Multiple reaction monitoring (MRM) chromatogram for compounds **11** and **12** in the extracts of the R5-grown cultures of *Streptomyces* sp. QL37 wild-type (WT), its *lugOV* mutant (Δ*lugOV*), and the *lugOV* mutant complemented with *lugOV* (Δ*lugOV*::*lugOV*). The complementation restored the production of **11** and **12**, although at lower levels than the wild-type

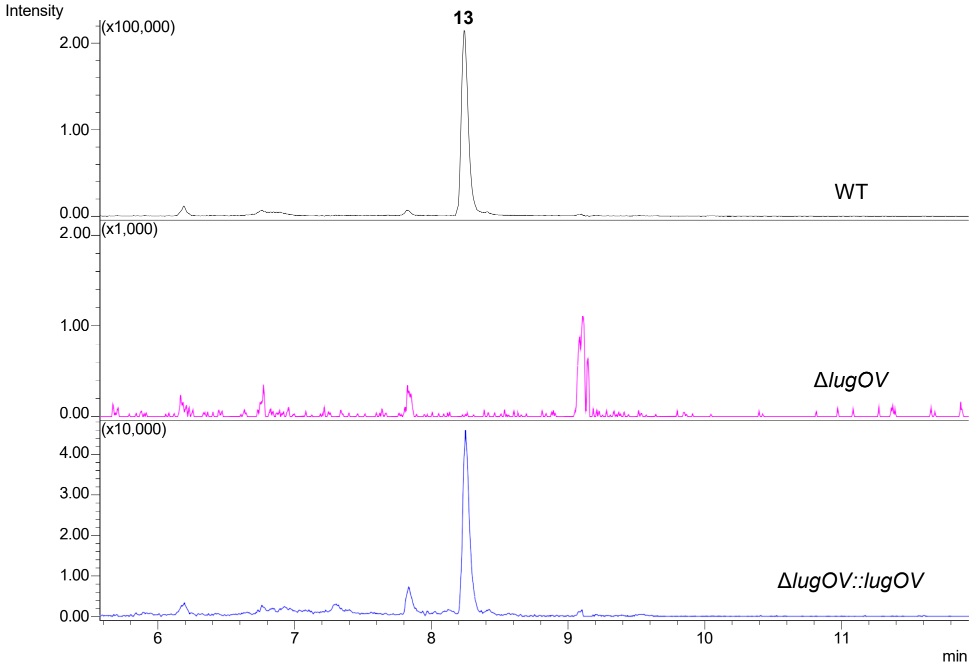

Fig. S22. Extracted ion chromatogram (XIC) of the mass peak of compound **13** in the extracts of the MM-grown cultures of *Streptomyces* sp. QL37 wild-type (WT), its *lugOV* mutant (Δ*lugOV*), and the *lugOV* mutant complemented with *lugOV* (Δ*lugOV::lugOV*). The complementation restored the production of **13**, although at lower levels than the wild-type

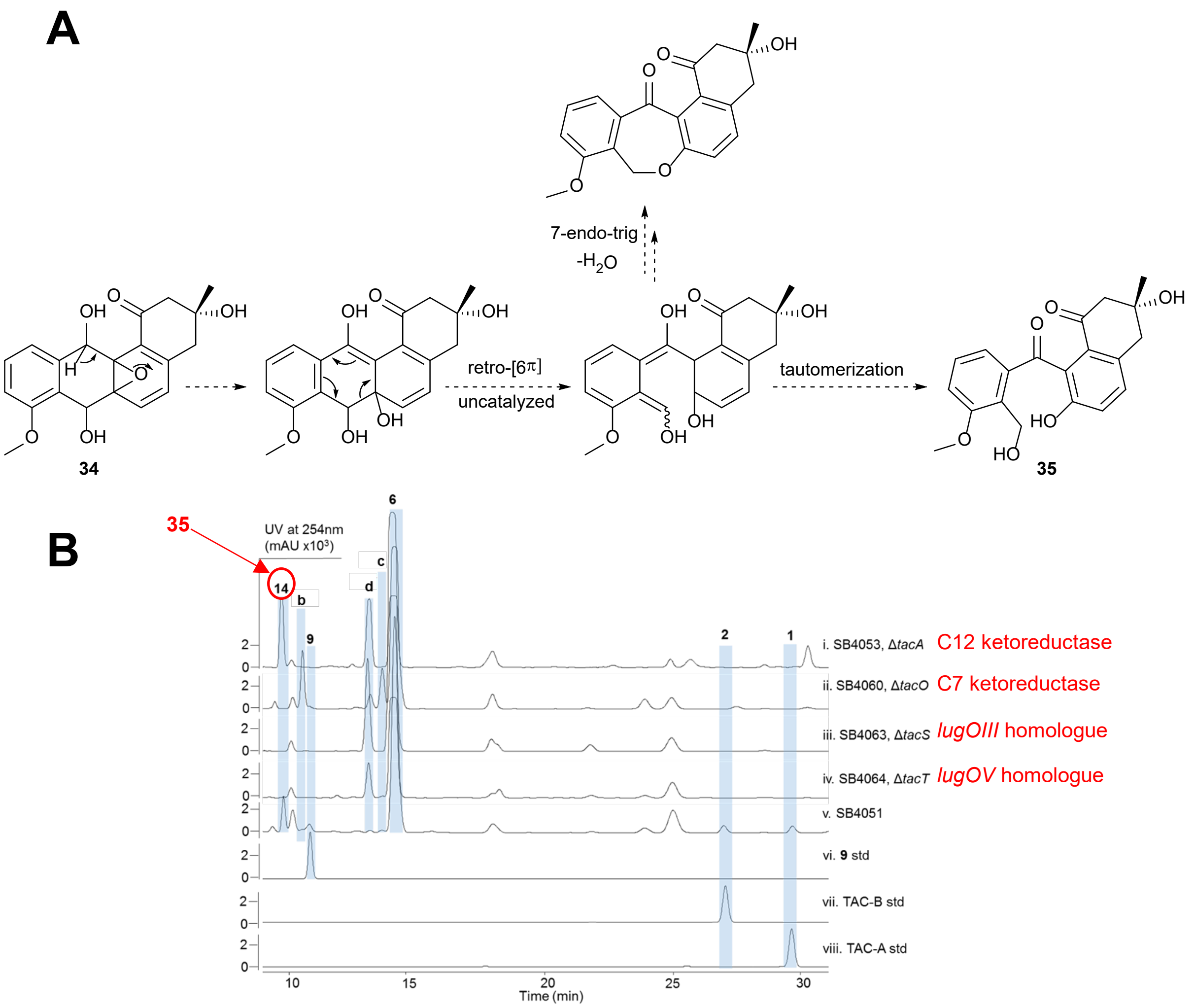

Fig. S23. (A) Proposed biosynthetic pathway for intermediate **35** as per a recently published review [6]. (B) Fig. adapted from a recent research showing the production of intermediate **35** upon the deletion of different post-PKS genes in the *tac* BGC, which is homologous to *lug* [7]

Supplementary References

1 Sambrook, J. F., E.F.; Maniatis, T. *Molecular Cloning: A Laboratory Manual*. (Cold Spring Harbor Laboratory, 1989).

2 MacNeil, D. J. *et al.* Analysis of Streptomyces avermitilis genes required for avermectin biosynthesis utilizing a novel integration vector. *Gene* **111**, 61-68 (1992). https://doi.org:10.1016/0378-1119(92)90603-m

3 Wu, C. S. *et al.* Lugdunomycin, an Angucycline-Derived Molecule with Unprecedented Chemical Architecture. *Angew. Chem. Int. Ed.* **58**, 2809-2814 (2019). https://doi.org:10.1002/anie.201814581

4 Zhu, H. *et al.* Eliciting antibiotics active against the ESKAPE pathogens in a collection of actinomycetes isolated from mountain soils. *Microbiology-Sgm* **160**, 1714-1726 (2014). https://doi.org:10.1099/mic.0.078295-0

5 Xiao, X. S. *et al.* Functional and Structural Insights into a Novel Promiscuous Ketoreductase of the Lugdunomycin Biosynthetic Pathway. *ACS Chem. Biol.* **15**, 2529-2538 (2020). https://doi.org:10.1021/acschembio.0c00564

6 Mikhaylov, A. A., Ikonnikova, V. A. & Solyev, P. N. Disclosing biosynthetic connections and functions of atypical angucyclinones with a fragmented C-ring. *Nat. Prod. Rep.* **38**, 1506-1517 (2021). https://doi.org:10.1039/d0np00082e

7 Cao, M. M. *et al.* Cryptic Sulfur Incorporation in Thioangucycline Biosynthesis. *Angew. Chem. Int. Ed.* **60**, 7140-7147 (2021). https://doi.org:10.1002/anie.202015570
