# Supplementary material for: Unravelling key enzymatic steps in C-ring cleavage during angucycline biosynthesis
Source: Commun Chem. 2023 Dec 18;6:281. doi: 10.1038/s42004-023-01059-1 (PMC10728087; doi:10.1038/s42004-023-01059-1)
Supplement: Supplementary file 3 — Description of Additional Supplementary Files [file 42004_2023_1059_MOESM3_ESM.pdf]

Description of Additional Supplementary Files

File name: Supplementary Data 1

Description: HRMS, UV, and NMR spectra of the metabolites identified in this study
