# Supplementary material for: Unravelling key enzymatic steps in C-ring cleavage during angucycline biosynthesis
Source: Commun Chem. 2023 Dec 18;6:281. doi: 10.1038/s42004-023-01059-1 (PMC10728087; doi:10.1038/s42004-023-01059-1)
Supplement: Supplementary file 4 — Supplementary Data 1 [file 42004_2023_1059_MOESM4_ESM.pdf]

Supplementary Data 1

Unravelling key enzymatic steps in C-ring cleavage during angucycline biosynthesis

Somayah S. Elsayed[1,7,*], Helga U. van der Heul[1,7], Xiansha Xiao[2], Aleksi Nuutila[3], Laura R. Baars[4], Changsheng Wu[5], Mikko Metsä-Ketelä[3], Gilles P. van Wezel[1,6,*]

[1]Department of Molecular Biotechnology, Institute of Biology, Leiden University, Sylviusweg 72, 2333BE, Leiden, The Netherlands

[2]Department of Structural Biology, Van Andel Institute, Grand Rapids, Michigan, United States

[3]Department of Life Technologies, University of Turku, Tykistönkatu 6, FIN-20014, Turku, Finland

[4]Department of Systems Pharmacology and Pharmacy, Leiden Academic Centre for Drug Research, Leiden University, Einsteinweg 55, 2333CC, Leiden, The Netherlands

[5]State Key Laboratory of Microbial Technology, Institute of Microbial Technology, Shandong University, 266237, Qingdao, P.R. China

[6]Department of Microbial Ecology, Netherlands Institute of Ecology (NIOO-KNAW), Droevendaalsesteeg 10, 6708PB, Wageningen, The Netherlands

[7]These authors contributed equally

*Correspondence: s.elsayed@biology.leideuniv.nl (S.S.E), g.wezel@biology.leidenuniv.nl (G.P.vW.)

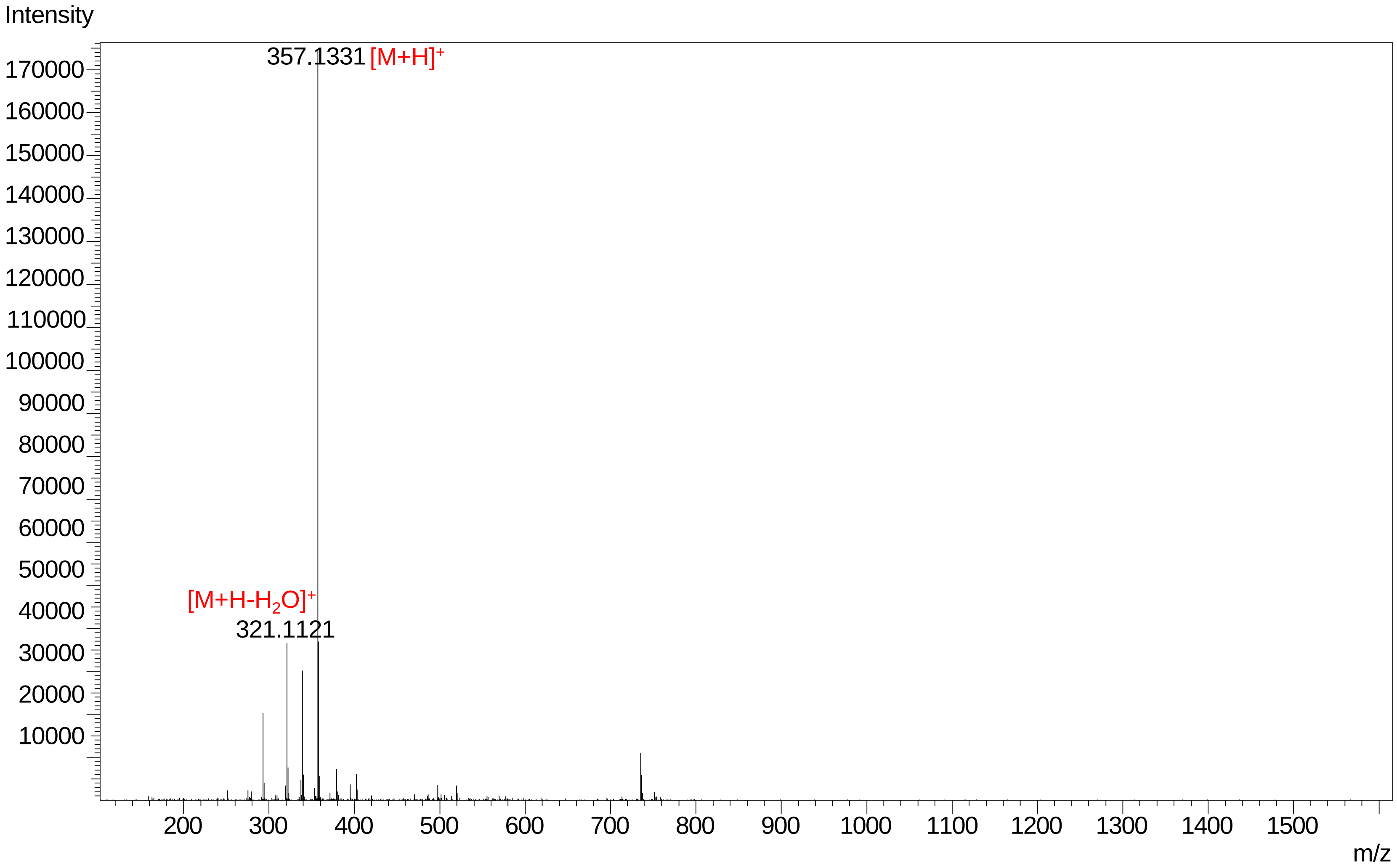

Fig. S24. (+)-HRESIMS spectrum of **16**

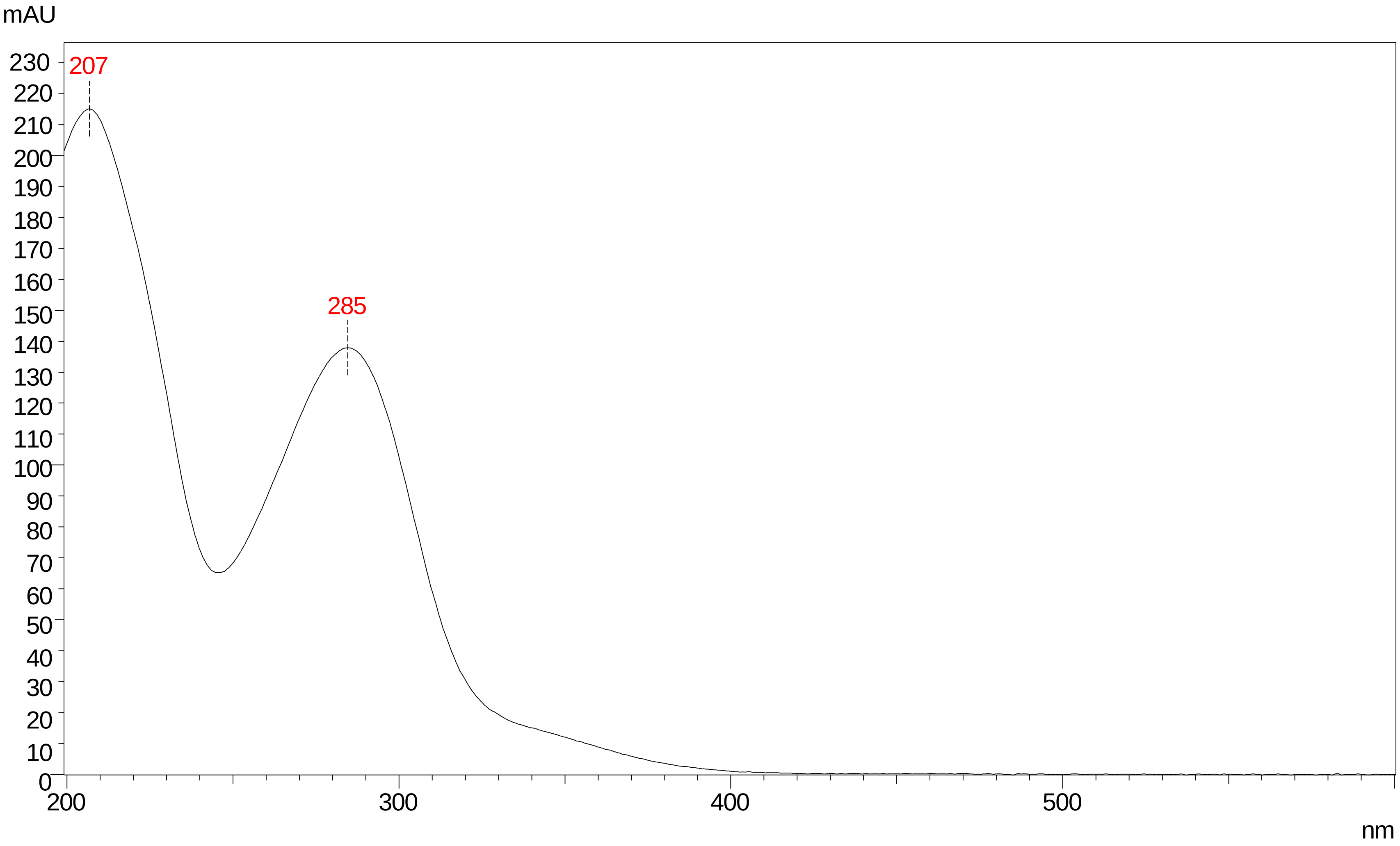

Fig. S25. UV spectrum of **16**

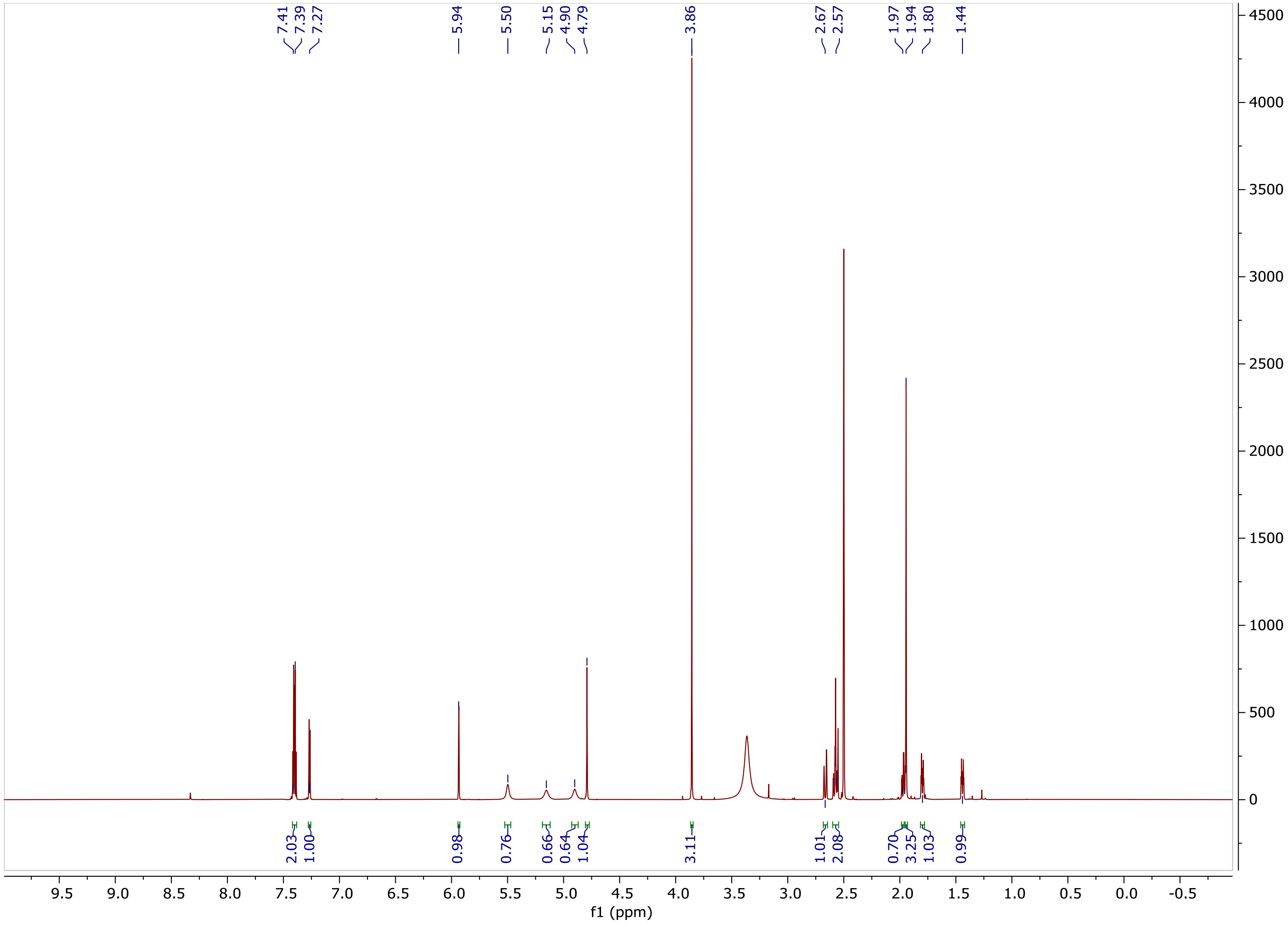

Fig. S26. ^{1}H NMR spectrum of **16** (850 MHz, in DMSO-*d6*)

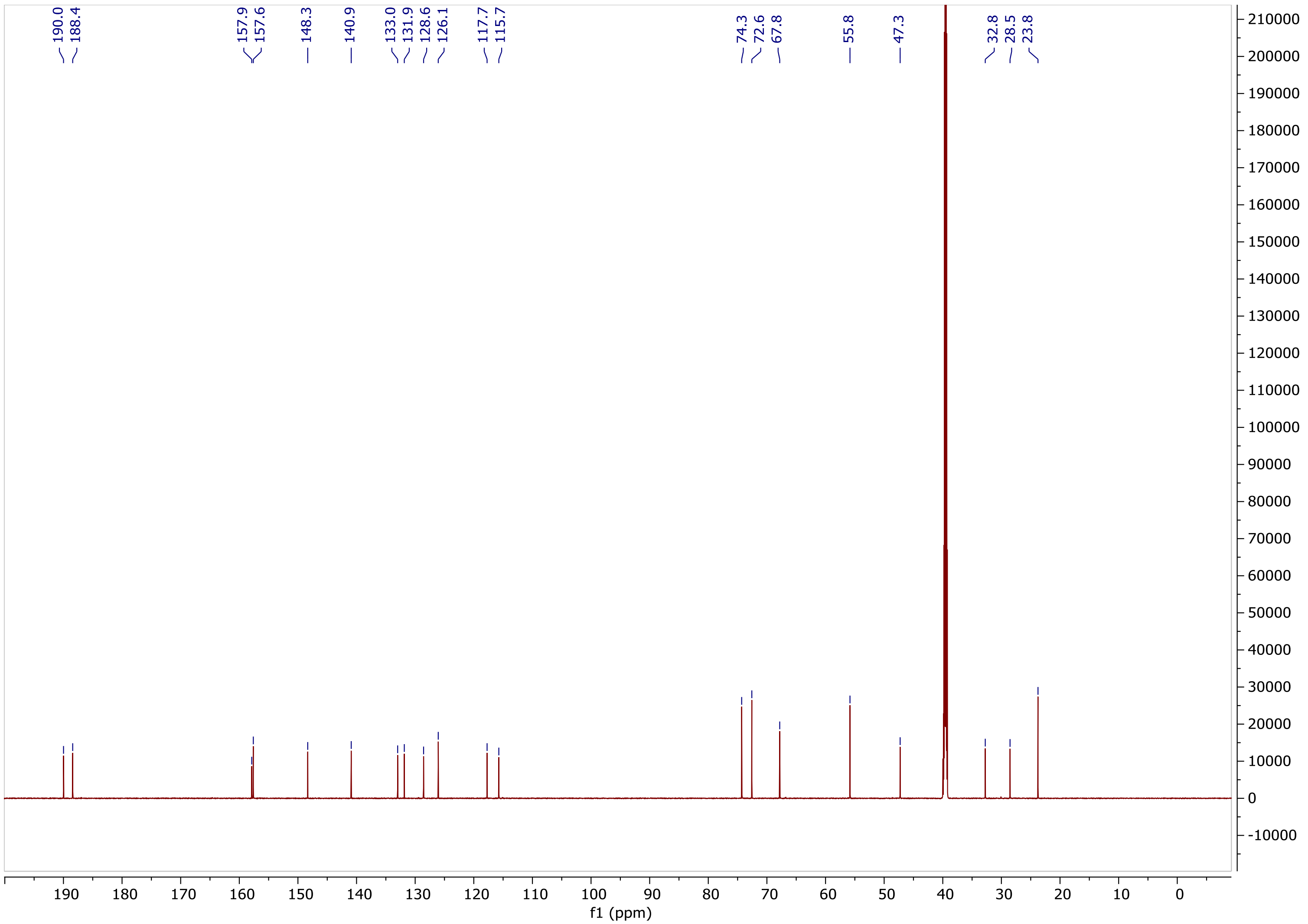

Fig. S27. ^{13}C NMR spectrum of **16** (213 MHz, in DMSO-*d6*)

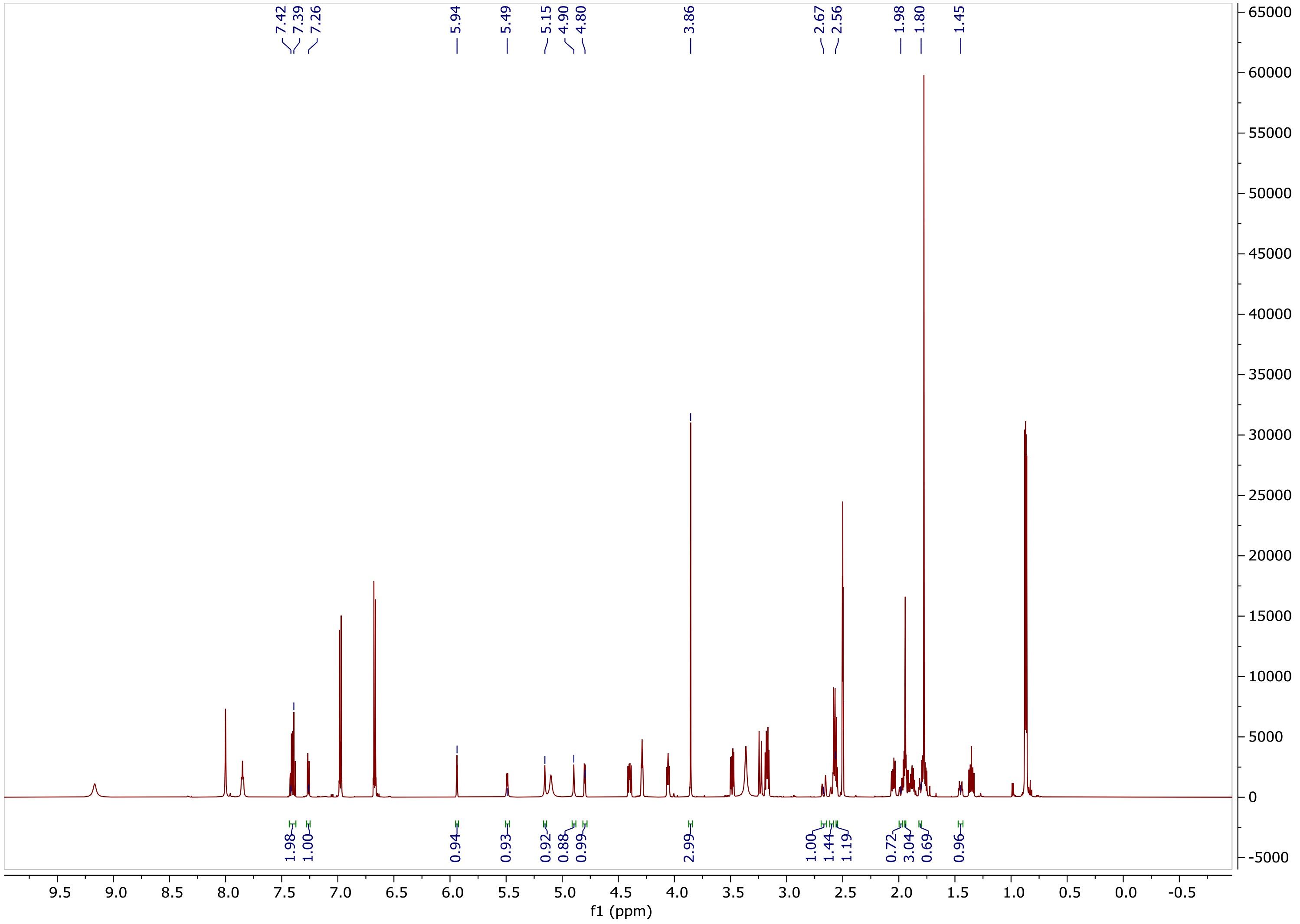

Fig. S28. ^{1}H NMR spectrum of **16** in mixture with **32** (600 MHz, in DMSO-*d6*)

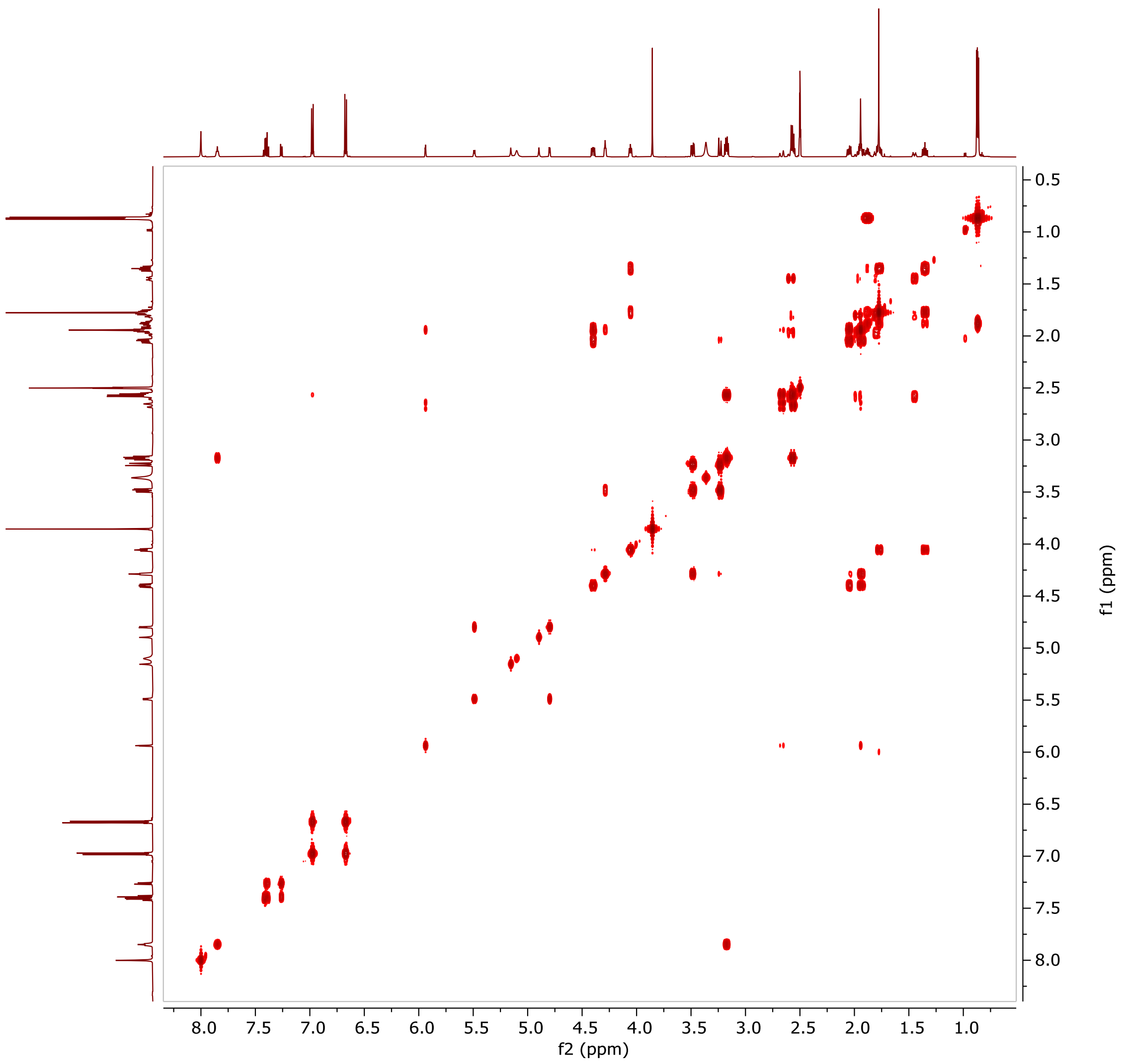

Fig. S29. COSY spectrum of **16** in mixture with **32** (600 MHz, in DMSO-*d6*)

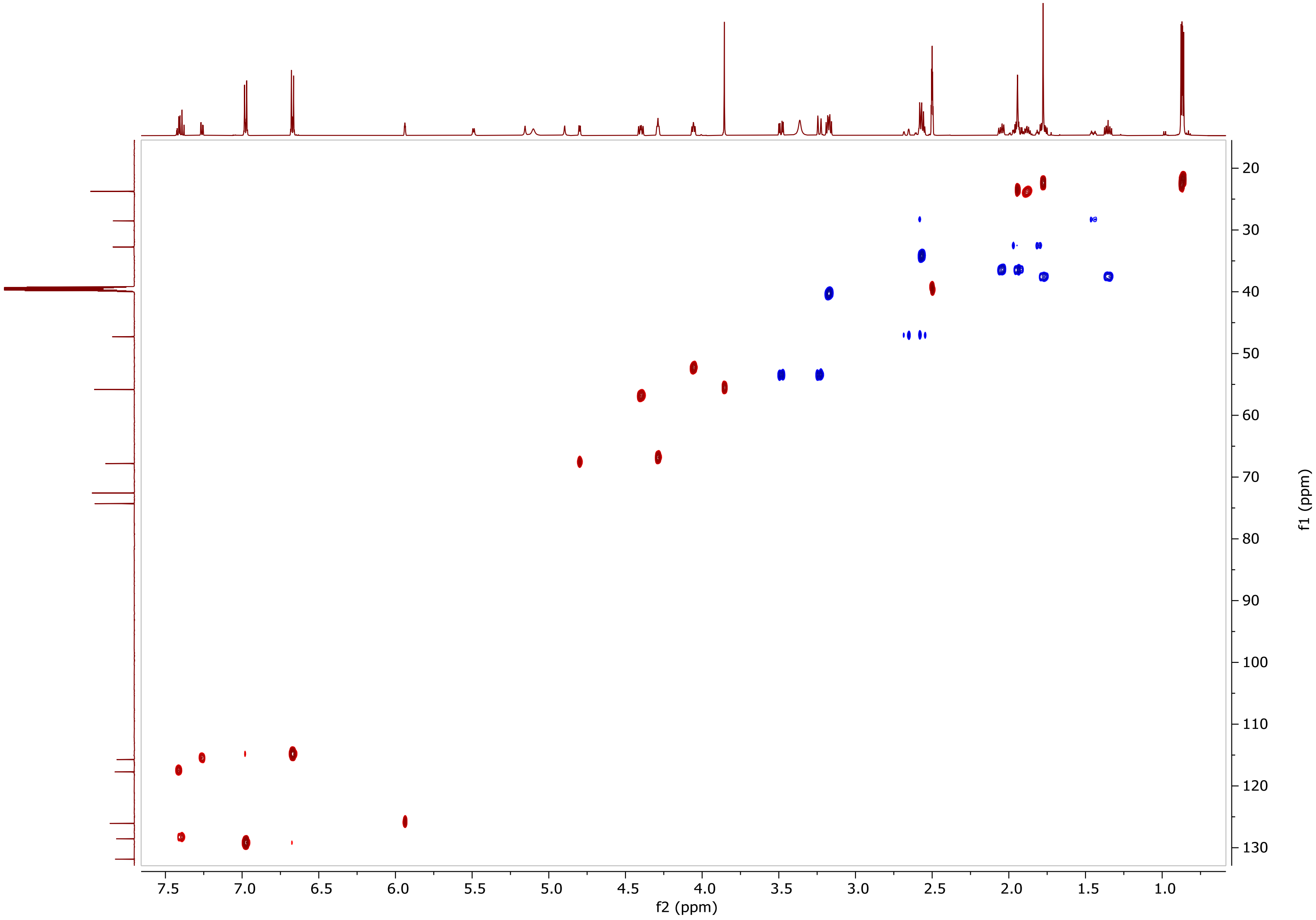

Fig. S30. Multiplicity-edited HSQC spectrum of **16** in mixture with **32** (600 MHz, in DMSO-*d6*)

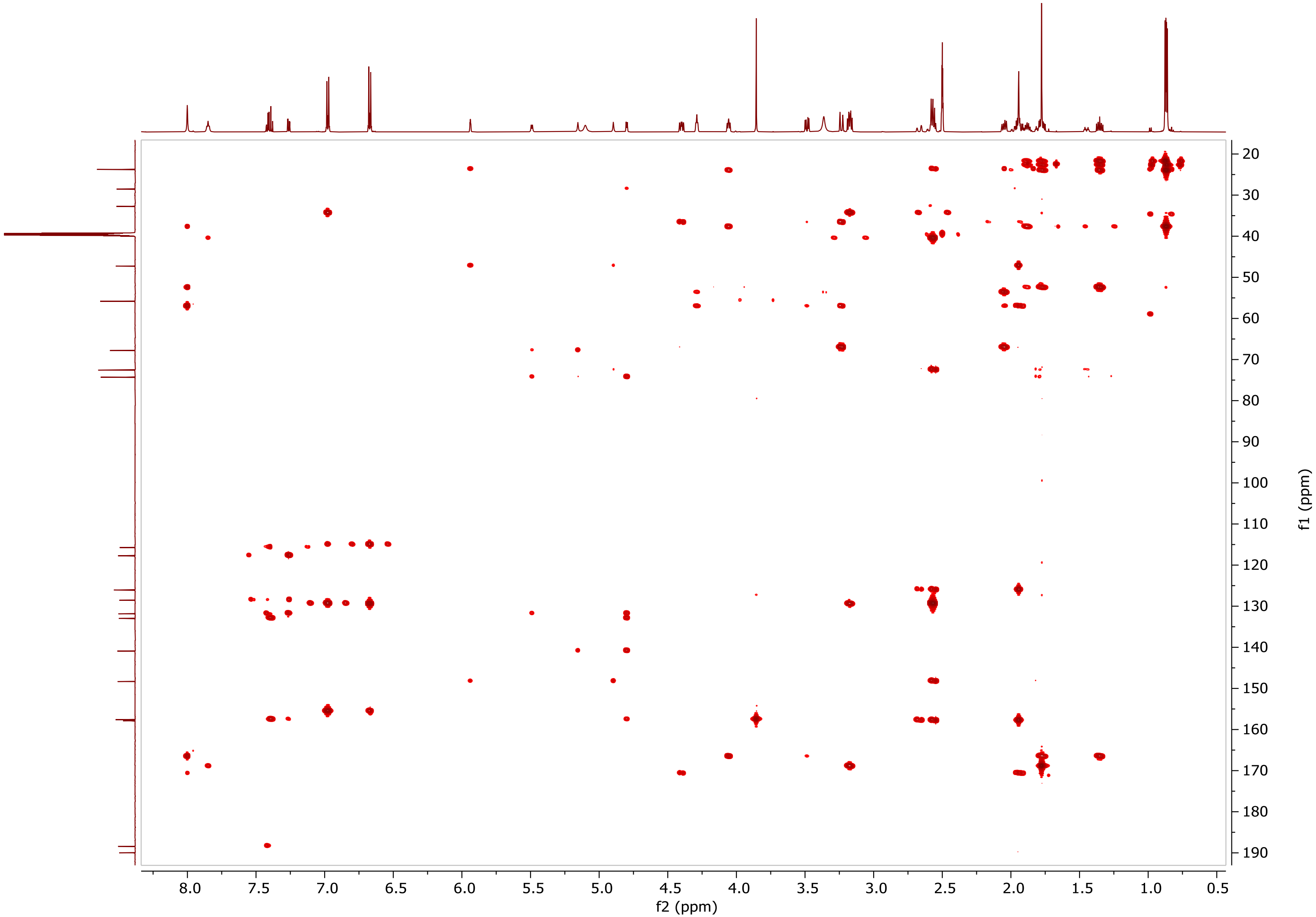

Fig. S31. HMBC spectrum of **16** in mixture with **32** (600 MHz, in DMSO-*d6*)

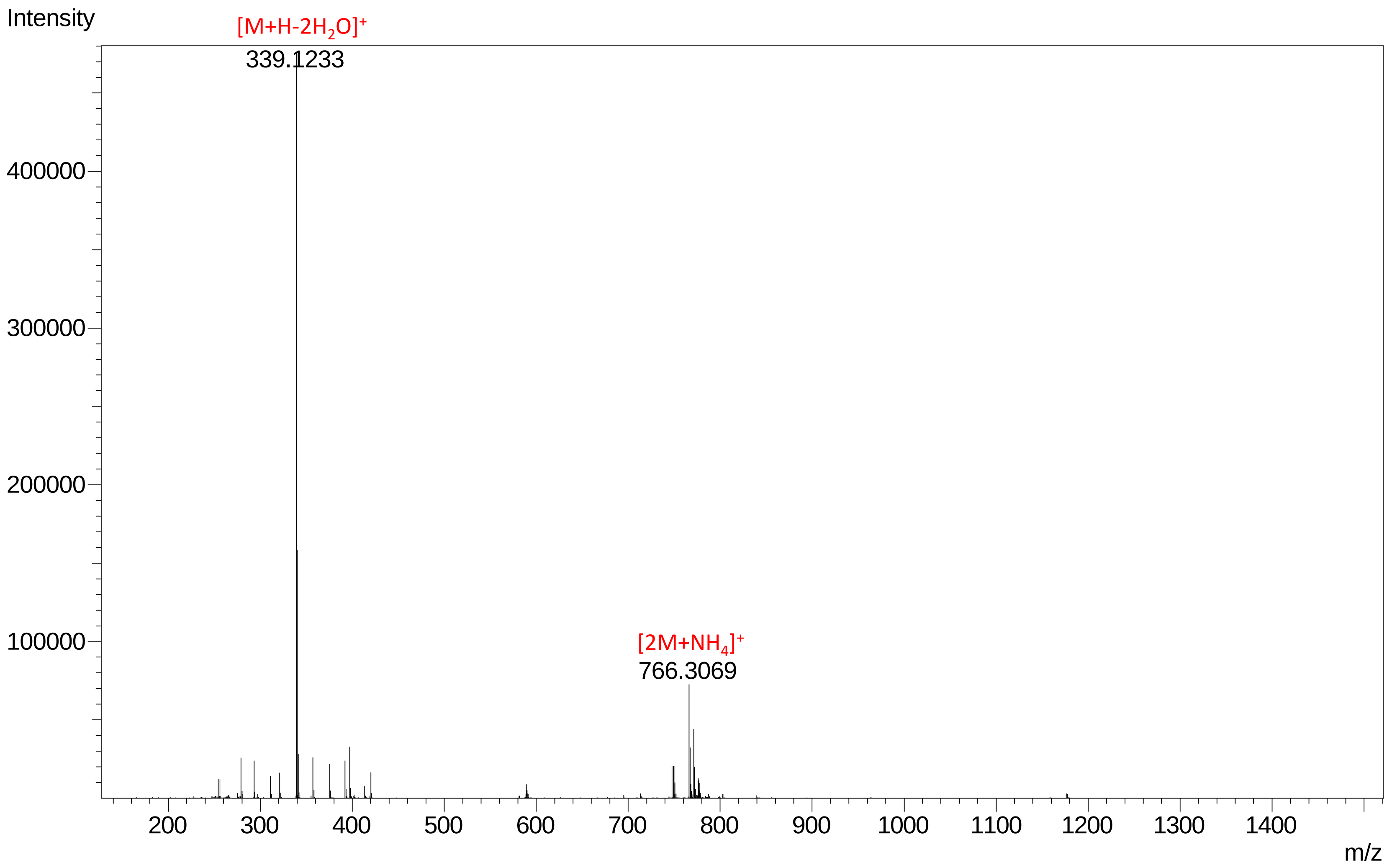

Fig. S32. (+)-HRESIMS spectrum of **17**

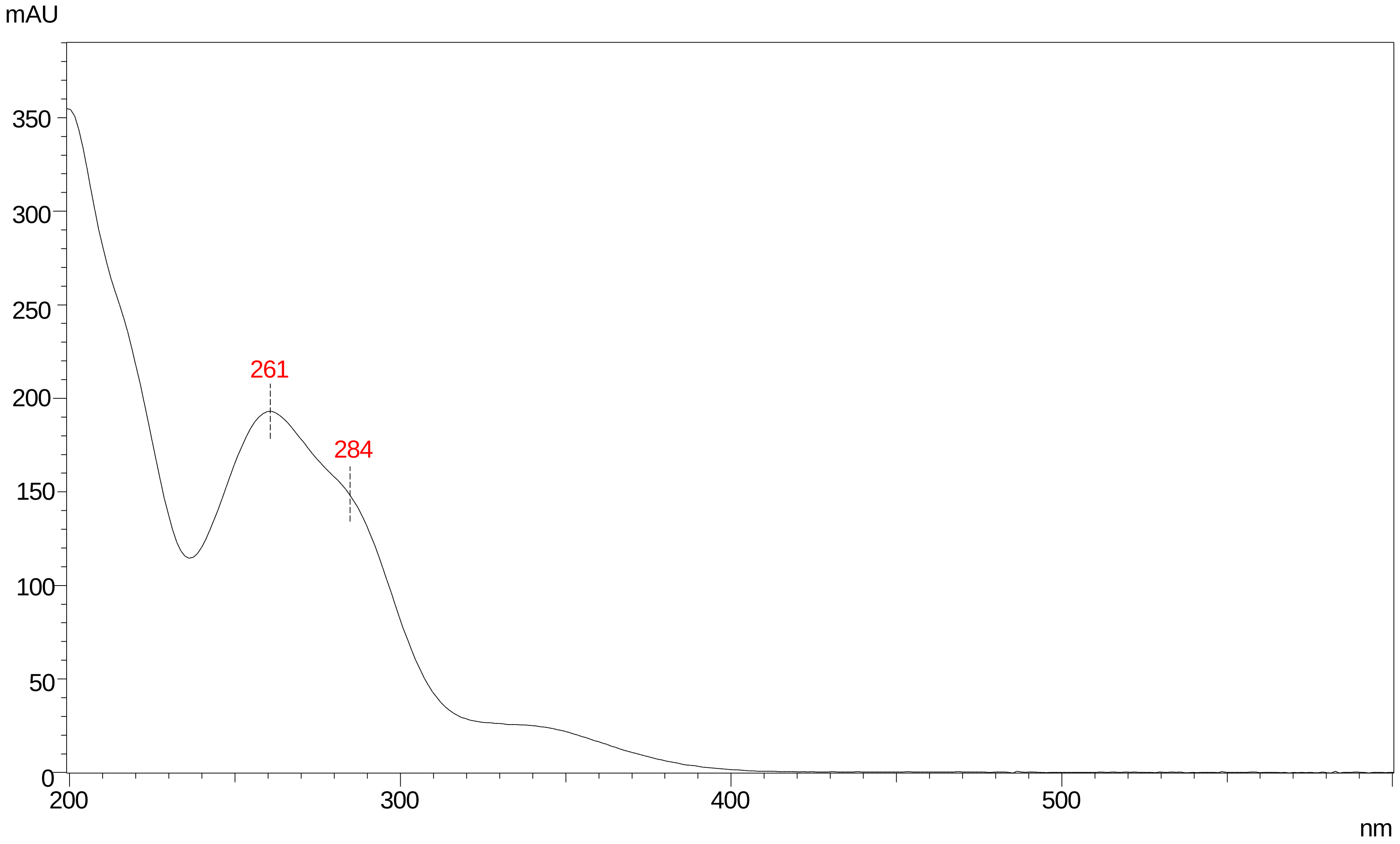

Fig. S33. UV spectrum of **17**

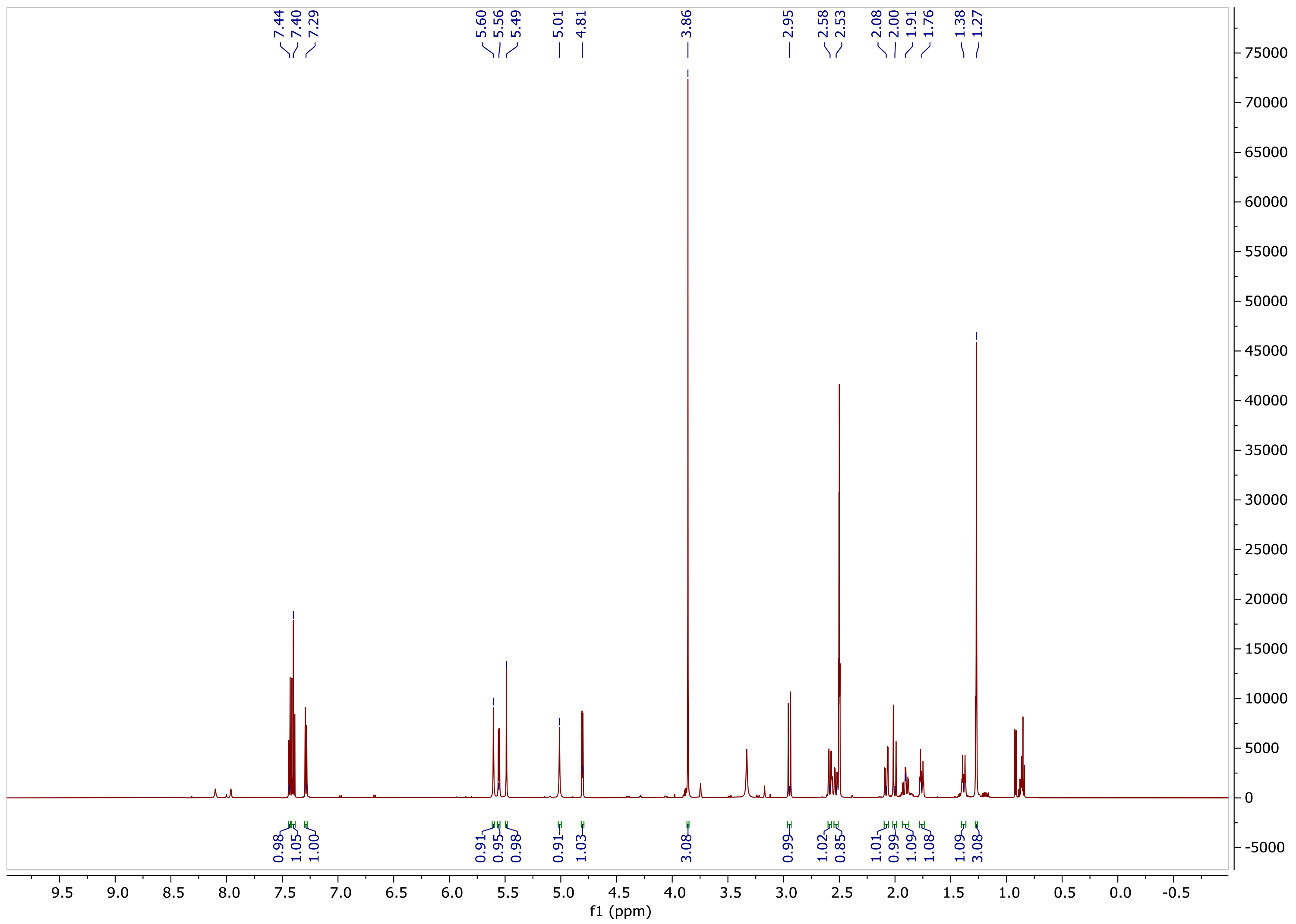

Fig. S34. ^{1}H NMR spectrum of **17** (600 MHz, in DMSO-*d6*)

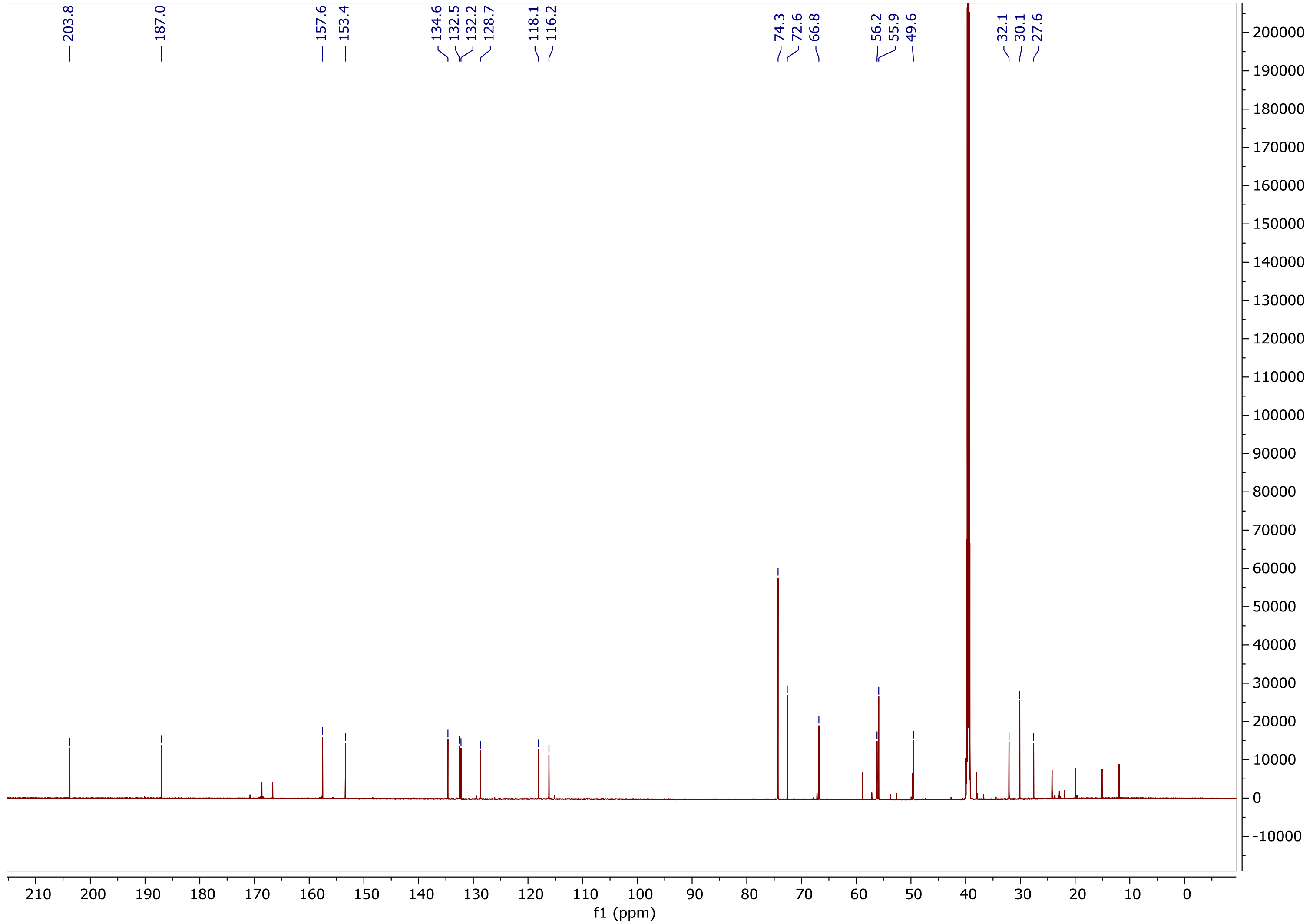

Fig. S35. ^{13}C NMR spectrum of **17** (213 MHz, in DMSO-*d6*)

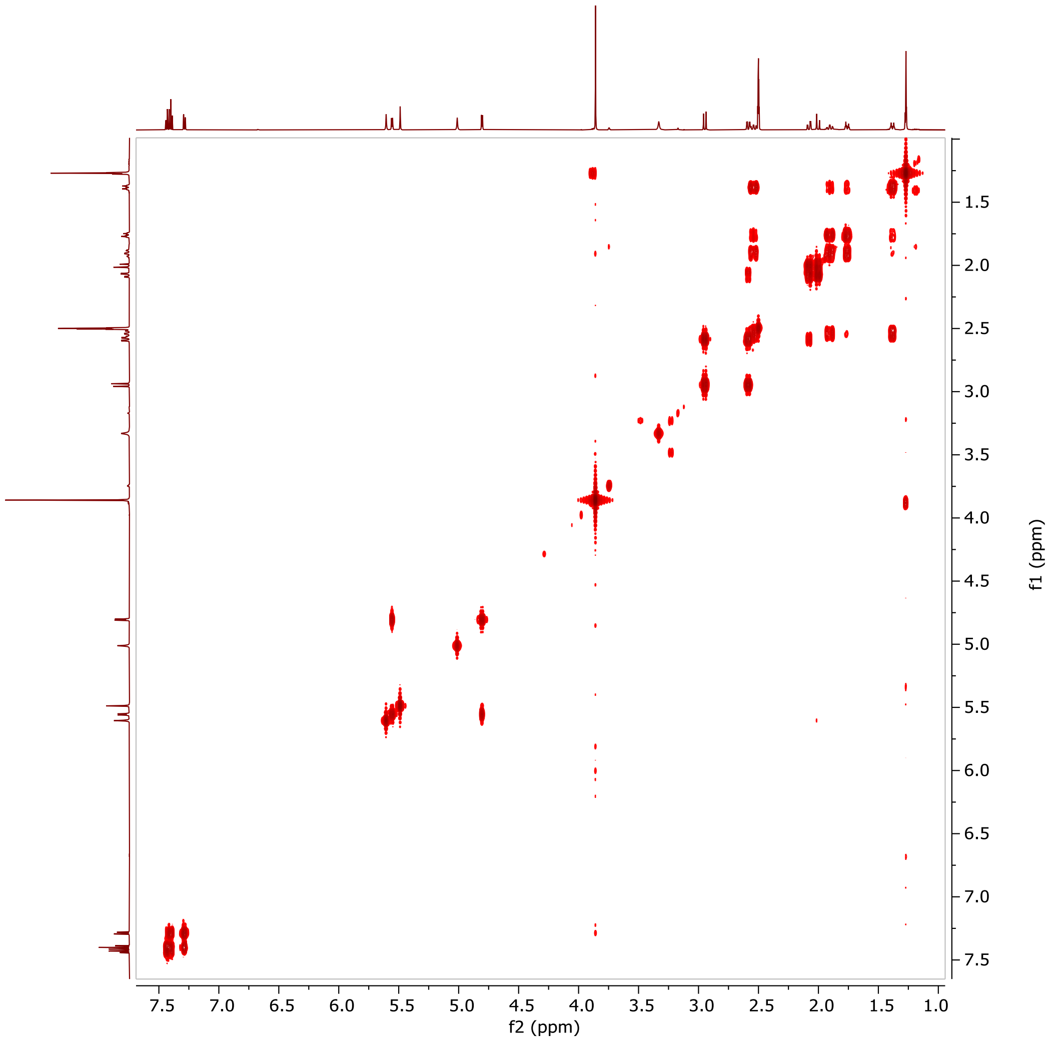

Fig. S36. COSY spectrum of **17** (600 MHz, in DMSO-*d6*)

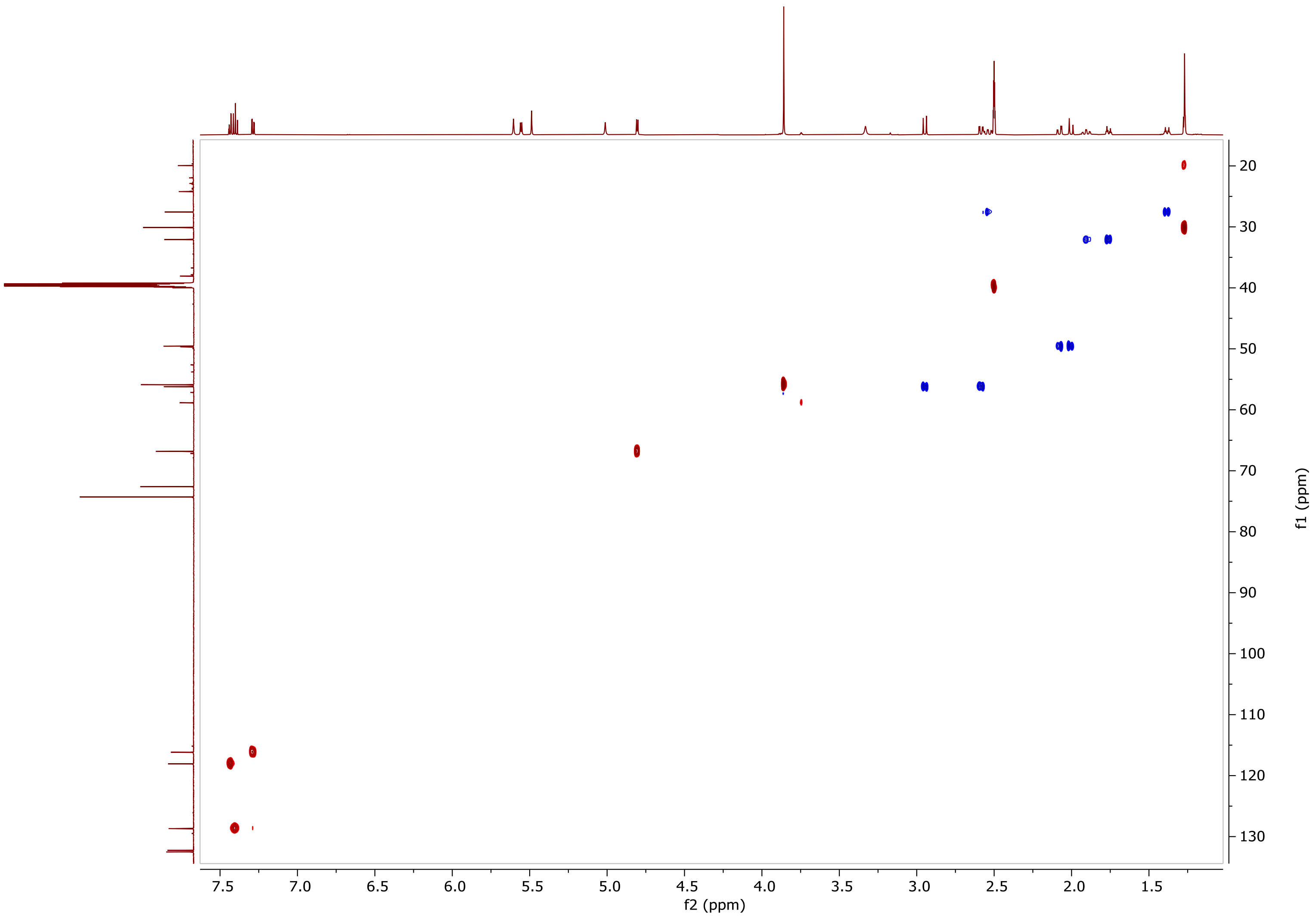

Fig. S37. Multiplicity-edited HSQC spectrum of **17** (600 MHz, in DMSO-*d6*)

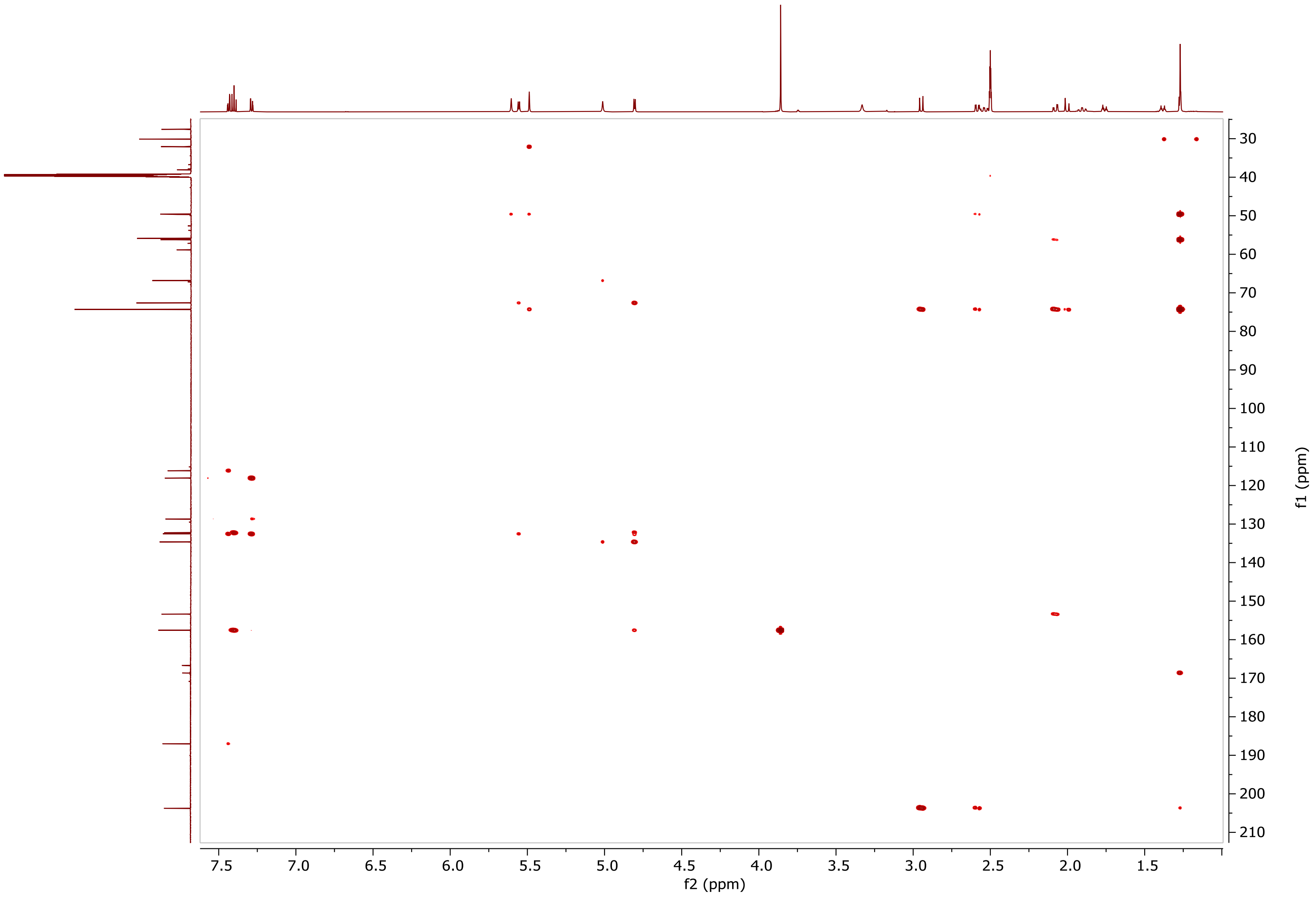

Fig. S38. HMBC spectrum of **17** (600 MHz, in DMSO-*d6*)

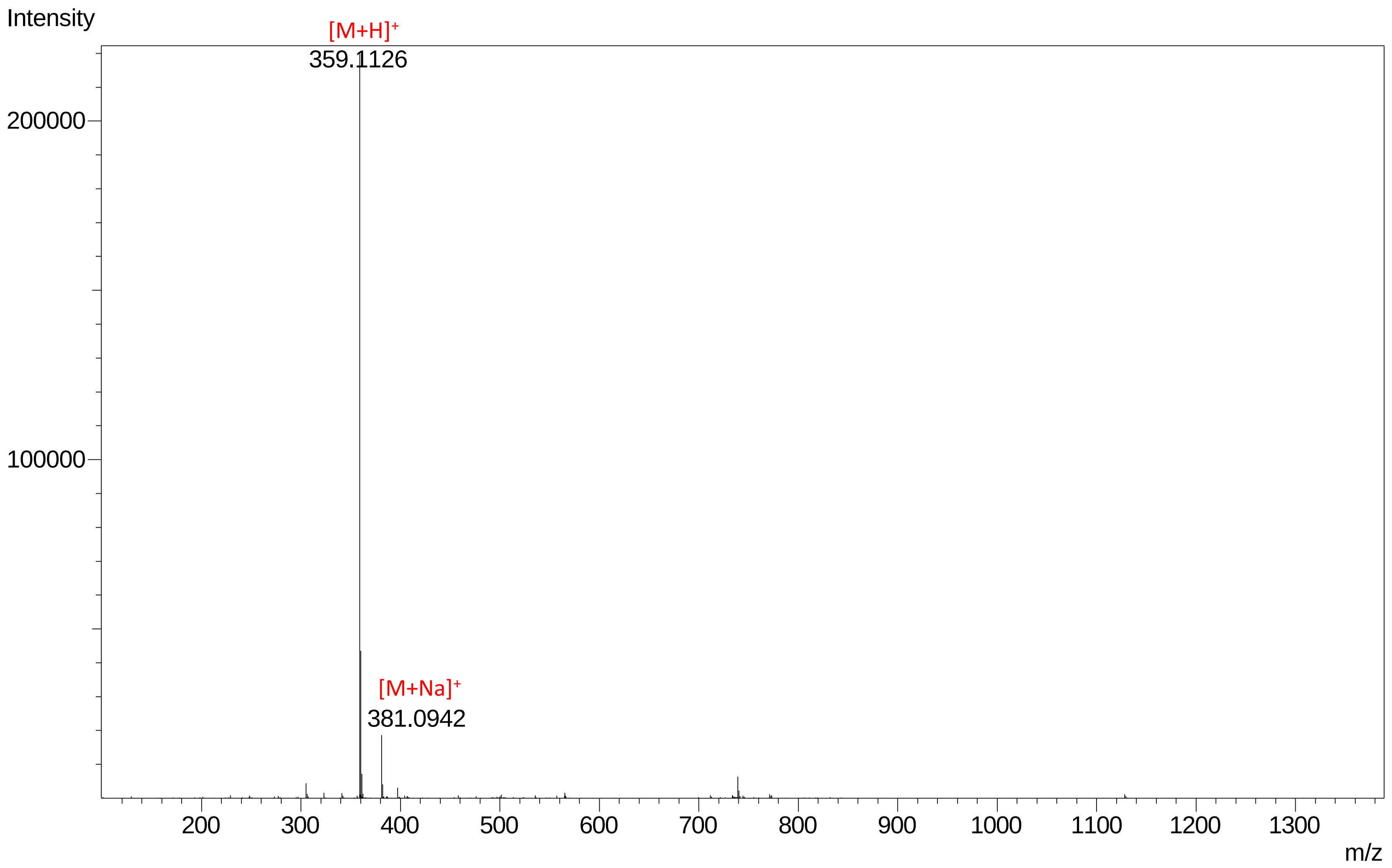

Fig. S39. (+)-HRESIMS spectrum of **18**

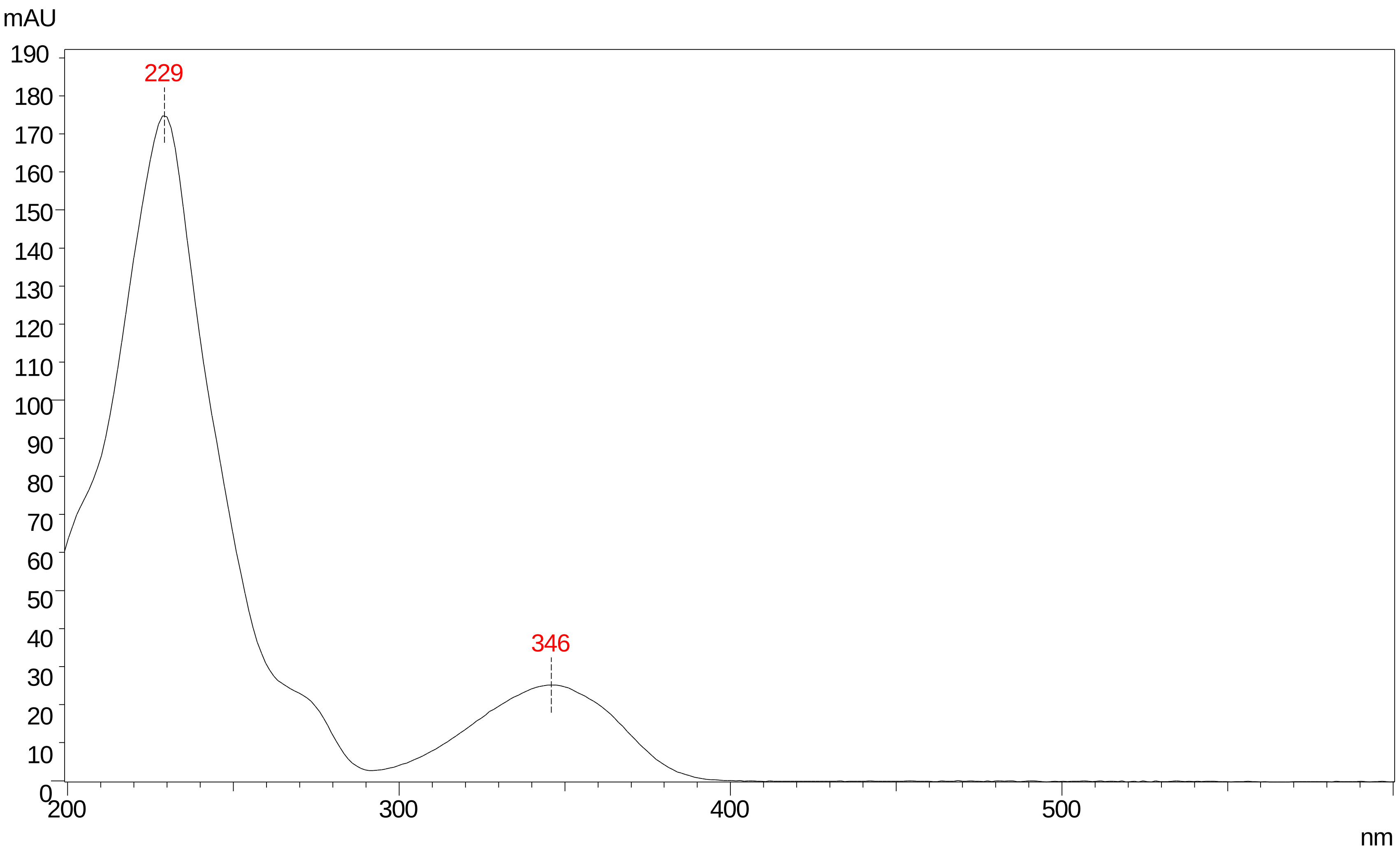

Fig. S40. UV spectrum of **18**

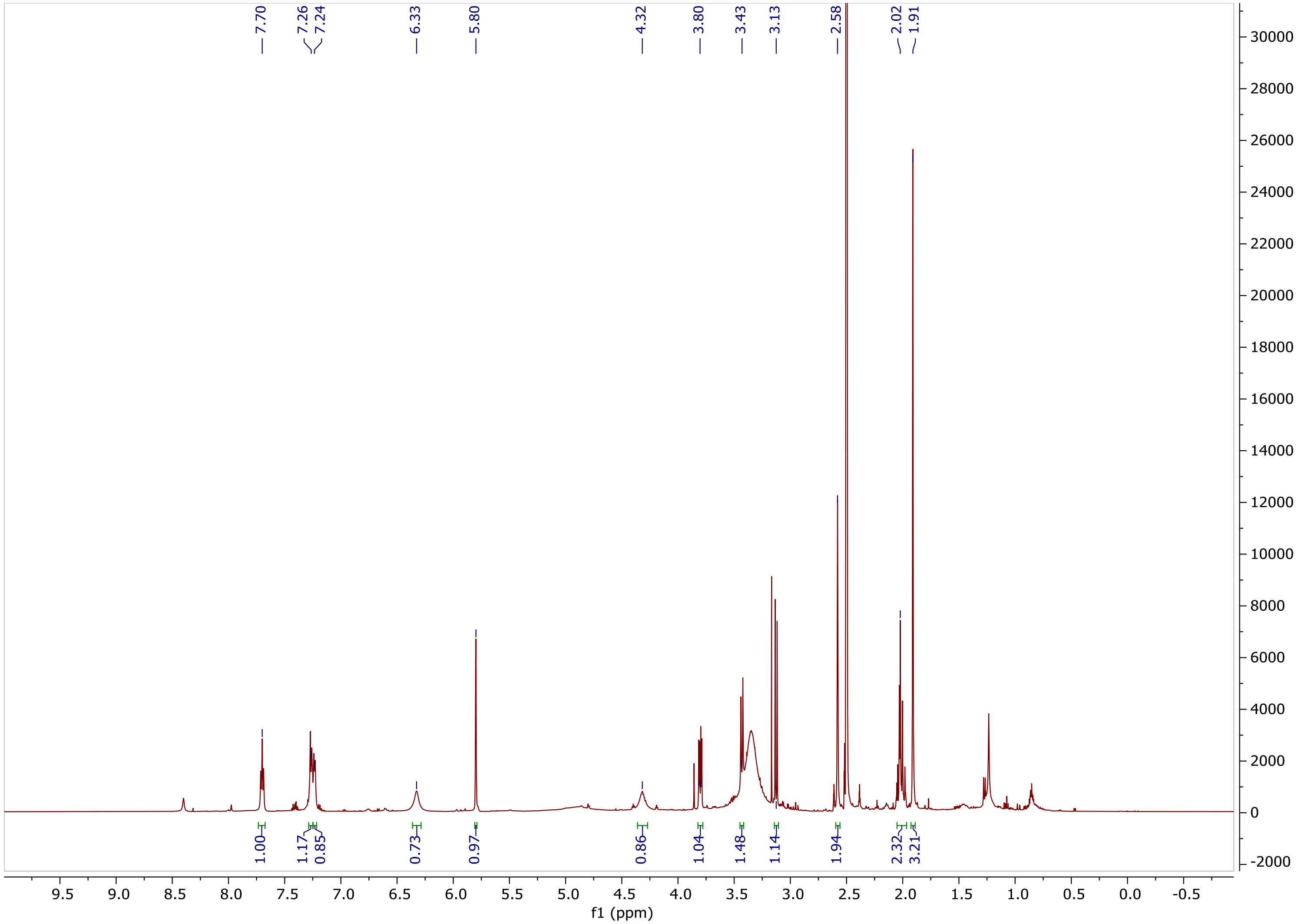

Fig. S41. ^{1}H NMR spectrum of **18** (600 MHz, in DMSO-*d6*)

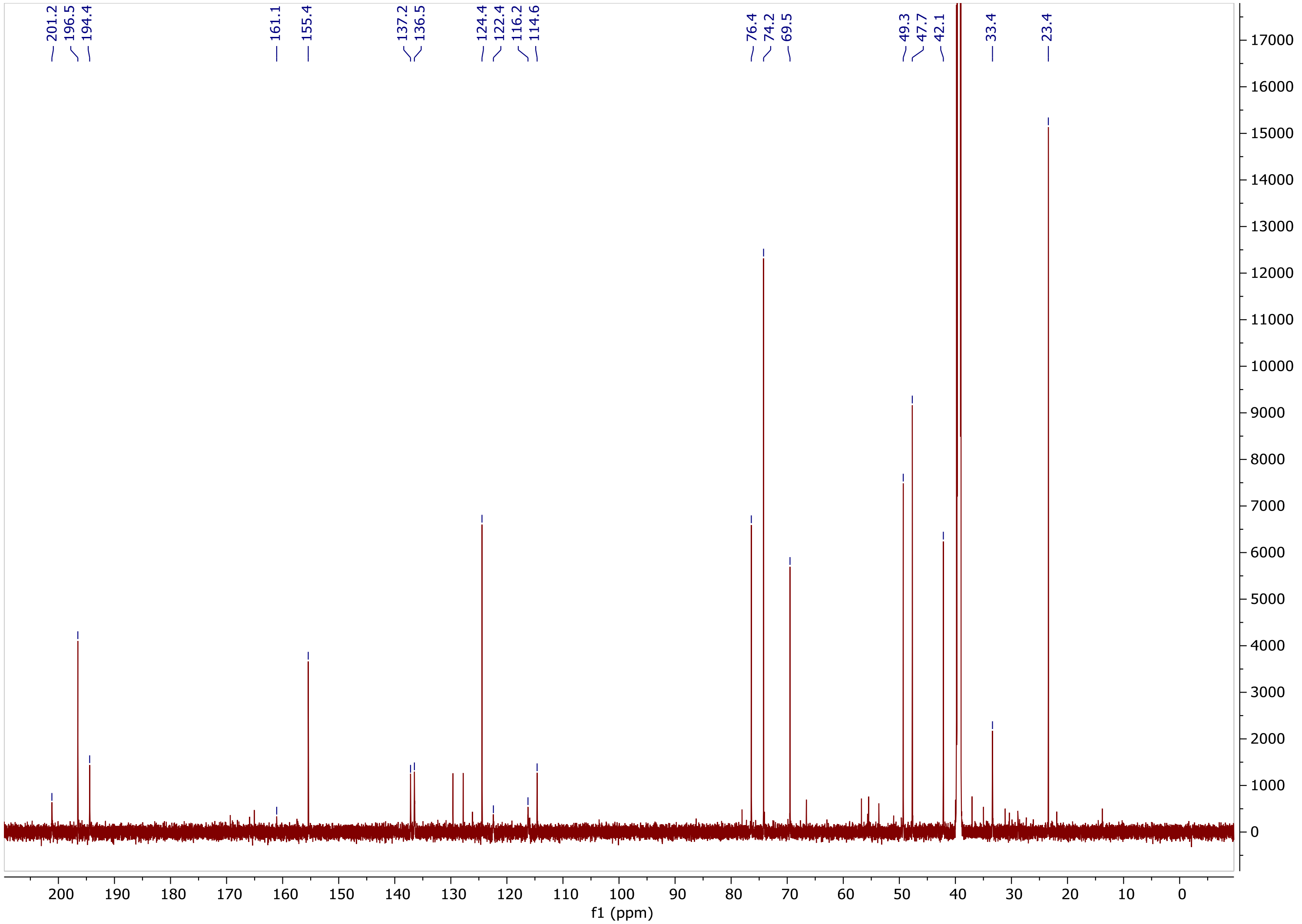

Fig. S42. ^{13}C NMR spectrum of **18** (213 MHz, in DMSO-*d6*)

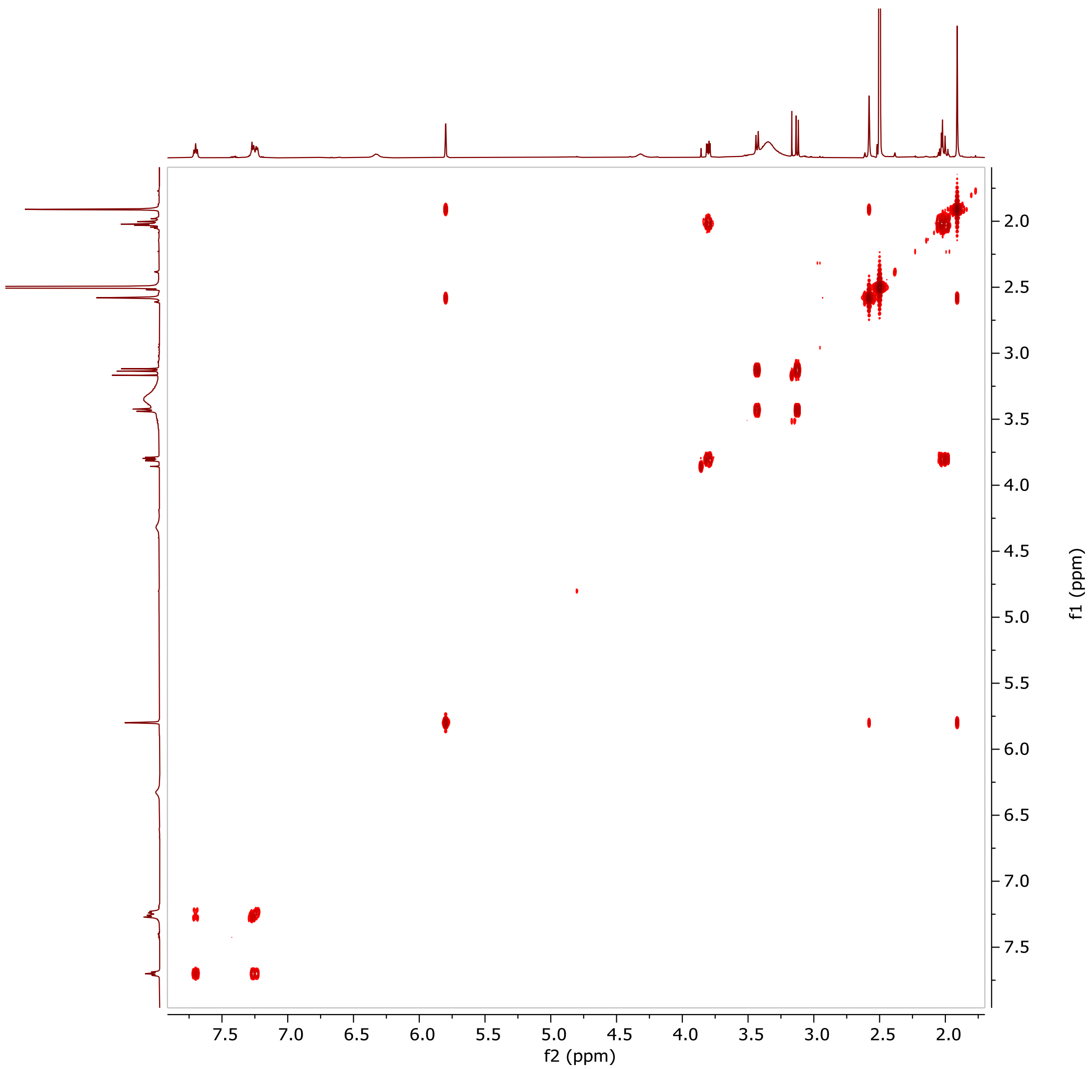

Fig. S43. COSY spectrum of **18** (600 MHz, in DMSO-*d6*)

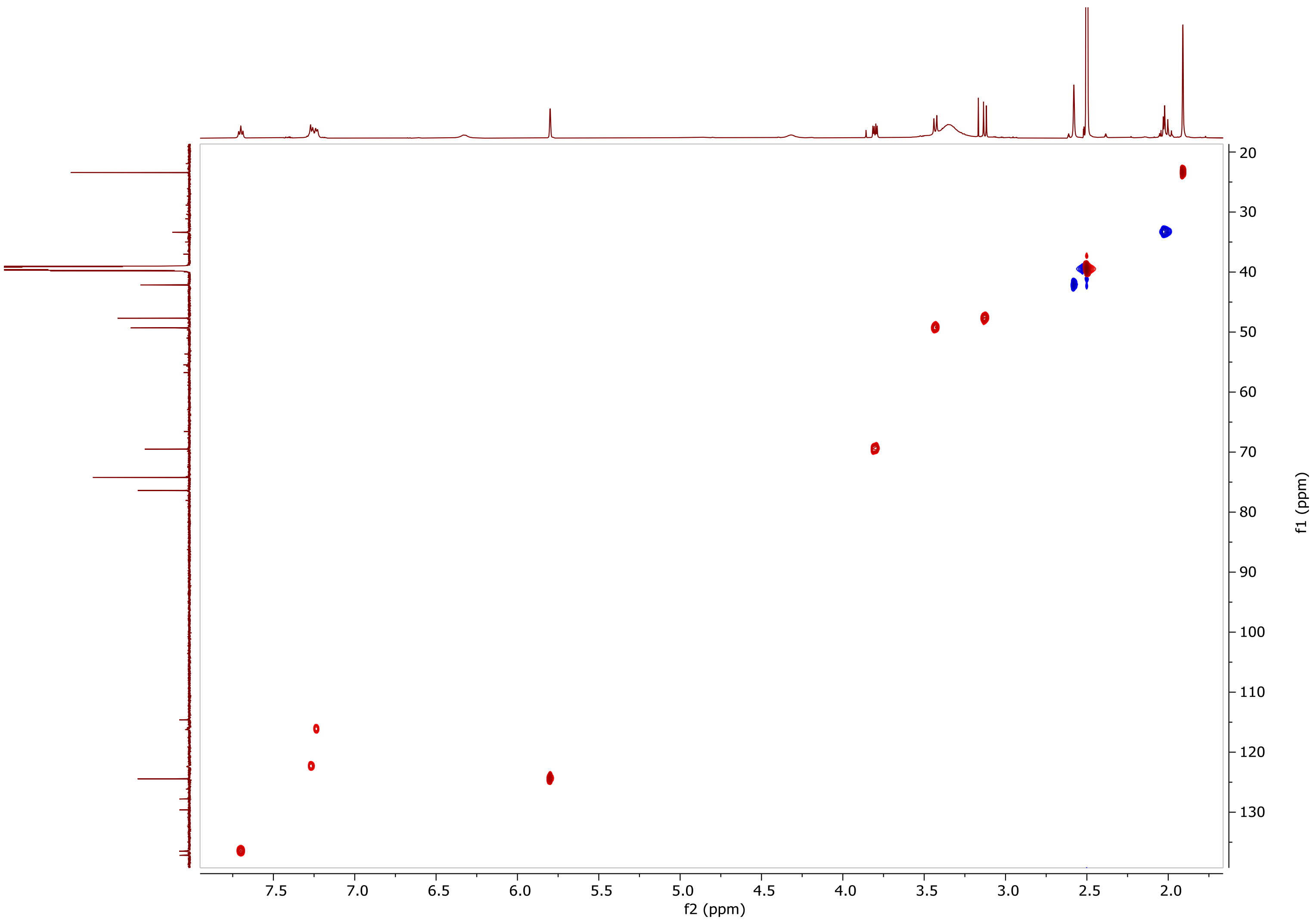

Fig. S44. Multiplicity-edited HSQC spectrum of **18** (600 MHz, in DMSO-*d6*)

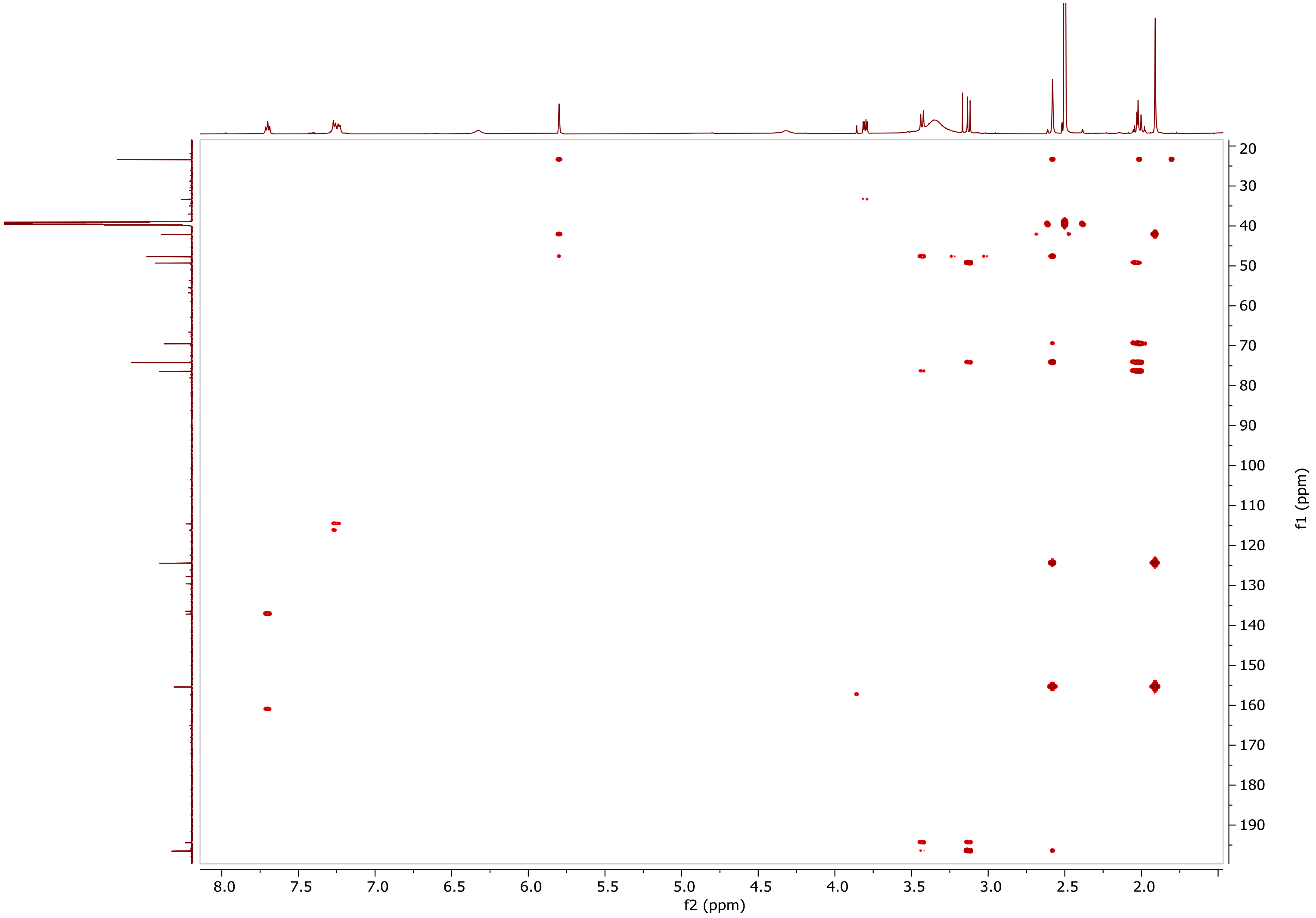

Fig. S45. HMBC spectrum of **18** (600 MHz, in DMSO-*d6*)

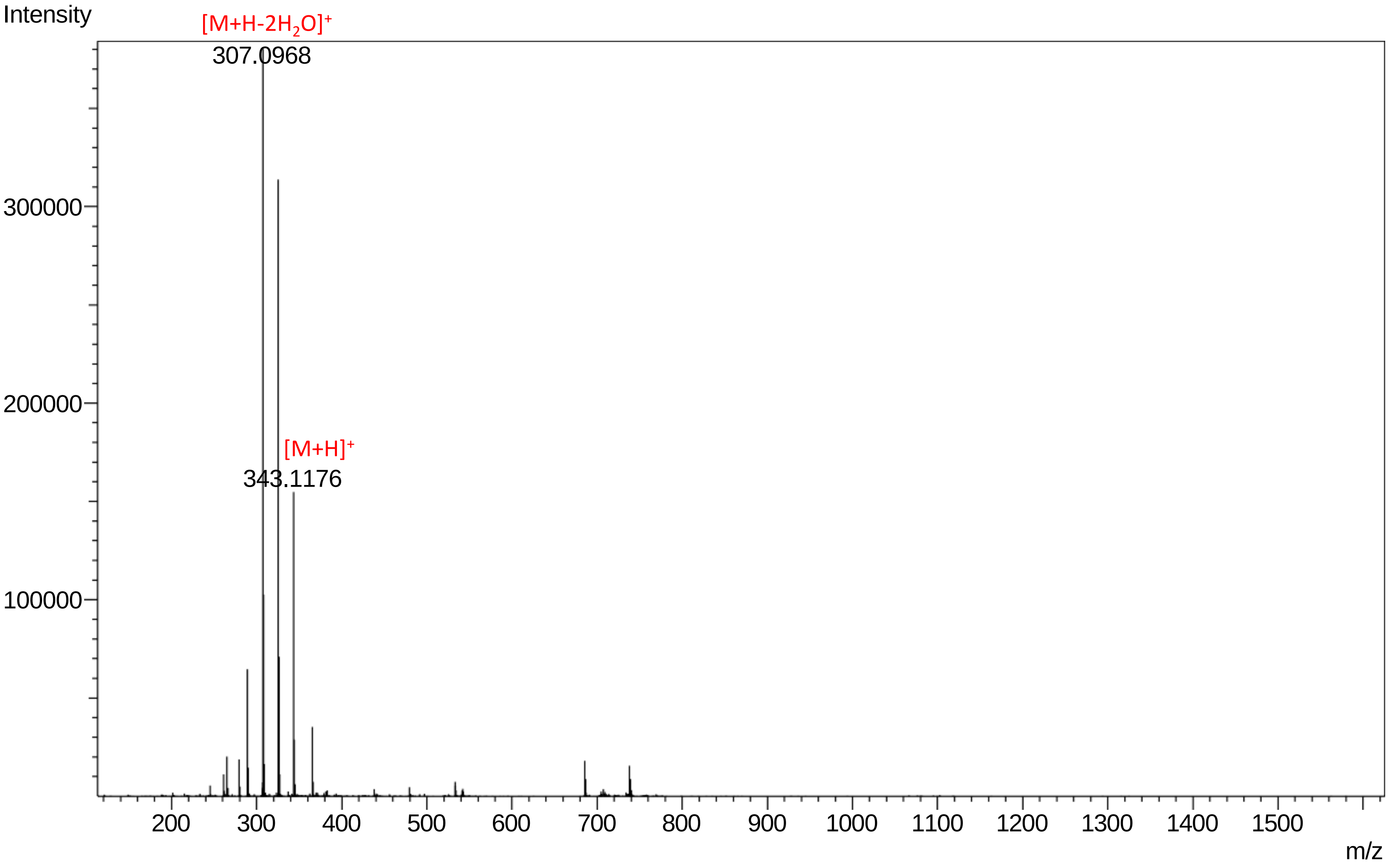

Fig. S46. (+)-HRESIMS spectrum of **19**

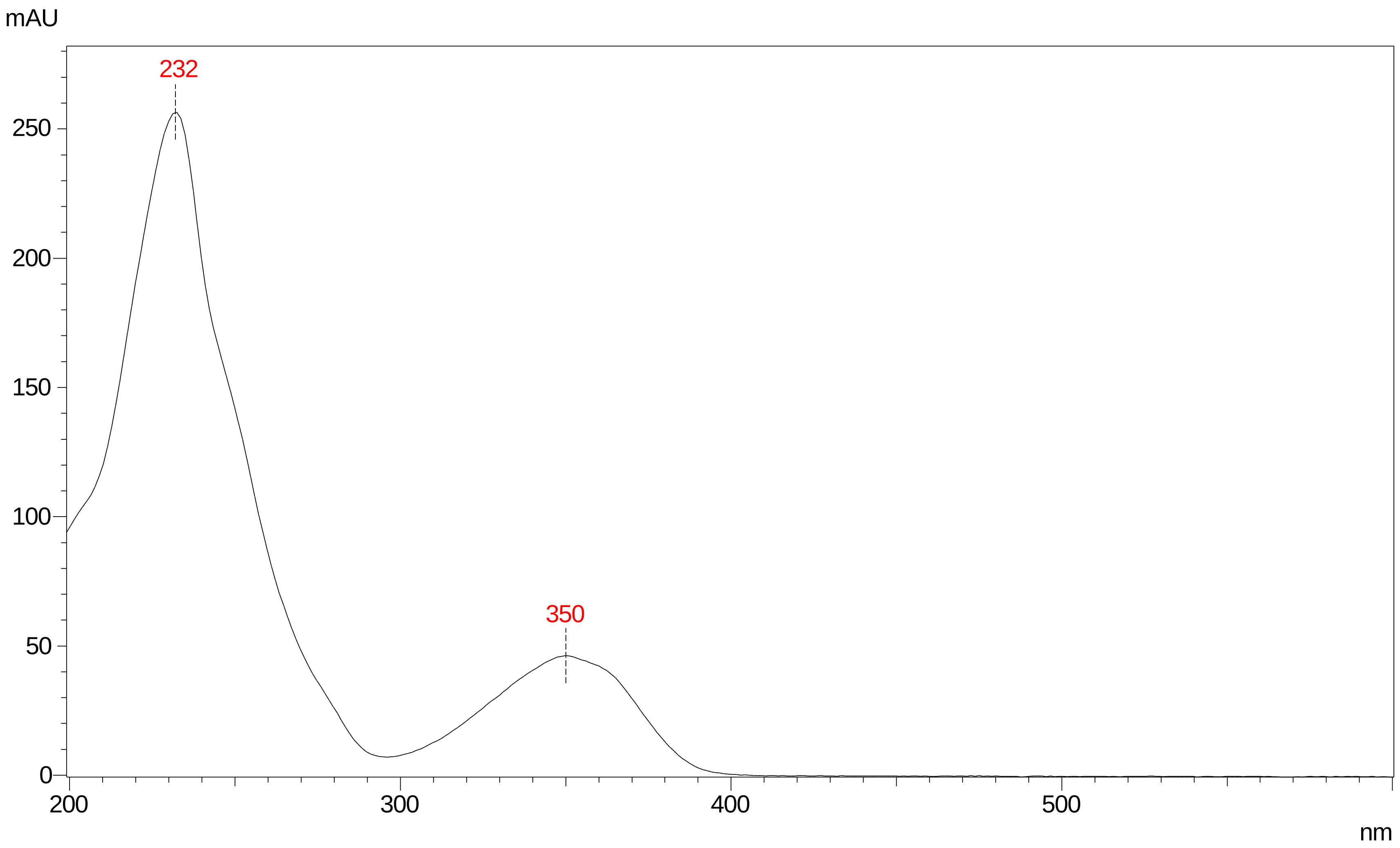

Fig. S47. UV spectrum of **19**

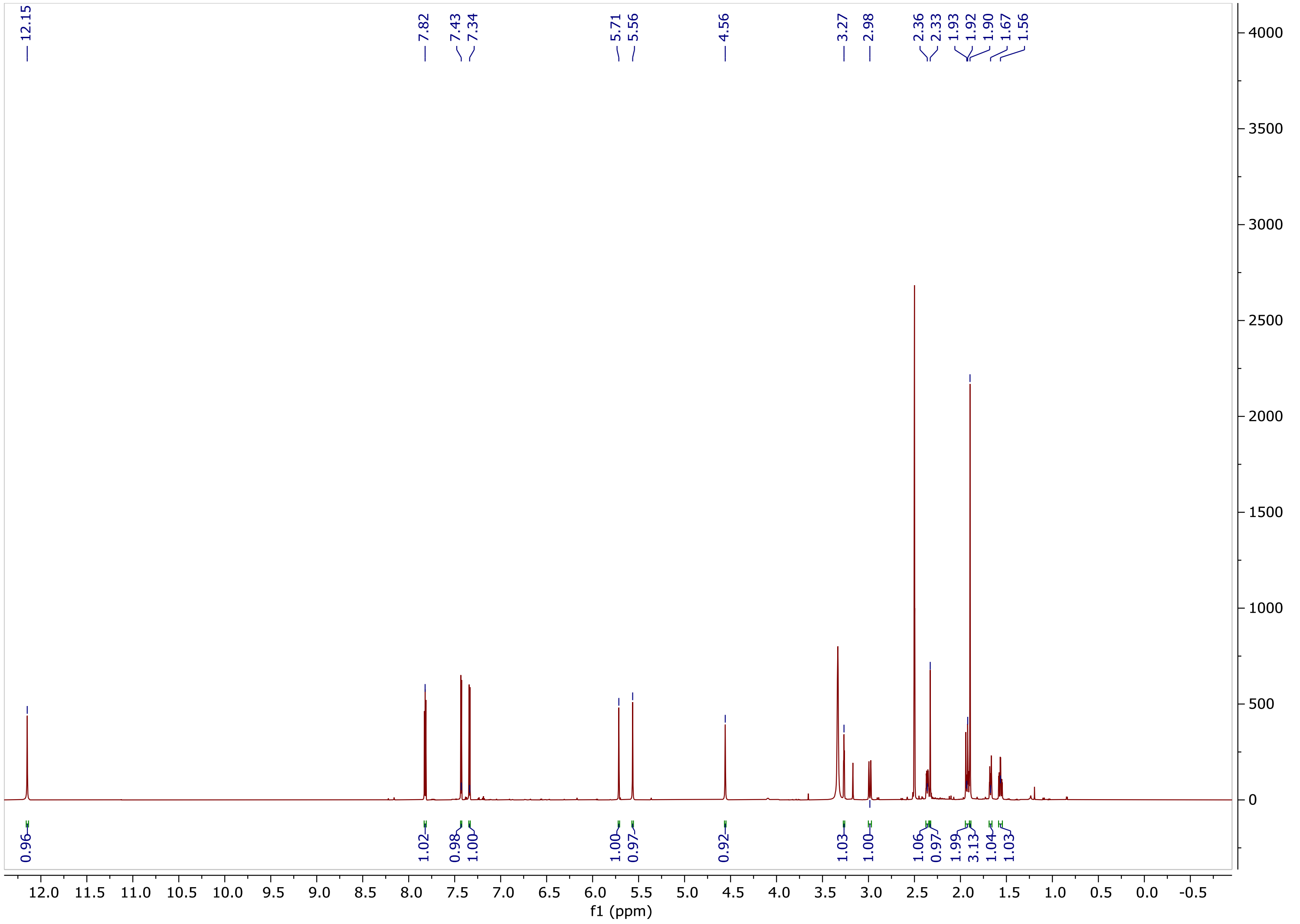

Fig. S48. ^{1}H NMR spectrum of **19** (850 MHz, in DMSO-*d6*)

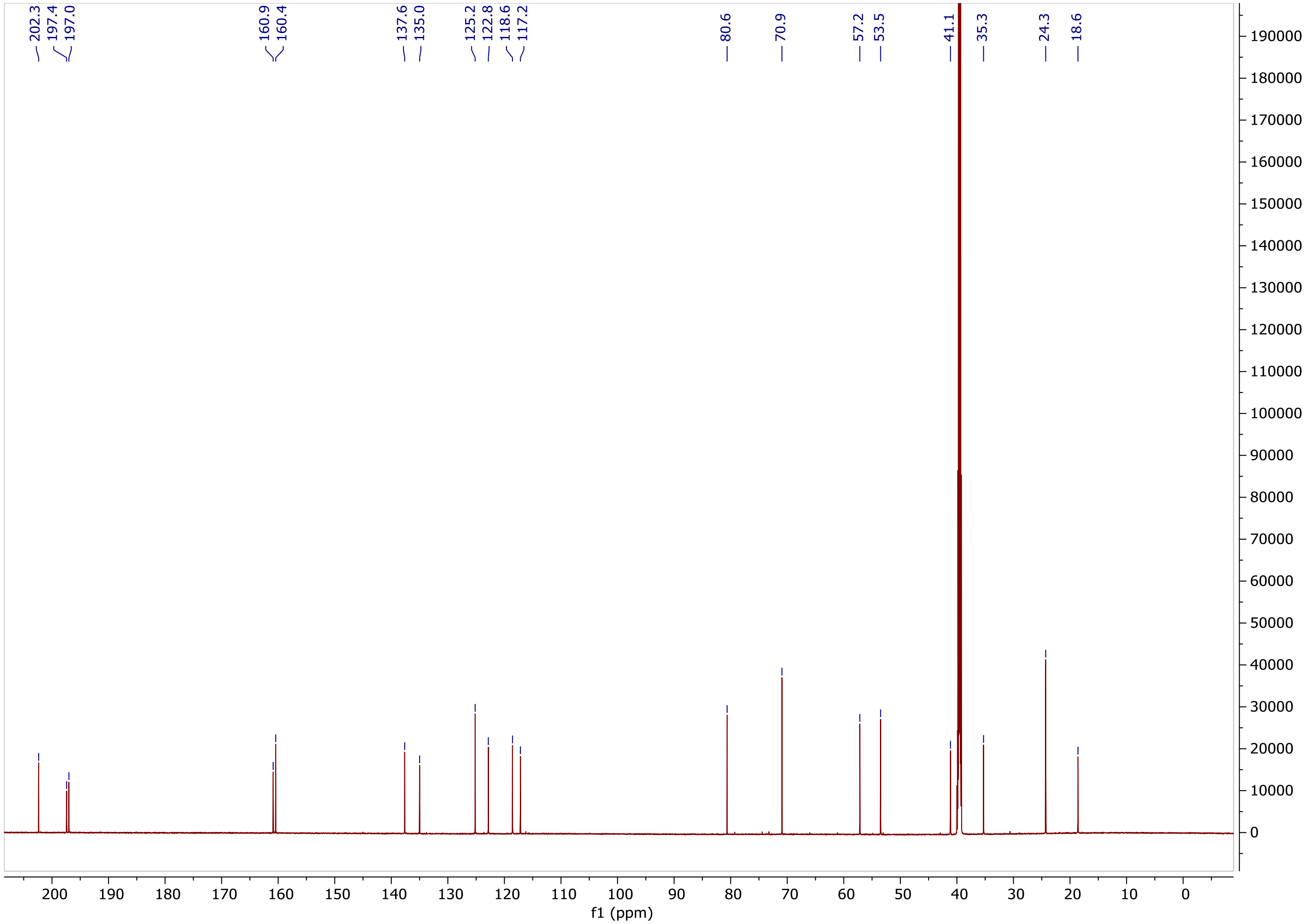

Fig. S49. ^{13}C NMR spectrum of **19** (213 MHz, in DMSO-*d6*)

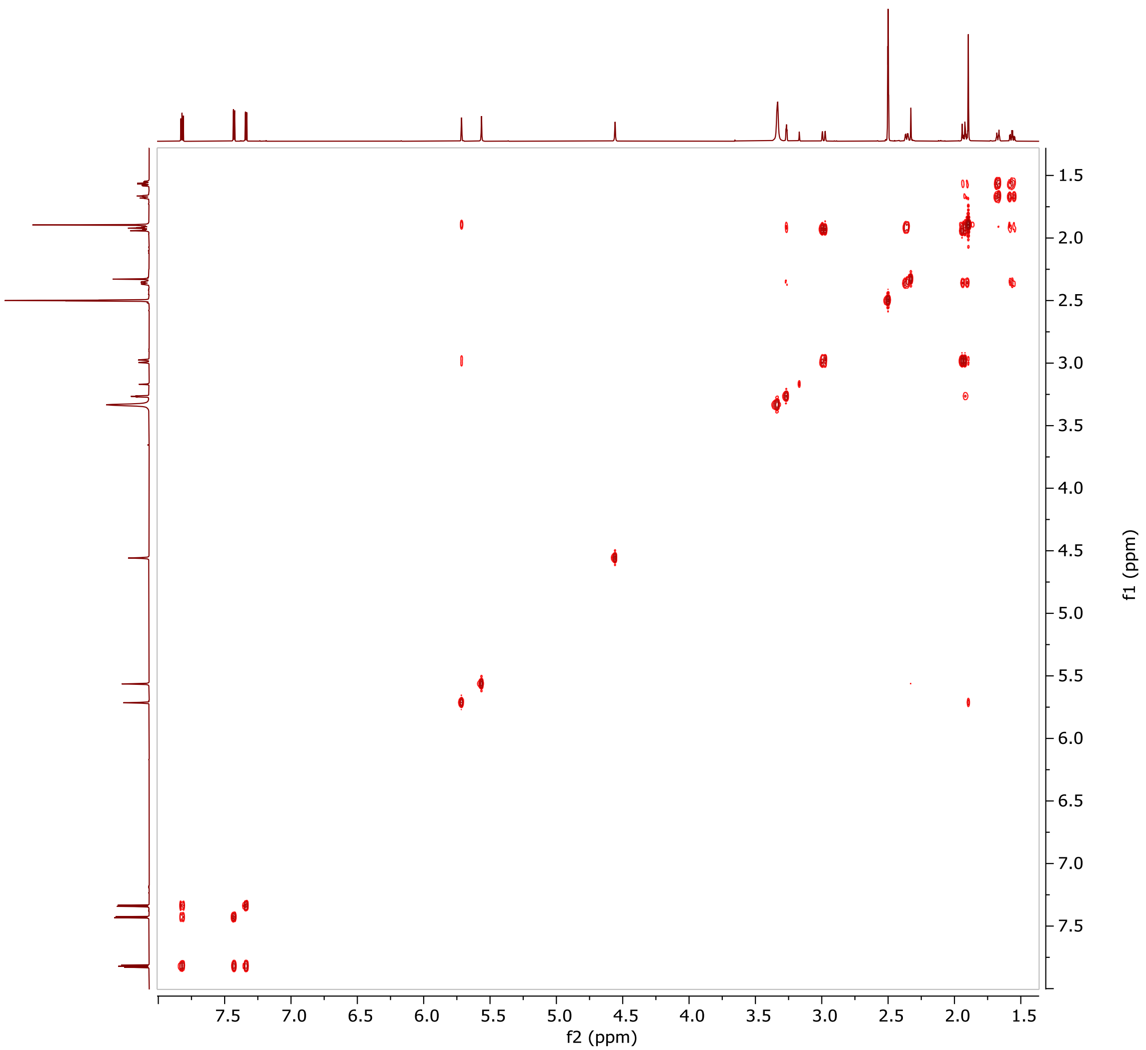

Fig. S50. COSY spectrum of **19** (850 MHz, in DMSO-*d6*)

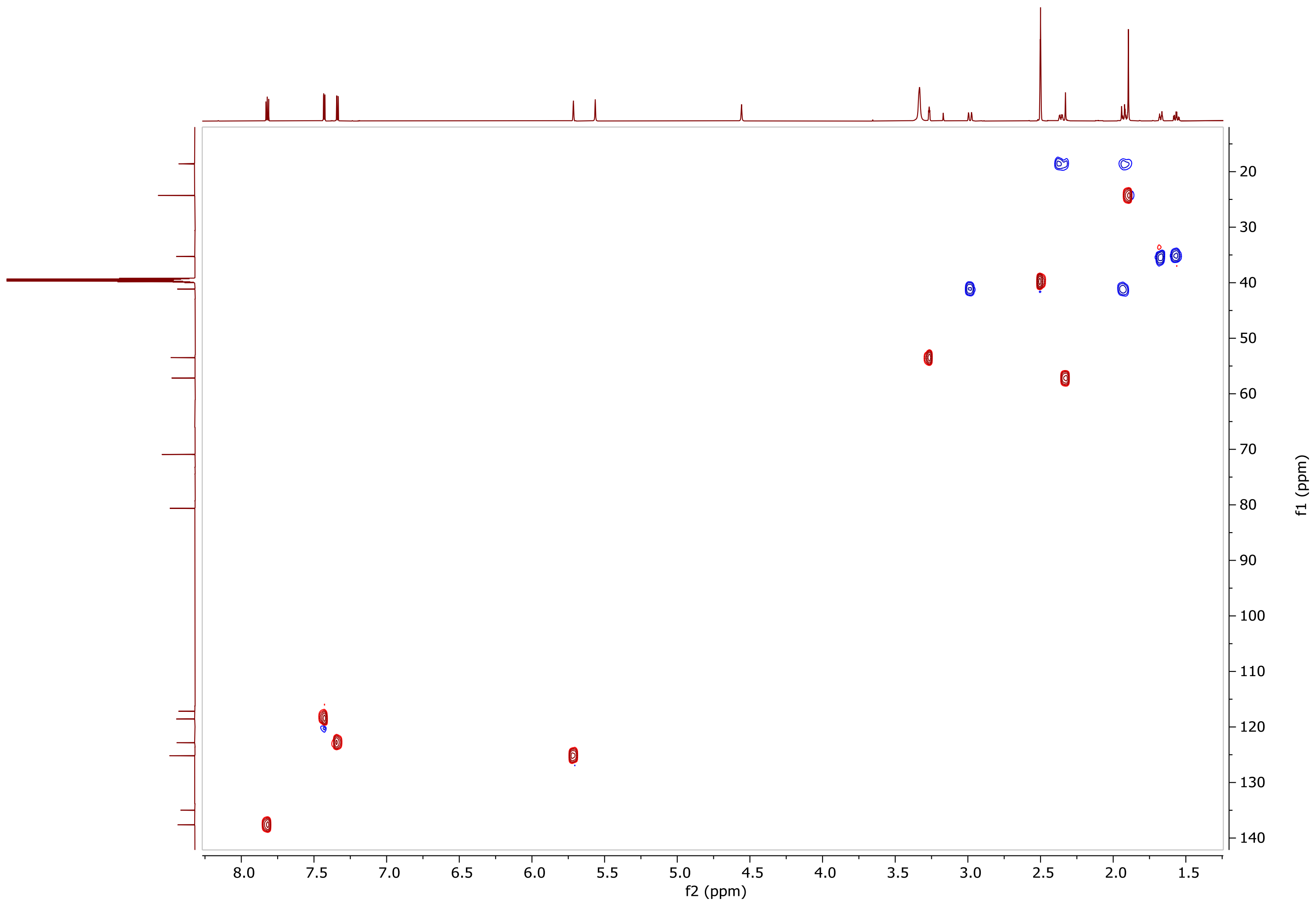

Fig. S51. Multiplicity-edited HSQC spectrum of **19** (850 MHz, in DMSO-*d6*)

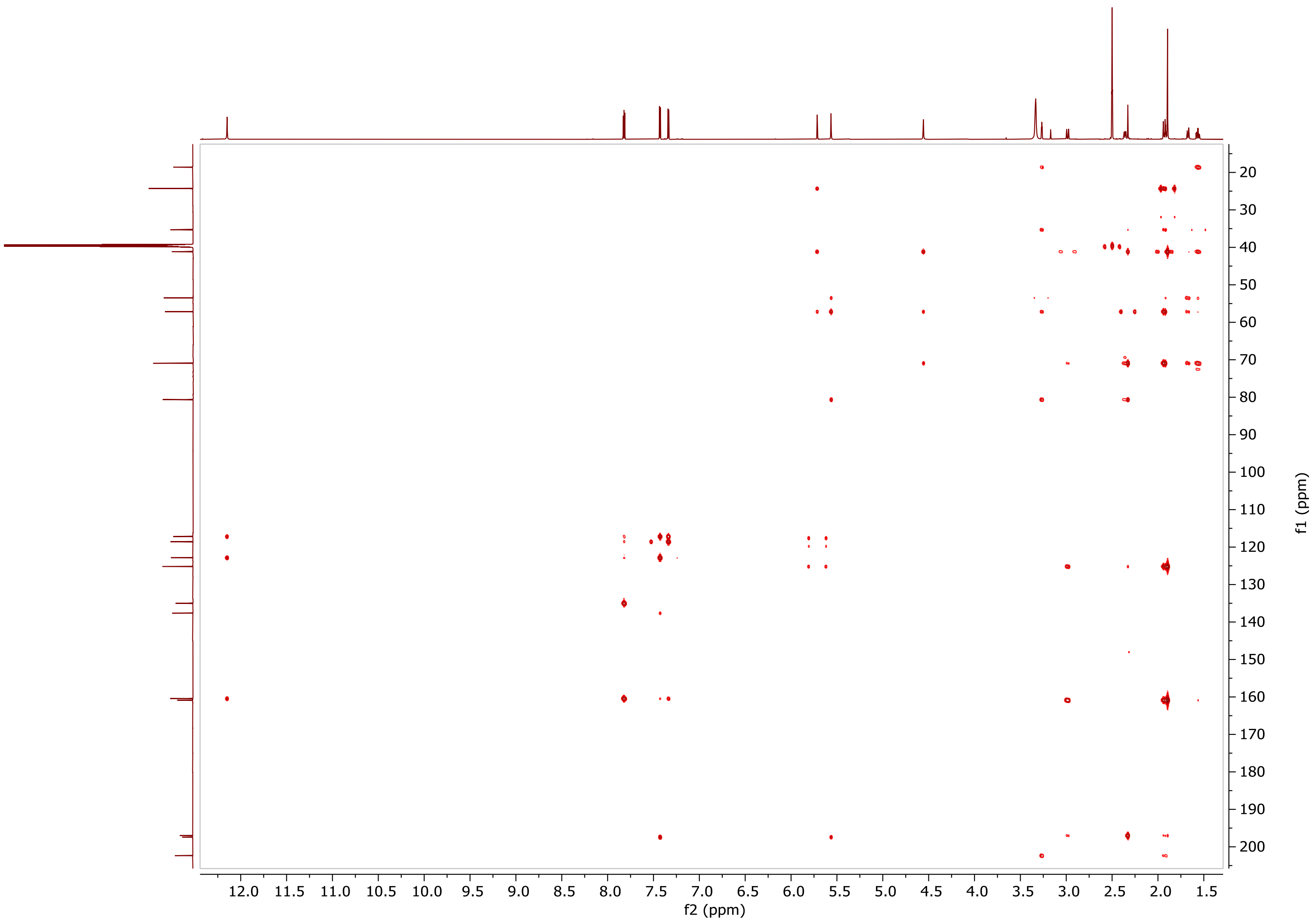

Fig. S52. HMBC spectrum of **19** (850 MHz, in DMSO-*d6*)

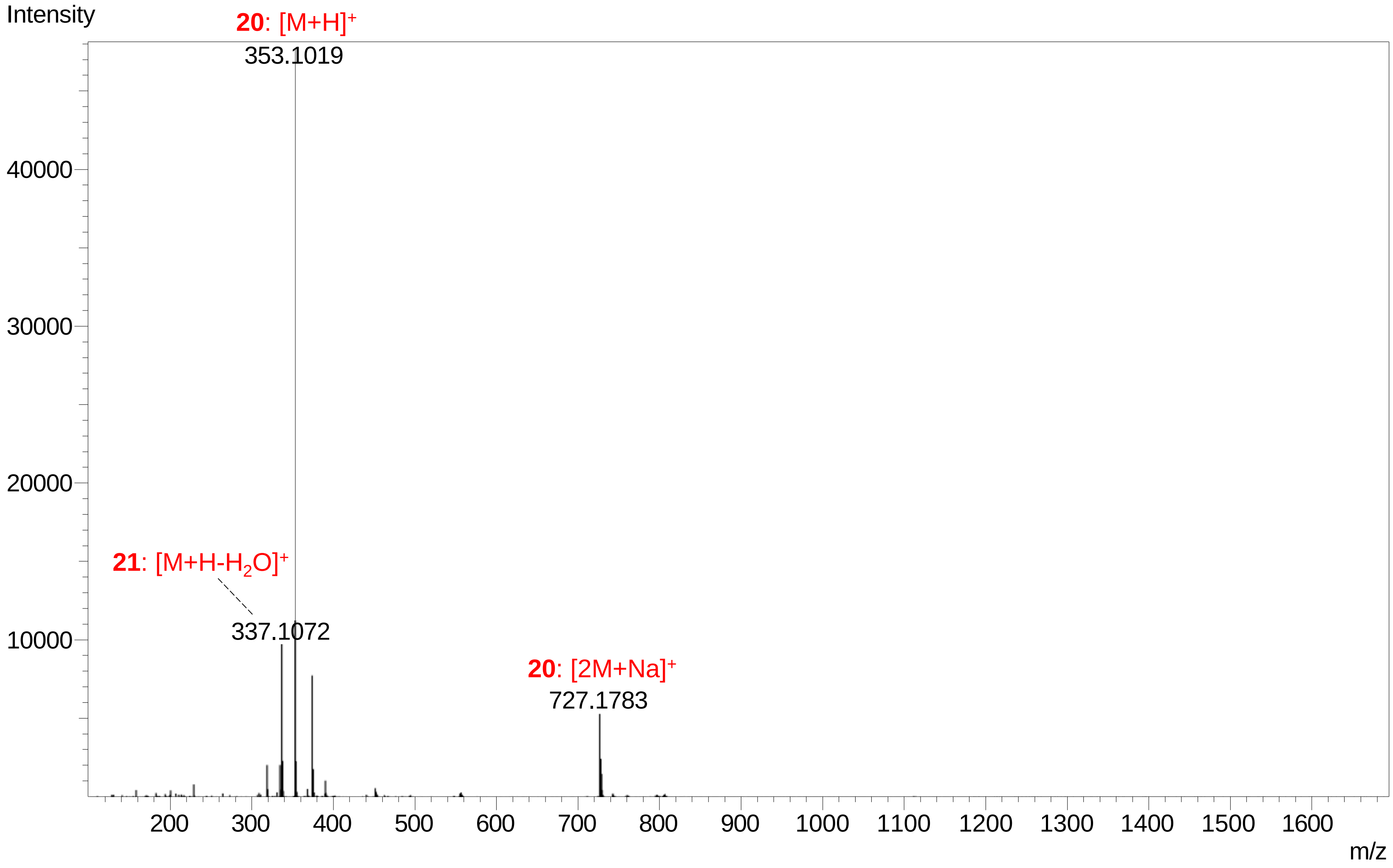

Fig. S53. (+)-HRESIMS spectrum of **20** and **21**

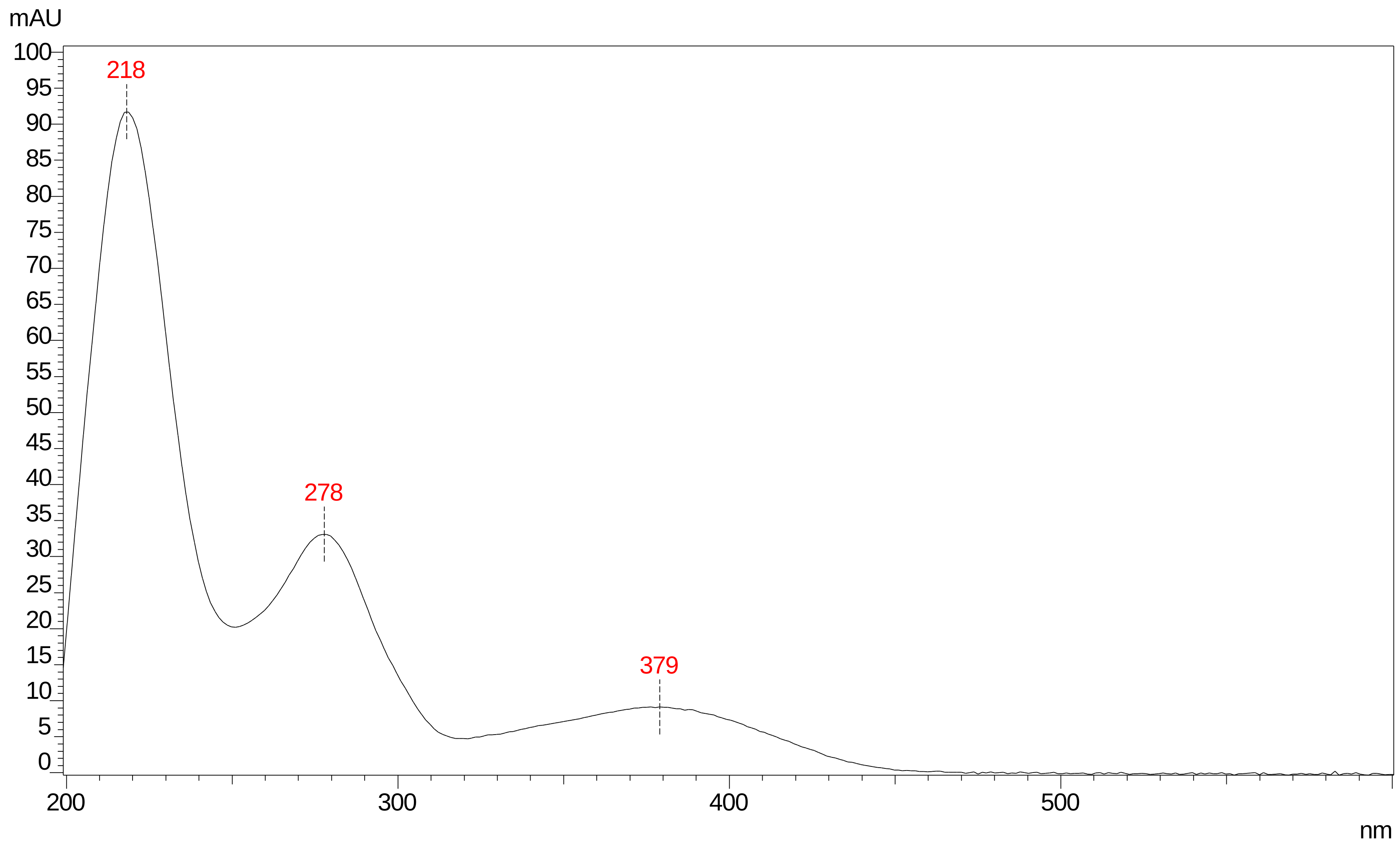

Fig. S54. UV spectrum of **20** and **21**

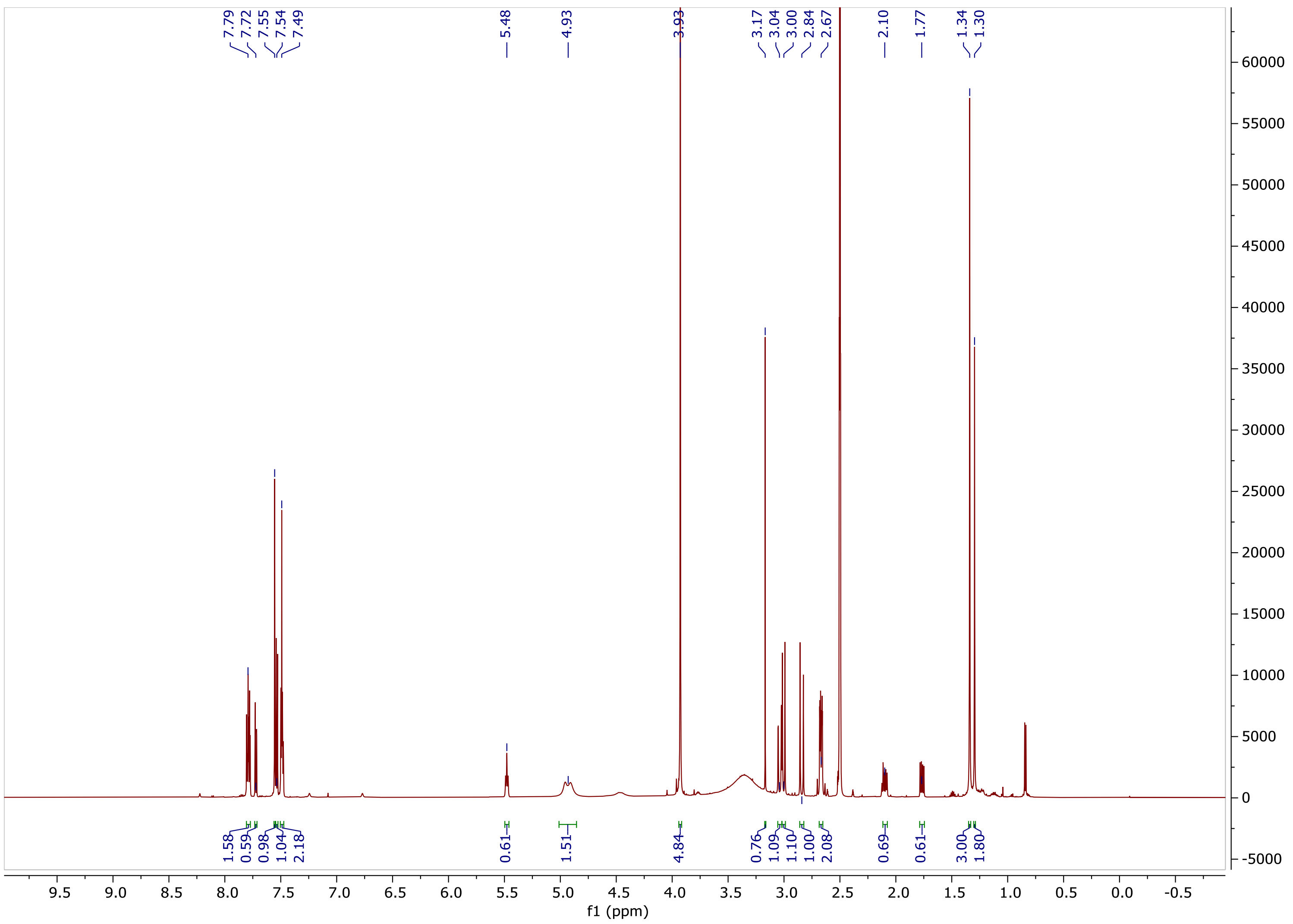

Fig. S55. ^{1}H NMR spectrum of **20** and **21** (600 MHz, in DMSO-*d6*)

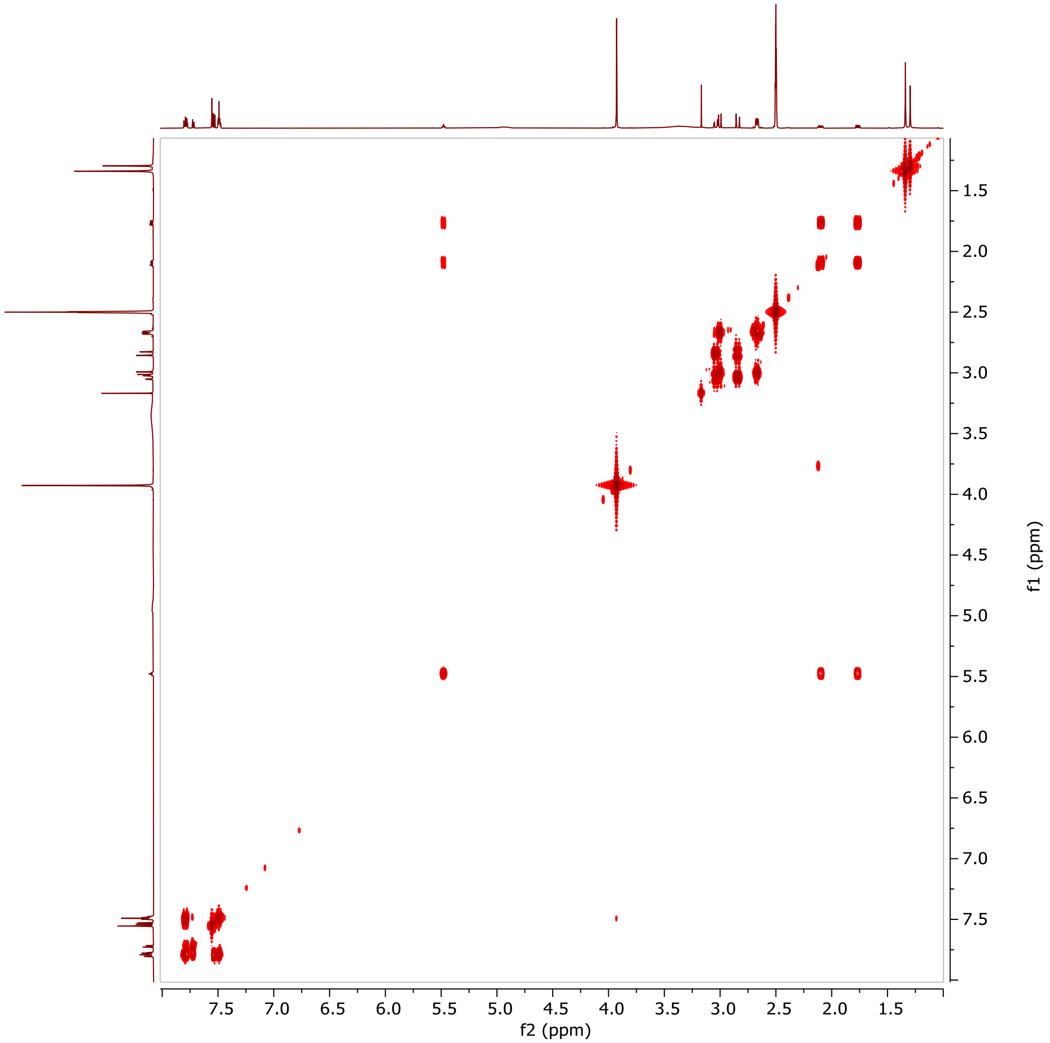

Fig. S56. COSY spectrum of **20** and **21** (600 MHz, in DMSO-*d6*)

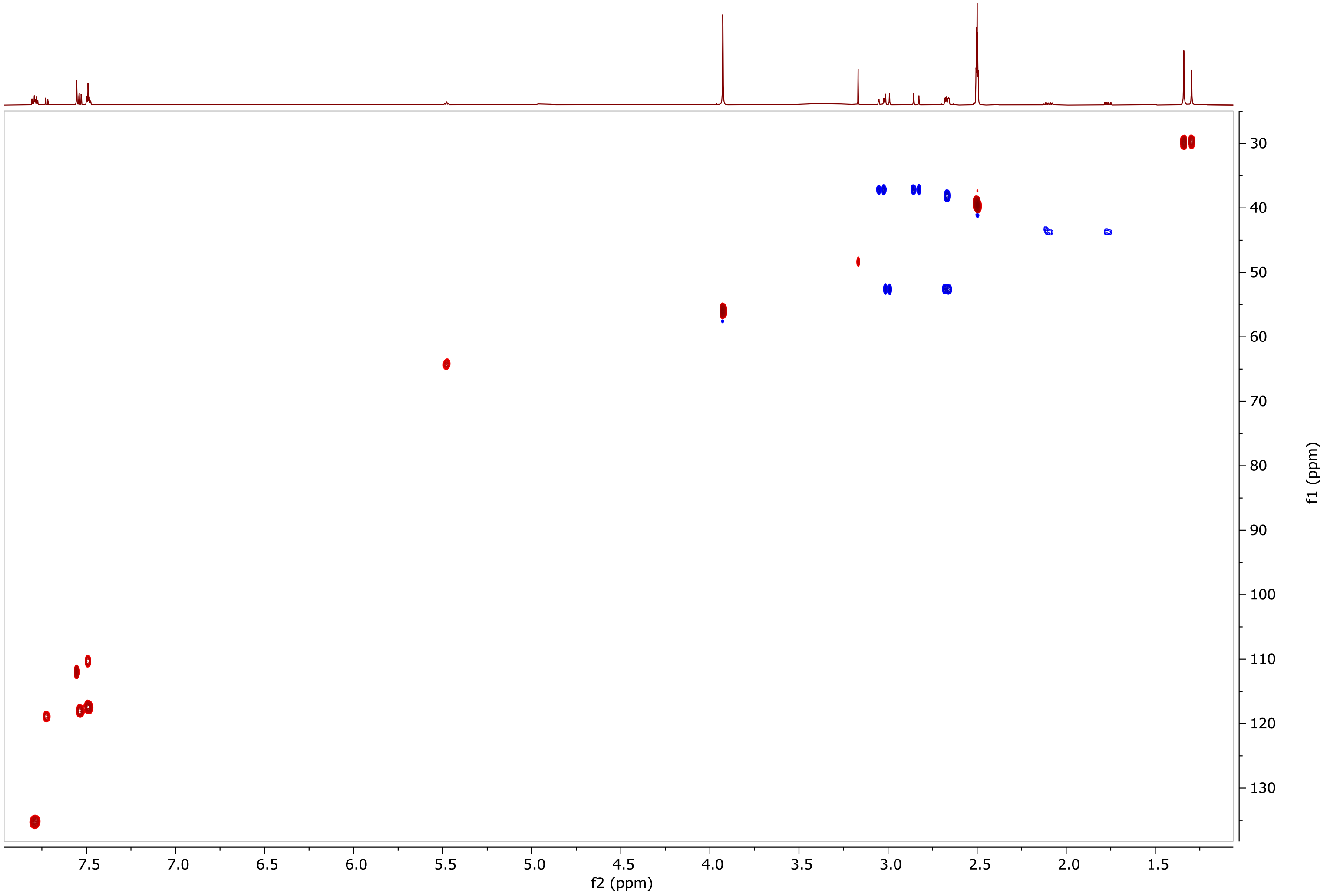

Fig. S57. Multiplicity-edited HSQC spectrum of **20** and **21** (600 MHz, in DMSO-*d6*)

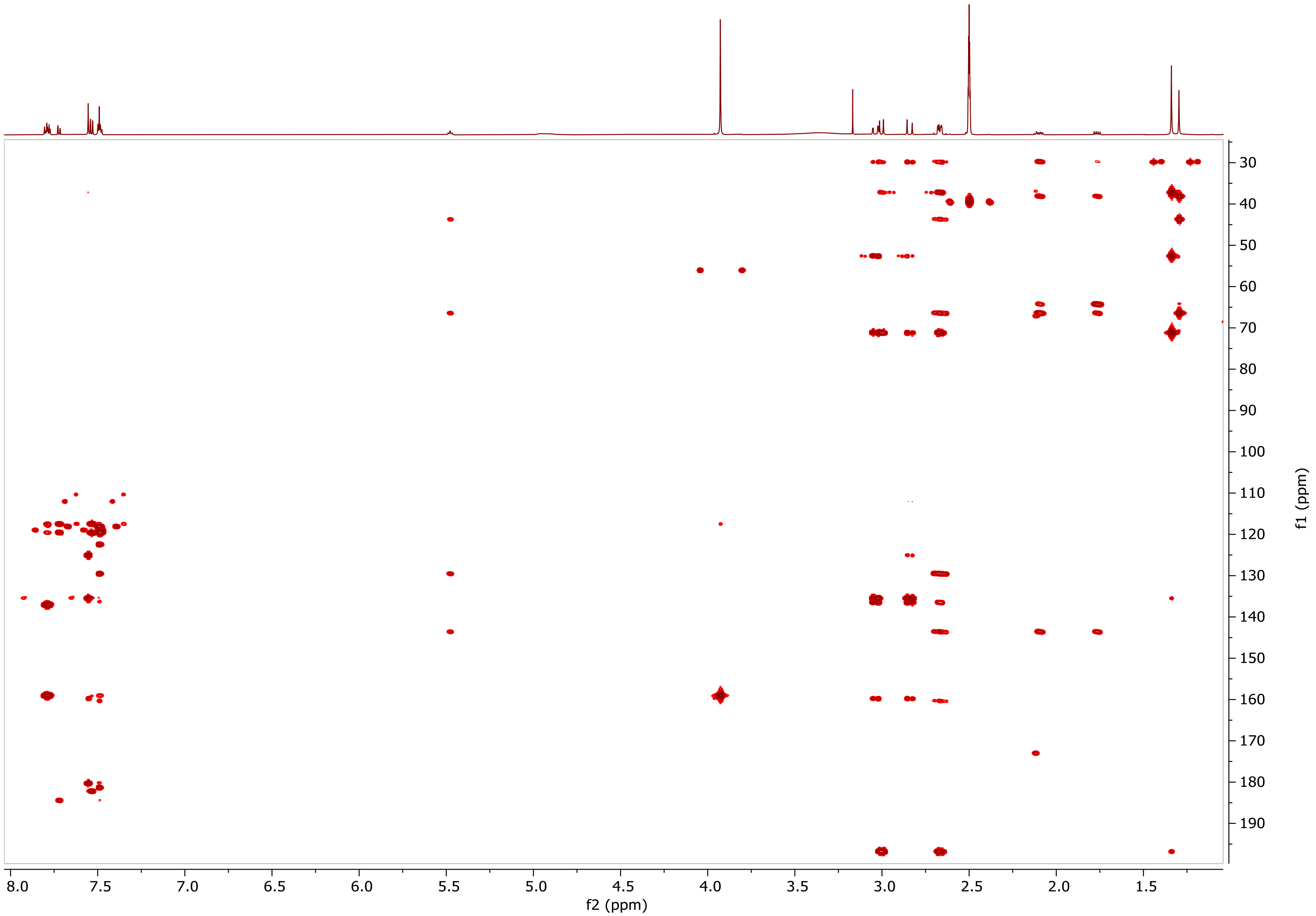

Fig. S58. HMBC spectrum of **20** and **21** (600 MHz, in DMSO-*d6*)

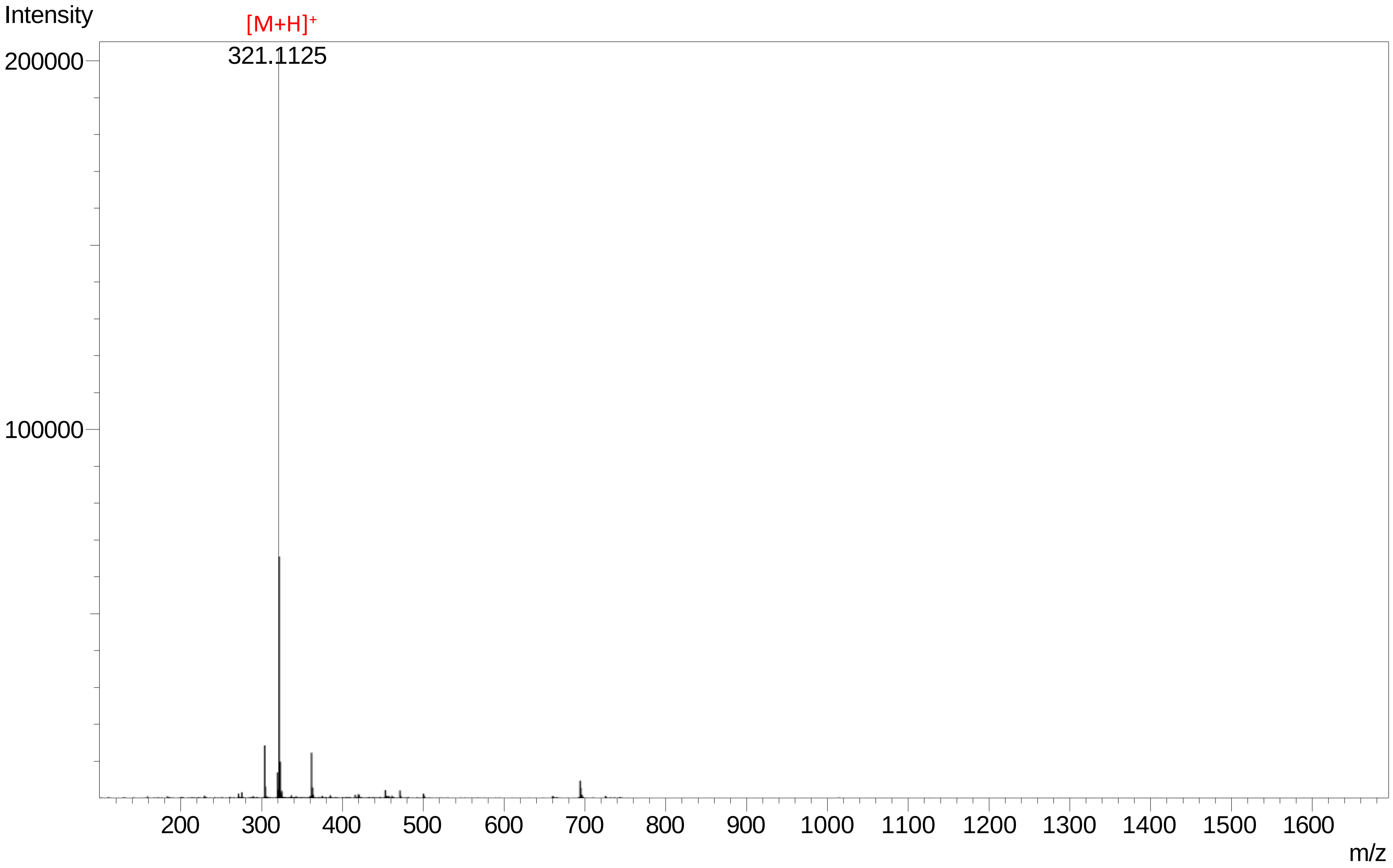

Fig. S59. (+)-HRESIMS spectrum of **22**

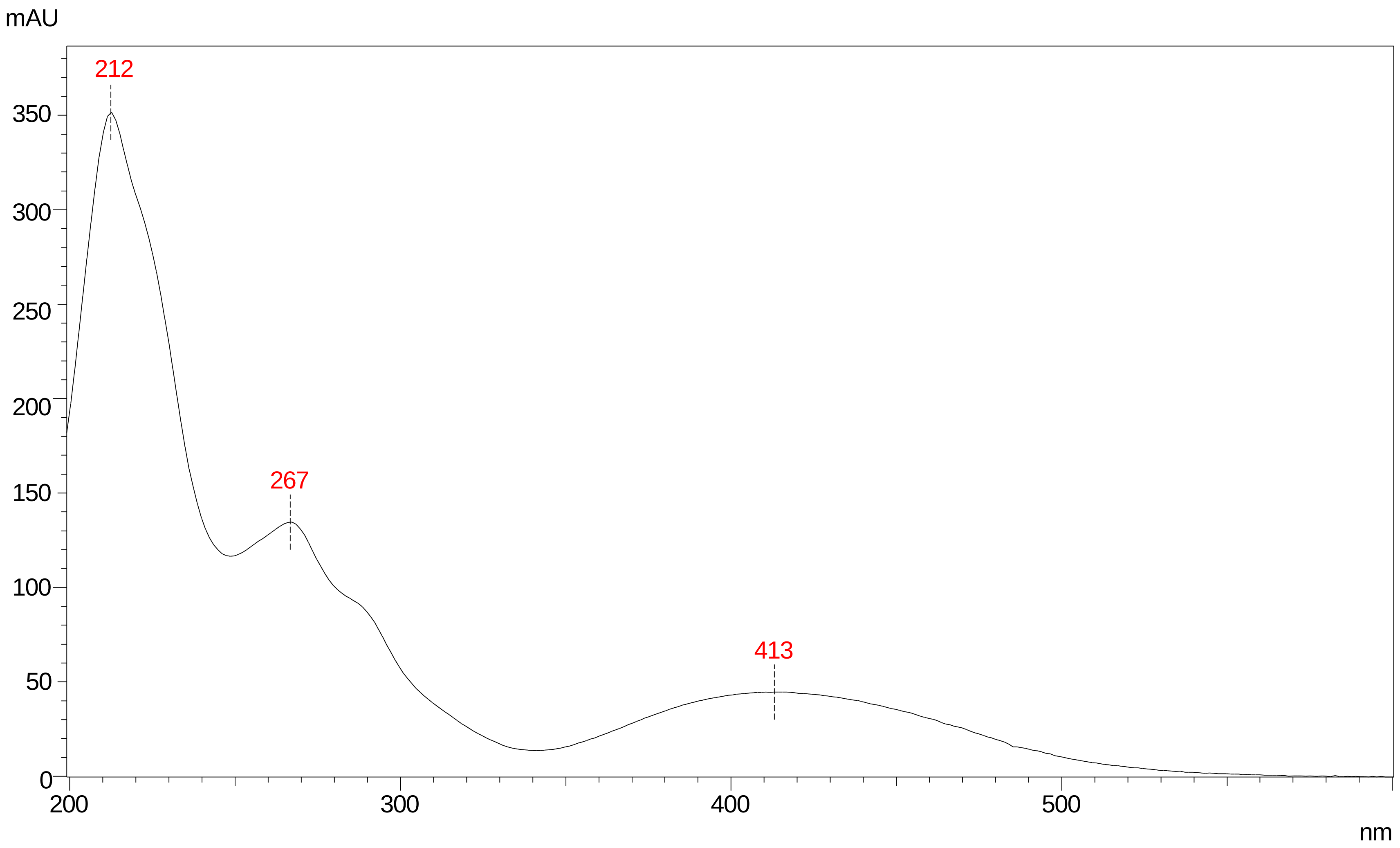

Fig. S60. UV spectrum of **22**

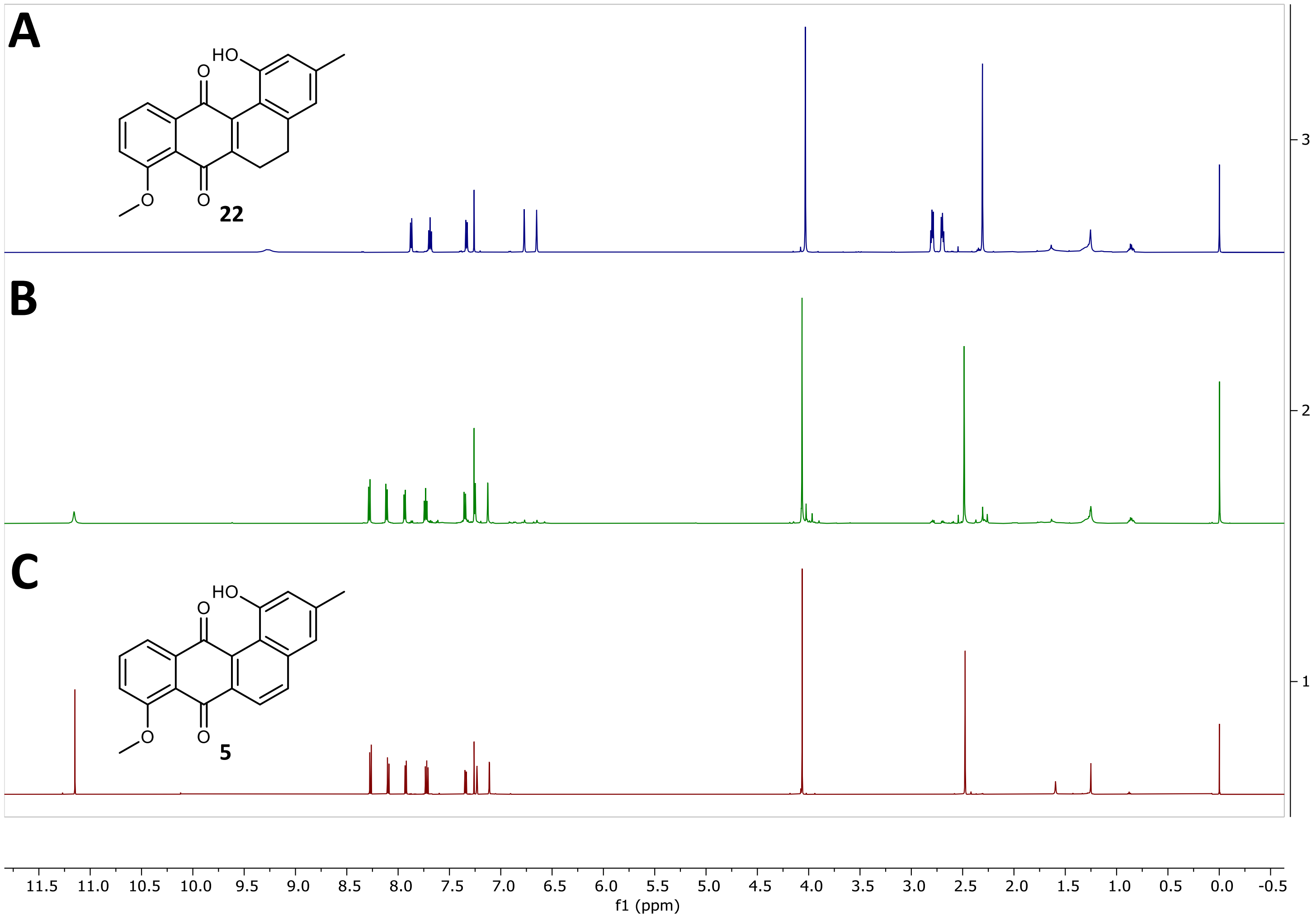

Fig. S61. (**A**) ^{1}H NMR spectrum of **22** directly after purification. (**B**) ^{1}H NMR spectrum of **22** when re-measured for 2D NMR spectra acquisition. (**C**) ^{1}H NMR spectrum of 8-*O*-methyltetrangulol (**5**) (all spectra were measured in $CDCl_3$ with added TMS at 600 MHz)

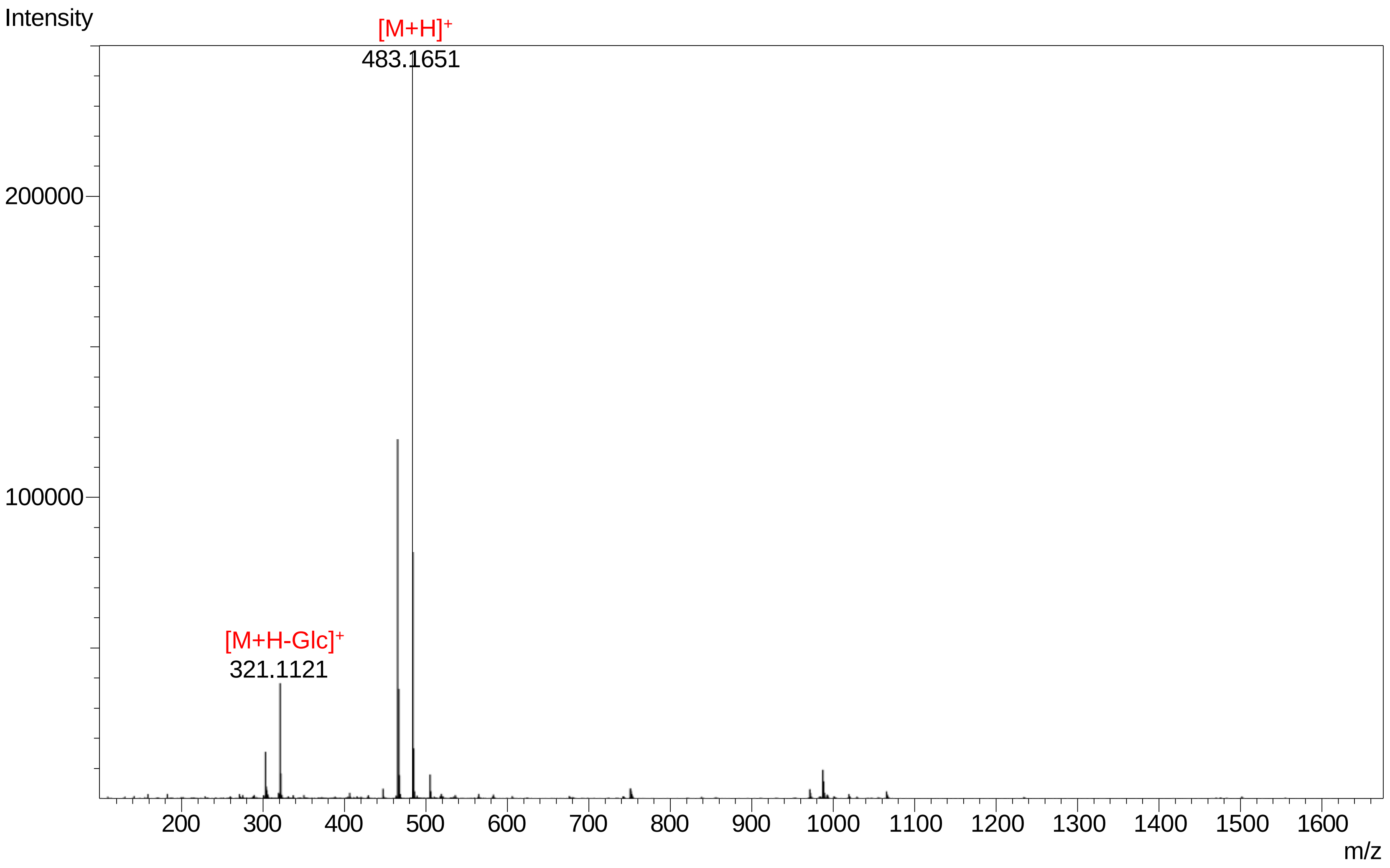

Fig. S62. (+)-HRESIMS spectrum of **23**

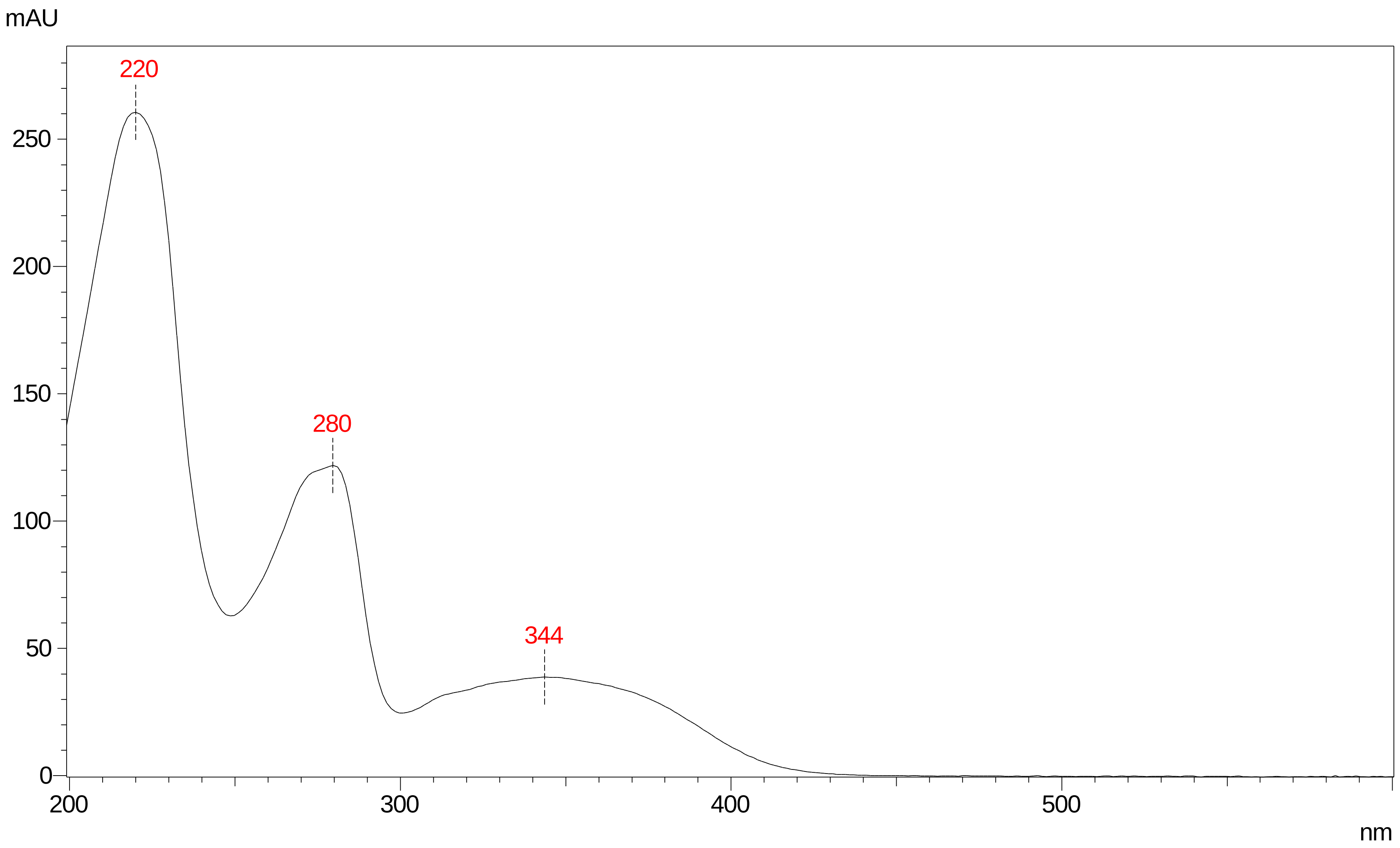

Fig. S63. UV spectrum of **23**

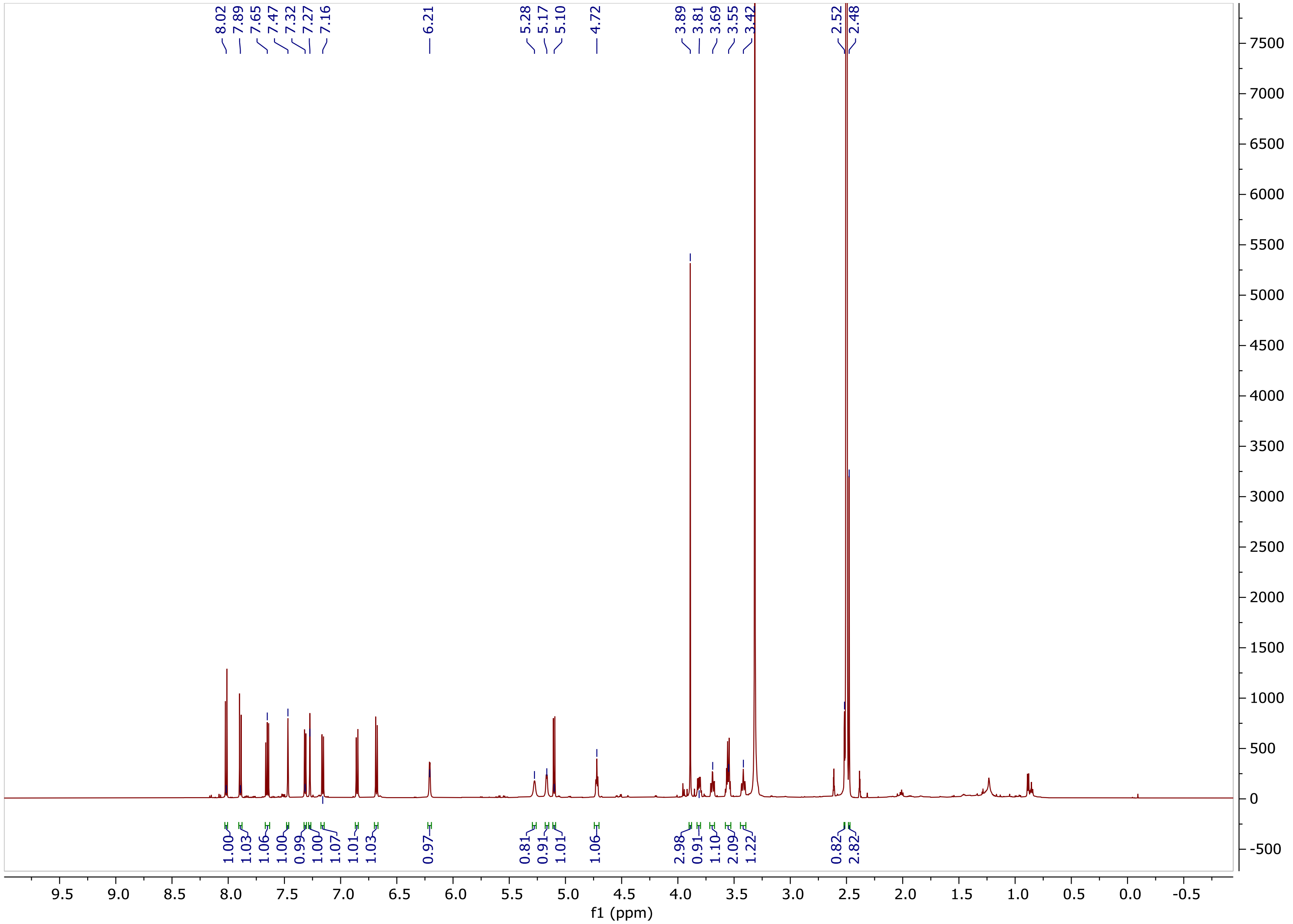

Fig. S64. ^{1}H NMR spectrum of **23** directly after purification (600 MHz, in DMSO-*d6*)

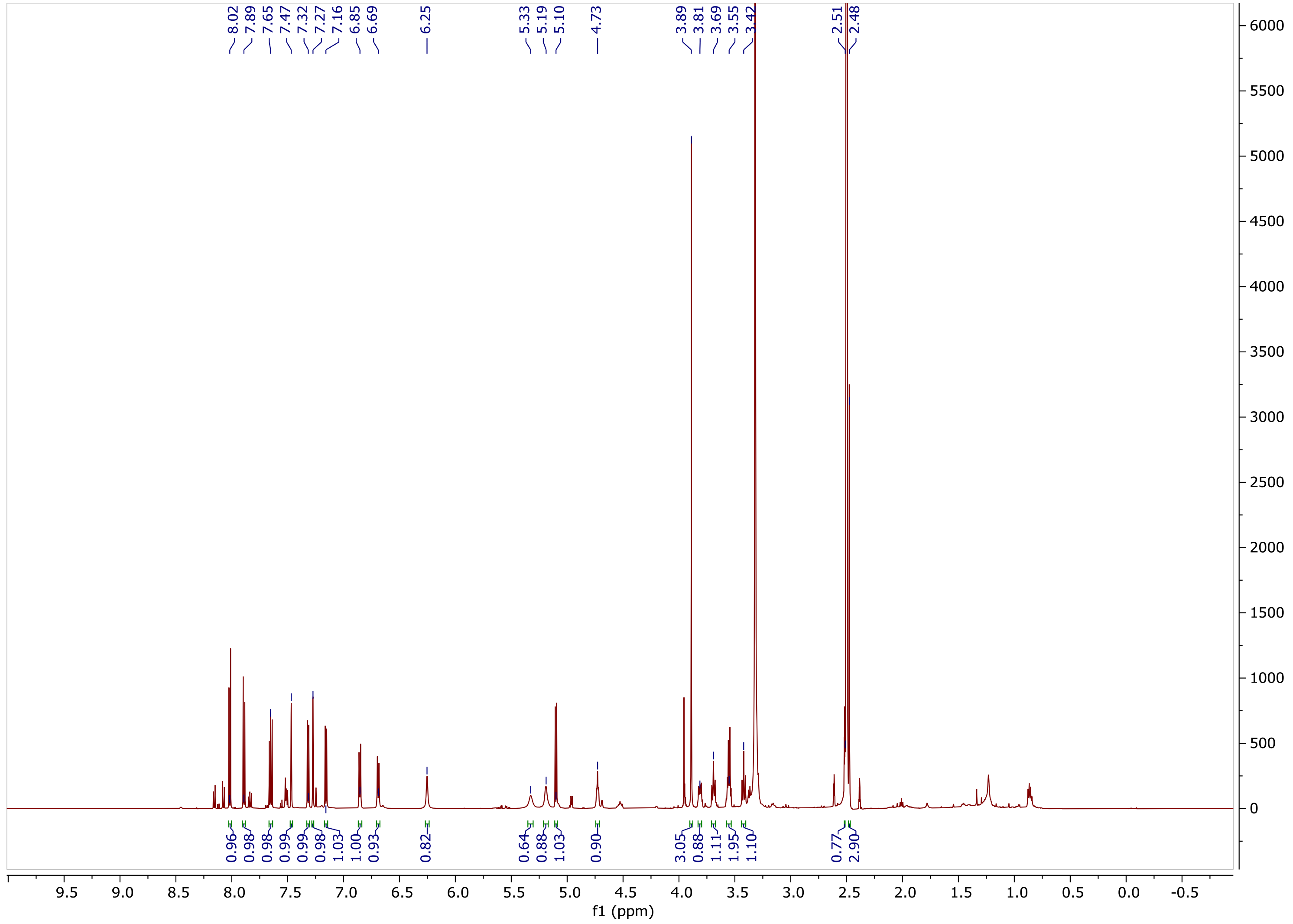

Fig. S65. ^{1}H NMR spectrum of **23** when re-measured for 2D NMR spectra acquisition (600 MHz, in DMSO-*d6*). Other signals started to appear due to the compound being unstable

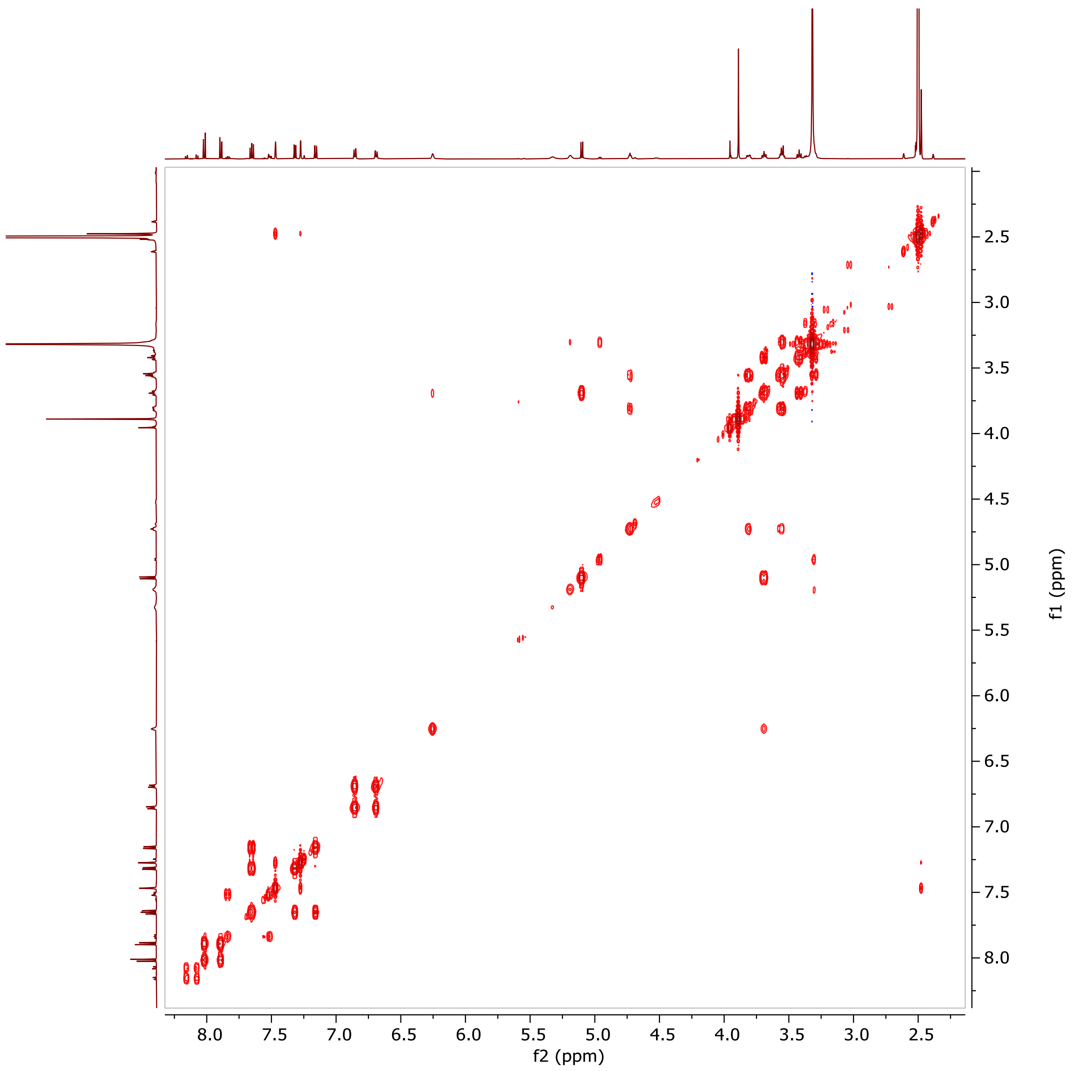

Fig. S66. COSY spectrum of **23** (600 MHz, in DMSO-*d6*)

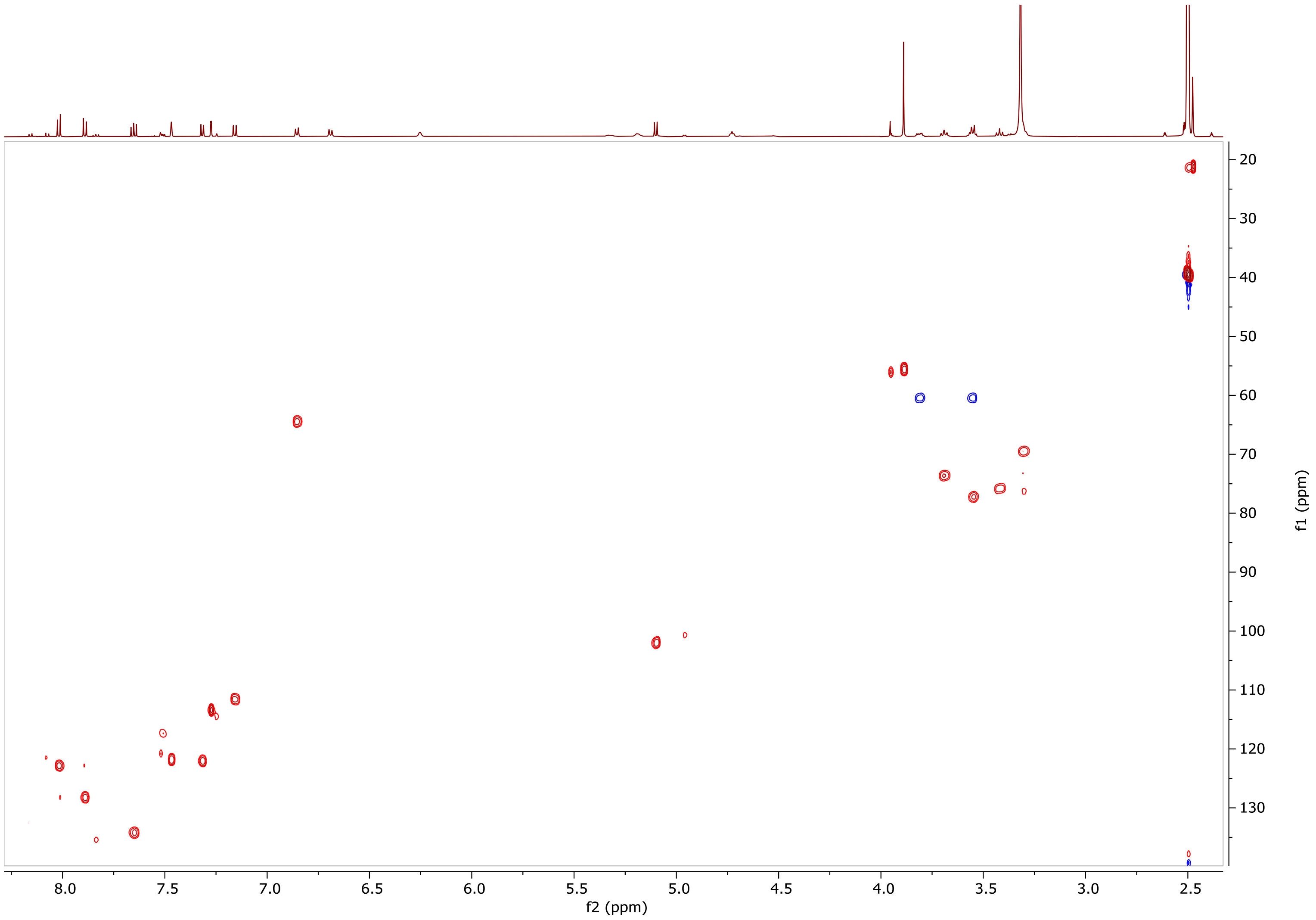

Fig. S67. Multiplicity-edited HSQC spectrum of **23** (600 MHz, in DMSO-*d6*)

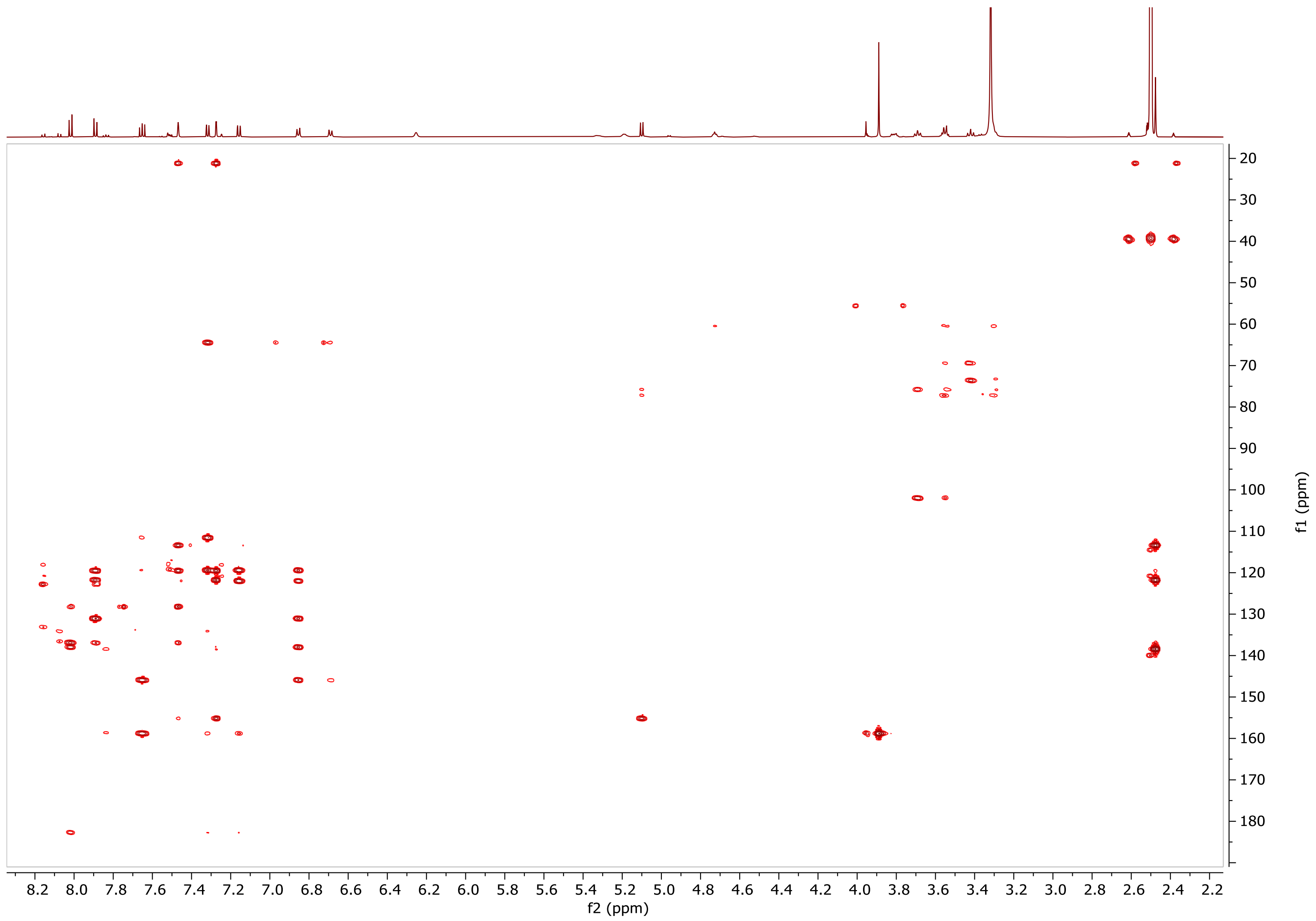

Fig. S68. HMBC spectrum of **23** (600 MHz, in DMSO-*d6*)

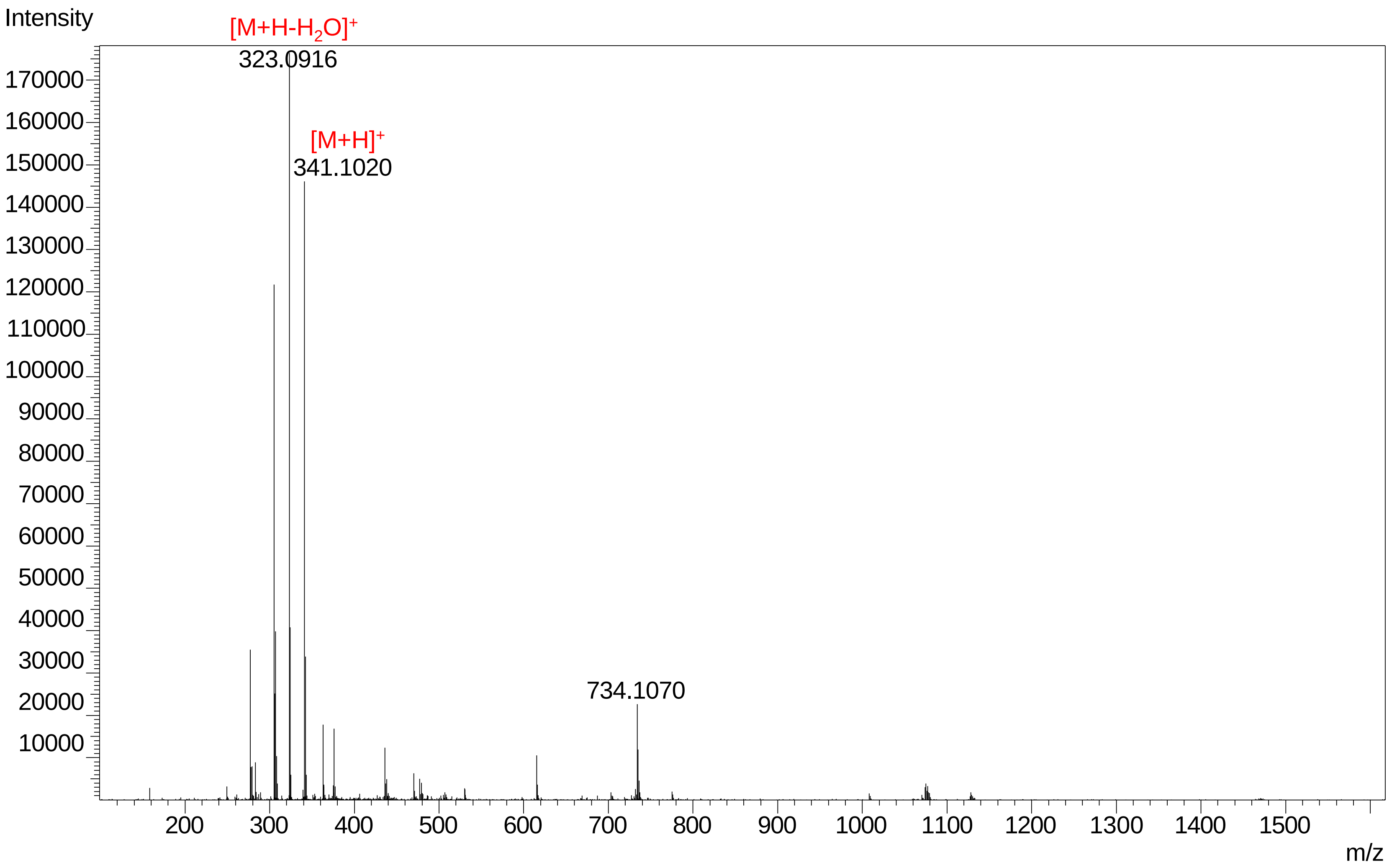

Fig. S69. (+)-HRESIMS spectrum of **24**

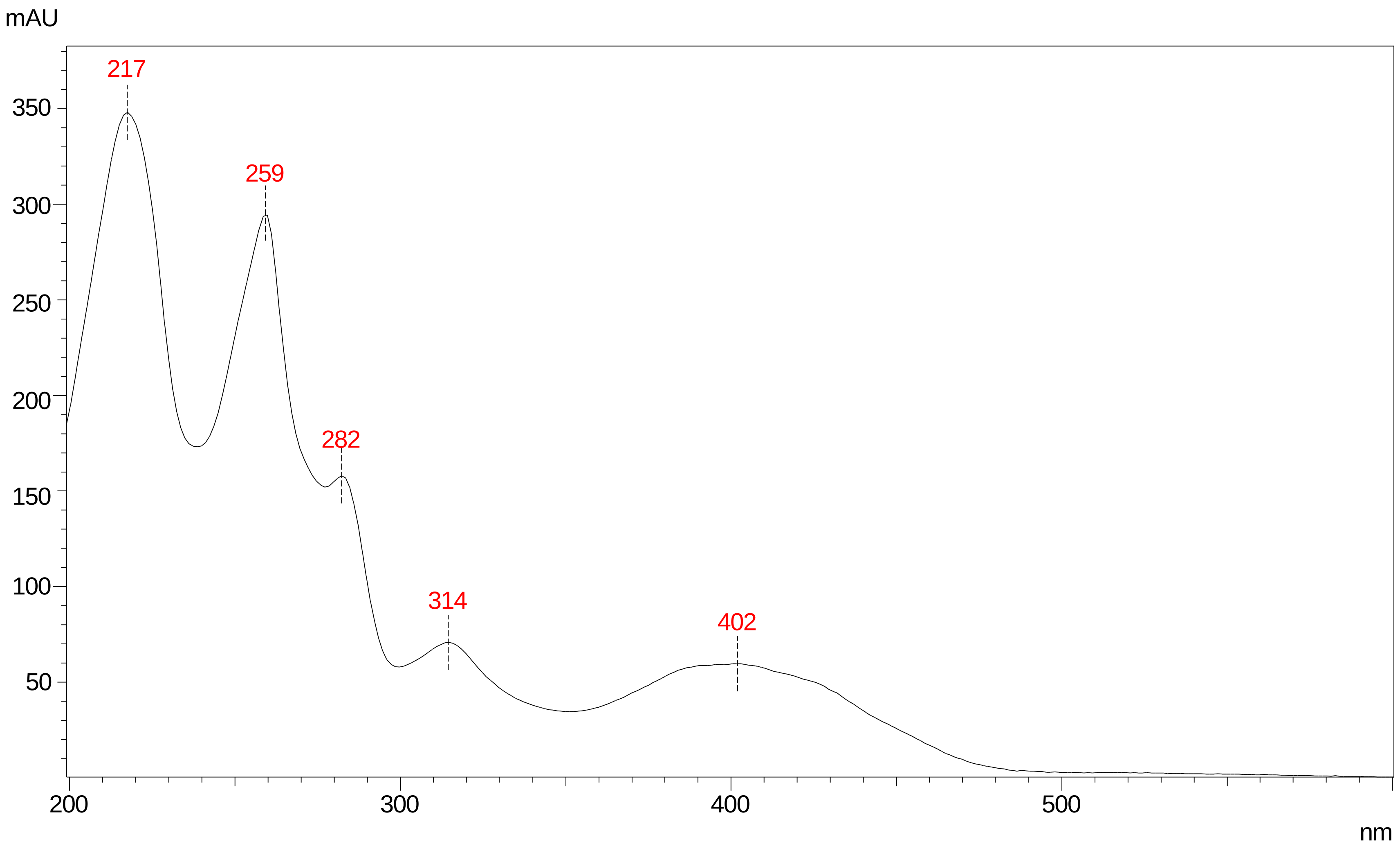

Fig. S70. UV spectrum of **24**

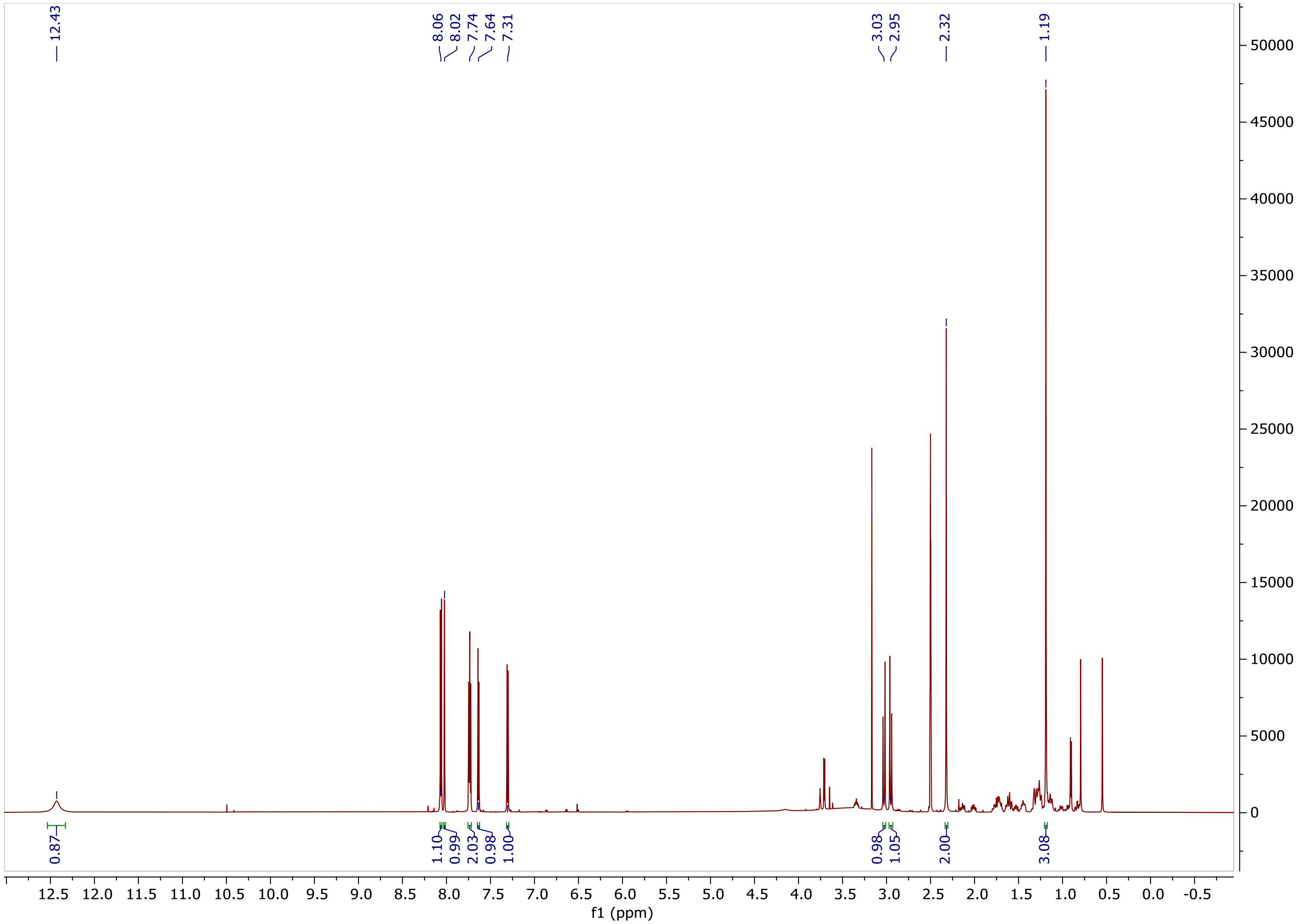

Fig. S71. ^{1}H NMR spectrum of **24** (600 MHz, in DMSO-*d6*)

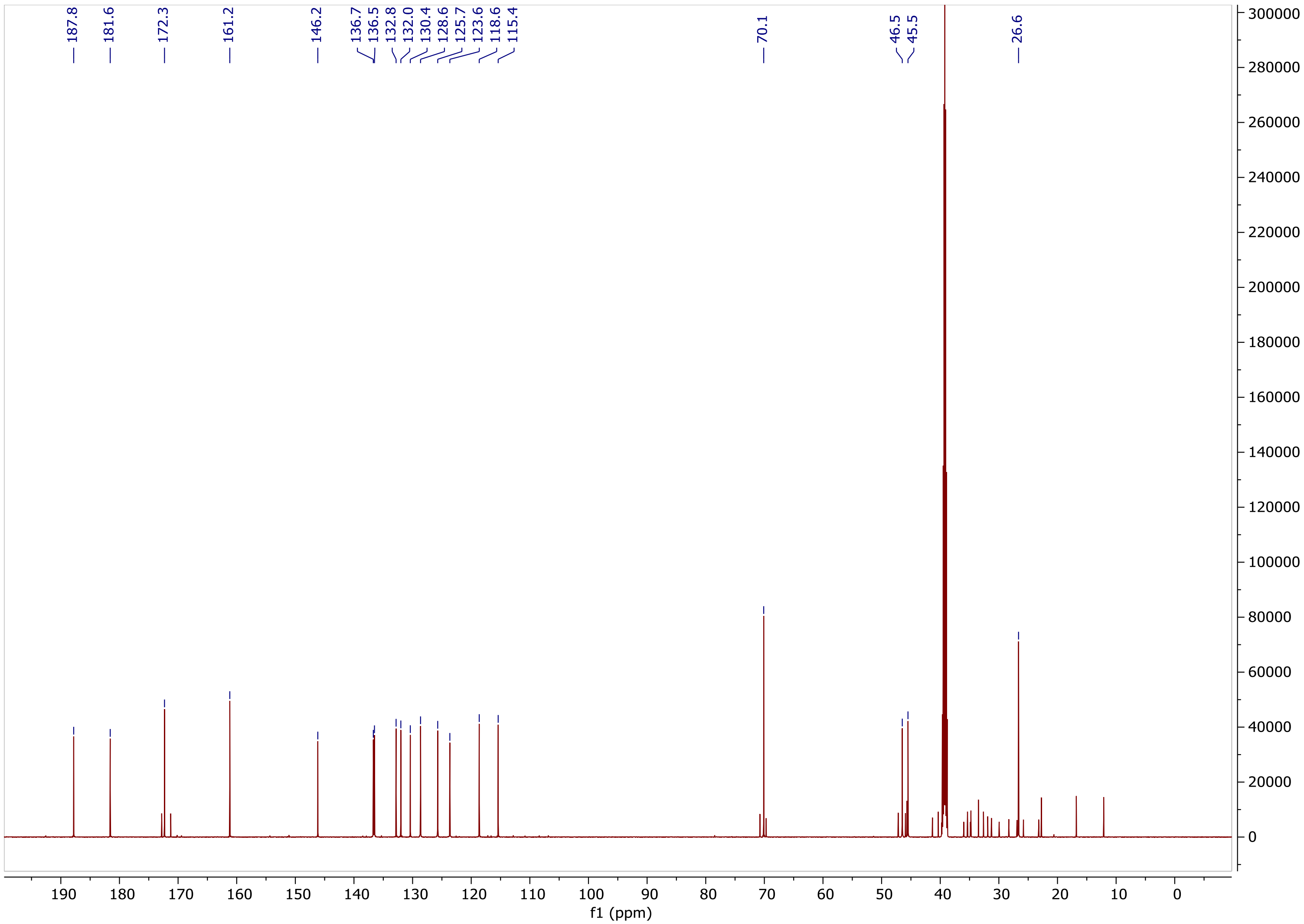

Fig. S72. ^{13}C NMR spectrum of **24** (150 MHz, in DMSO-*d6*)

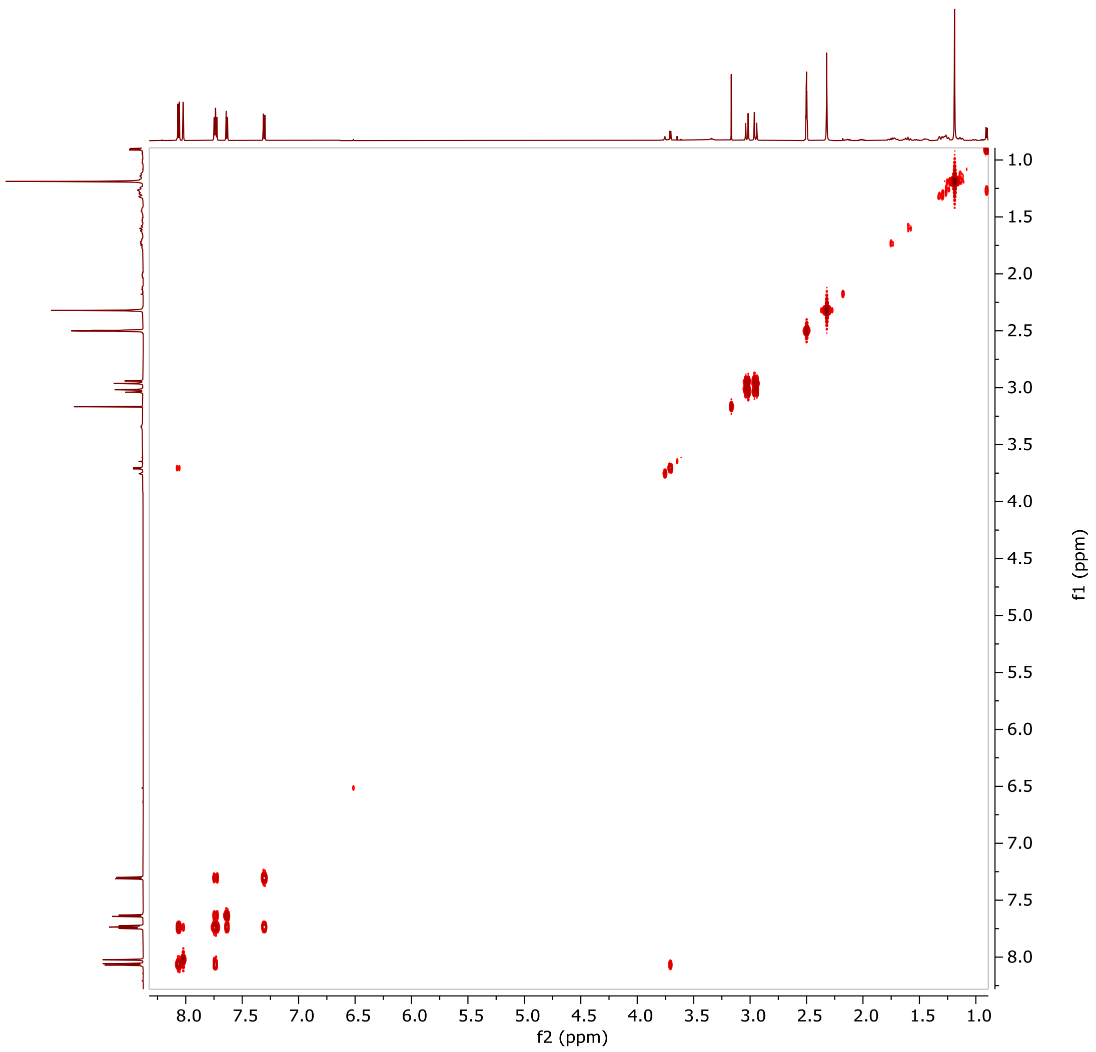

Fig. S73. COSY spectrum of **24** (600 MHz, in DMSO-*d6*)

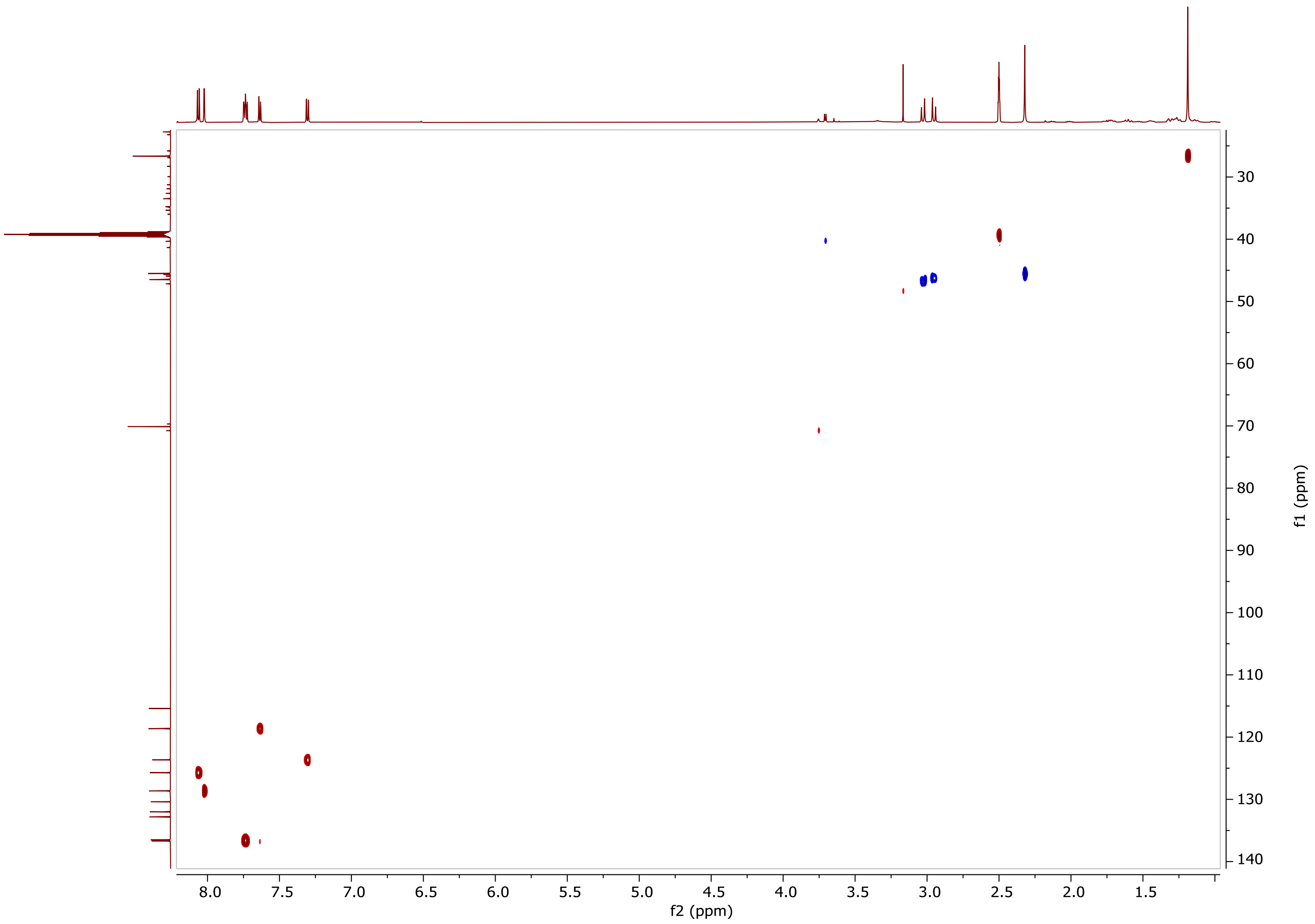

Fig. S74. Multiplicity-edited HSQC spectrum of **24** (600 MHz, in DMSO-*d6*)

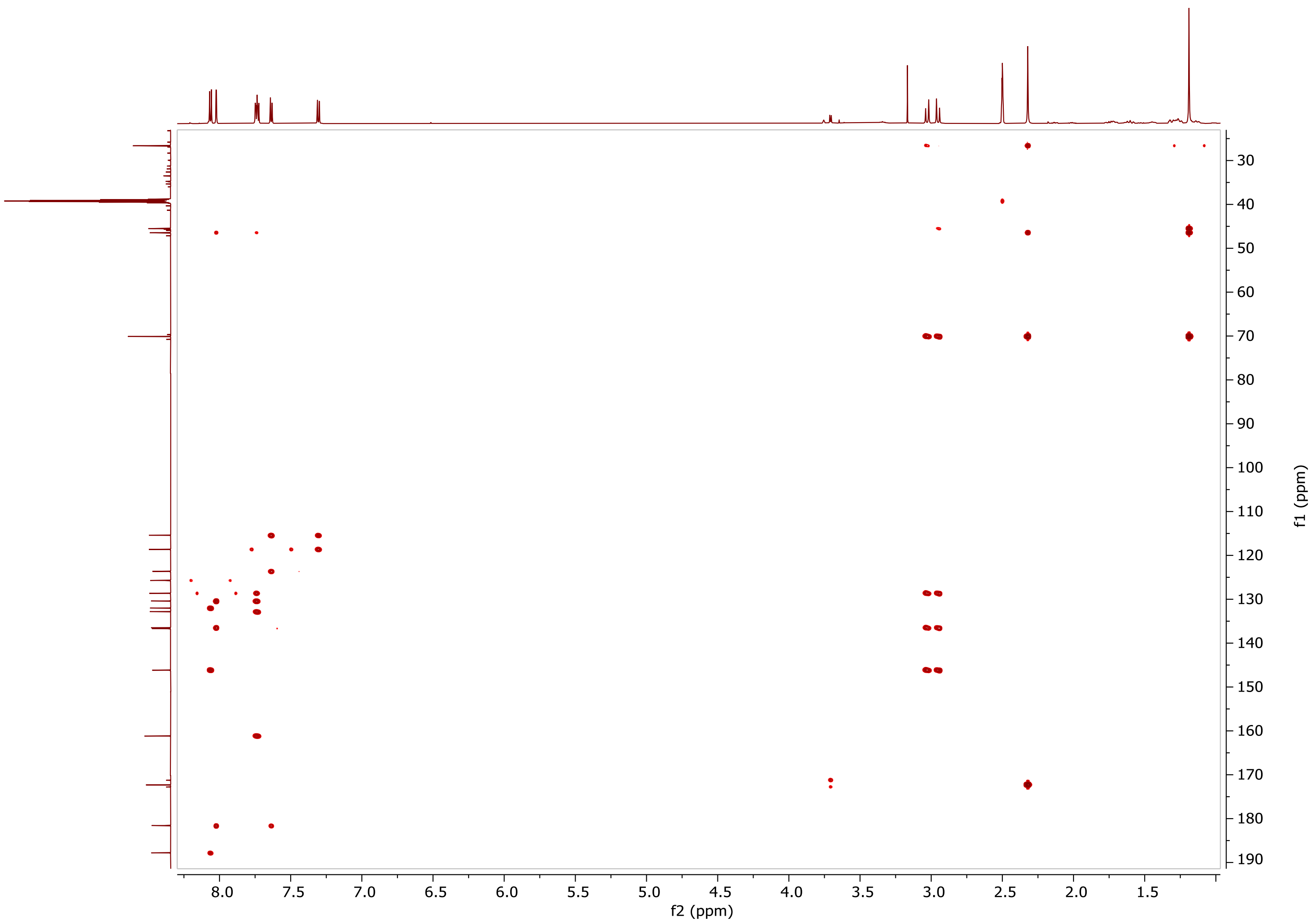

Fig. S75. HMBC spectrum of **24** (600 MHz, in DMSO-*d6*)

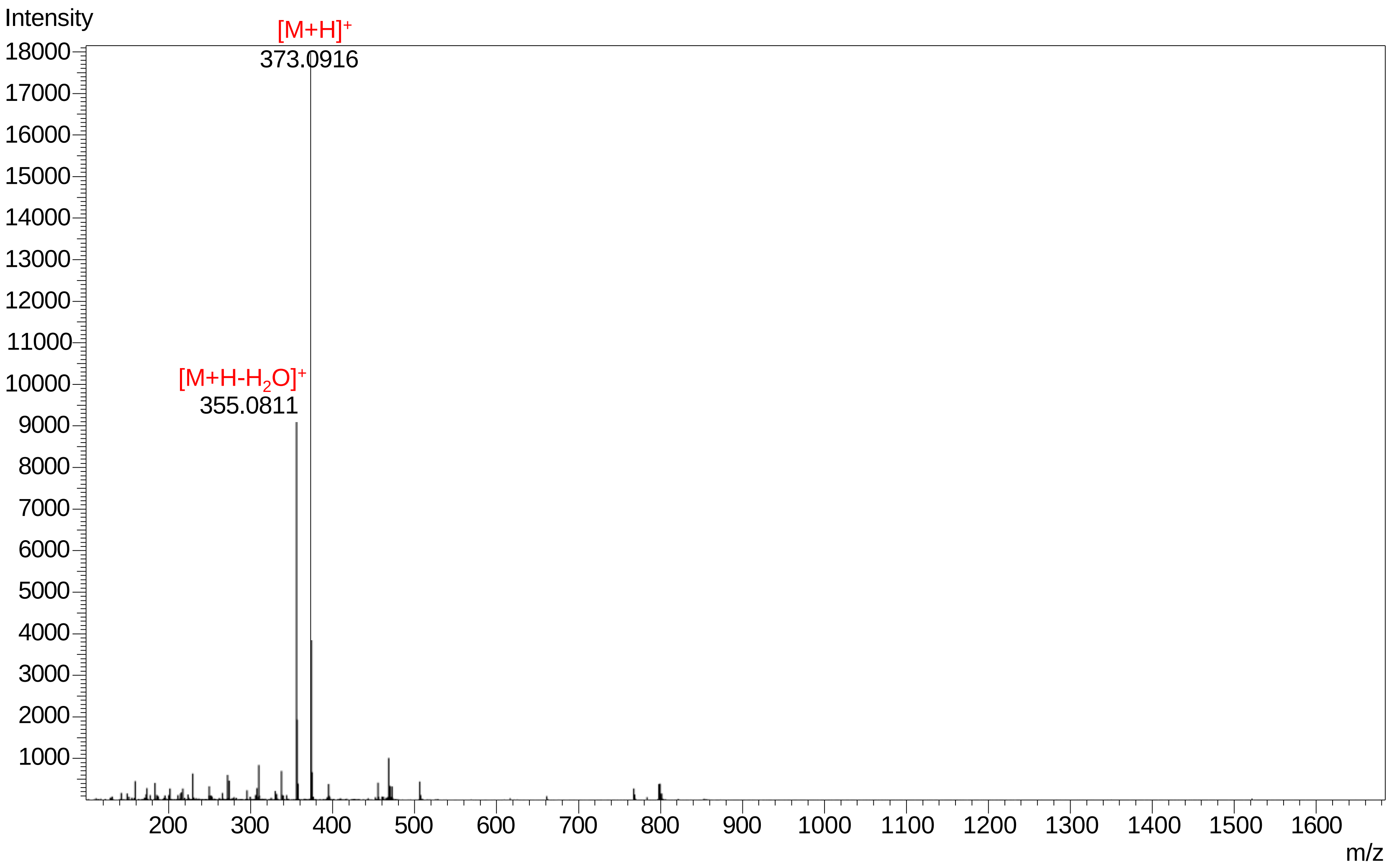

Fig. S76. (+)-HRESIMS spectrum of **25**

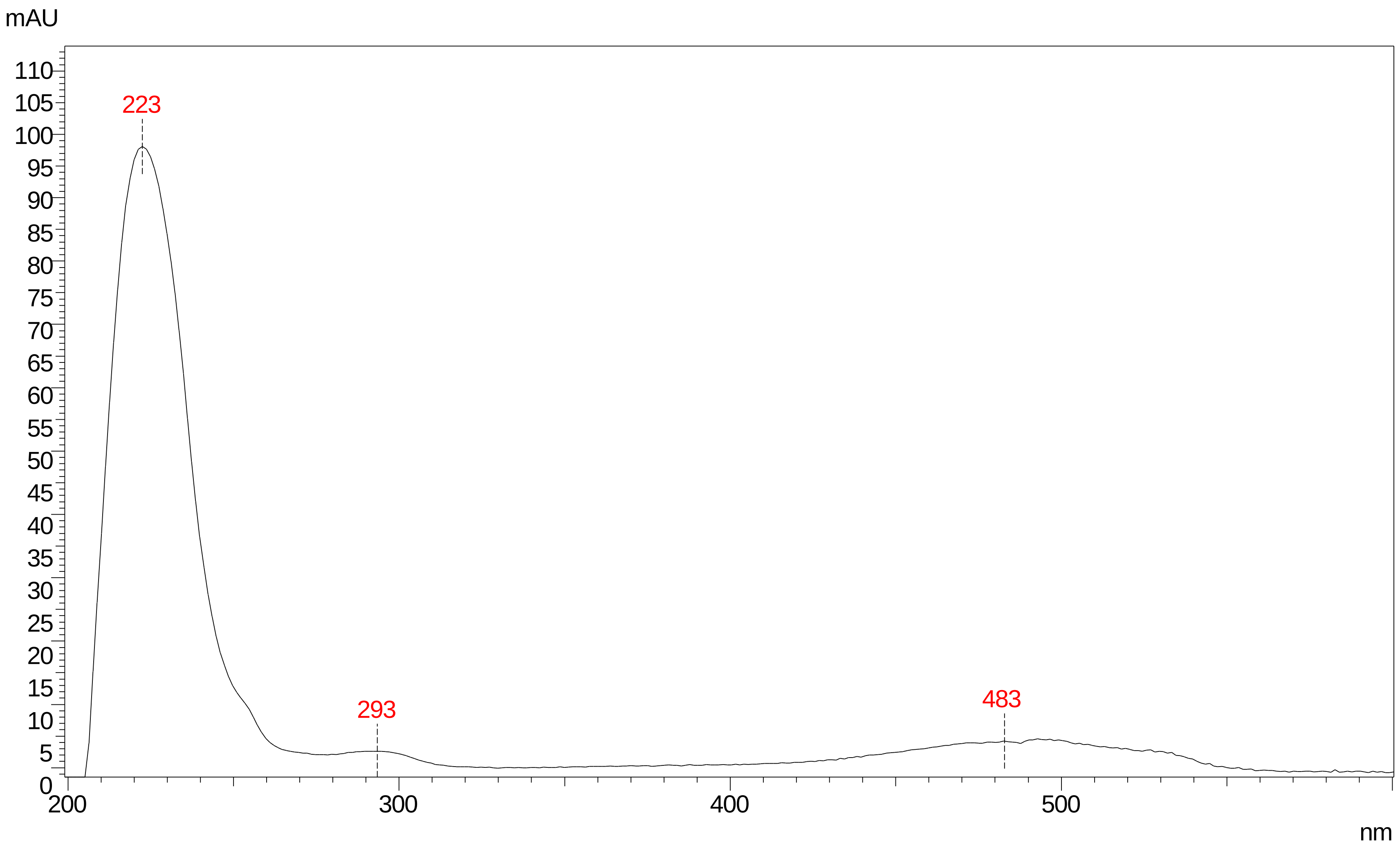

Fig. S77. UV spectrum of **25**

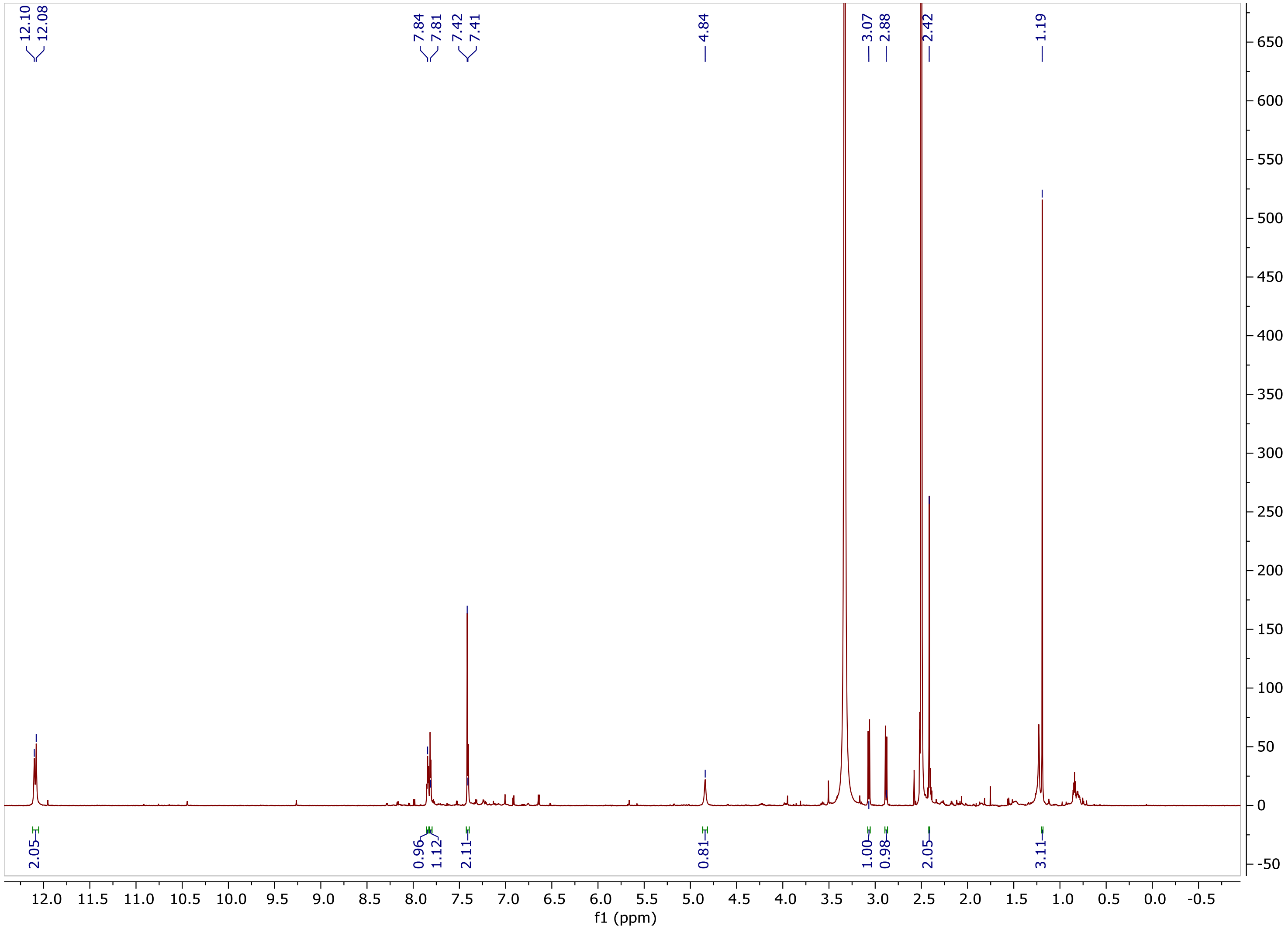

Fig. S78. ^{1}H NMR spectrum of **25** (850 MHz, in DMSO-*d6*)

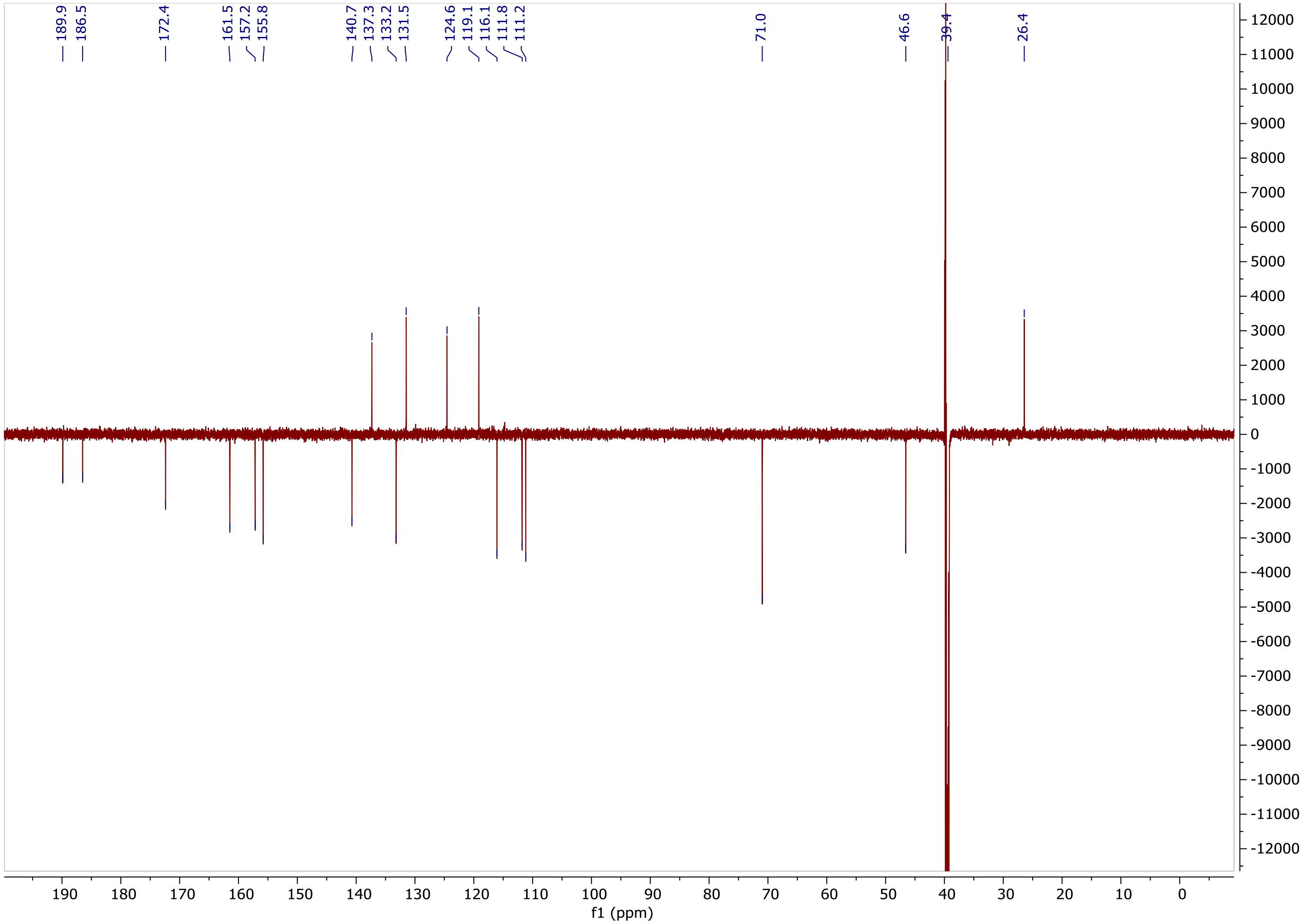

Fig. S79. ^{13}C APT spectrum of **25** (213 MHz, in DMSO-*d6*)

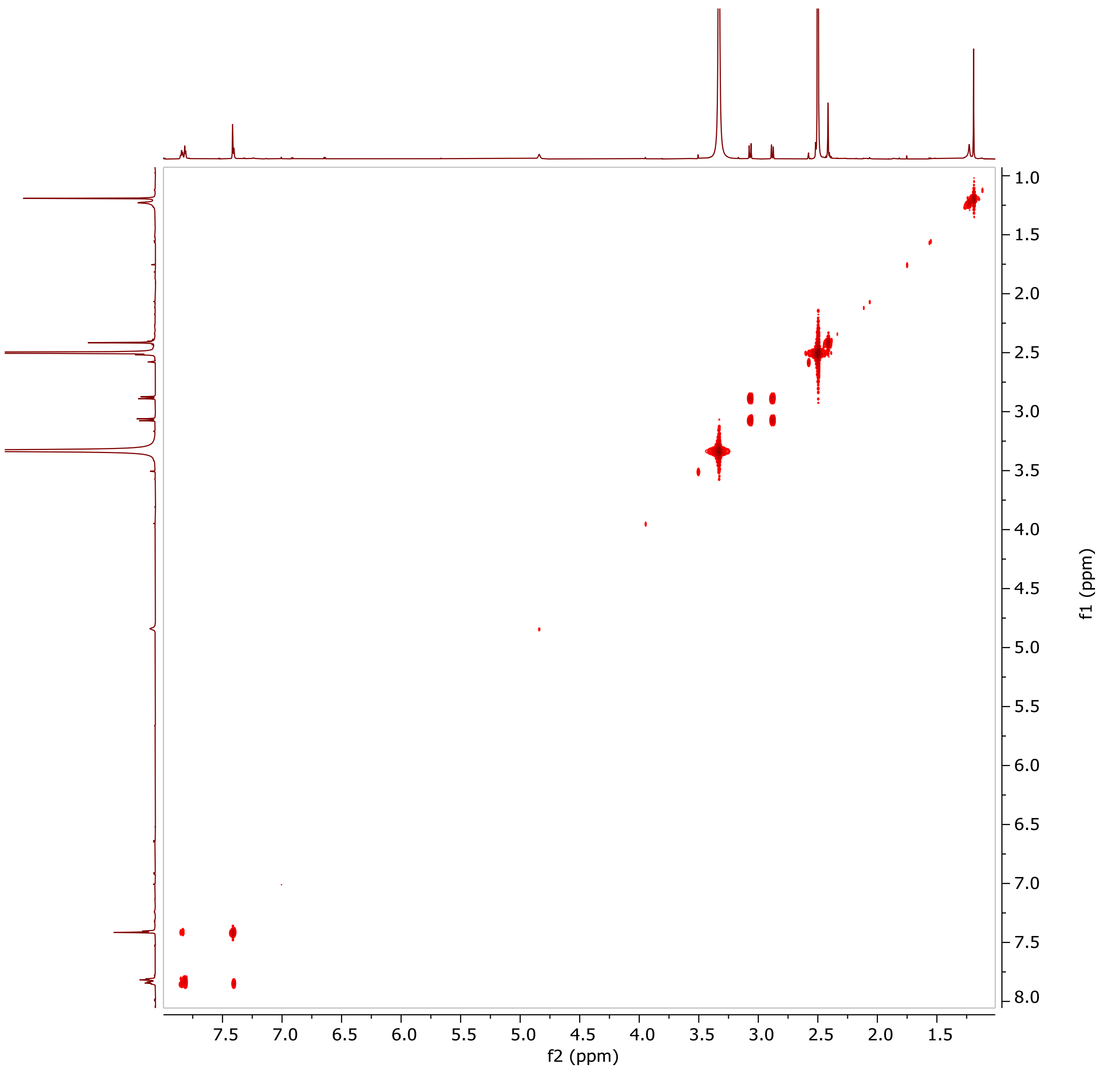

Fig. S80. COSY spectrum of **25** (850 MHz, in DMSO-*d6*)

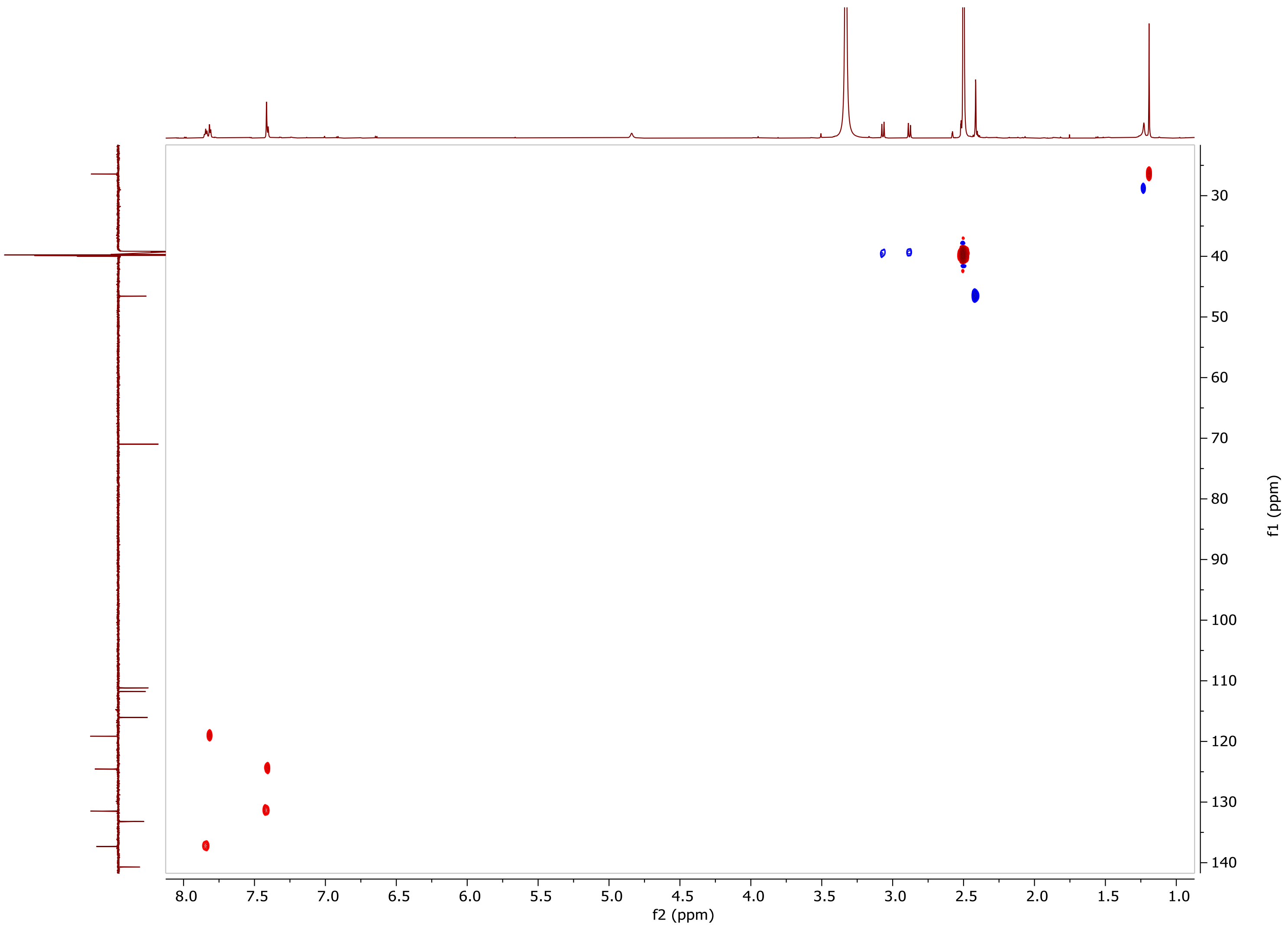

Fig. S81. Multiplicity-edited HSQC spectrum of **25** (850 MHz, in DMSO-*d6*)

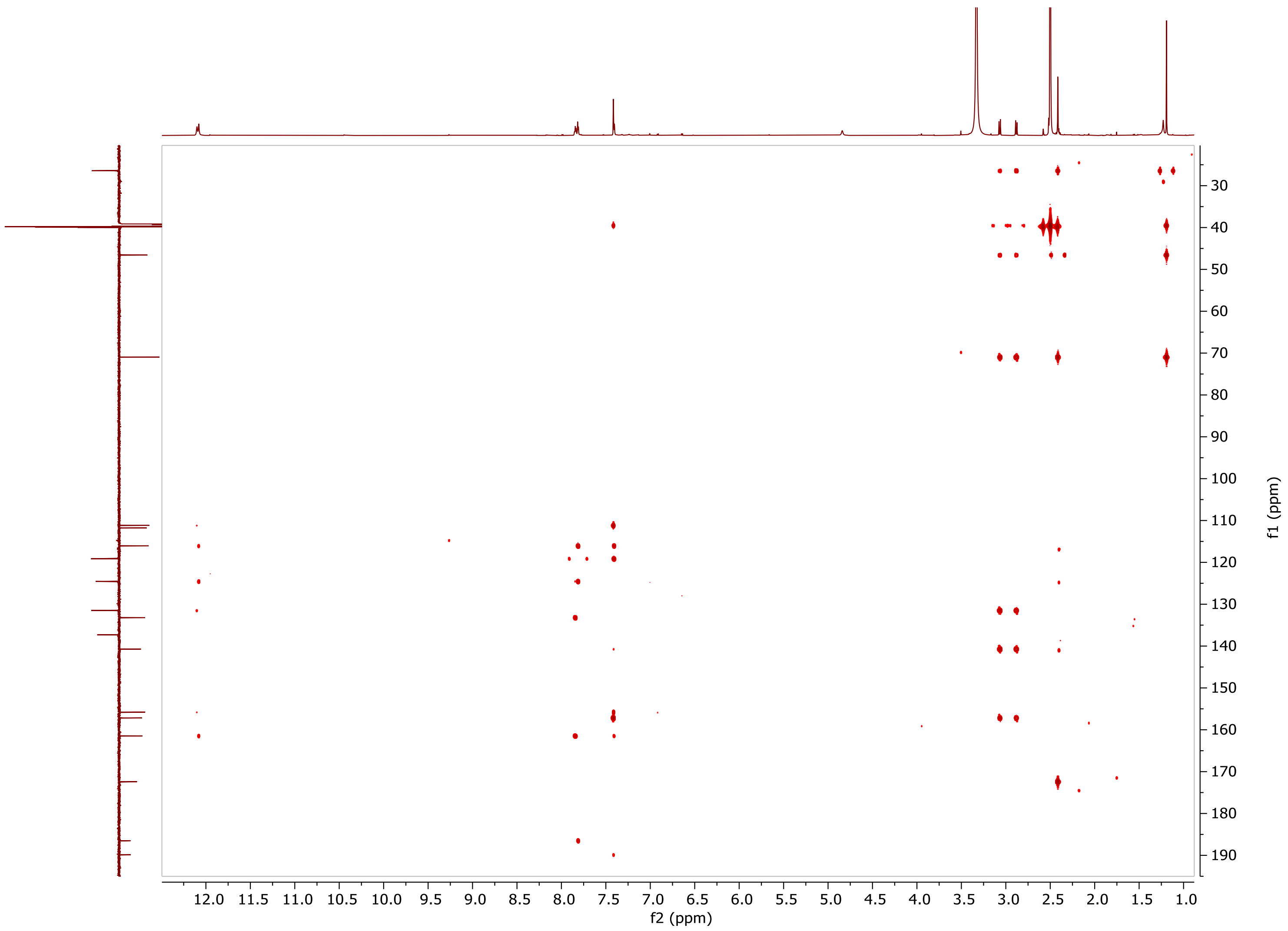

Fig. S82. HMBC spectrum of **25** (850 MHz, in DMSO-*d6*)

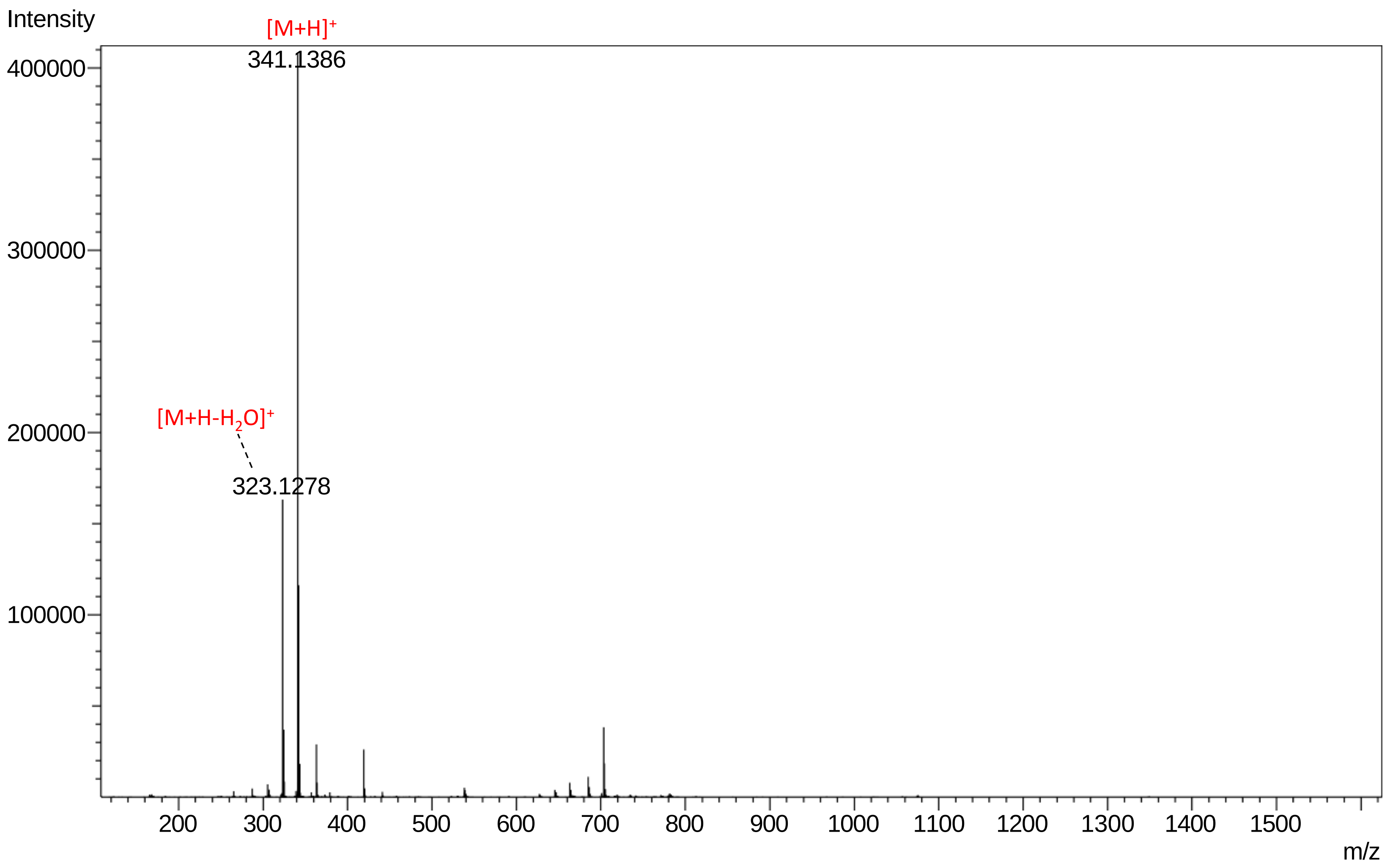

Fig. S83. (+)-HRESIMS spectrum of **26**

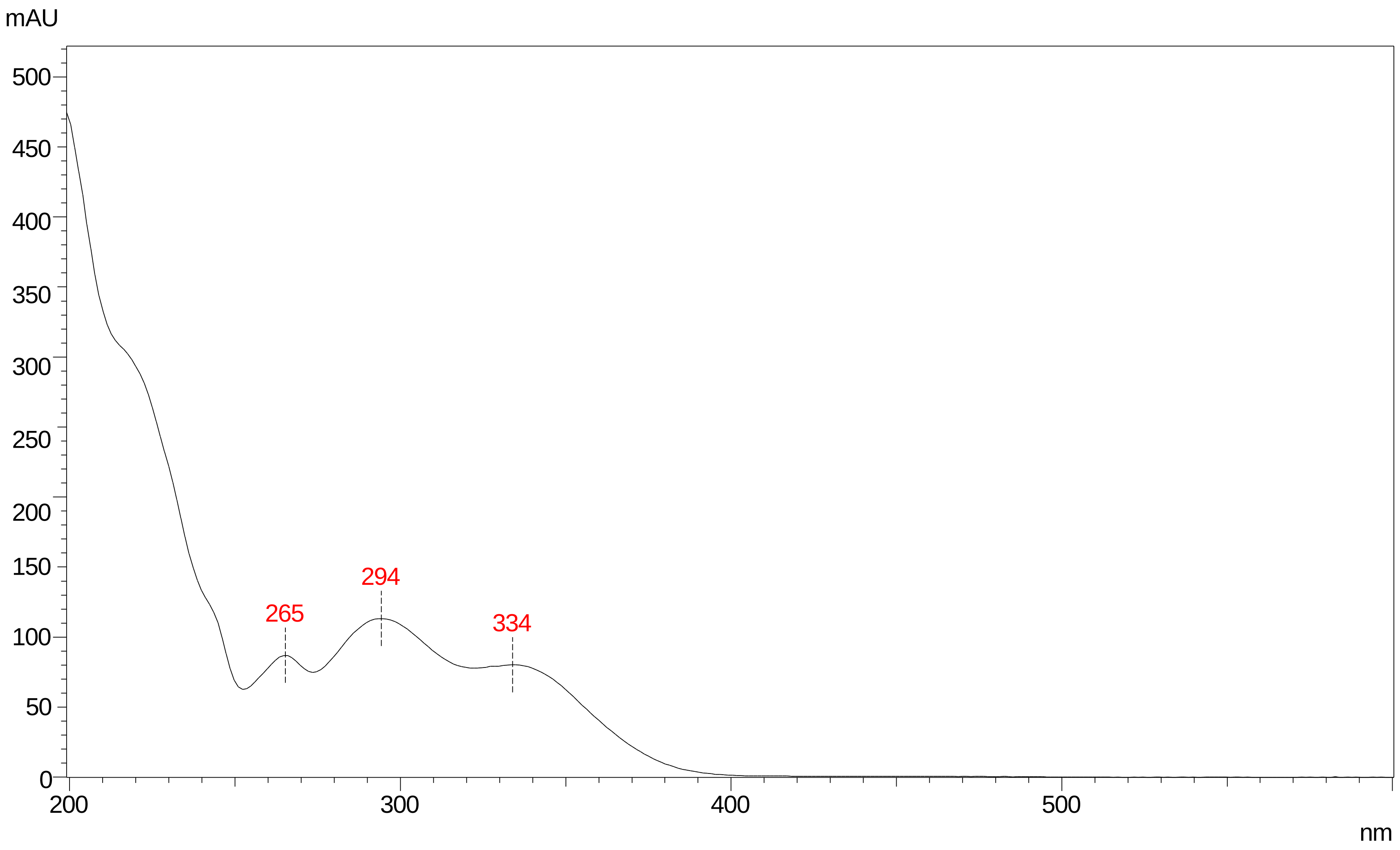

Fig. S84. UV spectrum of **26**

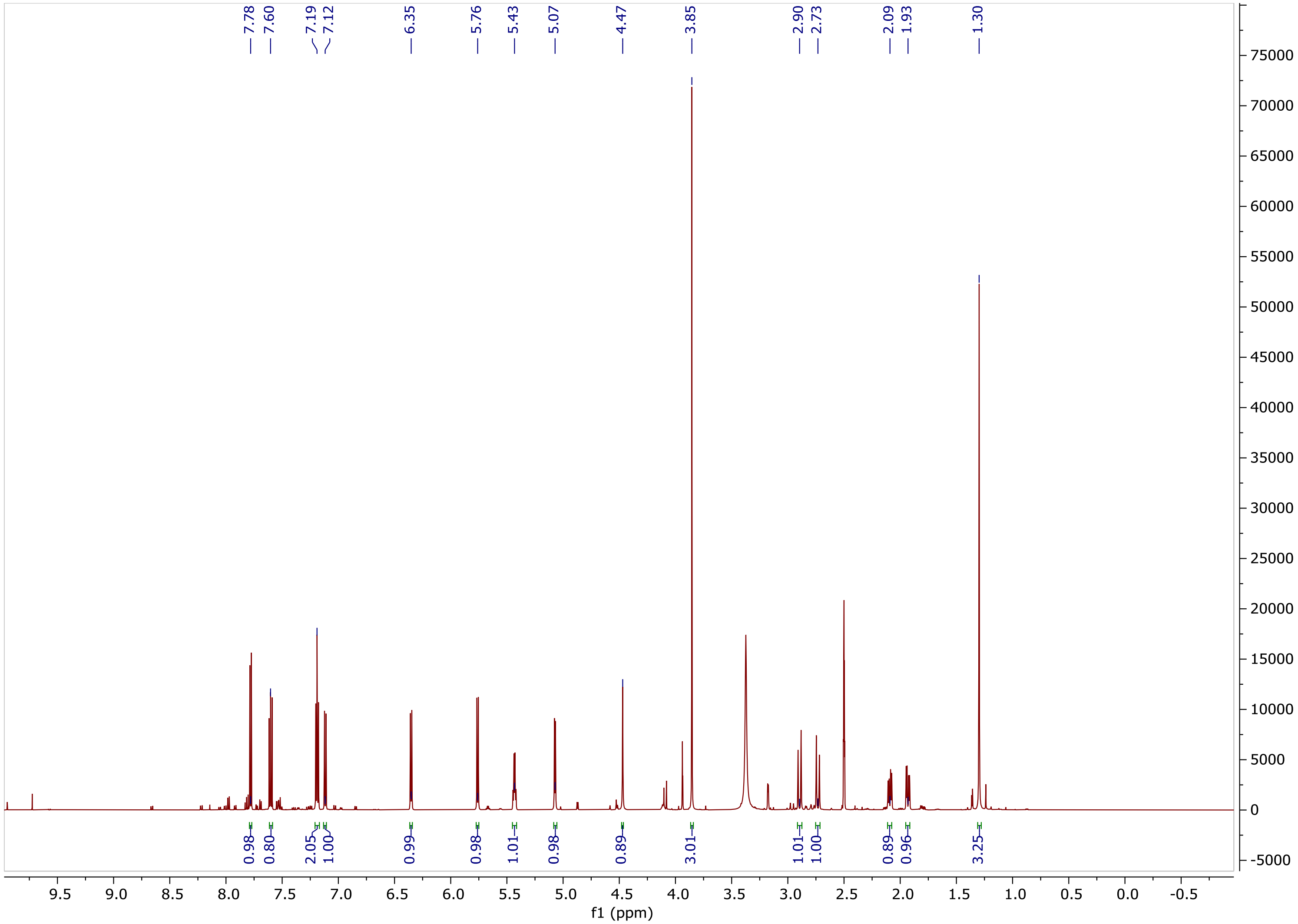

Fig. S85. ¹H NMR spectrum of **26** directly after purification (600 MHz, in DMSO-*d6*)

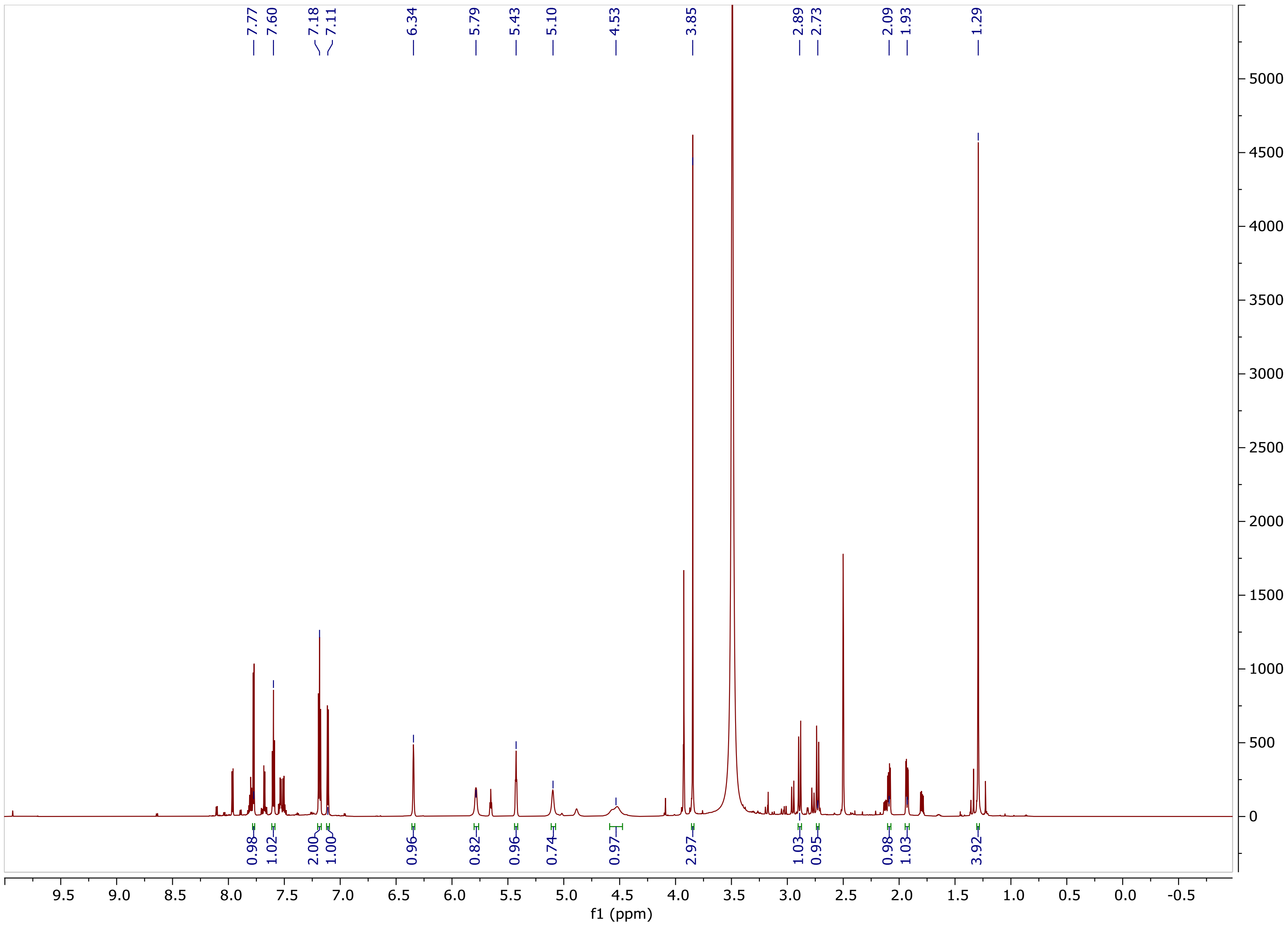

Fig. S86. ^{1}H NMR spectrum of **26** when re-measured for ^{13}C NMR spectrum acquisition (850 MHz, in DMSO-*d6*). Other signals started to appear due to the compound being unstable

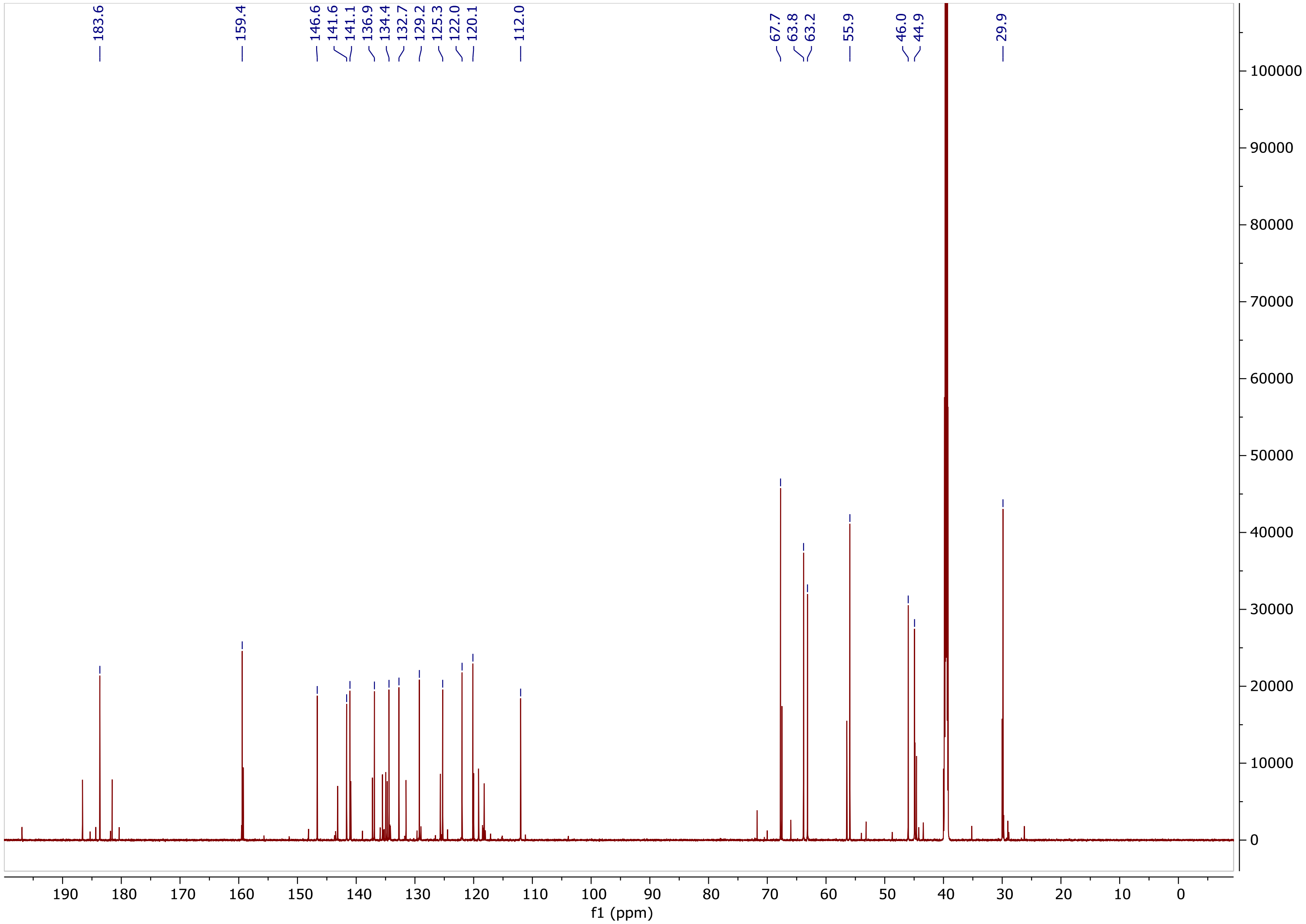

Fig. S87. ^{13}C NMR spectrum of **26** (213 MHz, in DMSO-*d6*)

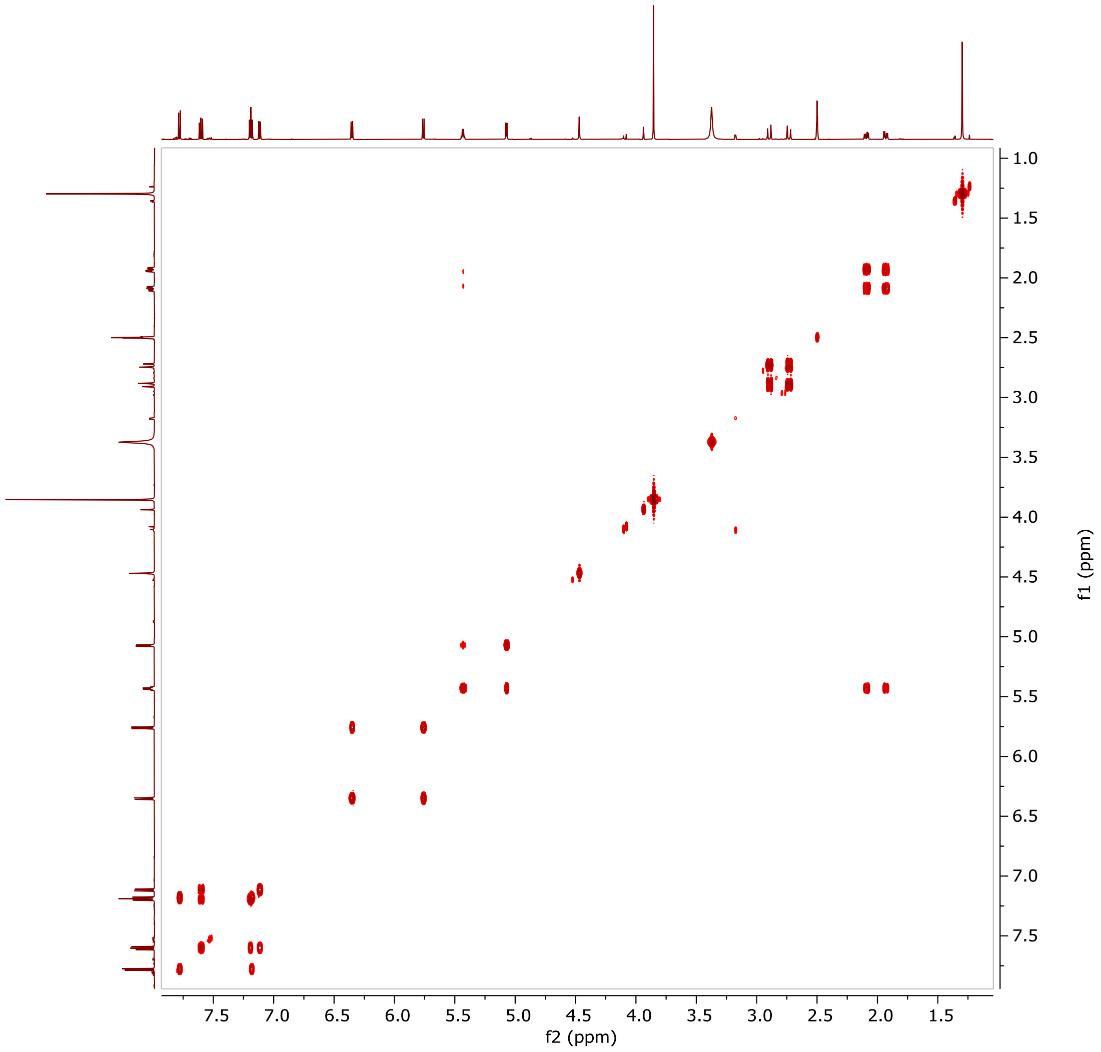

Fig. S88. COSY spectrum of **26** (600 MHz, in DMSO-*d6*)

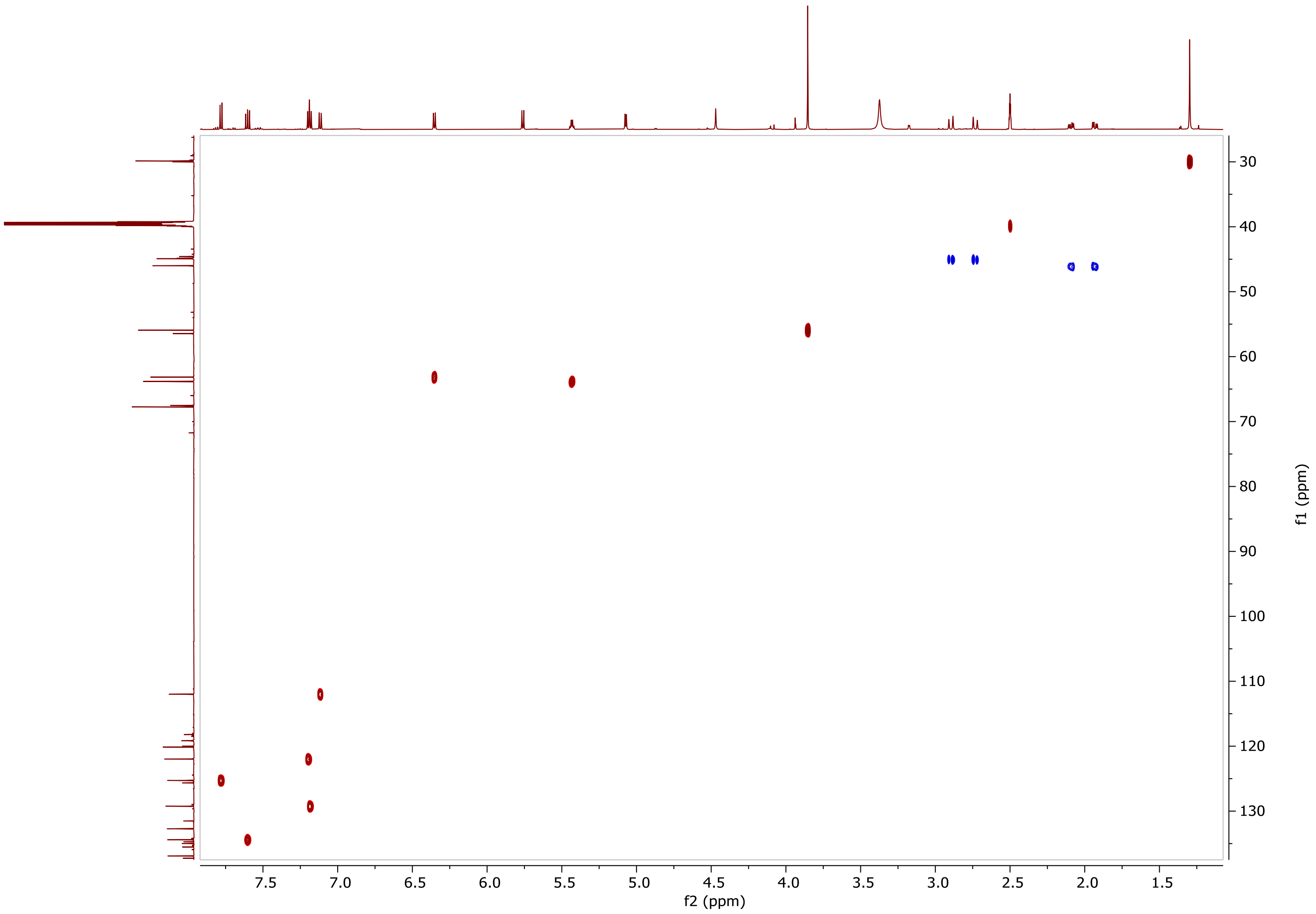

Fig. S89. Multiplicity-edited HSQC spectrum of **26** (600 MHz, in DMSO-*d6*)

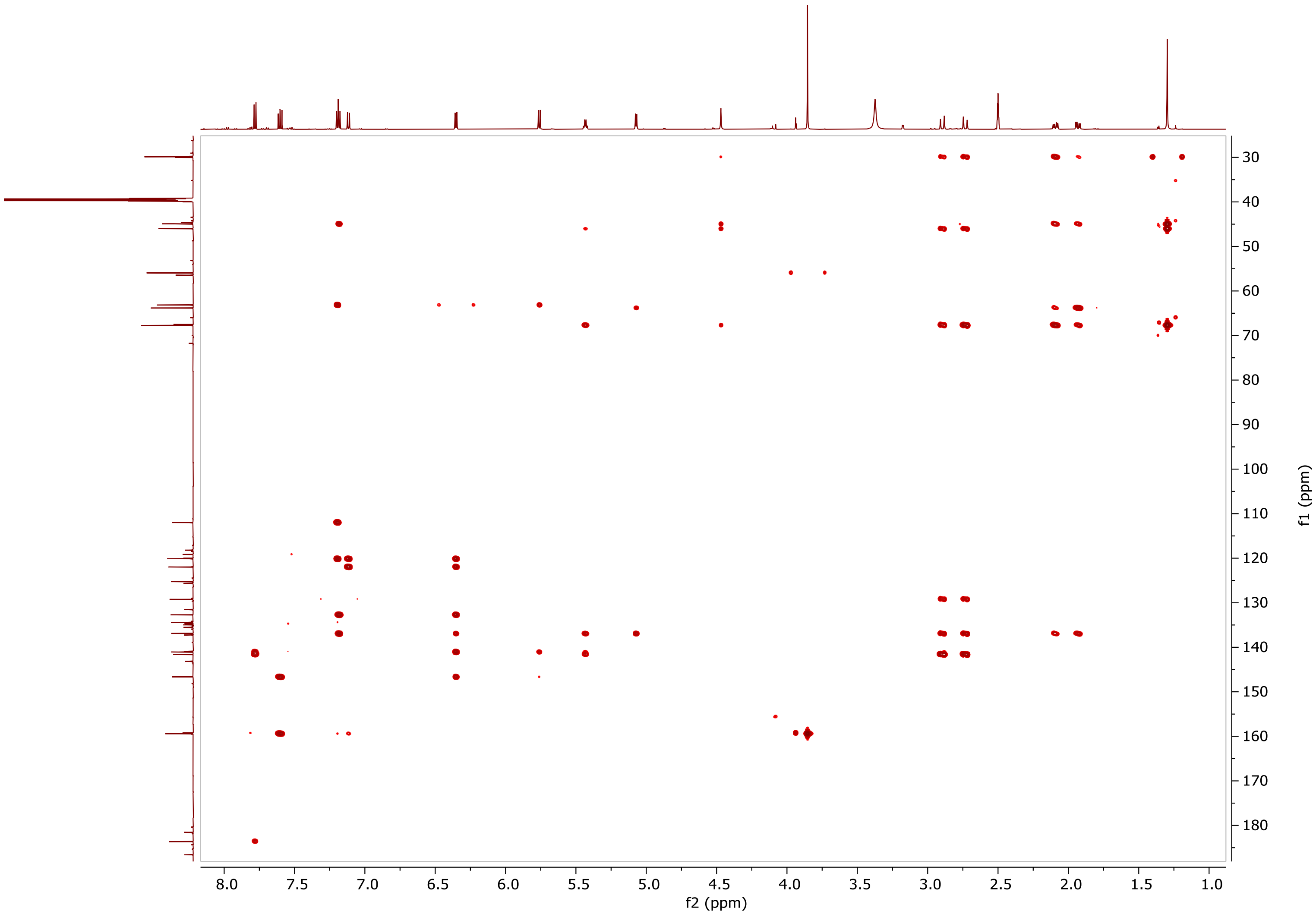

Fig. S90. HMBC spectrum of **26** (600 MHz, in DMSO-*d6*)

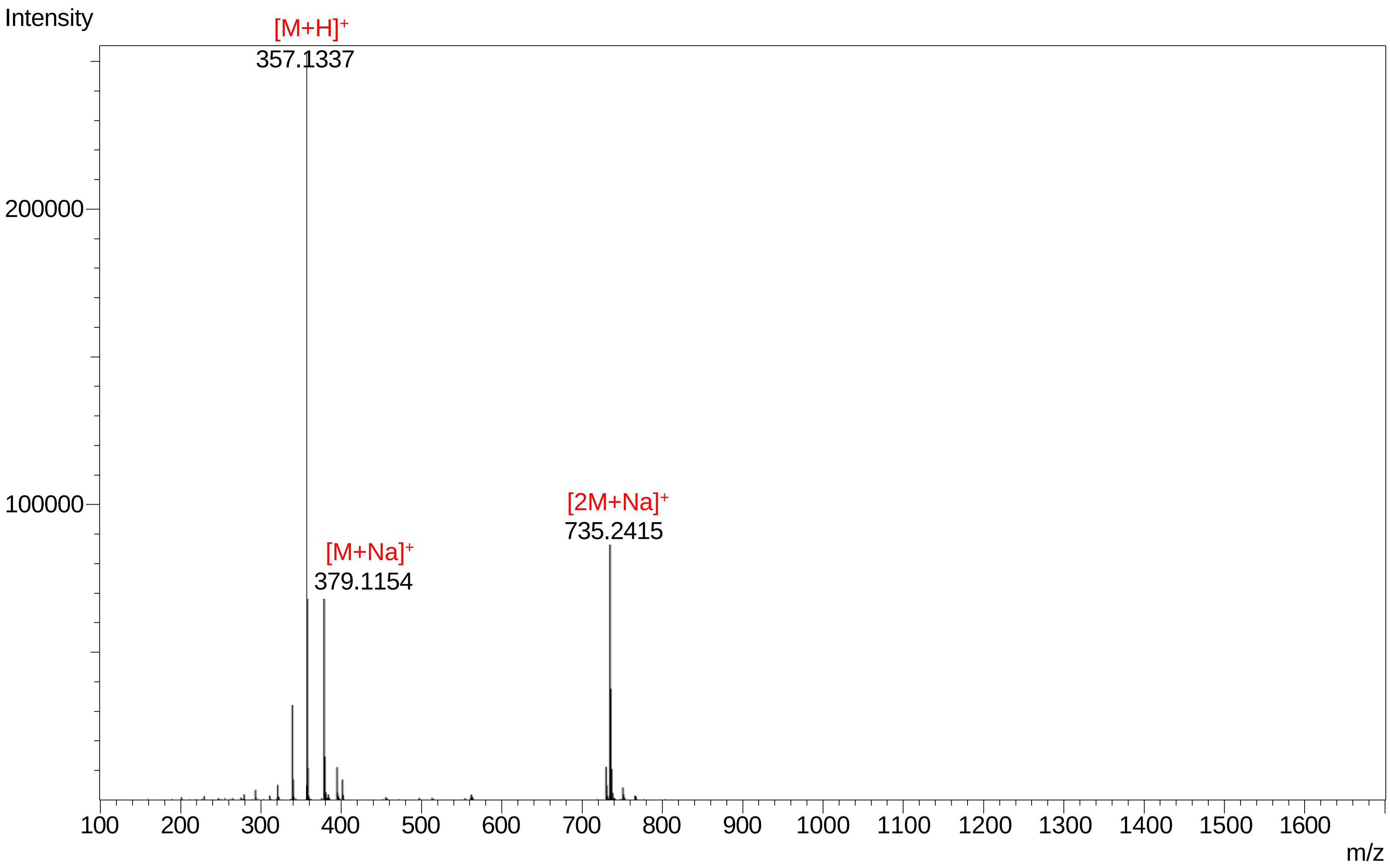

Fig. S91. (+)-HRESIMS spectrum of **27**

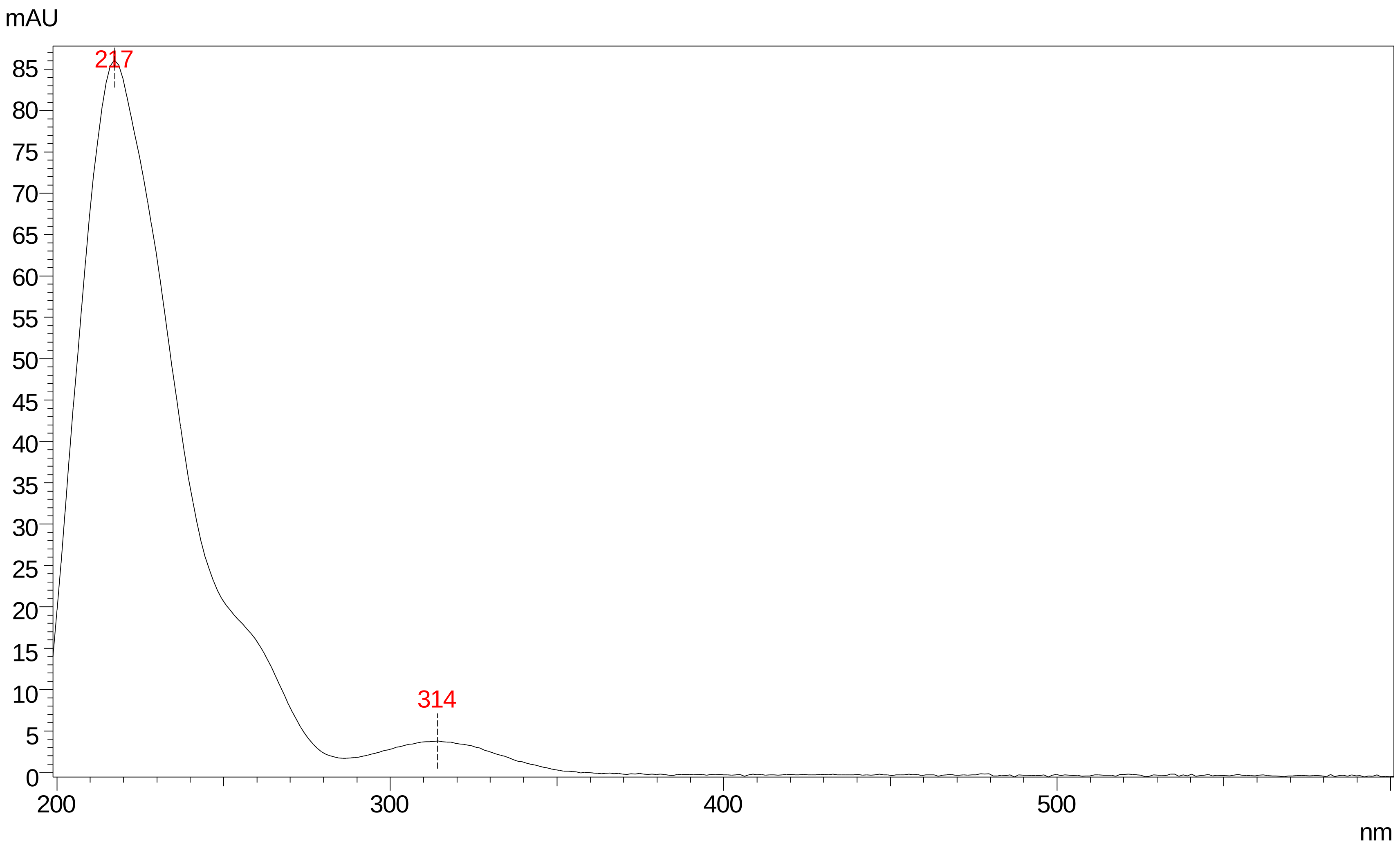

Fig. S92. UV spectrum of **27**

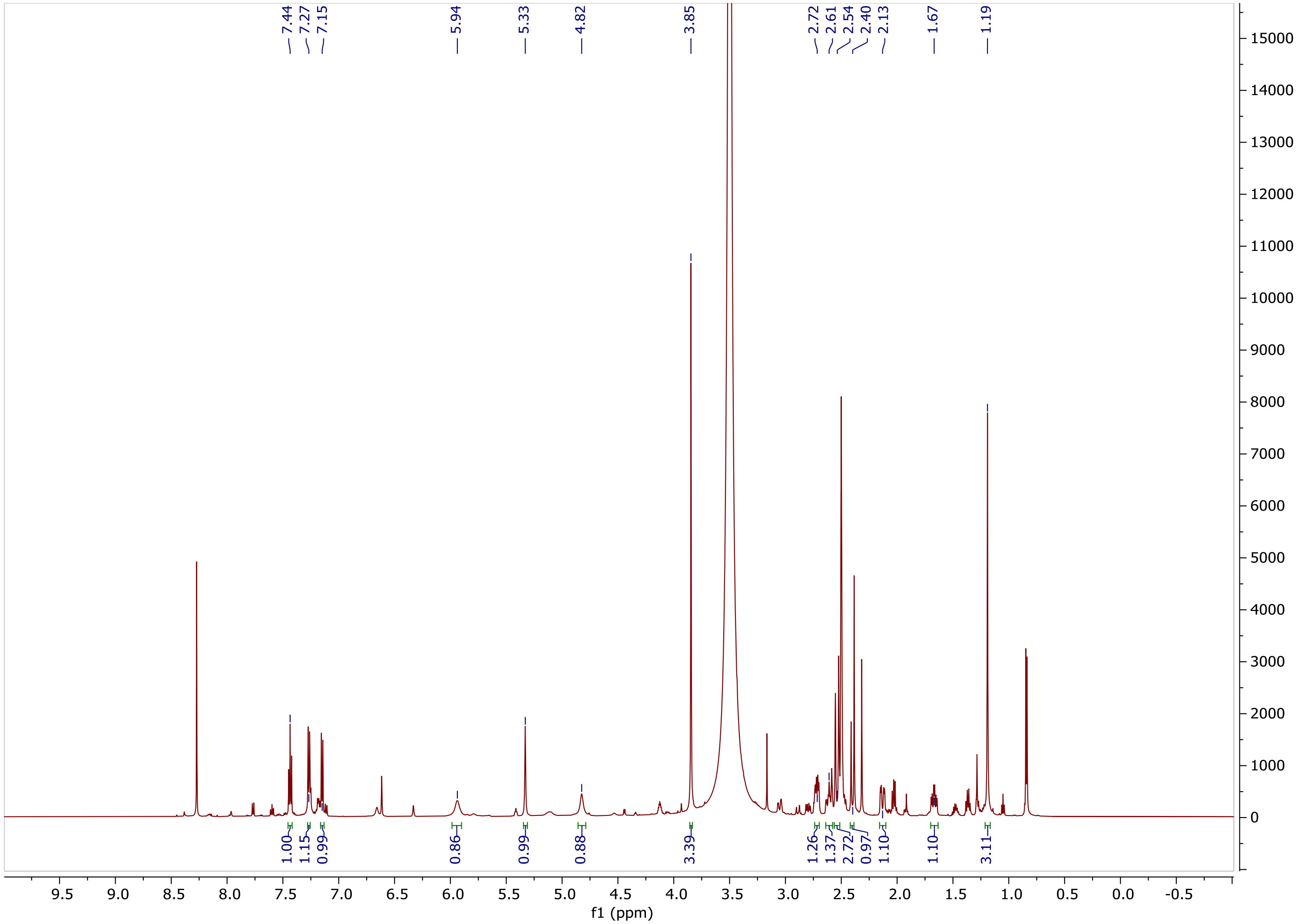

Fig. S93. ^{1}H NMR spectrum of **27** (600 MHz, in DMSO-*d6*)

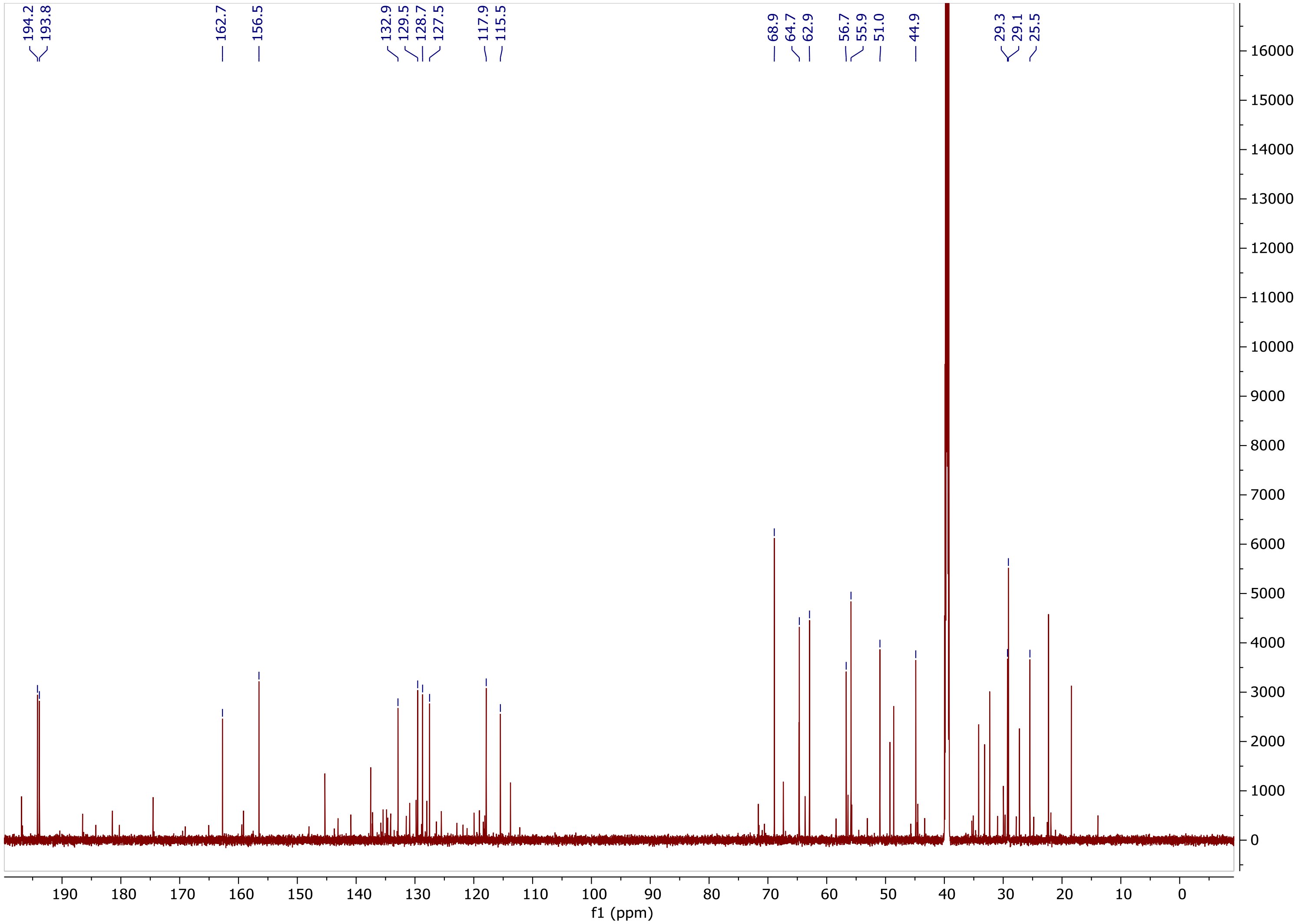

Fig. S94. ^{13}C NMR spectrum of **27** (213 MHz, in DMSO-*d6*)

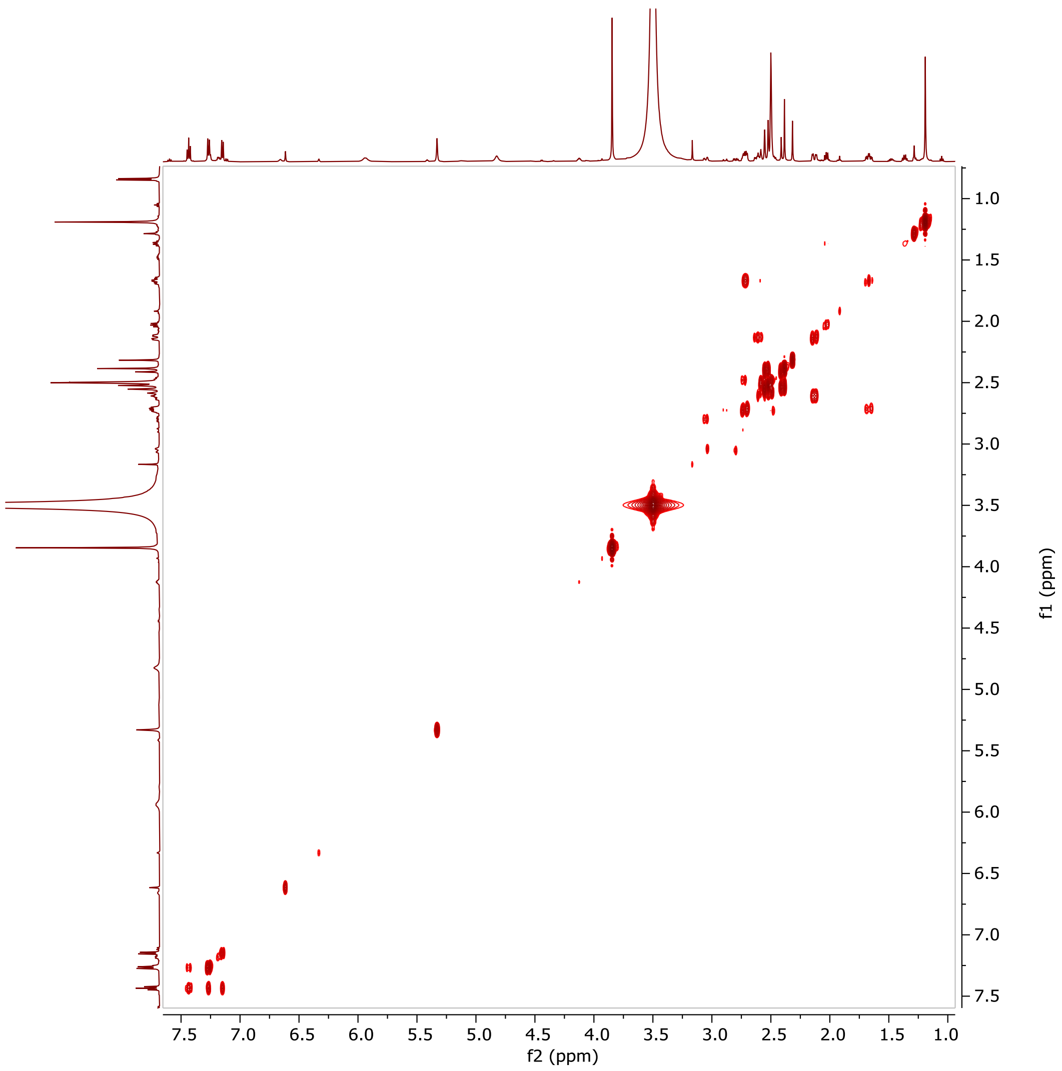

Fig. S95. COSY spectrum of **27** (600 MHz, in DMSO-*d6*)

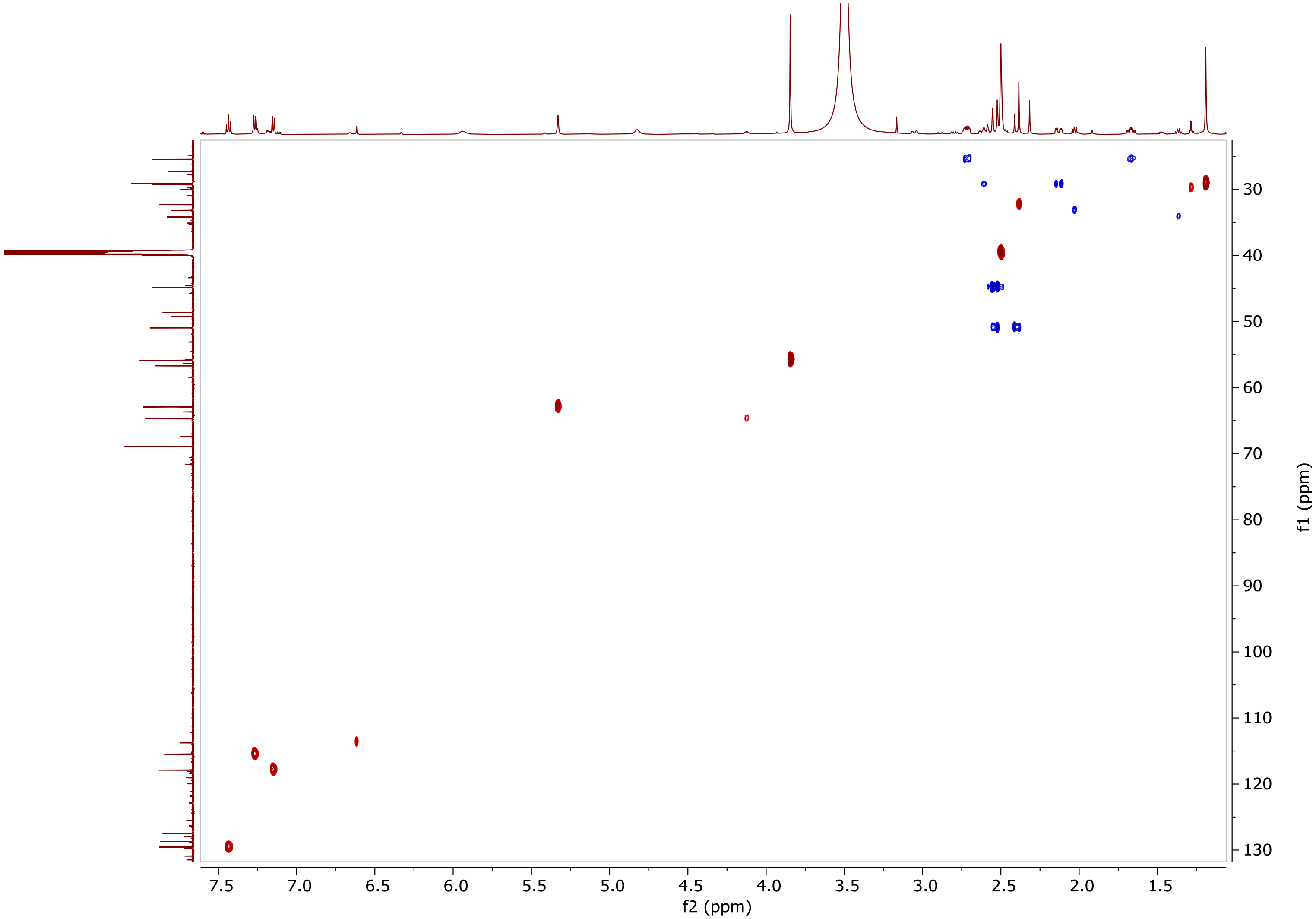

Fig. S96. Multiplicity-edited HSQC spectrum of **27** (600 MHz, in DMSO-*d6*)

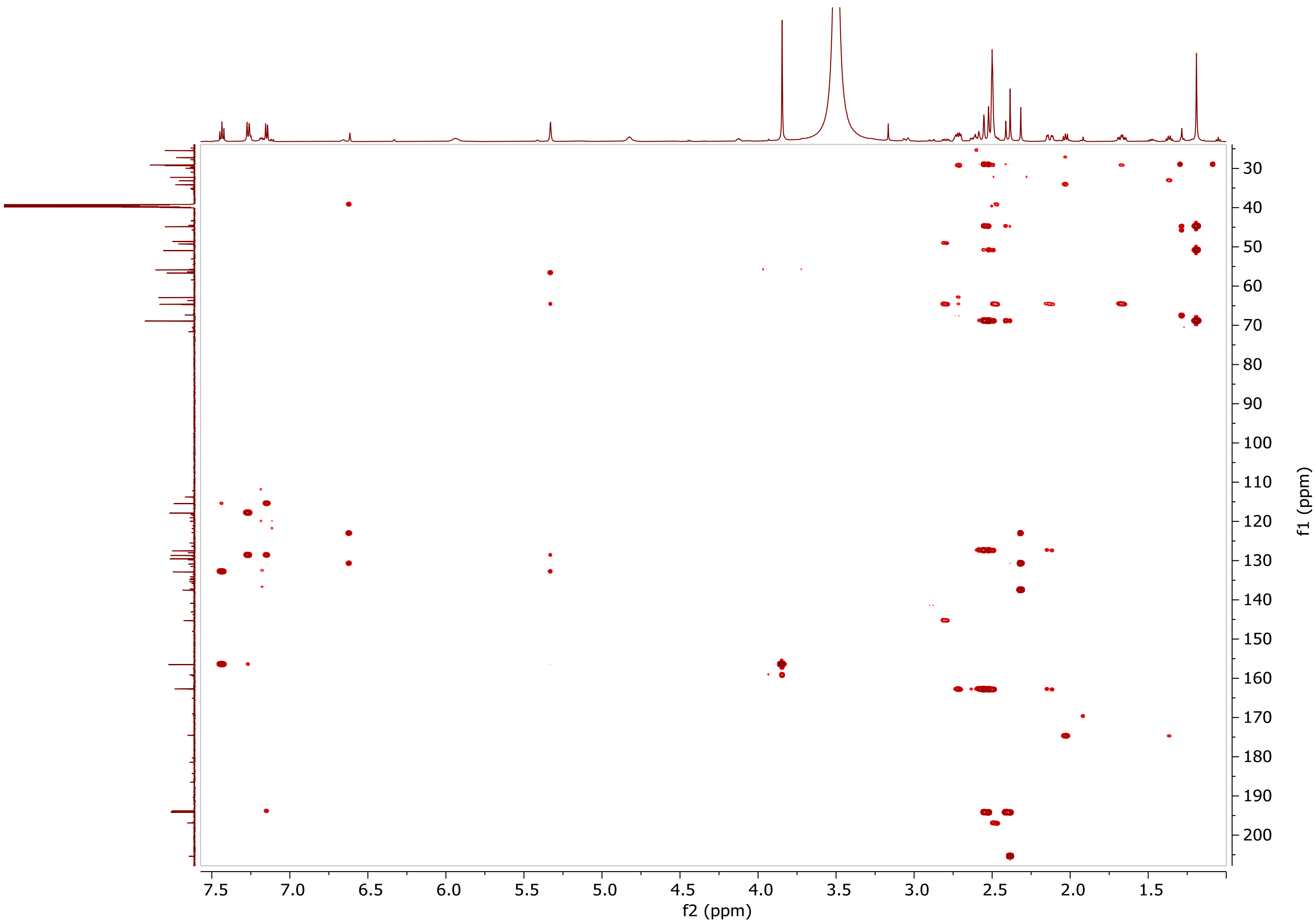

Fig. S97. HMBC spectrum of **27** (600 MHz, in DMSO-*d6*)

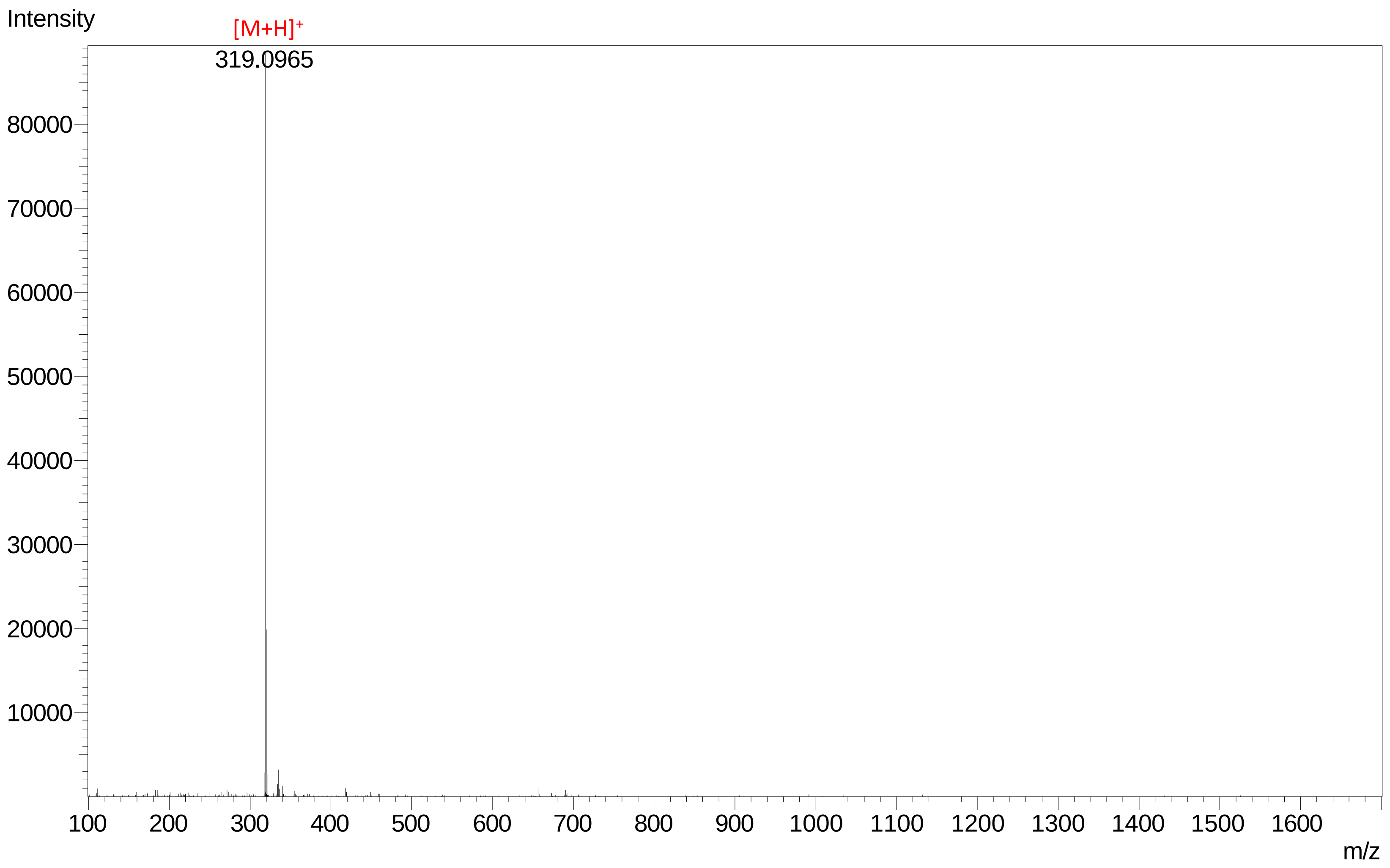

Fig. S98. (+)-HRESIMS spectrum of **5**

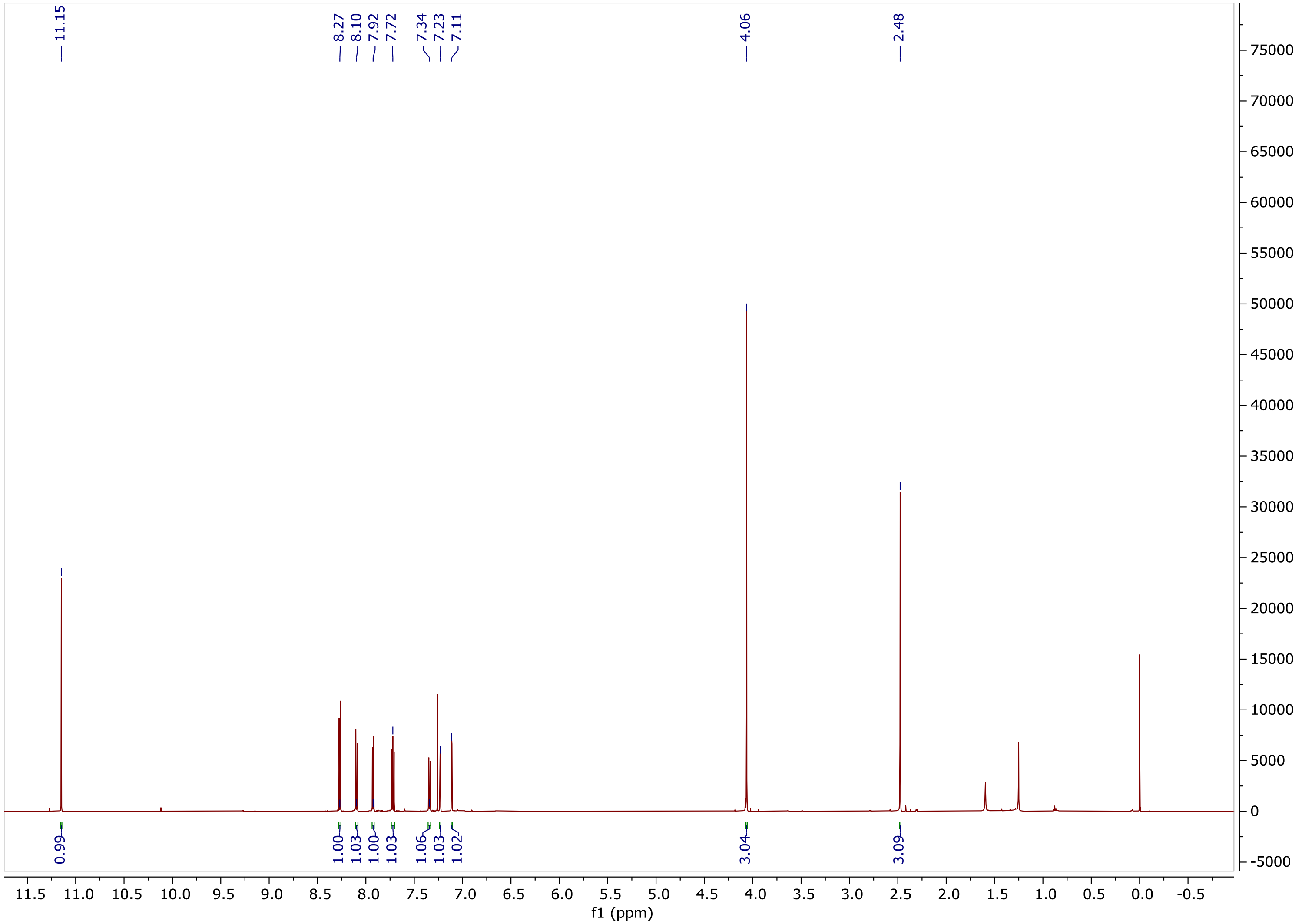

Fig. S99. ^{1}H NMR spectrum of **5** (600 MHz, in $CDCl_3$ with added TMS)

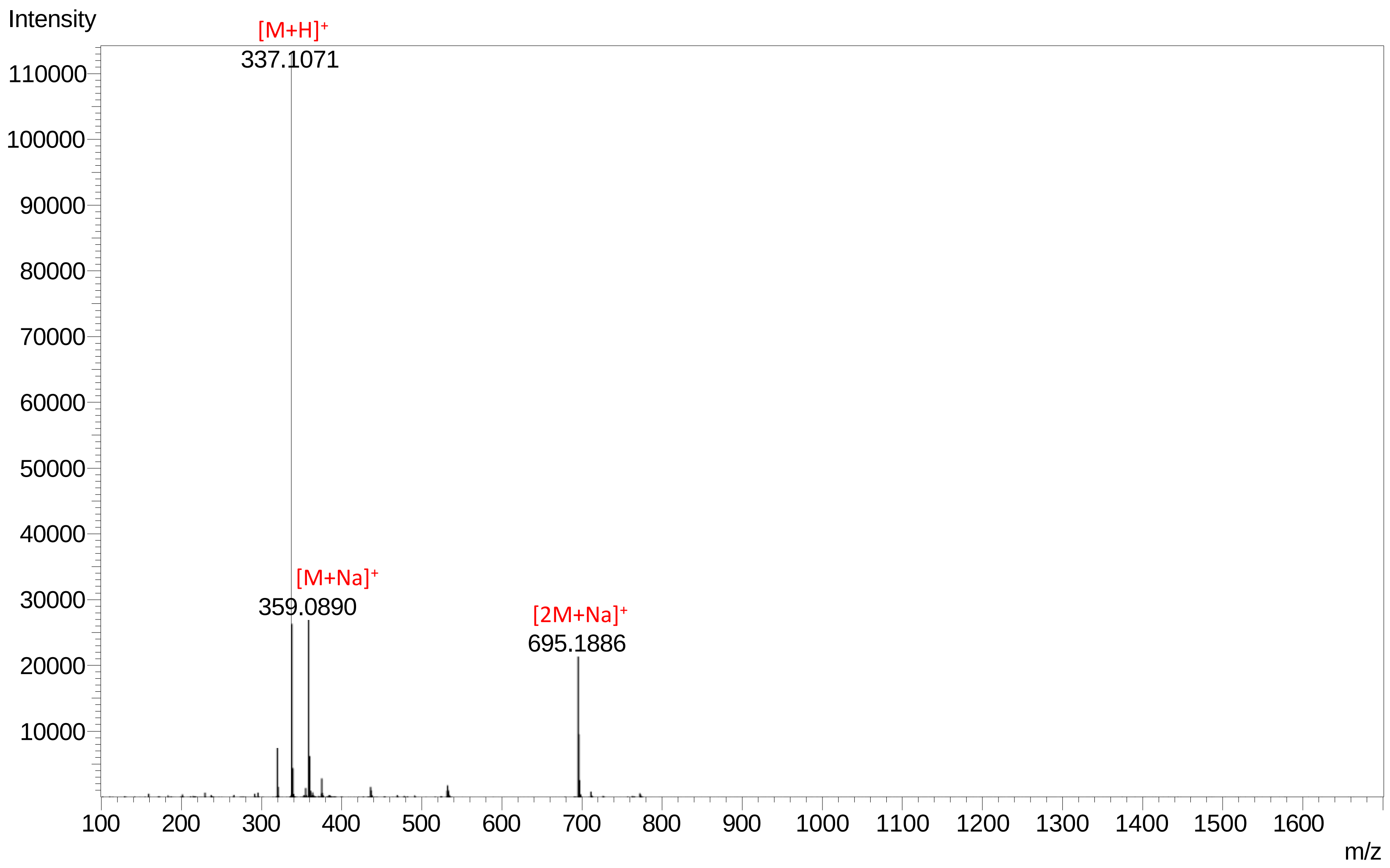

Fig. S100. (+)-HRESIMS spectrum of **6**

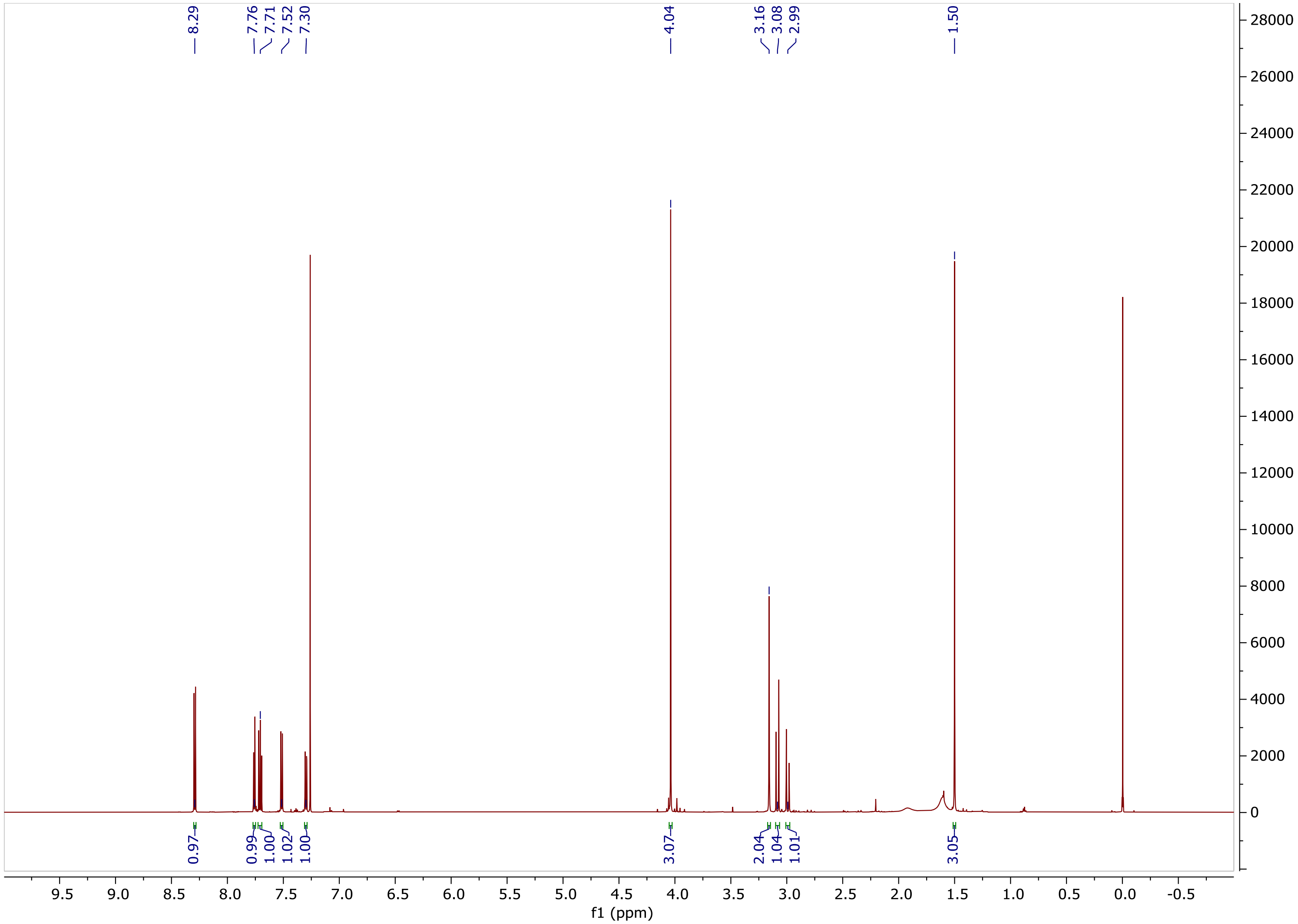

Fig. S101. ^{1}H NMR spectrum of **6** (600 MHz, in $CDCl_3$ with added TMS)

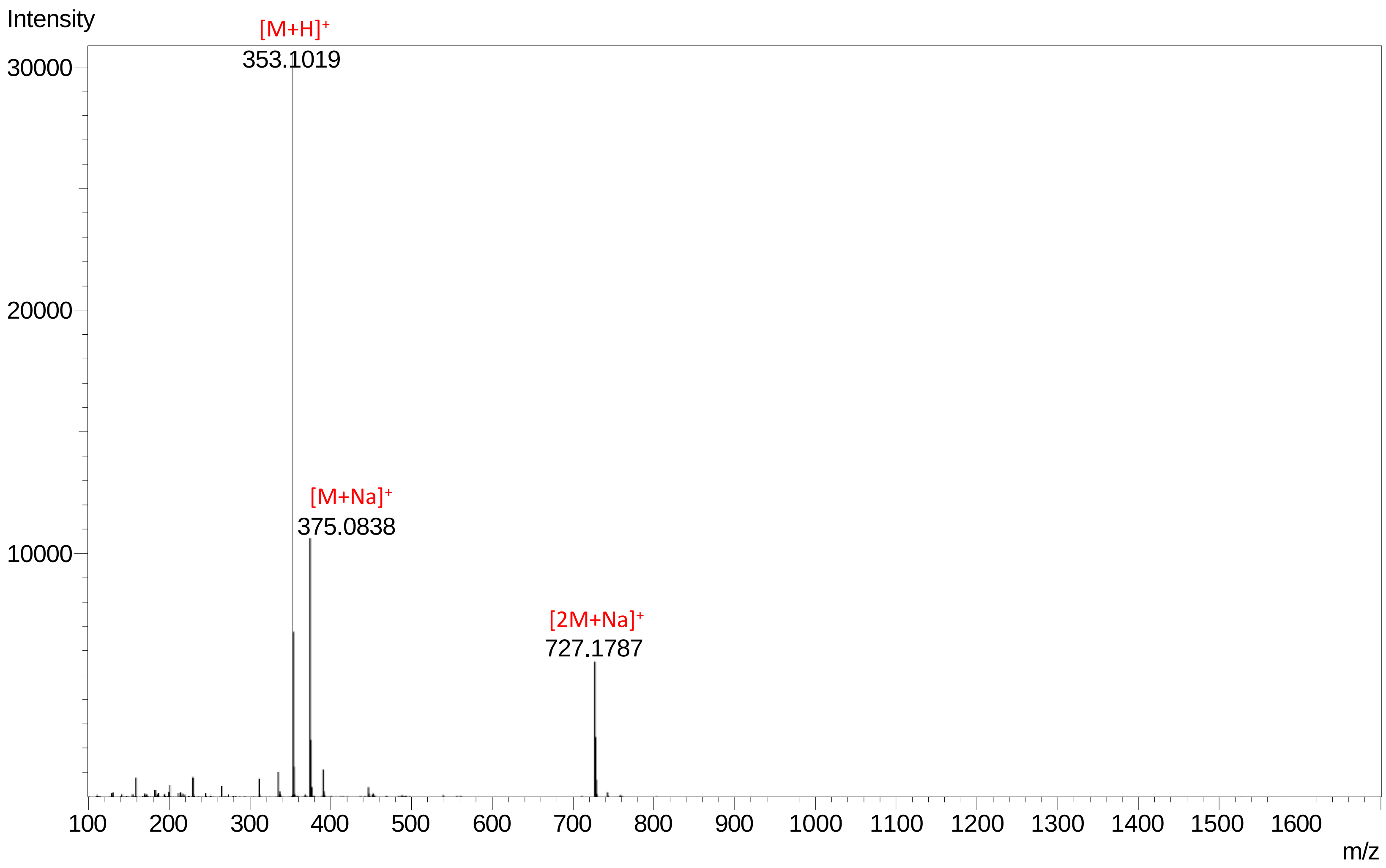

Fig. S102. (+)-HRESIMS spectrum of **7**

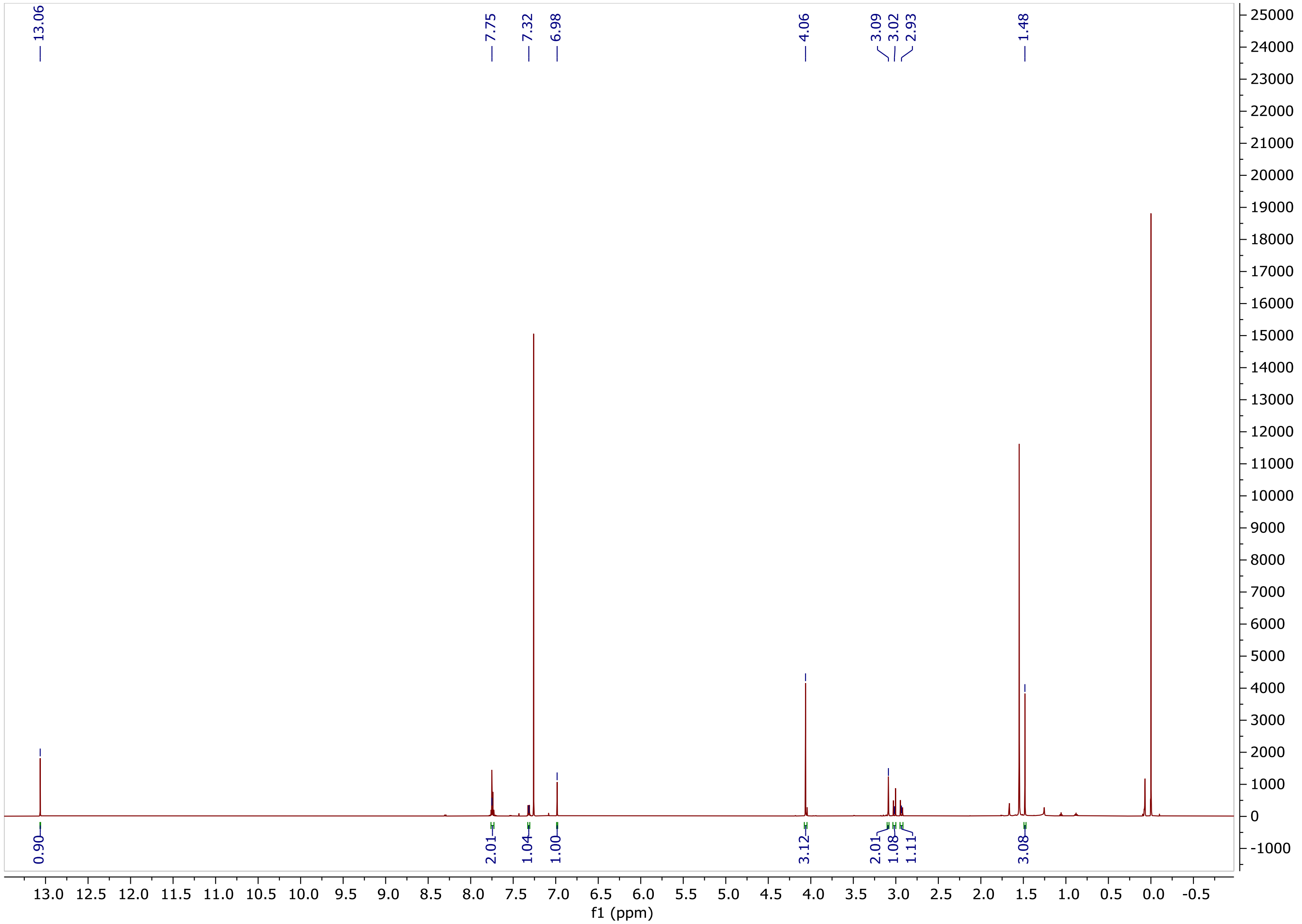

Fig. S103. ^1H NMR spectrum of **7** (600 MHz, in $CDCl_3$ with added TMS)

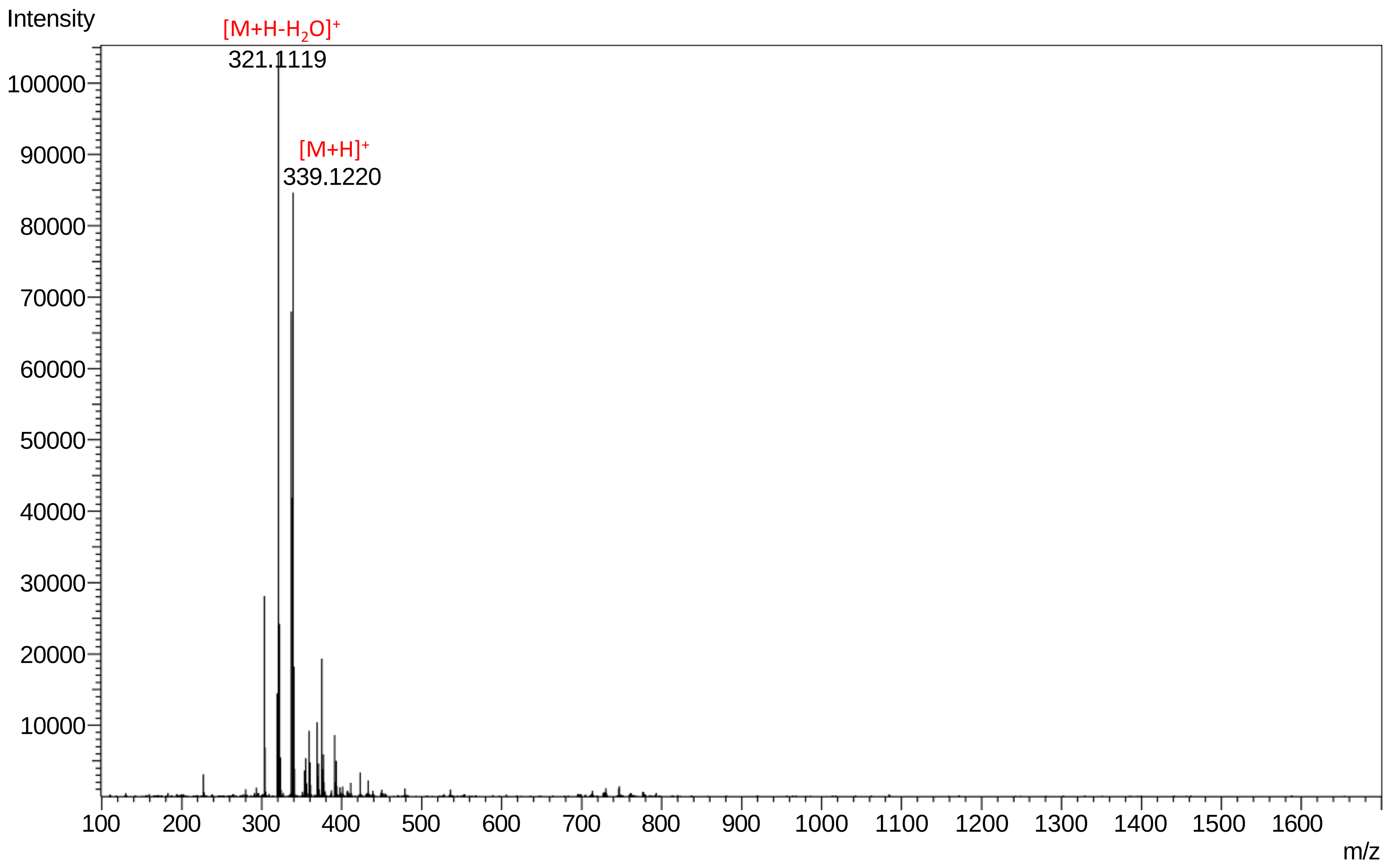

Fig. S104. (+)-HRESIMS spectrum of **15**

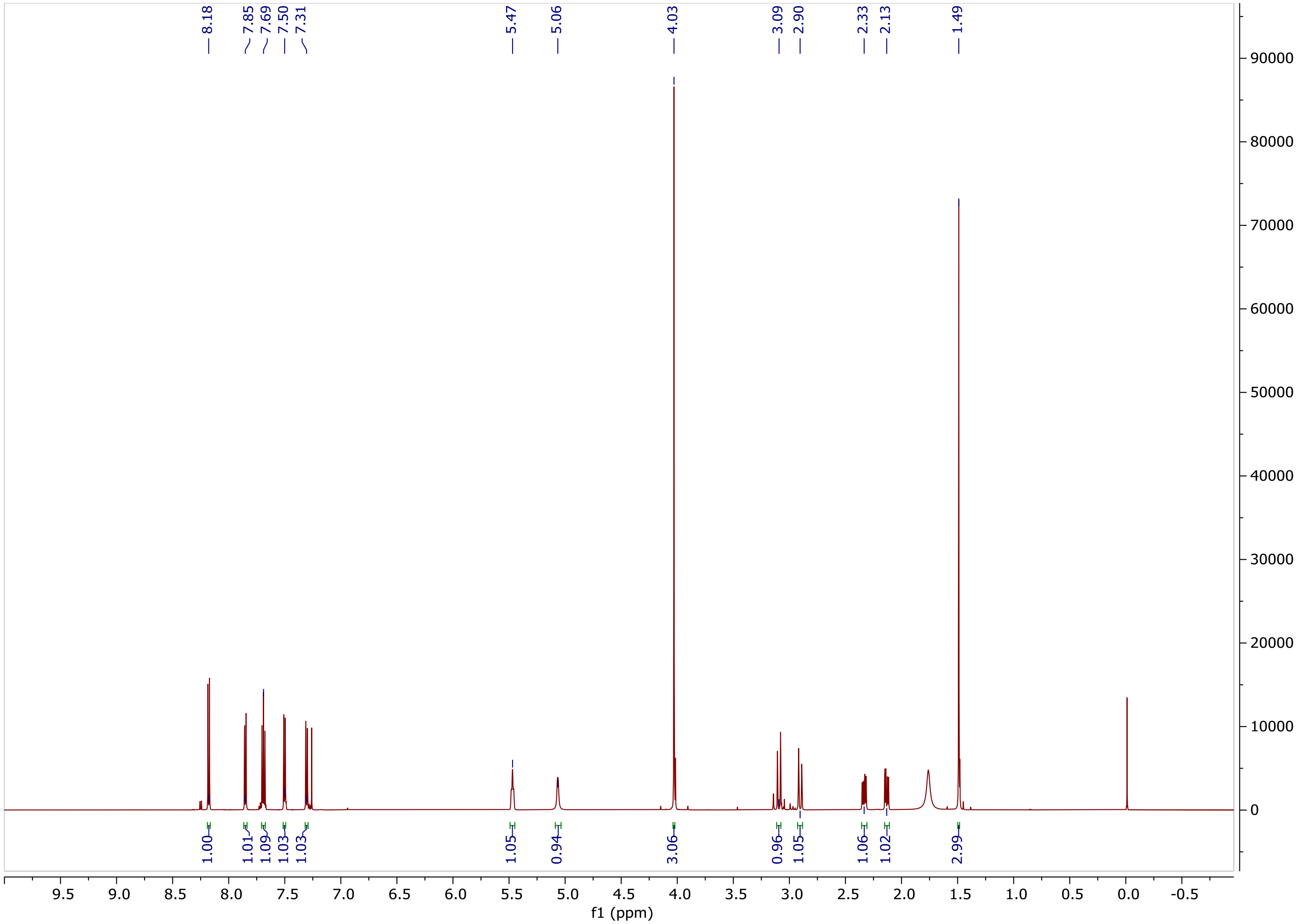

Fig. S105. ^{1}H NMR spectrum of **15** (600 MHz, in $CDCl_3$ with added TMS)

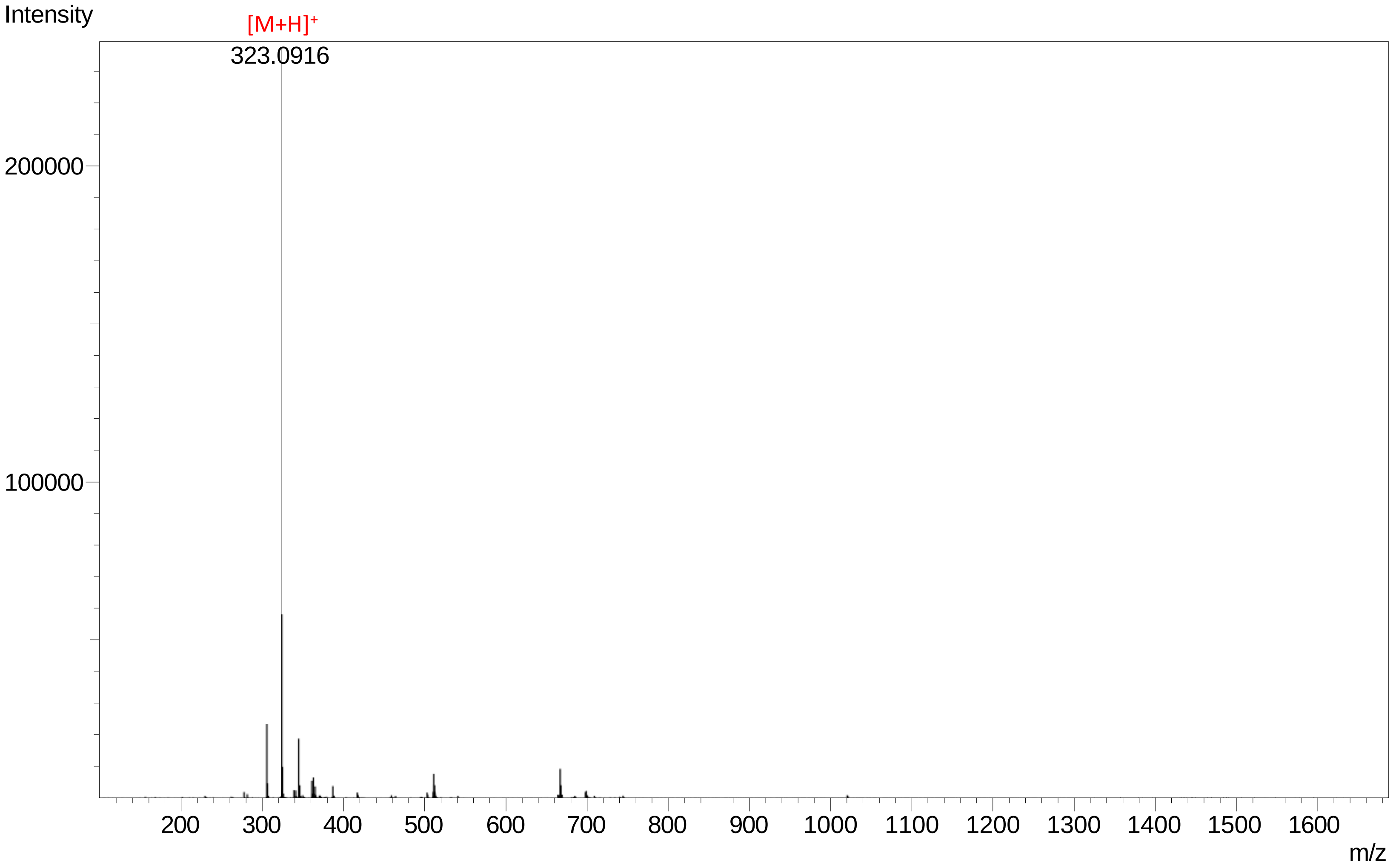

Fig. S106. (+)-HRESIMS spectrum of **28**

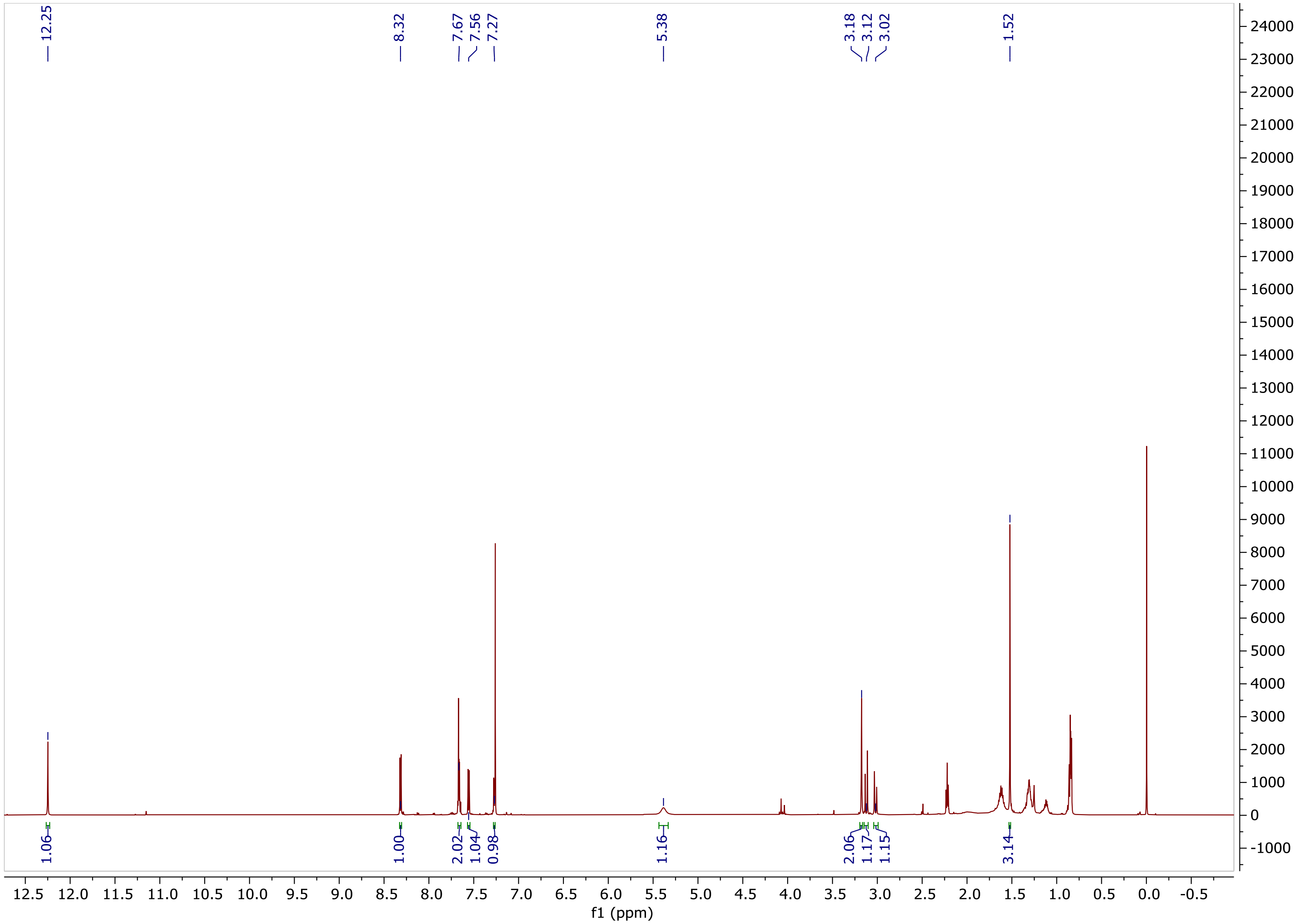

Fig. S107. ^{1}H NMR spectrum of **28** (600 MHz, in $CDCl_3$ with added TMS)

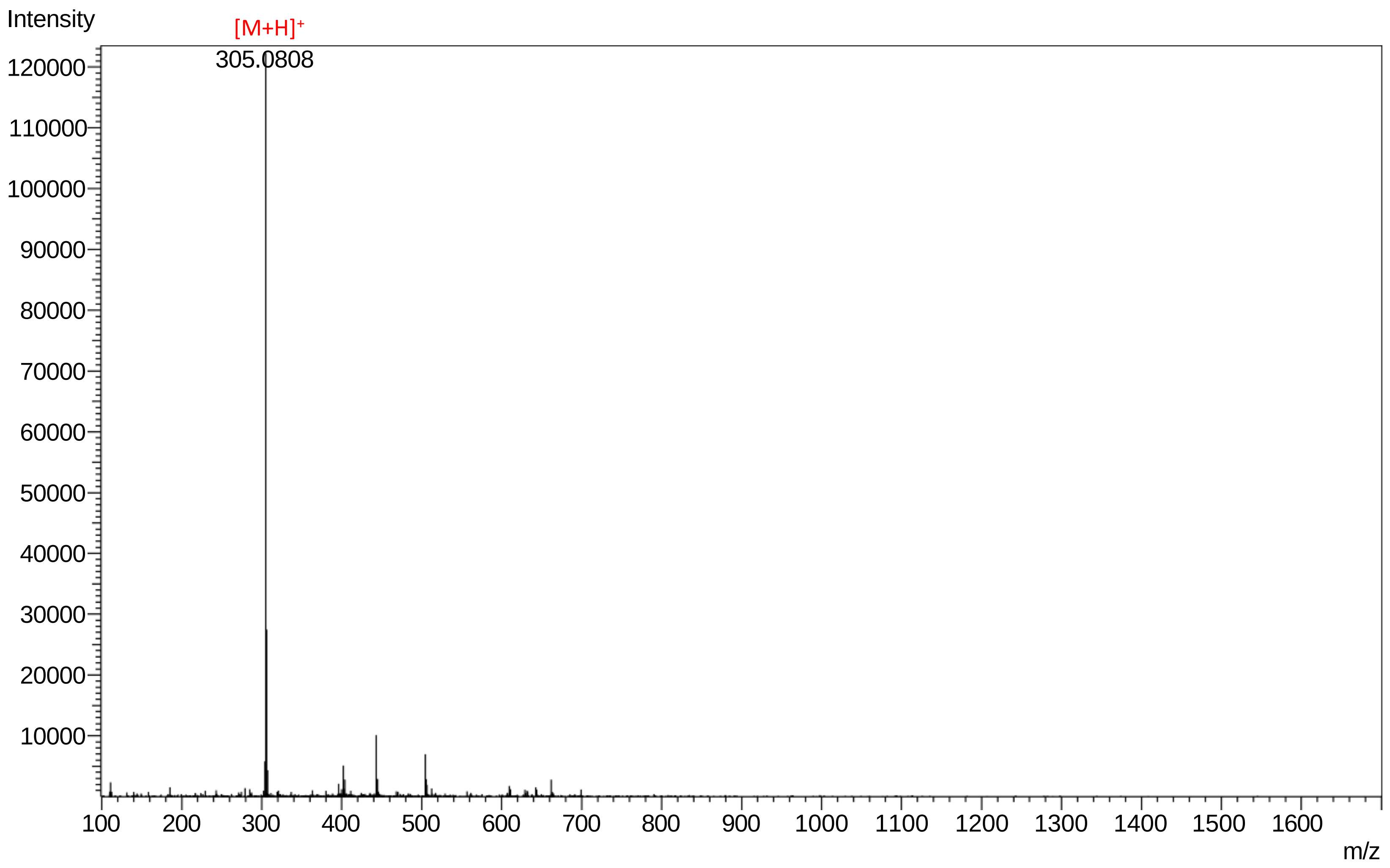

Fig. S108. (+)-HRESIMS spectrum of **29**

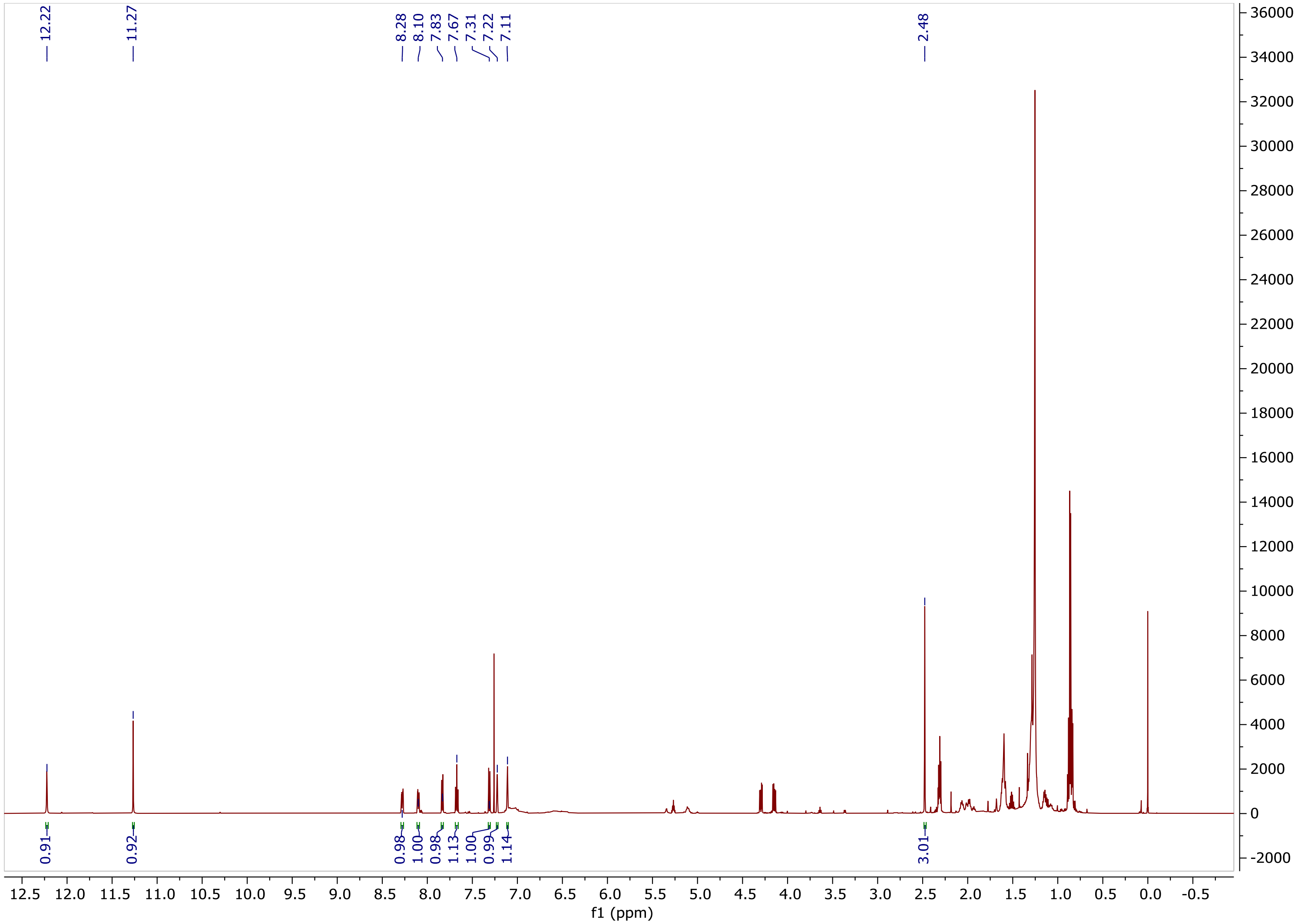

Fig. S109. ^{1}H NMR spectrum of **29** (600 MHz, in $CDCl_3$ with added TMS)

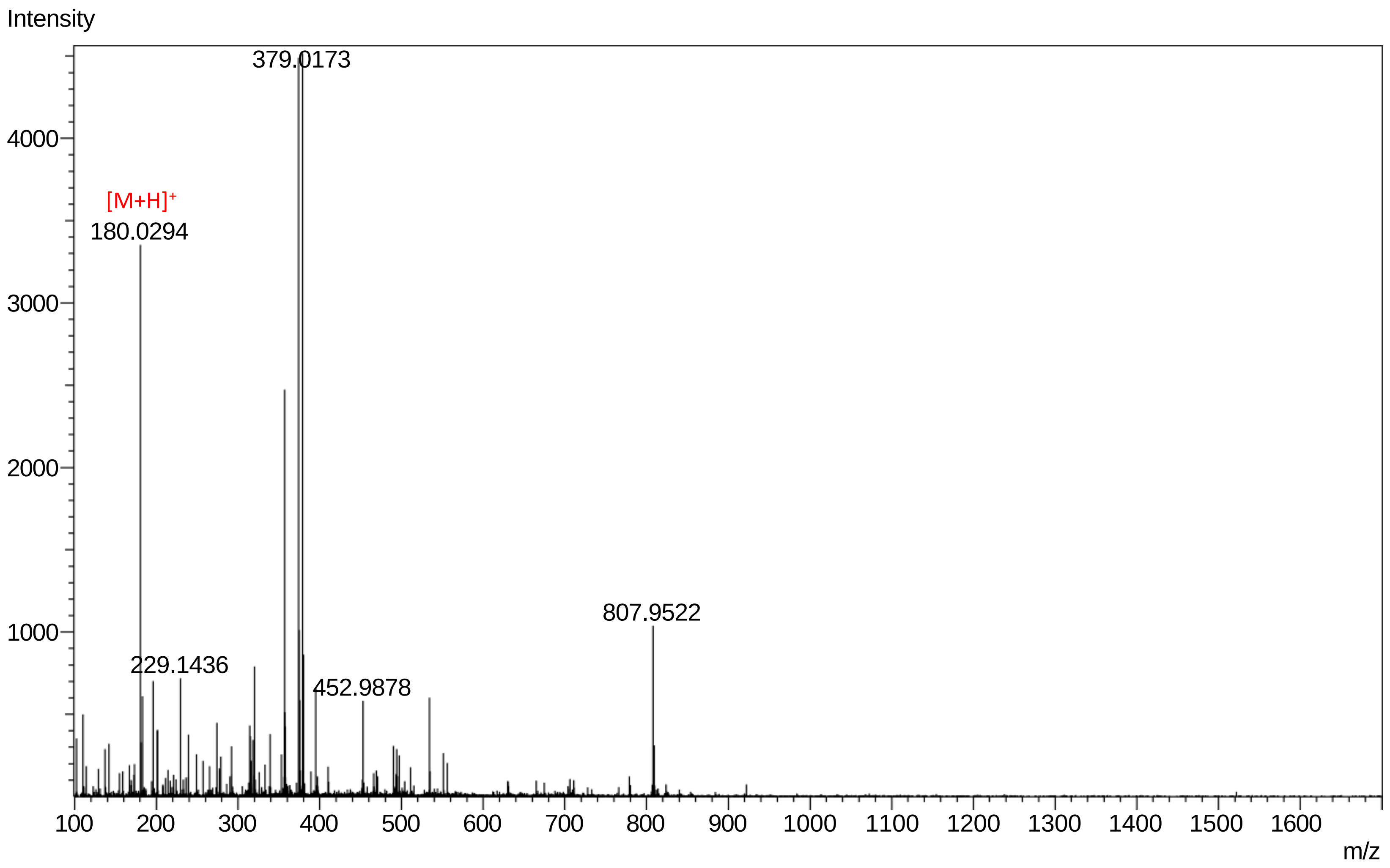

Fig. S110. (+)-HRESIMS spectrum of **30**

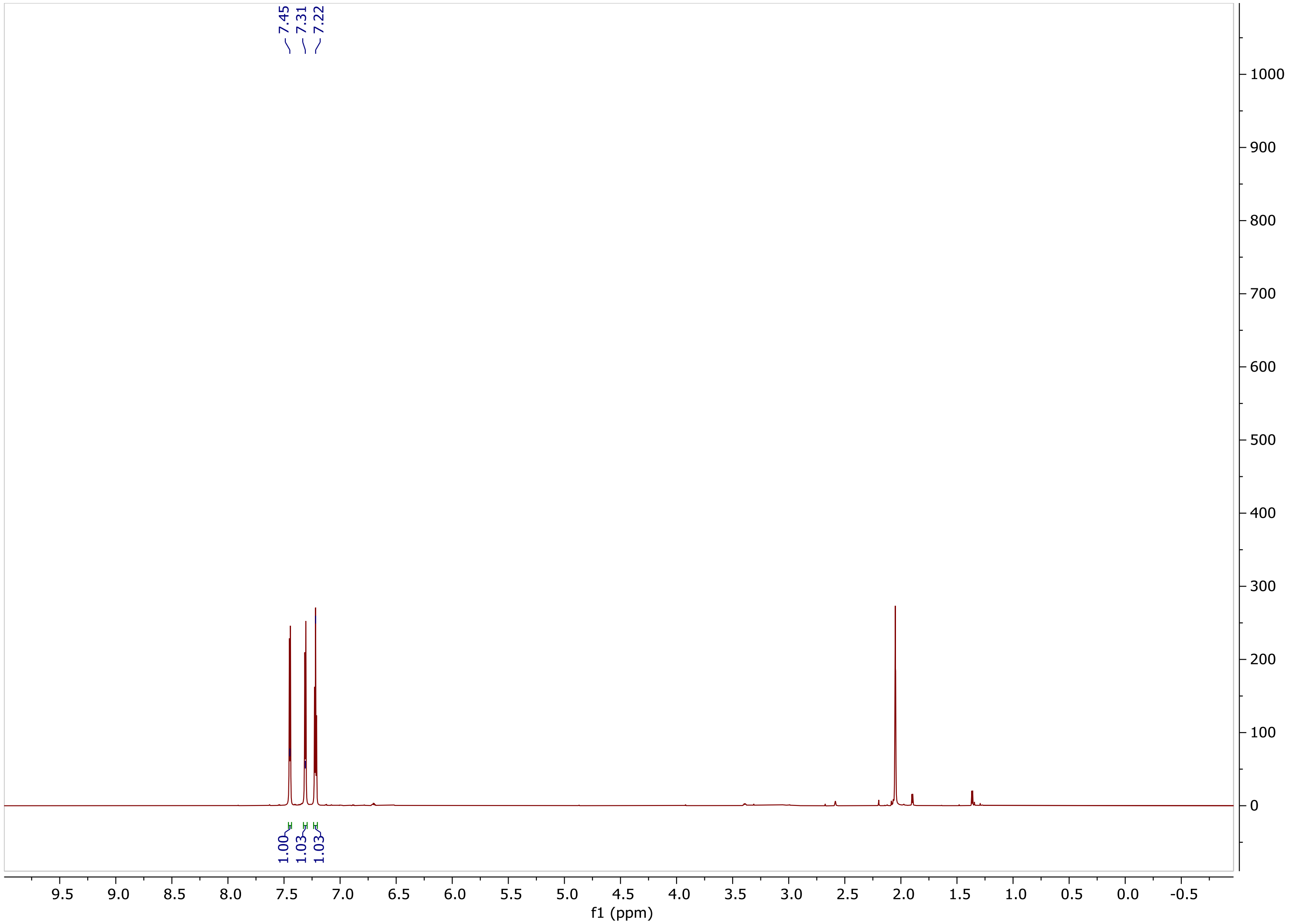

Fig. S111. ^{1}H NMR spectrum of **30** (600 MHz, in DMSO-*d6*)

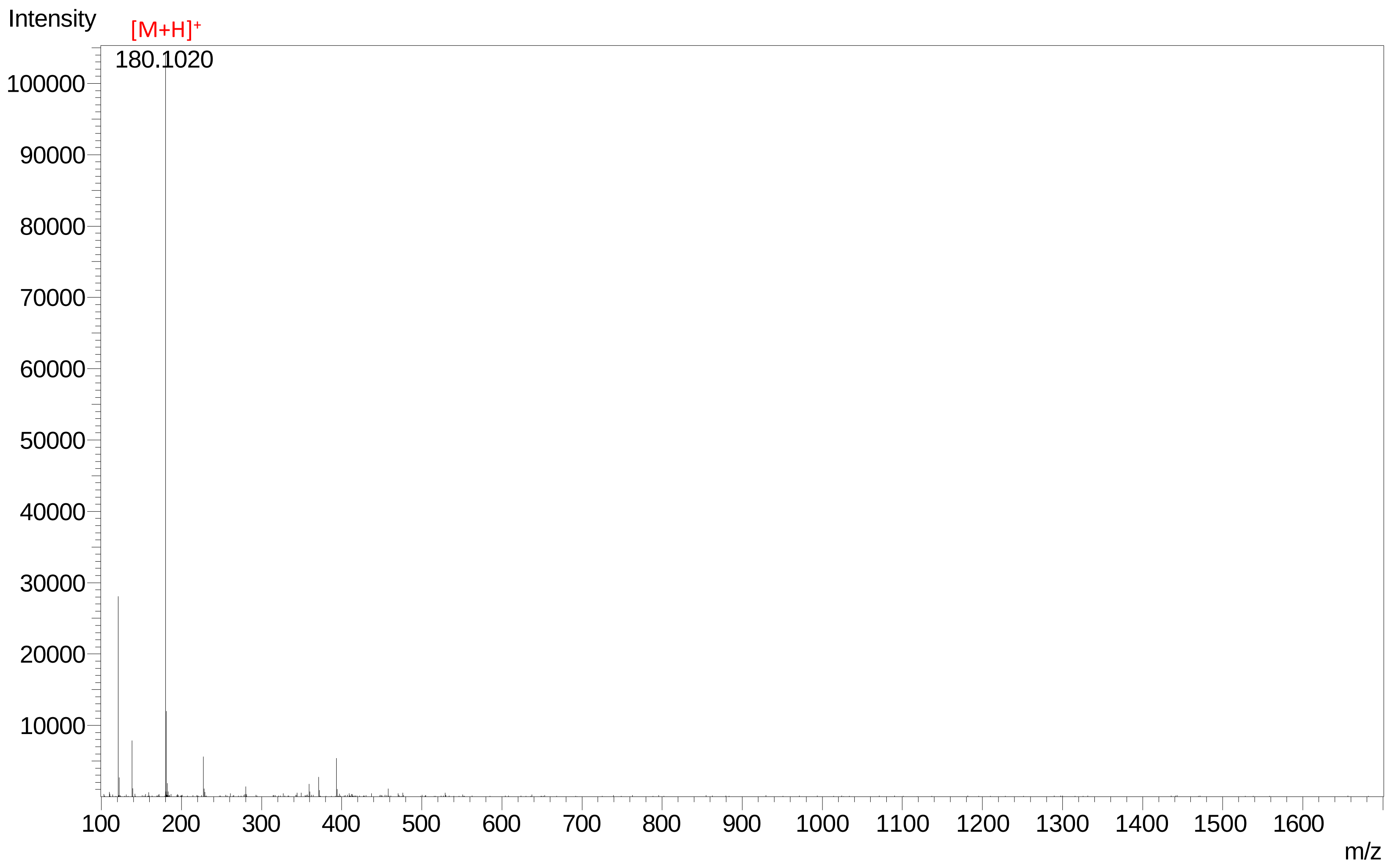

Fig. S112. (+)-HRESIMS spectrum of **31**

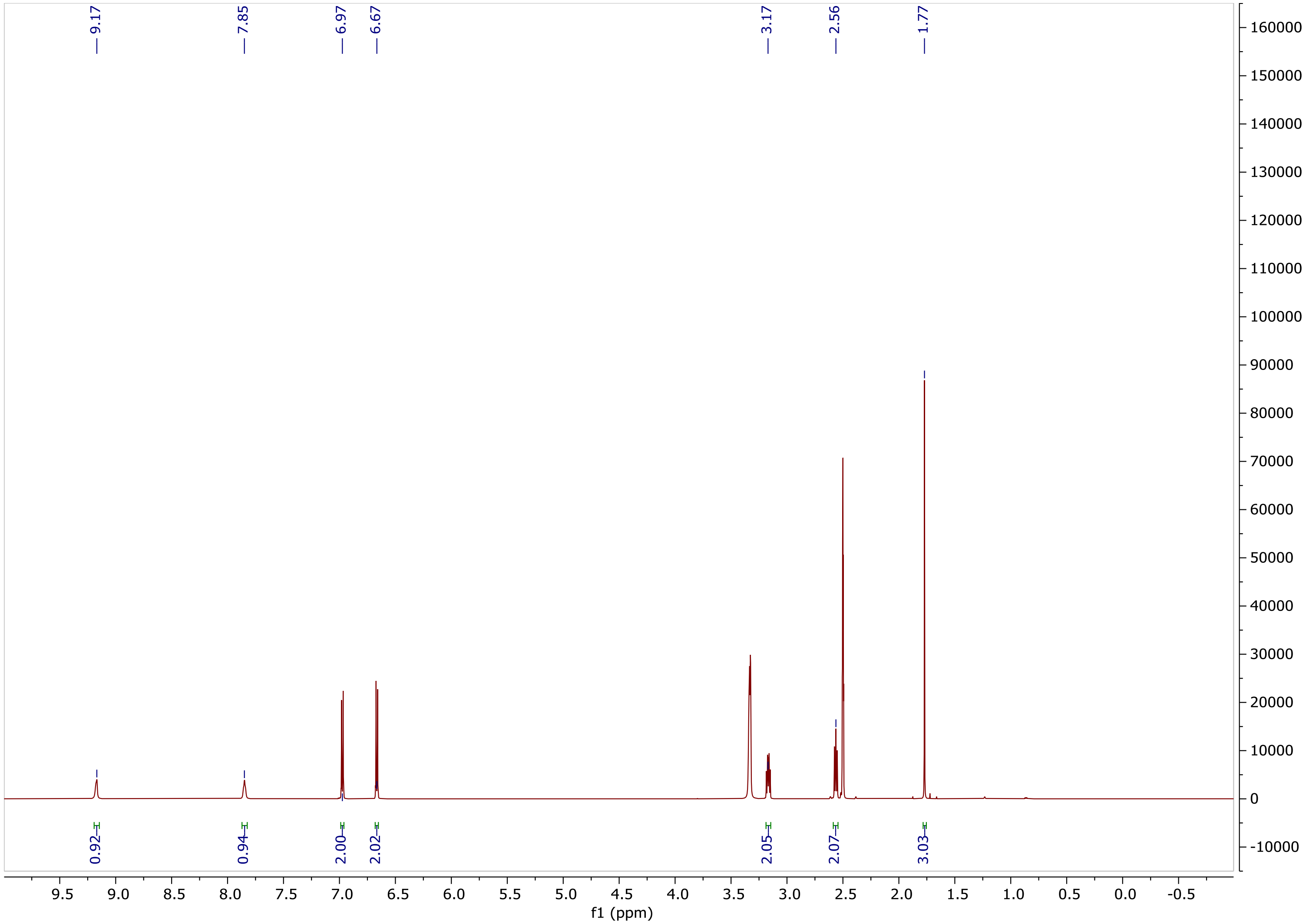

Fig. S113. ^{1}H NMR spectrum of **31** (600 MHz, in DMSO-*d6*)

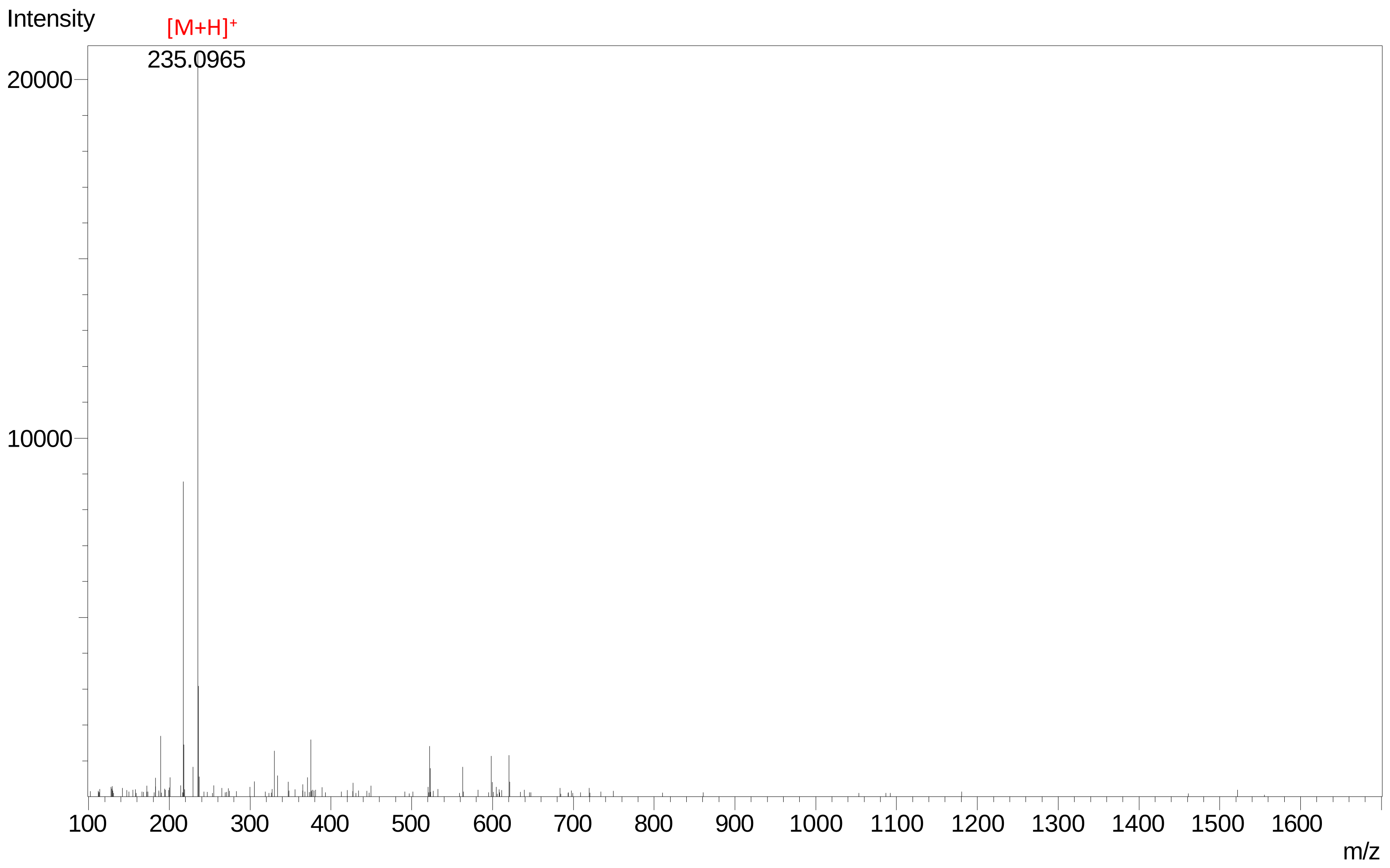

Fig. S114. (+)-HRESIMS spectrum of **32**

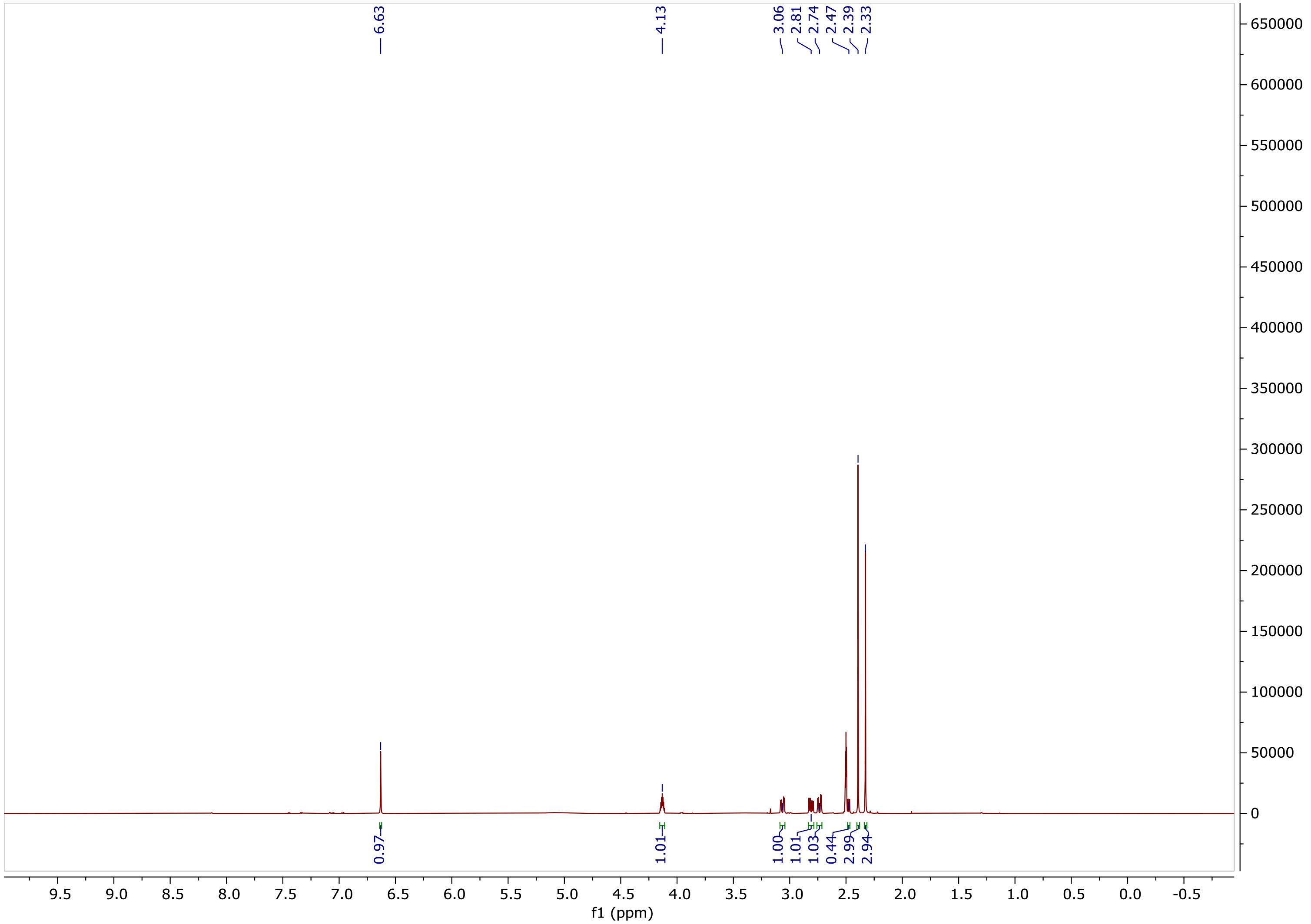

Fig. S115. ^{1}H NMR spectrum of **32** (600 MHz, in DMSO-*d6*)

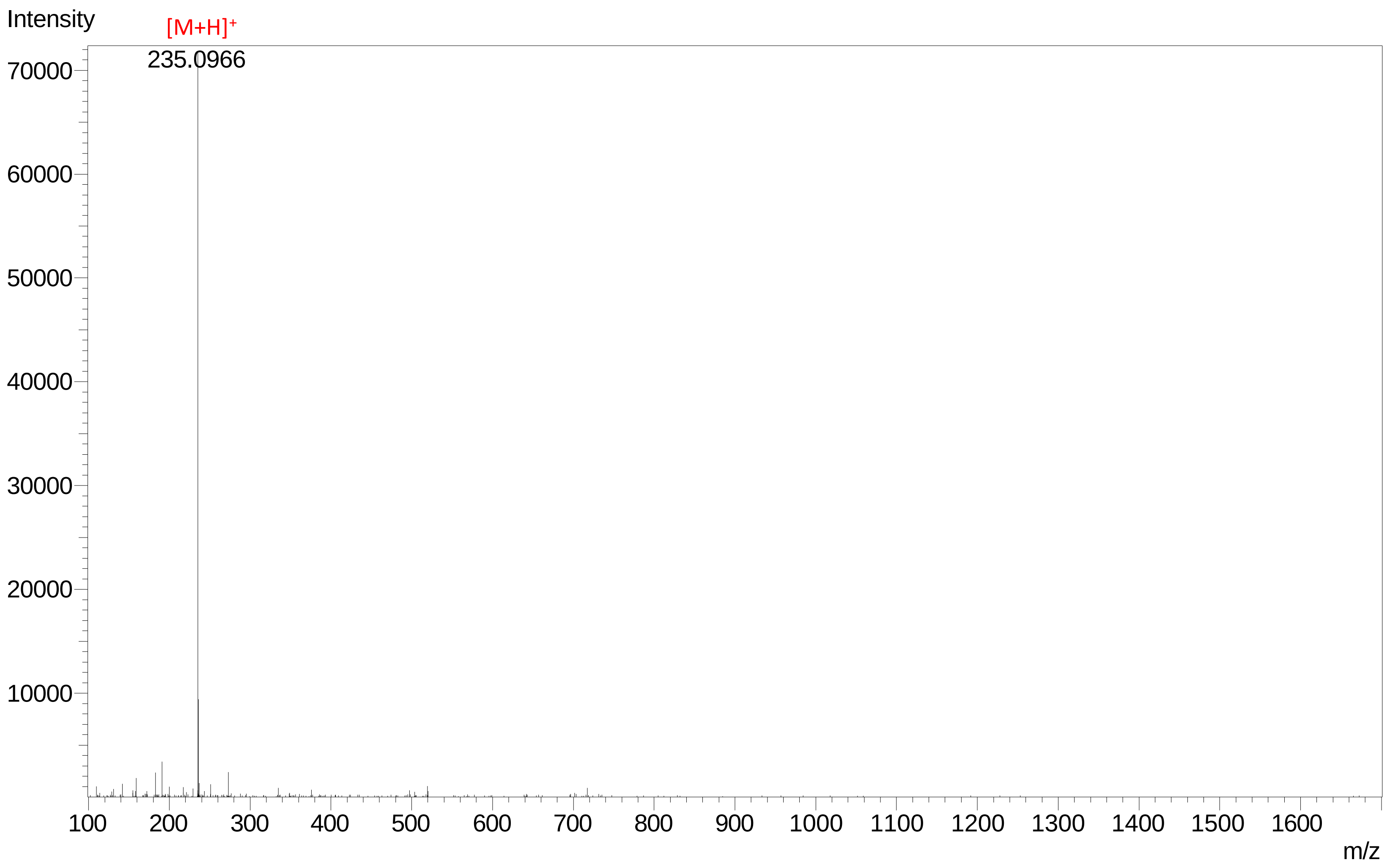

Fig. S116. (+)-HRESIMS spectrum of **33**

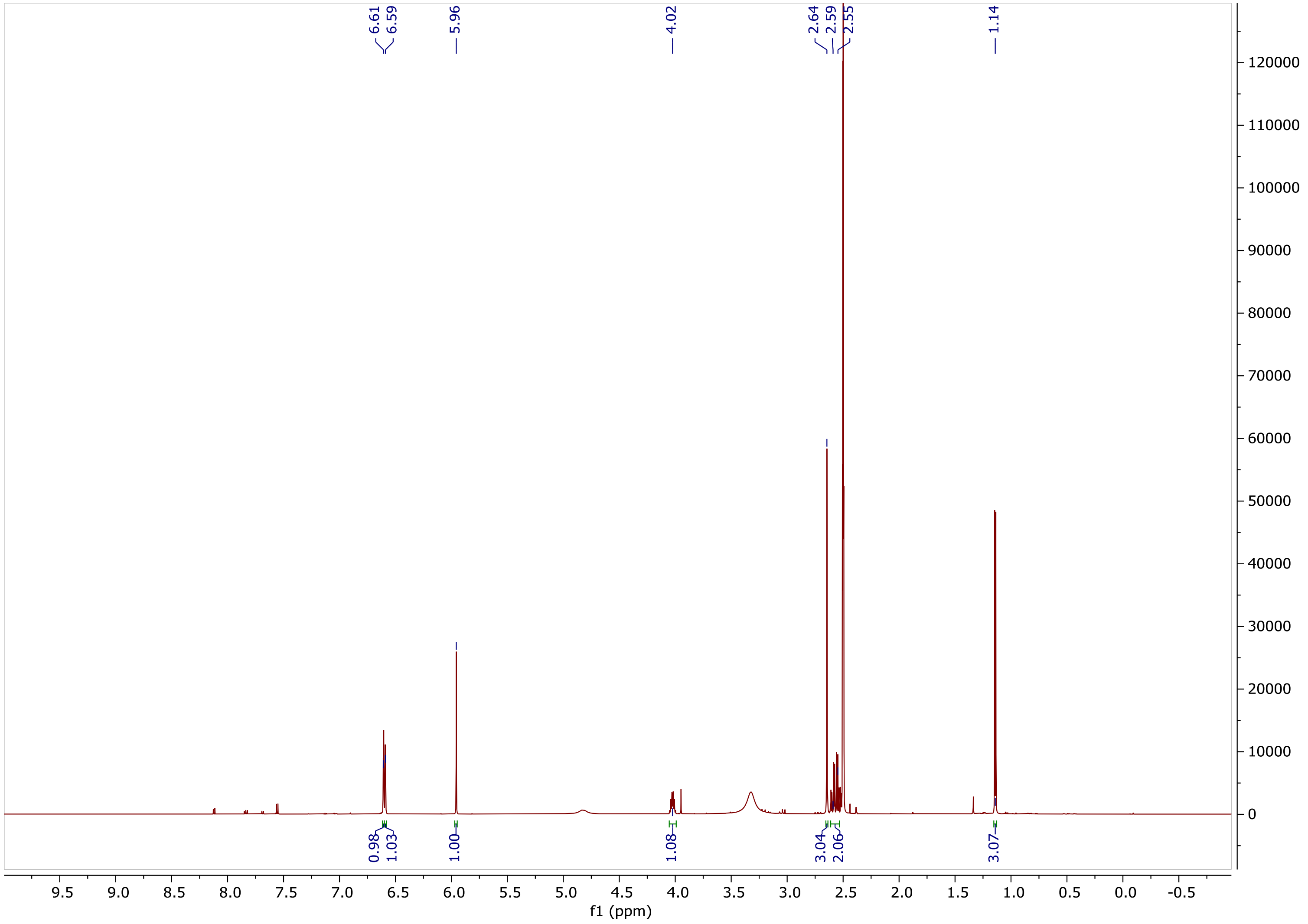

Fig. S117. ^{1}H NMR spectrum of **33** (600 MHz, in DMSO-*d6*)
